# Discovery of novel enasidenib analogues targeting inhibition of mutant isocitrate dehydrogenase 2 as antileukaemic agents

**DOI:** 10.1080/14756366.2022.2157411

**Published:** 2023-01-11

**Authors:** Ahmed F. Khalil, Tarek F. El-Moselhy, Eman A. El-Bastawissy, Rasha Abdelhady, Nancy S. Younis, Mervat H. El-Hamamsy

**Affiliations:** a Department of Pharmaceutical Chemistry, Faculty of Pharmacy, Tanta University, Tanta, Egypt; b Department of Pharmacology and Toxicology, Faculty of Pharmacy, Fayoum University, Fayoum, Egypt; c Department of Pharmaceutical Sciences, College of Clinical Pharmacy, King Faisal University, Al Hofuf, Saudi Arabia

**Keywords:** Synthesis, triazine, hydrazine, IDH2, enasidenib, leukaemia, anticancer

## Abstract

Mutant isocitrate dehydrogenase (IDH) 2 “IDH2m” acquires a neo-enzymatic activity reducing α-ketoglutarate to an oncometabolite, D-2-hydroxyglutarate (2-HG). Three *s*-triazine series were designed and synthesised using enasidenib as a lead compound. *In vitro* anticancer screening *via* National Cancer Institute “NCI” revealed that analogues **6a, 6c**, **6d**, **7g,** and **7l** were most potent, with mean growth inhibition percentage “GI%” = 66.07, 66.00, 53.70, 35.10, and 81.15, respectively, followed by five-dose screening. Compounds **6c, 6e,** and **7c** were established as the best IDH2^R140Q^ inhibitors compared to enasidenib, reporting IC_50_ = 101.70, 67.01, 88.93, and 75.51 nM, respectively. More importantly, **6c, 6e,** and **7c** displayed poor activity against the wild-type IDH2, IC_50_ = 2928, 2295, and 3128 nM, respectively, which implementing high selectivity and accordingly safety. Furthermore, **6c** was screened for cell cycle arrest, apoptosis induction, and western blot analysis. Finally, computational tools were applied to predict physicochemical properties and binding poses in IDH2^R140Q^ allosteric site.

## Introduction

**Figure 1.**
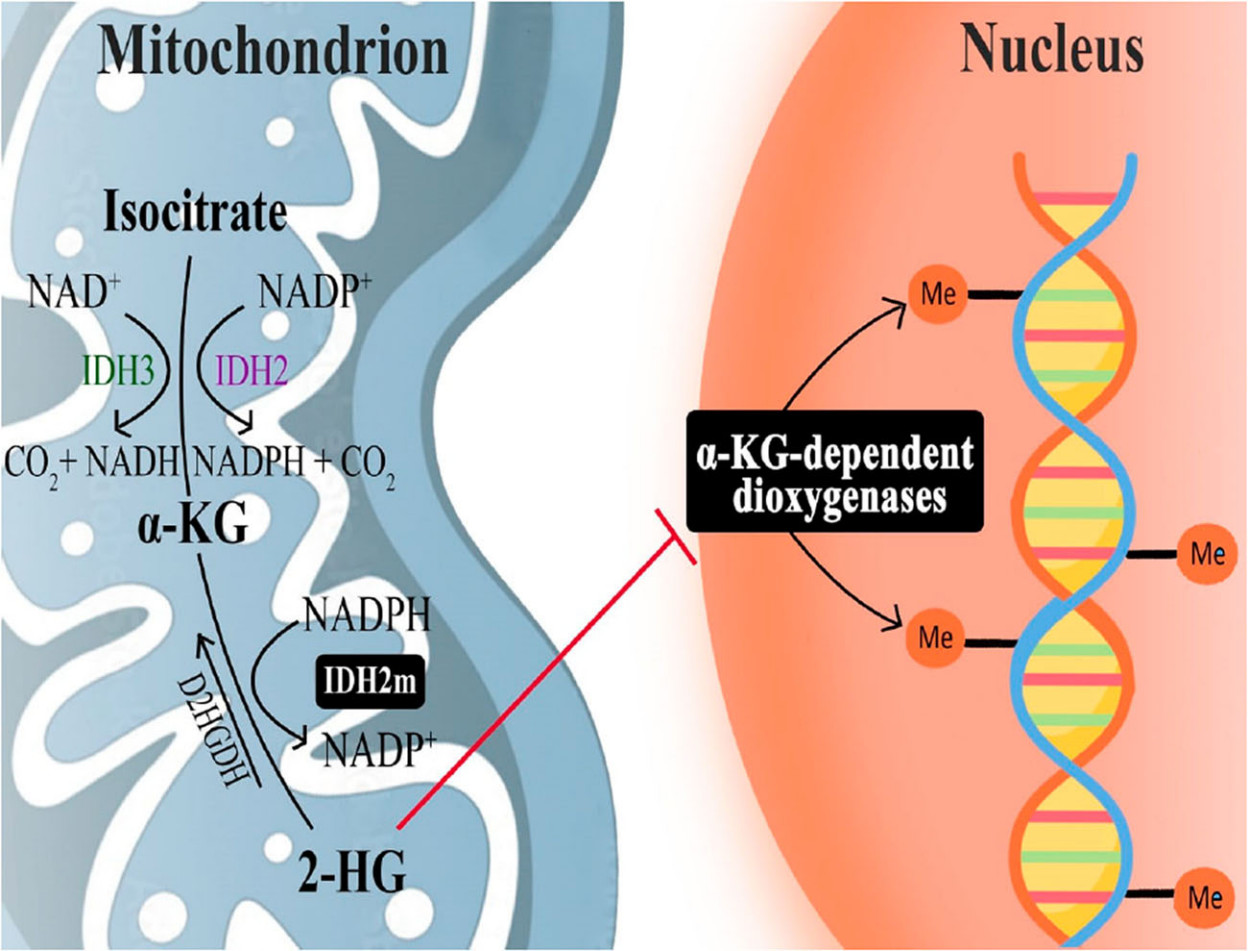
Function of wild-type IDH in homeostasis and activity of mutant IDH in disease. α-KG, α-ketoglutarate; 2-HG, D-2-hydroxyglutarate; D2HGDH, D-2-hydroxyglutarate dehydrogenase; IDH, isocitrate dehydrogenase; IDH2m, mutant IDH2.

Cancer is considered one of the most threatening diseases devasting the human health. It is expected that by 2040, the number of new cancer cases rise to 29.5 million per year and to 16.4 million cancer-related deaths^1^. Global Cancer Observatory estimated 474 519 new cases of leukaemia worldwide and 5231 in Egypt in the latest estimate^2^. Acute myeloid leukaemia (AML) is a type of leukaemia in which bone marrow produces large number of abnormal blood cells. It is characterised by clonal enlargement of myeloid, which forms all blood cells, with reduced capacity for differentiation. Once it’s a build-up in the bone marrow, causes restriction of traditional blood cells production^3^. According to American Cancer Society (ACS), AML is considered the most common cancer type of leukaemia in adult and represents 1 out of 3 cases diagnosed with leukaemia in childhood and teens^4^. The evolution of new efficient therapeutic agents with anticancer properties to overcome this condition is a major objective in medicinal chemistry. It is well established that tumour initiation and maintenance are dependent on metabolic reprogramming of cancer cell^5–7^. This means, the tumour cells use altered metabolic pattern compared to normal tissues^5^^,^^8^. Isocitrate dehydrogenase (IDH) is a vital metabolic enzyme in the Krebs cycle. It has three isoforms, IDH1 found in cytoplasm, IDH2, and IDH3 located in mitochondria^9^. Normally, IDH2 converts isocitrate by oxidative decarboxylation to alpha-ketoglutarate (α-KG) using NADP^+^ or NAD^+^ as cofactors^10^. Point mutations in the active site of IDH2 affecting Arg140 or Arg172 (R140 or R172) result in multiple tumours including; low-grade gliomas, secondary glioblastomas (GBM), angioimmunoblastic T-cell lymphomas, myelodysplastic syndrome (MDS), and AML^11–13^. The mutant IDH2 acquires a neo-enzymatic activity that reduces α-KG by NAPH to the oncometabolite called, D-2-hydroxyglutarate (2-HG)^14^. In consequence, accumulation of high levels of 2-HG competitively inhibit dioxygenases like histone and DNA demethylases and proteins that regulate cellular epigenetic status as displayed in Figure 1 ^10^^,^^15^. This epigenetic dysregulation leads to impairment of haematopoietic differentiation^16^^,^^17^.

**Figure 2.**
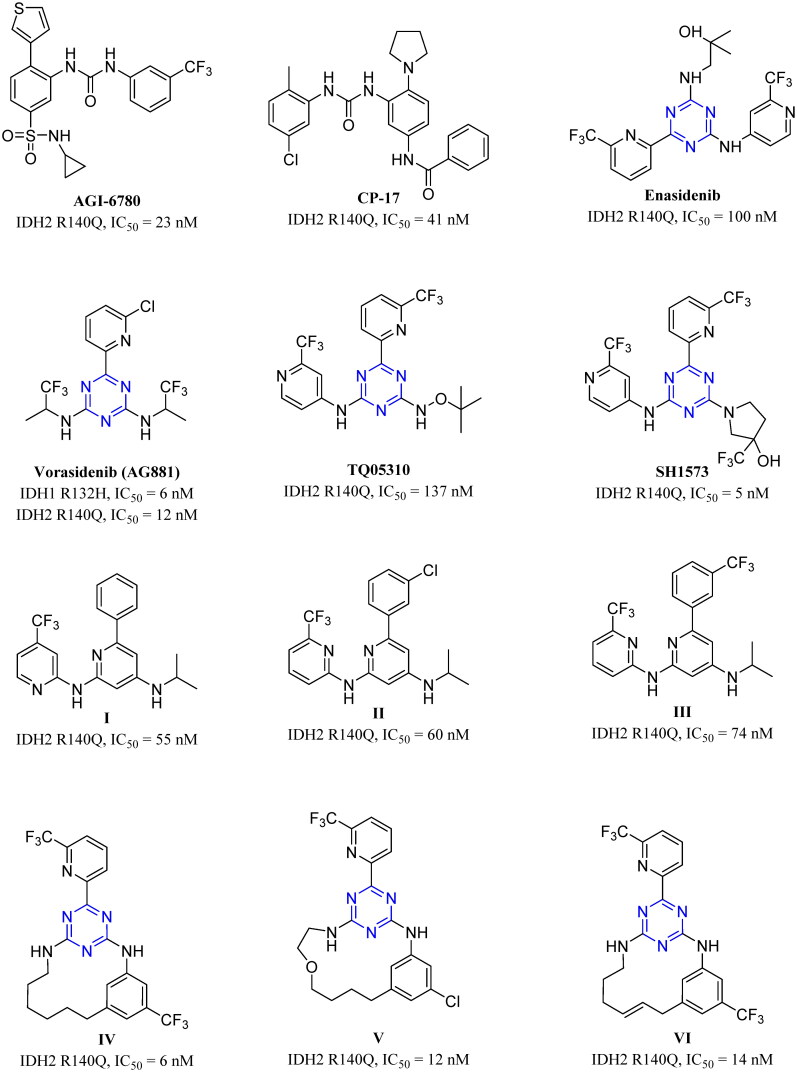
Chemical structures of representative mutant IDH2^R140Q^ inhibitors.

Synthesis of nitrogen-containing heterocyclic molecules has been receiving great interest owing to their utility for wide variety of biological receptors^18^. *s*-Triazine scaffold reported many biological activities, such as antibacterial, antiviral, antifungal, and especially anticancer^18–20^. Cyanuric chloride which is the starting core for preparation of several *s*-triazine derivatives has the advantages of low cost and ease of nucleophilic substitution of the three chlorine atoms^21^^,^^22^. Based on that, many biologically active compounds, including IDH2 inhibitors, have been evolved. Reported IDH2 mutant inhibitors are AGI-6780^23^, enasidenib^24^, CP-17^25^, vorasidenib (AG881)^26–28^, HMPL-306^29^, TQ05310^30^, and SH1573^31^. In addition, two classes of inhibitors are reported including pyridine^32^, and macrocyclic derivatives^33^ (Figure 2).

**Figure 3.**
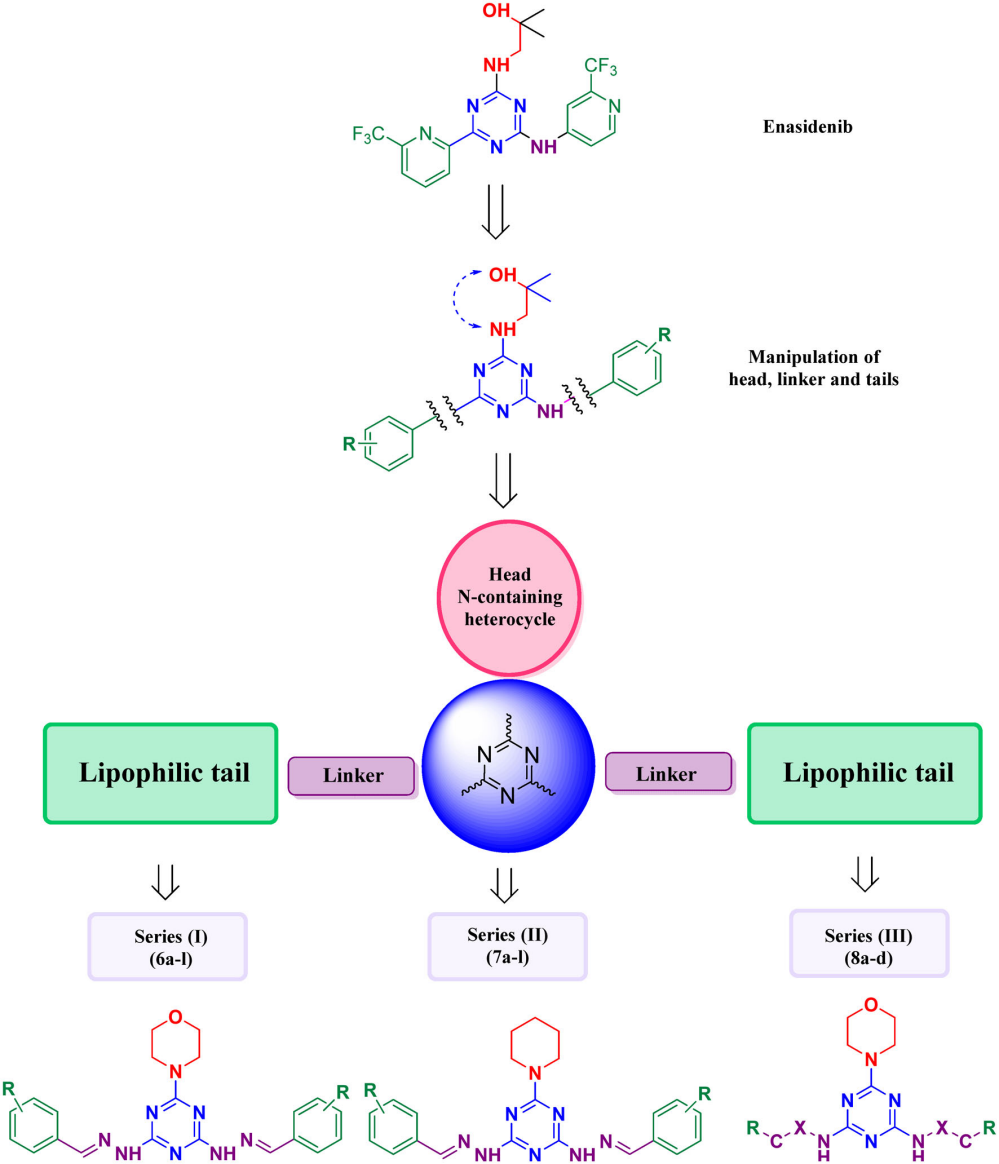
Rational design of novel target compound in series **(I), 6a–l**, series **(II), 7a–l**, and **(III), 8a–d**, taking enasidenib as a lead compound.

Enasidenib is a first in class and only FDA-approved inhibitor of mutant IDH2 for the treatment of adult patients with relapsed or refractory AML^34^. Enasidenib has lowered 2-HG levels in blood of AML patients, reduced blast counts and increased myeloid cells percentage^24^. Dual tail approach is one of the most effective approaches for compounds design which has been widely applied in some anticancer targets^35–37^. As disclosed cocrystal structure of enasidenib with IDH2^R140Q^ (PDB ID: 5I96), Enasidenib binding is anchored by multiple hydrogen bonds (H-bonds) formed with Q316 and many hydrophobic interactions^24^. It was noticed that binding site of enasidenib is a symmetric and hydrophobic pocket^24^. The symmetric hydrophobic pocket is located within homodimer interface. Herein, for assessment of the dual tail strategy in case of IDH2, we designed and synthesised 28 novel *s*-triazine inhibitors with symmetric hydrophobic tails using enasidenib as a lead compound. Enasidenib structure is composed of four integrated moieties: *s*-triazine as a central core, hydroxy alkyl amine as a head, and two lipophilic tails either with or without linkers^24^^,^^32^. Relying on these features, we reported three novel series. In series **(I),** 12 new target compounds, **6a–l**, were constructed with a central *s*-triazine ring bearing morpholine, as bioisostere of hydroxy alkyl amine, and two groups of substituted aromatic rings as the lipophilic symmetric tails connected to the *s*-triazine core with a methylene hydrazine linker. Regarding series **(II),** 12 novel compounds, **7a–l,** were designed as analogues to series **(I)** with piperidine as the heterocyclic head as bioisostere of morpholine. In series **(III),** 4 new target compounds**, 8a–d**, were assembled with the crucial *s*-triazine scaffold, morpholine head, and two symmetric lipophilic tails connected to *s*-triazine ring with diverse manipulated linkers as shown in Figure 3.

## Results and discussion

### Chemistry

**Scheme 1.**
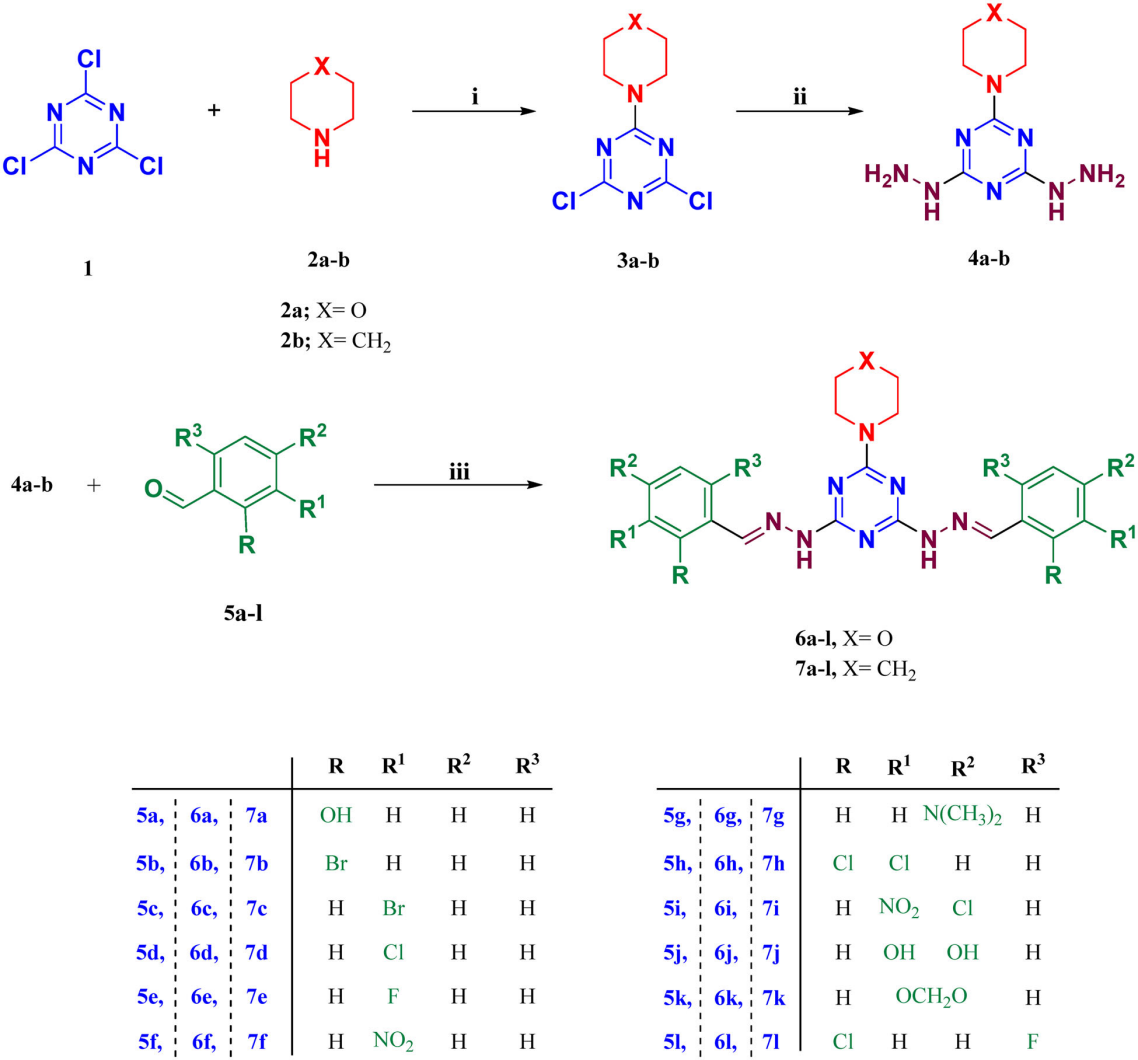
Synthesis of target compounds in series **(I), 6a–l** and series **(II), 7a–l.** Reagents and conditions: i: Acetone, Na_2_CO_3_, 0–5 °C, 4 h; ii: Acetonitrile, hydrazine hydrate 99%, heat under reflux; iii: Ethanol, *glacial* acetic acid, and heat under reflux.

Target compounds, of series **(I), 6a–l,** and series **(II)**, **7a–l** were prepared as displayed in Scheme 1. The three chlorine atoms of cyanuric chloride, **1** disclosed diverse reactivity and can be substituted gradually at different temperatures^38^. Compounds **3a,b** were prepared from cyanuric chloride **1** *via* nucleophilic substitution of the first chlorine atom with morpholine, **2a** or piperidine, **2b** at 0–5 °C to afford analogues, **3a** and **3b,** respectively. Compounds **3a,b** underwent nucleophilic substitution of the remaining two chlorine atoms with two hydrazine groups through heating under reflux with excess amount of hydrazine hydrate to provide the trisubstituted *s*-triazine derivatives, **4a,b**, respectively. Condensation of hydrazine derivatives, **4a,b** with various aldehydes yielded the corresponding hydrazones, **6a–l** and **7a–l**, respectively.

**Scheme 2.**
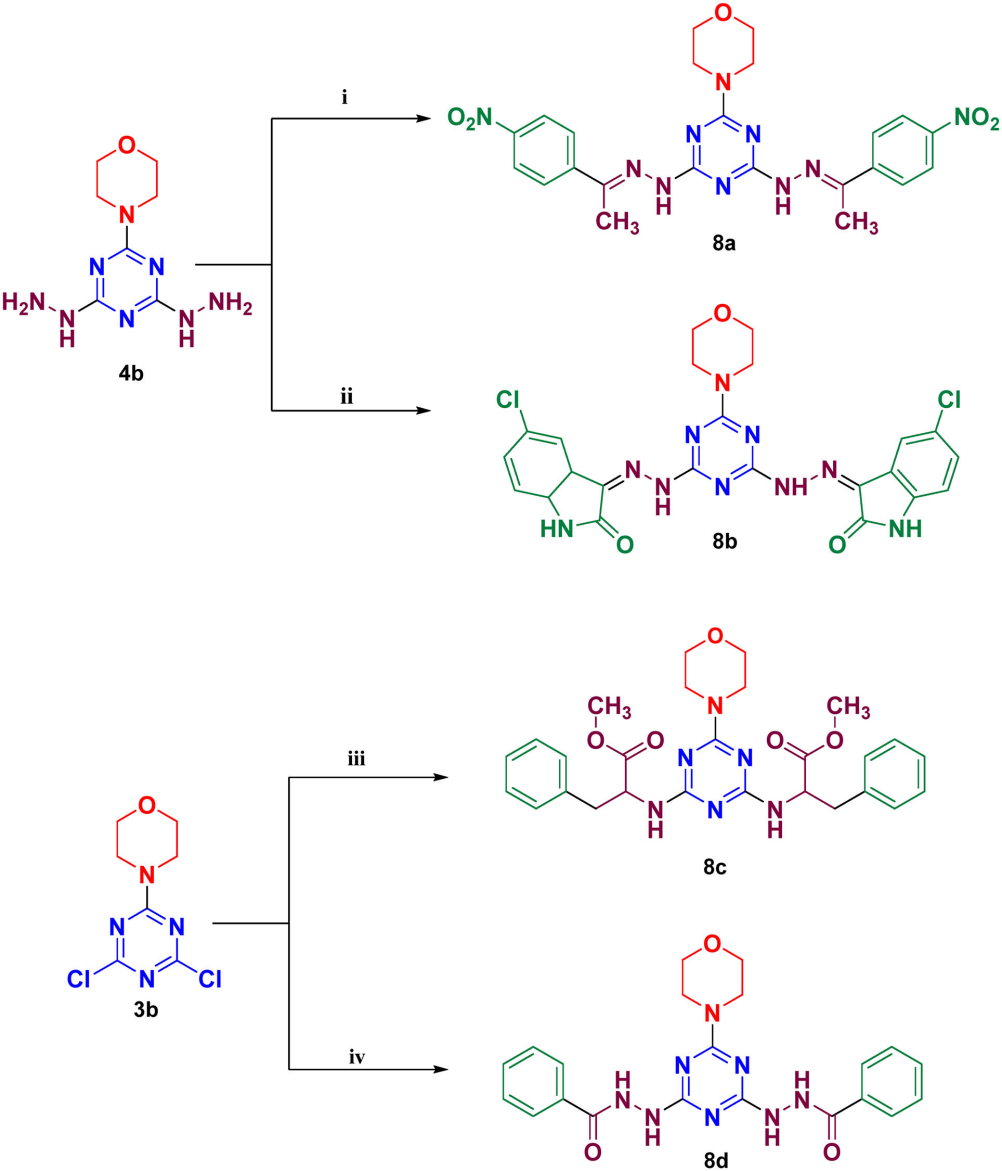
Synthesis of compounds in series **(III), 8a–d.** Reagents and conditions: i: 4-Nitroacetophenone, ethanol, *glacial* acetic acid, reflux 8 h. ii: 5-Chloroisatin, ethanol, *glacial* acetic acid, reflux 8 h. iii: Acetonitrile, L-phenylalanine methyl ester hydrochloride, Na_2_CO_3_ solution, reflux 96 h. iv: Benzohydrazide, acetonitrile, and reflux 6 h.

Target compounds in series (**III**), were synthesised as depicted in **Scheme 2**. Synthesis of **8a** and **8b** were accomplished through condensation of substituted hydrazine, **4b** with two different ketones; *p*-nitroacetophenone and 5-chloroisatin respectively. Nucleophilic aromatic substitution reaction between compound, **3b** and excess amount of either phenylalanine methyl ester or benzoyl hydrazine afforded analogues, **8c** and **8d**, respectively, bearing two variable linkers. 5-Chloroisatin and benzoyl hydrazine were synthesised as previously reported methods^39^. The chemical structures of synthesised compounds were confirmed by elemental analysis, ^1^H and ^13^C NMR, as well as mass spectrometry.

In this study, twenty-eight compounds were synthesised, whereas their chemical structures were confirmed *via* ^1^H NMR, ^13^C NMR, NOESY, elemental analysis as well as EI-MS (Supplementary file contains spectra). ^1^H NMR spectra of target compounds in series (**I**) and (**II**) displayed a characteristic singlet of the imine proton (–N = CH) in the range, 7.96–8.63 ppm with concurrent disappearance of hydrazine-NH_2_ signal of **4a,b** at 4.1 ppm which postulated the formation of imine (–N = CH) bond. Moreover, a characteristic downfield singlet signal at 10.53–13.34 ppm corresponding to the (–NHN = C–) proton was observed. Compounds, **6a, 6j, 7a**, and **7j** were characterised by manifestation of additional downfield signals of (–OH) group at 9.15–11.31 ppm, whereas analogues, **6g, 6k, 7g**, and **7k** displayed singlets of aliphatic (CH_3_/CH_2_) protons at 3.02, 6.12, 2.97, and 6.08 ppm, respectively. Concerning ^1^H NMR spectra of series (III), compound **8a** was characterised by upfield singlet at 2.38 ppm related to the aliphatic methyl protons (N = C–CH_3_). In addition, compound **8b,** revealed the (NH) proton of isatine moiety, as a singlet further downfield at 10.88 and 11.37 ppm. Besides, a doublet of NH proton in compound, **8c** was recognised at 8.49 ppm. Finally, compound, **8d** showed two singlets concerning the (**NH**-NHCO) and (NH-**NH**CO) protons assigned at 8.89 and 10.26 ppm, respectively.

**Figure 4.**
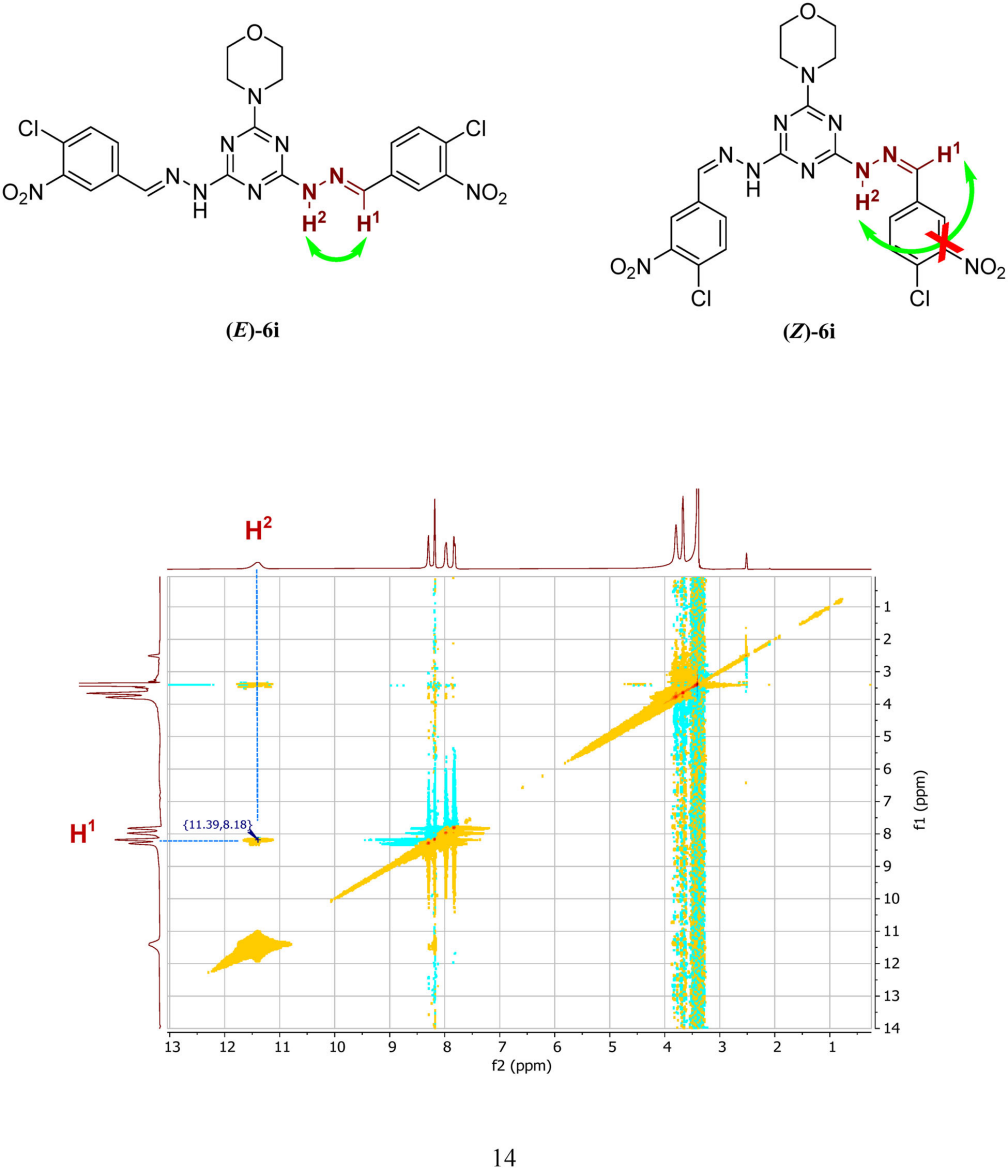
^1^H-^1^H Homonuclear 2 D NOESY spectrum for *E-*isomer of compound **6i**.

The geometry of synthesised hydrazones (**6a–l** and **7a–l)**, was confirmed as *E*-isomer rather than *Z*-isomer relying on interpretation of the 2D NOESY spectra achieved for compound **6i**. ^1^H–^1^H homonuclear NOESY spectrum discovered a NOE signal which is assigned between two protons; H^1^ (CH = N, *δ* = 8.18 ppm) and H^2^ (NH-N = C, *δ* = 11.39 ppm), which is in agreement with the *E*-isomer (*E*-**6i**) configuration and is not tolerable in the putative (*Z*-**6i**) attributable to the larger intramolecular H-H distance of *Z*-isomer as demonstrated in Figure 4. Therefore, target compounds in series (**I**) and (**II**) are established as the *E*-isomers^40^^,^^41^.

### Biological evaluation

#### In vitro anticancer screening at National Cancer Institute (NCI-USA)

Target compounds of series **(I); 6a–l, (II); 7a–l** and **(III); 8a–d,** were submitted to National Cancer Institute “NCI” (www.dtp.nci.nih.gov). Our target compounds were accepted for assessment of anticancer activity in a single-dose test. Accordingly, five analogues (**6a**, **6c**, **6d**, **7g**, and **7l**) were further subjected to five-dose experiment.

#### In vitro preliminary cytotoxic activity at a single dose of 10 μM against full NCI 60 cancer cells panel

**Figure 5.**
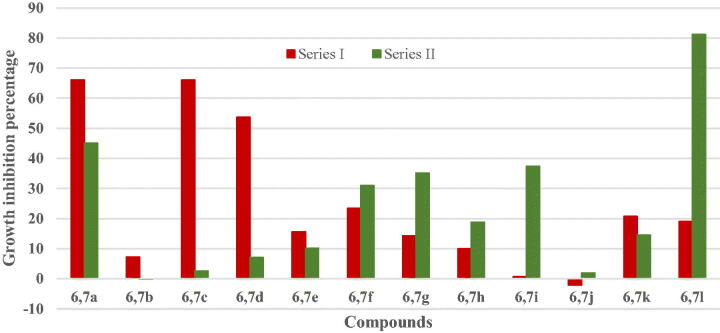
Mean growth inhibition percentage (GI %) on 60 cancer cell lines for series **(I)** and series **(II)** in the single dose experiment.

Initially, the anticancer effects of target compounds have been assessed at a single (10 μM) dose. The obtained data have been reported as mean-graph of the percent growth (G%) of the treated cancer cells. Percentage growth inhibition (GI%) is calculated (100 − G%) and pronounced in Table 1. Inspection of *in vitro* antitumor screening data revealed that our *s*-triazine derivatives exposed variable anticancer activity ranging from low, moderate, to high potency. Preliminary examination of NCI data showed that compounds in series **(I)** with morpholine head and their corresponding analogues in series **(II)** with piperidine moiety demonstrated an overall comparable activity as reported in Figure 5. Target compounds in series **(III)** with manipulated linkers disclosed lower potency. Superiorly, eight *s*-triazine derivatives from the three series (**6a**, **6c**, **6d**, **7a**, **7g**, **7i**, **7l**, and **8b**) have shown potent broad-spectrum anticancer activity against most the examined cell lines, whereas most of the remaining triazine derivatives, 20 derivatives, have exerted selective anti-proliferative actions towards certain cancer cell lines (Supplementary). The GI% exerted by the examined *s*-triazine derivatives **6a**, **6c**, **6d**, **7a**, **7g**, **7i**, **7l,** and **8b** have been listed in Table 1.

**Table 1.** Preliminary anticancer effects of single dose (10 *μ*M) of *s*-triazine derivatives **6a**, **6c**, **6d**, **7a**, **7 g**, **7i**, **7l**, and **8b** against 60 human subpanel cancer cell lines declared as the percentage cell growth inhibition (GI%).

Subpanel cell lines	Growth inhibition percentage (GI %)							
	6a	6c	6d	7a	7g	7i	7l	8b
Leukaemia								
CCRF-CEM	57.65	**79.05**	**75.18**	**78.34**	**74.20**	38.67	**77.73**	**79.75**
HL-60(TB)	**96.26**	**110.20**	**109.74**	52.57	**137.97**	58.1	**98.73**	27.78
K-562	**74.64**	**85.49**	**88.23**	**61.84**	**96.92**	**68.28**	**67.73**	**67.14**
MOLT-4	54.86	**89.80**	**93.96**	59.08	**87.95**	57.66	**86.29**	37.48
RPMI-8226	46.36	**65.83**	**61.19**	57.37	**67.36**	**81.72**	**96.73**	**63.60**
SR	**84.03**	**92.07**	**92.30**	**60.18**	**114.07**	36.83	**83.92**	**70.79**
Non-small cell lung cancer								
A549/ATCC	**87.04**	57.40	53.38	52.18	21.55	−11.20	**78.36**	−1.37
EKVX	**77.82**	50.98	27.82	**69.87**	2.99	22.00	**86.19**	14.84
HOP-62	**74.28**	47.72	−9.83	44.19	18.25	56.32	**74.13**	**62.44**
HOP-92	**87.15**	36.22	34.55	21.61	16.02	−23.07	**101.32**	**73.99**
NCI-H226	**76.72**	56.72	39.65	18.20	−1.93	38.57	46.14	42.69
NCI-H23	58.46	56.93	29.37	44.22	3.27	18.2	55.17	**82.84**
NCI-H322M	55.47	47.23	46.16	41.08	−0.12	28.75	**64.24**	21.94
NCI-H460	**67.51**	58.82	21.79	**60.50**	29.08	1.33	**80.13**	11.84
NCI-H522	39.06	**66.45**	86.08	52.53	3.84	48.08	**89.64**	**94.91**
Colon cancer								
COLO 205	52.04	**76.23**	**63.11**	**75.48**	16.53	−0.27	**116.72**	51.30
HCC-2998	41.13	**68.49**	41.04	**60.43**	−3.10	32.58	**75.65**	24.46
HCT-116	**80.81**	**78.76**	**77.2**	22.01	**85.13**	**78.03**	**80.49**	54.64
HCT-15	**96.13**	**108.94**	**111.07**	22.55	**99.88**	50.33	**96.06**	8.40
HT29	**65.76**	**93.39**	**91.79**	54.71	**104.67**	**92.57**	**96.50**	8.90
KM 12	47.32	**69.70**	**69.79**	52.48	6.98	**94.55**	**89.24**	18.80
SW-620	**61.5**	**75.30**	**81.91**	32.18	35.82	36.29	**82.81**	25.67
CNS cancer								
SF-268	27.74	31.58	34.13	45.67	−5.33	35.13	**63.34**	50.89
SF-295	**80.42**	**70.86**	42.35	47.41	−6.74	6.78	**97.43**	54.35
SF-539	48.19	43.61	56.40	40.62	**112.27**	**63.69**	43.75	**83.97**
SNB-19	58.32	22.05	12.05	44.05	13.95	27.44	**74.23**	**109.43**
SNB-75	**104.09**	−7.50	−2.64	25.60	−1.39	−36.06	**62.51**	**100.67**
U251	**78.04**	57.30	39.61	49.59	49.97	**81.50**	**73.21**	**96.19**
Melanoma								
LOX IMVI	**95.96**	**94.77**	**67.40**	**60.31**	**173.29**	34.01	**80.37**	**76.78**
MALME-3M	47.37	**153.96**	**143.74**	33.08	**167.33**	−1.47	**95.81**	43.58
M14	**62.38**	**68.29**	**64.84**	53.57	27.17	7.70	**88.10**	22.51
MDA-MB-435	41.07	**66.57**	50.74	30.87	39.61	19.28	**96.25**	36.49
SK-MEL-2	27.99	**97.36**	**101.89**	30.78	−10.79	37.8	**125.78**	34.71
SK-MEL-28	**71.96**	**82.88**	**70.18**	36.56	58.34	22.22	55.11	44.95
SK-MEL-5	34.20	**79.26**	55.87	25.60	13.27	48.87	40.95	53.94
UACC-257	46.66	**64.12**	**60.86**	20.85	−4.45	56.87	**88.28**	18.02
UACC-62	11.18	43.96	8.33	29.72	−0.61	34.01	**81.67**	48.43
Ovarian cancer								
IGROV1	**63.00**	57.18	49.35	54.81	6.23	47.25	**82.32**	45.01
OVCAR-3	**66.39**	57.55	43.71	**80.77**	34.42	**107.45**	**77.35**	47.29
OVCAR-4	**88.25**	45.82	33.6	**70.94**	−6.05	13.14	**78.95**	21.34
OVCAR-5	**63.03**	27.60	4.53	28.44	3.87	−8.09	**72.15**	4.93
OVCAR-8	**87.26**	**62.58**	50.51	50.41	43.4	**68.96**	**74.19**	49.91
NCI/ADR-RES	**77.06**	58.24	38.19	**80.97**	41.7	**69.76**	**84.77**	1.85
SK-OV-3	**88.13**	7.13	−29.62	12.04	−31.8	1.24	**60.86**	44.55
Renal cancer								
786-0	**79.66**	**75.48**	**69.44**	26.78	11.81	−8.84	**71.74**	26.29
A498	NT	NT	**95.69**	17.02	−15.11	−8.06	47.2	**78.37**
ACHN	**71.90**	**73.69**	**74.23**	**63.13**	**67.57**	**64.84**	**95.79**	25.48
CAKI-1	**95.44**	**84.64**	62.40	24.47	10.06	27.58	**85.87**	27.98
RXF 393	**100.65**	**168.01**	**82.55**	16.96	6.71	0.13	**96.62**	**86.34**
SN 12C	**72.81**	55.80	40.07	40.48	49.57	27.66	**76.81**	56.08
TK-10	52.05	33.24	−0.21	2.29	−18.38	6.20	**72.77**	−18.46
UO-31	**80.18**	**74.5**	**73.15**	51.67	19.69	52.54	**90.98**	21.35
Prostate cancer								
PC-3	**75.90**	**63.47**	58.52	55.25	15.19	46.60	**81.33**	43.85
DU-145	**64.49**	**72.68**	57.48	45.22	22.48	**70.38**	39.03	11.63
Breast cancer								
MCF7	**59.97**	**77.94**	57.05	**64.28**	**68.89**	**99.09**	**96.90**	56.91
MDA-MB-231/ATCC	**63.72**	33.87	28.72	34.54	31.42	51.67	57.89	**63.69**
HS 578T	53.04	33.8	32.54	23.41	−6.05	46.16	**66.74**	**107.82**
BT-549	23.67	47.32	7.26	NT	−10.63	2.15	31.62	**106.36**
T-47D	53.94	43.55	31.48	51.76	53.97	51.82	**89.05**	**70.65**
MDA-MB-468	**60.84**	**98.91**	**69.24**	36.20	**63.73**	31.23	**114.15**	**76.37**
Mean GI %	66.07	66.00	53.69	45.09	35.10	37.38	81.15	47.89

NT: not tested.

GI% less than 10 indicates low activity; GI% 10:60 indicates moderate activity; GI% 60:100 indicates strong activity (coloured with red); GI % more than 100 indicates very strong activity (lethal), (coloured with red); GI % greater than 60 were bolded.

**Figure 6.**
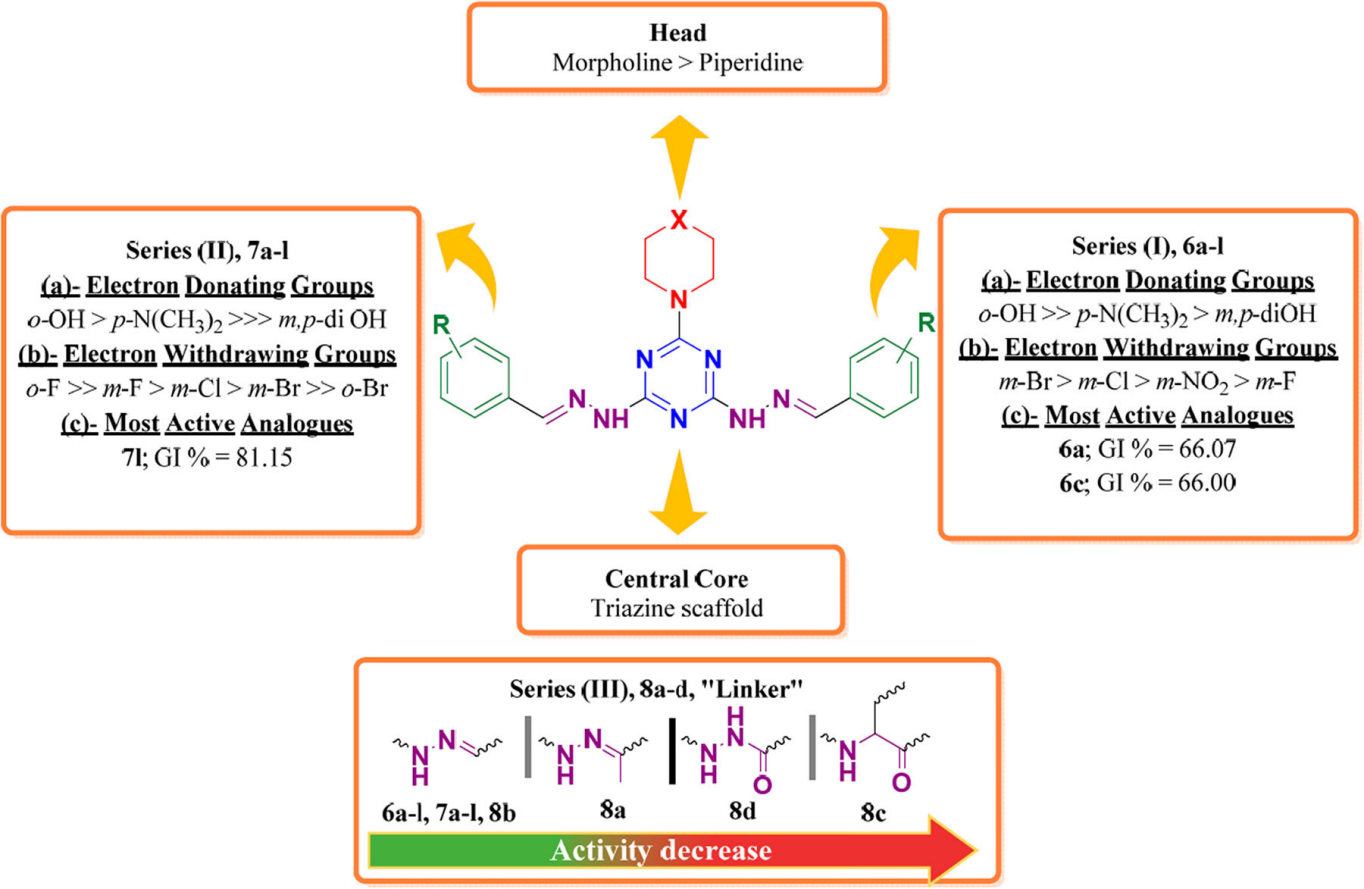
Summary of structure–activity relationship (SAR) of series **(I)**, **(II)**, and **(III)** as anticancer agents against 60 human subpanel cancer cell lines relying on the values of mean GI%.

Each series pronounced its own structure–activity relationships (SARs) as following:

- Anticancer activity of series **(I)** was largely affected by substituents on the symmetric lipophilic tails. SAR was deduced as following depending on GI % values demonstrated in Table 1:
  - Electron donating groups (EDG) at *ortho* position enhanced activity as noticed in compound **6a** (*o*-OH group, GI %= 66.07) which demonstrated one of the two most active analogues in series **(1)**. Placement of EDG to the *meta* or the *para* positions diminished activity as reported for analogues **6j** (*m,p*-di OH, GI %= -2.12), and **6g** (*p*-N(CH_3_)_2_, GI %= 14.28).
  - Electron withdrawing groups (EWG) such as halogens at *meta* position improved potency as reported in compounds **6c (***m*-Br, GI% = 66) and **6d** (m-Cl, GI%= 53.69). Size and electronegativity of halogens at *meta* position increased potency in the order: *m*-Br > *m*-Cl ≫> *m*-F. The presence of halogens at *ortho* position reduced the activities of analogues **6b** (*o*-Br, GI% = 7.16), **6h** (*o,m*-di Cl, GI% = 10), and **6l** (*o*-Cl, *o’*-F, GI% = 19.00). Moreover, Nitro group at *meta* position in diminished activity of analogues **6f** (*m*-NO_2_, GI% = 23.39), and **6i** (*m*-NO_2_, *p*-Cl, GI% = 0.67) compared to halogenated analogues.
- Concerning series **(II),** the most active analogue **7l** reported the strongest anticancer activity (GI% = 81). Substituents at phenyl groups of the lipophilic tails demonstrated variable impact relying on their nature and position as following:
  - EDG like hydroxyl group at *ortho* position of analogue **7a,** revealed moderate anticancer effect (GI% = 45.09). The presence of *m,p*-di (OH) groups in compound **7j,** abolished its activity (GI% = 1.89). Introduction of *p*-N(CH_3_)_2_ to analogue **7g** enhanced its potency (GI% = 35.10).
  - EWG, such as halogens in **7l** (GI% = 81.15), enhanced its potency because of fluoride and chloride, atoms at both *ortho* positions of its phenyl tails. The presence of *o*-Br in **7b** (GI% = −0.12), *m*-Br in **7c** (GI% = 2.56), and *m*-Cl in **7d** (GI% = 7.03) abolished anticancer activity. Introduction of *m*-F in **7e** (GI% = 10.18) revealed moderate activity. Accordingly, type and position of different halogens affect anticancer activity in the following orders: *o*-F ≫ *m*-F > *m*-Cl > *m*-Br ≫ *o*-Br. Moreover, nitro group at *meta* position enhanced potency of **7f** (GI% = 30.94) and **7i** (GI% = 37.38).
- In case of series **(III),** the corresponding hydrazones of acetophenone, **8a** (GI %= 23.27), and isatin, **8b** (GI% = 47.89) displayed moderate anticancer activity. Isatin tails enhanced the potency compared to acetophenone. Furthermore, introduction of amino acid ester as lipophilic tails in **8c** or benzohydrazide in **8d** abolished activity as recorded in Table 2. SAR of target compounds of series **(I), (II),** and **(III)** is summarised in Figure 6.

**Table 2.** Cytotoxic effects of five doses (0.01–100 μM) for compounds, **6a**, **6c**, **6d**, **7g**, **7l**, and enasidenib towards 60 human subpanel cancer cell lines declared as GI_50_ (μM).

Subpanel cell lines	GI_50_ (μM)												
	6a	6c	6d	7g	7l	En.^a^		6a	6c	6d	7g	7l	En.
Leukaemia							Melanoma						
CCRF-CEM	**0.53**	**0.57**	1.34	1.21	NT^b^	19.95	SK-MEL-2	6.06	2.43	2.18	1.99	1.69	NT
HL-60(TB)	**0.19**	**0.15**	**0.50**	2.20	2.00	19.95	SK-MEL-28	3.20	NT	2.86	2.00	3.41	15.84
K-562	**0.68**	**0.33**	**0.69**	**0.89**	3.08	31.62	SK-MEL-5	5.86	1.99	1.72	1.73	1.88	12.58
MOLT-4	1.55	**0.68**	1.49	2.87	NT	12.58	UACC-257	6.53	NT	2.47	1.83	2.39	19.95
RPMI-8226	2.78	1.68	1.90	1.92	2.26	19.95	UACC-62	2.45	NT	4.54	1.84	2.35	15.84
SR	**0.32**	**0.16**	**0.32**	**0.36**	NT	19.95	Ovarian cancer						
Non-small cell lung cancer							IGROV1	**0.97**	2.92	4.61	2.96	3.16	31.62
EKVX	3.28	NT^b^	4.23	3.13	1.54	19.95	NCI/ADR-RES	**0.69**	3.25	4.58	3.19	2.76	15.84
HOP-92	1.65	1.24	2.17	1.47	1.47	25.11	Renal cancer						
Colon cancer							786-0	**0.71**	**0.53**	1.07	1.69	3.44	19.95
COLO 205	5.53	1.24	1.23	1.89	1.78	19.95	A498	1.09	**0.14**	1.67	1.58	2.40	15.84
HCT-116	**0.82**	**0.78**	1.27	1.64	2.83	15.84	ACHN	1.58	2.21	2.11	2.68	3.04	25.11
HCT-15	**0.24**	**0.42**	**0.91**	1.87	2.82	19.95	RXF 393	**0.27**	**0.16**	1.00	2.12	2.65	19.95
HT29	5.89	**0.61**	1.34	1.76	2.90	15.84	SN 12C	18.3	NT	4.99	1.87	2.85	19.95
SW-620	1.78	**0.83**	1.78	1.89	3.51	25.11	UO-31	**0.37**	1.01	1.61	1.72	2.01	19.95
CNS cancer							Prostate cancer						
SF-295	7.67	4.94	4.13	1.96	1.01	15.84	DU-145	6.09	1.31	2.62	3.92	11.7	19.95
SF-539	7.29	3.83	5.39	1.99	7.65	15.84	Breast cancer						
SNB-75	1.82	4.54	3.33	2.80	2.36	15.84	MDA-MB-231/ ATCC	2.42	3.97	9.16	1.80	7.65	19.95
Melanoma													
LOX IMVI	**0.28**	2.66	2.99	1.61	3.77	15.84	BT-549	5.19	3.54	3.22	1.55	4.45	15.84
MALME-3M	8.69	1.88	2.86	1.86	2.21	19.95	T-47D	3.34	3.94	3.54	2.18	1.71	15.84
MDA-MB-435	9.53	NT	4.24	1.94	2.86	15.84	MDA-MB-468	4.11	1.91	1.65	1.64	1.86	19.95

^a^En.: Enasidenib; ^b^NT: not tested.

Red colour indicates GI_50_ = 1–2 μM, Blue colour indicates GI% less than 1 μM. Bold values indicate GI_50_ less than 1μM.

#### In vitro cytotoxic activity at five doses (0.01–100 μM) for analogues 6a, 6c, 6d, 7g, and 7l against full NCI 60 cancer cell panel

Aforesaid outcomes of single-dose anticancer screening for target compounds (Table 1) figured out that analogues, **6a**, **6c**, **6d**, **7g**, and **7l** are susceptible for further screening against 60 cell lines of the nine different cancer types, at five doses (0.01–100 μM) to determine GI_50_, TGI, and LC_50_ as informed in Table 2. It was observed that leukaemia subpanel cell lines were the most sensitive cancer cells where tested compounds reported the best GI_50_ values in nanomolar range. The most active analogue, **6c** reported, GI_50_ = 0.15 and 0.16 μM against HL-60(TB) and SR, respectively.

Moreover, compound **6c** displayed high potency towards colon cancer cell lines, HCT-116, HCT-15, HT29, and SW-620 reporting, GI_50_ **=** 0.78, 0.42, 0.60, and 0.82 μM, respectively. Renal cancer cell lines A498 and RXF 393 were significantly, sensitive to compound **6c** and reported GI**_50_** = 0.14 and 0.16 μM, respectively. Compounds **6a**, **6d**, **7g**, and **7l** disclosed high potency against various types of cancer cells as revealed in Table 2. Compound **6a** reported the best cytotoxic activity, GI**_50_** = 0.24, 0.28, and 0.69 μM, against colon cancer cells (HCT-15), melanoma (LOX IMVI), and ovarian cancer cells (NCI/ADR-RES), respectively.

#### In vitro isocitrate dehydrogenase 2 enzyme inhibition assay

The most active cytotoxic analogues, **6a**, **6c**, **6d**, **7g**, and **7l** as well as other selected compounds, **6e, 6g, 6l, 7a, 7c, 7d**, and **7e** were evaluated for their abilities to inhibit the IDH2^R140Q^ mutant *via* an enzyme-based assay. Enasidenib was used as a positive control and the results were expressed as IC_50_ values as presented in Table 3. Compounds **6c**, **6e**, and **7c** established the best enzyme inhibition activity with IC_50_ = 101.70, 67.01, and 88.93 nM, respectively, compared to enasidenib which reported IC_50_ = 75.5 nM. Accordingly, analogue **6e** was the strongest IDH2^R140Q^ inhibitor and was more active than the standard drug, enasidenib. To evaluate the selectivity profile of our inhibitors, compounds **6c**, **6e**, **6l**, **7a**, **7c**, **7e**, and **7l** were selected for enzyme inhibition assay against wild type IDH2. The results confirmed that these inhibitors selectively inhibit IDH2^R140Q^ by 21–59–fold more than the wild type which gives an early indication on the safety of these inhibitors towards normal cells.

**Table 3.** *In vitro* inhibition of mutant and wild type IDH2 enzymes by selected target compounds.

Compound	IC_50_ nM ± SD^a^		Compound	^a^IC_50_ nM ± SD	
	IDH2^R140Q^	IDH2^wt^		IDH2^R140Q^	IDH2^wt^
**6a**	372.90 ± 18.20	NT^b^	**7a**	130.10 ± 6.35	5418 ± 210
**6c**	101.70 ± 4.97	2928 ± 110	**7c**	88.93 ± 4.34	3128 ± 120
**6d**	1868 ± 91.20	NT	**7d**	369.6 ± 18.00	NT
**6e**	67.01 ± 3.27	2295 ± 90	**7e**	176.00 ± 8.60	5071 ± 200
**6g**	336.20 ± 16.40	NT	**7g**	505.40 ± 24.70	NT
**6l**	158.00 ± 7.72	3438 ± 130	**7l**	159.80 ± 7.81	9496 ± 370
**Enasidenib**	75.5 ± 3.69	2330 ± 90			

^a^The mean ± SD of three experiments; ^b^NT: not tested.

#### In vitro cytotoxicity against human normal cells (human embryonic kidney)

Safety profile of compound **6c**, as a cytotoxic agent, was investigated towards normal cells, human normal embryonic kidney cells (HEK-293), in comparison to a reference drug, staurosporine^42^^,^^43^. As displayed in Table 4, compound **6c** exhibited better safety by reporting lower cytotoxicity against the normal cell (HEK-293), IC_50_ = 53.69 μM compared to staurosporine, IC_50_ = 35.33 μM.

**Table 4.** Cytotoxic activity of compound **6c** and staurosporine against the normal kidney cells of human embryo (HEK-293).

Compound	Cytotoxicity, IC_50_ (μM)^a^
**6c**	53.69 ± 2.94
Staurosporine	35.33 ± 1.93

^a^The mean ± SD of three experiments.

#### In vitro cell cycle analysis

Targeting cancer cell cycle has been emerged as a viable approach for cancer treatment^44^. To recognise the role of compound **6c** in GI of cancer cells, and induction of apoptosis in different phases, DNA flow cytometric analysis was performed to measure the effect of compound **6c** on cell cycle progression for leukaemia, HL-60(TB) cancer cells. HL-60(TB) cancer cells were treated with compound **6c** at GI_50_ concentration (158 nM) and DMSO, as a negative control, for 24 h, stained with propidium iodide (PI) and then analysed by flow cytometer. The results are reported in Table 5.

**Table 5.** Cell cycle analysis of HL-60(TB) cells treated with compound **6c** and DMSO as a negative control.

Compound	Cell cycle distribution (%)			
	%G0–G1	%S	%G2/M	%Pre-G1
**6c**	54.06	41.18	4.76	28.02
DMSO	46.29	33.92	19.79	1.95

**Figure 7.**
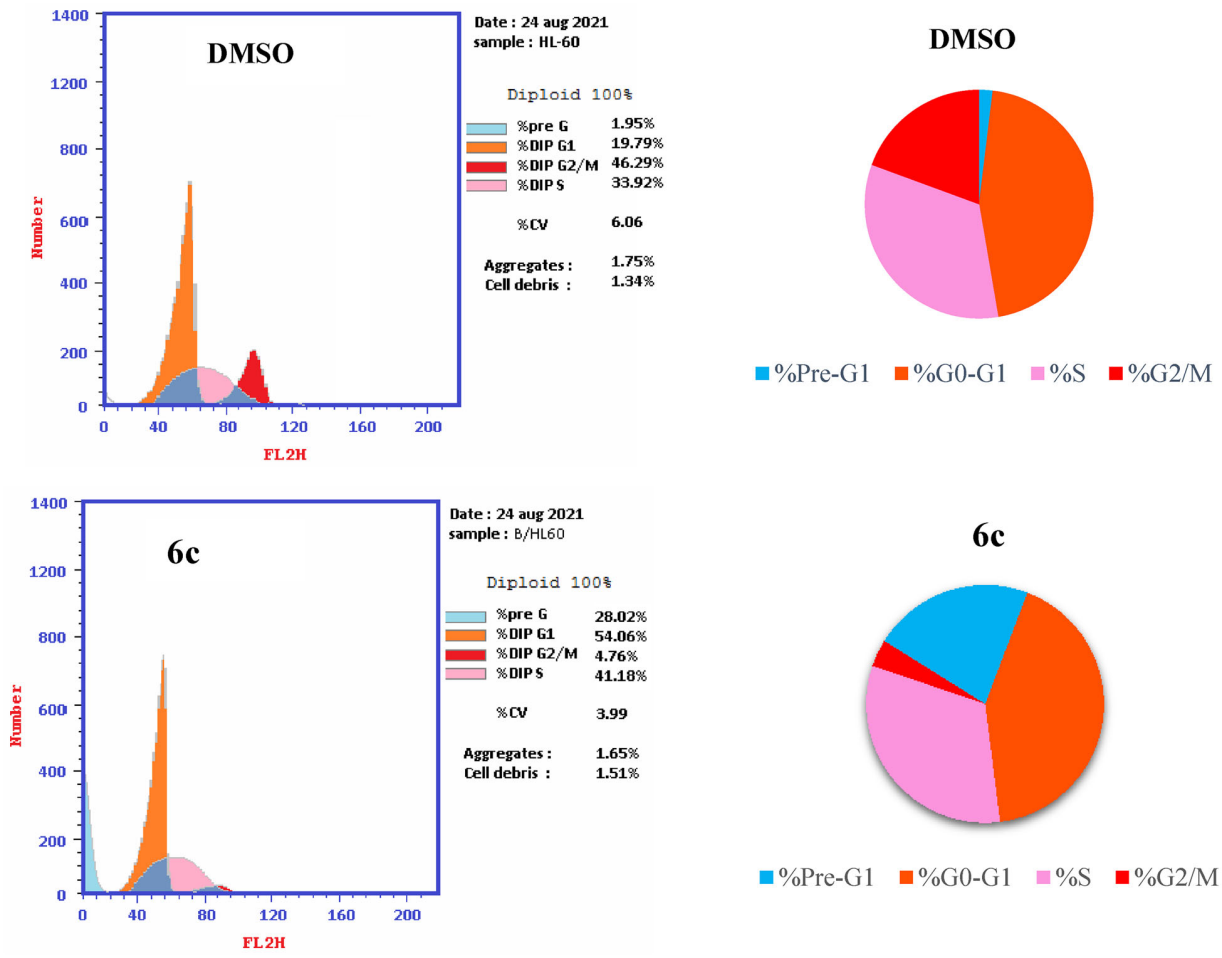
Effect of DMSO (upper two panels) and compound **6c** (lower two panels) on the cell cycle distribution of HL-60(TB) cancer cell line.

A significant increase in the percentage of apoptotic cells at the pre-G1 phase (28.02%) upon exposure to compound **6c** by 14-fold compared to DMSO (1.95%), was identified with concurrent decrease in the G2/M phase (4.76%) for **6c** relative to DMSO (19.79%). Furthermore, an increase in the cells at S and G0–G1 phases (41.18 and 54.06%, respectively) was detected compared to control (33.92 and 46.29%, respectively) as shown in Figure 7. Arresting cell growth in G1/S phase and alteration of the pre-G1 phase are considered significant remarks for compound **6c** to induce apoptosis in HL-60(TB) cancer cells.

### Detection of apoptosis

**Figure 8.**
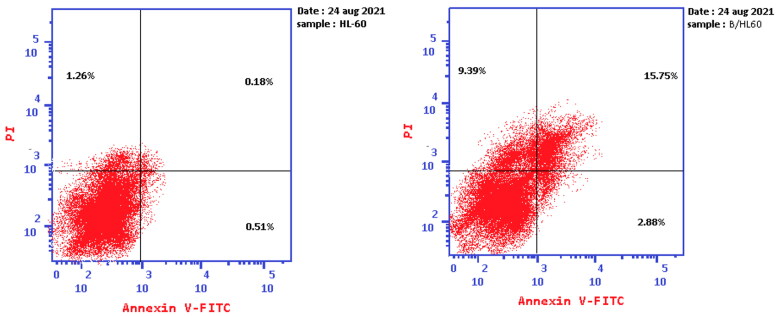
Apoptosis assay on HL-60(TB) cancer cell line induced by DMSO (left panel) and compound **6c** (right panel) the four quadrants identified as: LL: viable; LR: early apoptotic; UR: late apoptotic; UL: necrotic.

**Figure 9.**
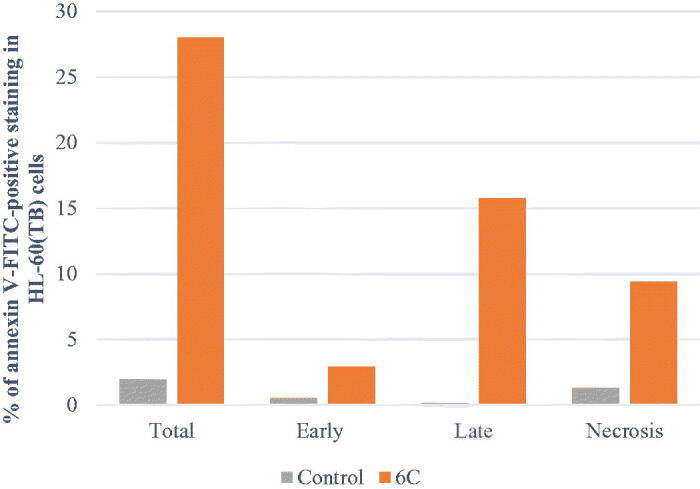
Summary of the Annexin V-FITC Apoptosis assay results of compound **6c** and DMSO on the percentage of HL-60(TB) cells stained positive for Annexin V-FITC.

Extrinsic as well as intrinsic apoptosis in leukaemia, HL-60(TB) cancer cells, induced by compound **6c** was evaluated by Annexin V and PI staining. Herein, HL-60(TB) cells were incubated with compound **6c** at GI_50_ (158 nM) concentration for 24 h. Compound **6c** induced an early apoptosis (2.88%), in HL-60(TB) at 24 h compared to control (0.51%) and enhanced late apoptotic induction (15.75%) by more than 85-fold over the untreated cells (0.18%). Moreover, compound **6c** induced necrosis by 7 times more than control as declared in Figures 8 and 9. This determination was consistent with the data obtained from cell cycle analysis revealed in Figure **7**.

### Western blot analysis for apoptotic markers, Caspase 3 and Caspase 9

**Figure 10.**
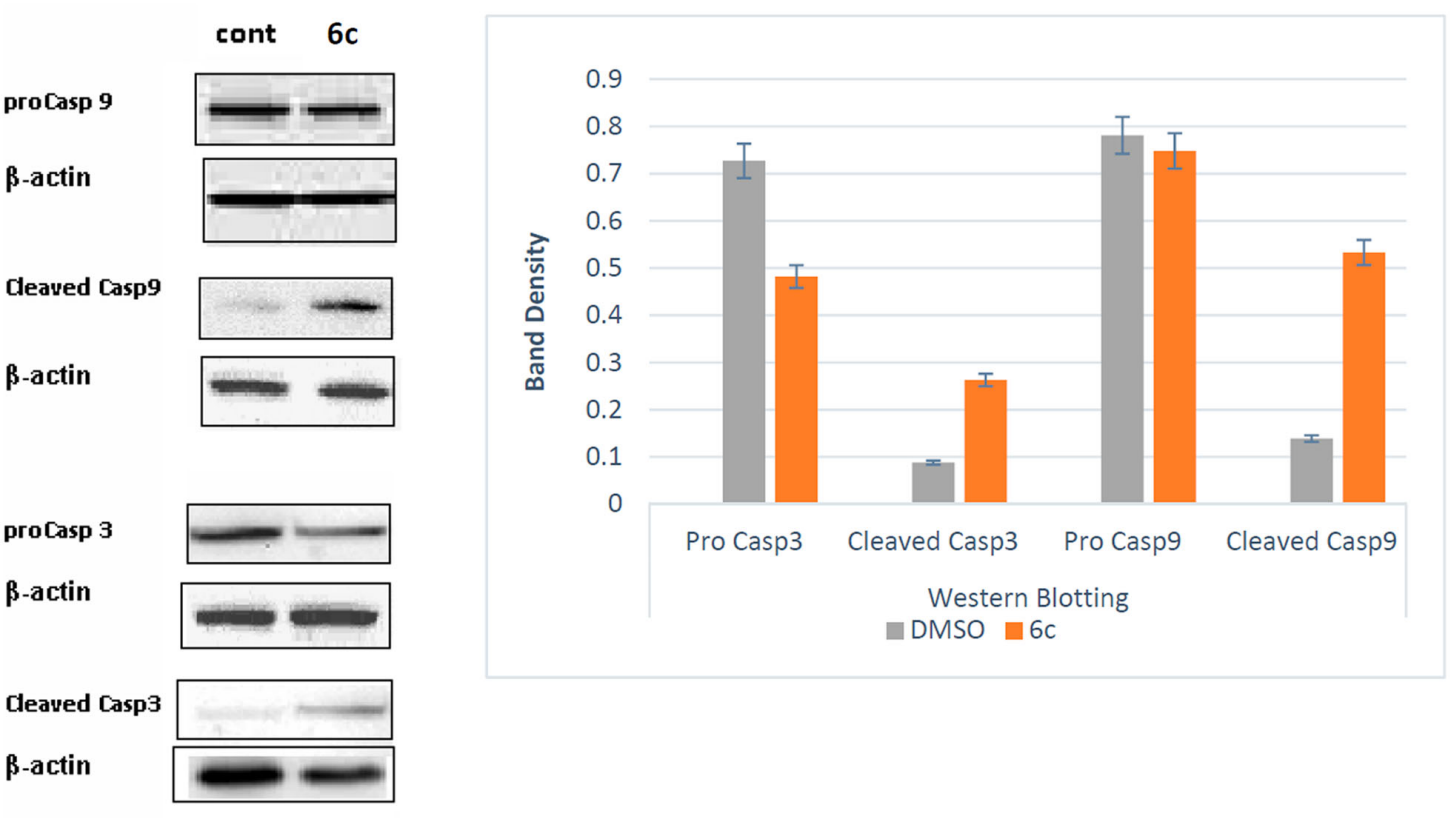
Impact of compound **6c** on expression of active Caspase 3 and Caspase 9 levels in HL-60(TB) cancer cells.

Cysteine-containing aspartic acid-specific proteases, caspases provide essential links in cell controlling the apoptotic machinery^45^^,^^46^. For this reason, this study was further extended to investigate the mechanism of compound **6c** to provoke apoptosis in HL-60(TB) leukaemia cell line. Treatment of HL-60(TB) cancer cells with **6c** significantly induced the expression of active caspases 3 and 9 by about 3 and 4-fold, respectively, in comparison to the control (Figure 10).

### In silico studies

#### Molecular docking study

**Figure 11.**
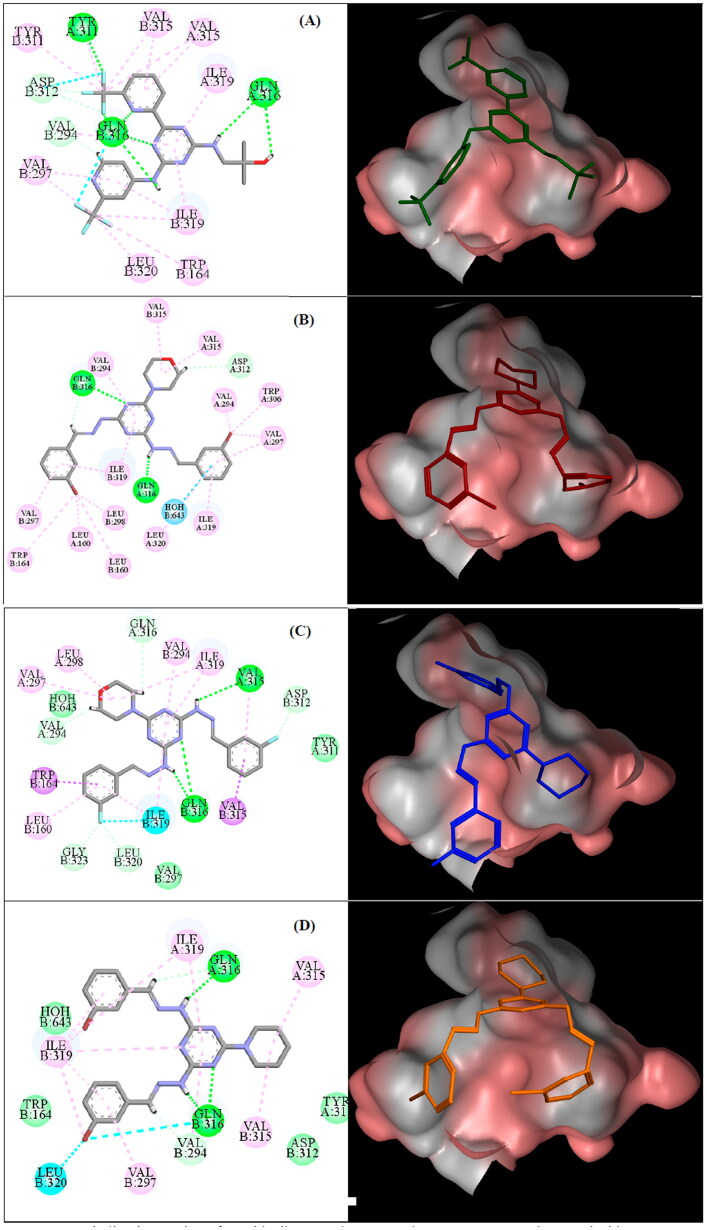
Binding interaction of enasidenib (A) and compounds **6c** (B), **6e** (C), and **7c** (D) inside IDH2^R140Q^ allosteric site (PDB ID: 5I96). 2D pose binding of the compound (left), green lines (H-bond), pink lines (hydrophobic interactions), cyan lines (halogen bond), and 3D surface representation of the compound in the allosteric site (right).

Docking study was accomplished, by molecular operating environment (MOE 2020.09, Montreal, QC, Canada), to examine the probable types of binding modes for the most active analogues, **6c**, **6e**, **7c**, and enasidenib on the allosteric site of the crystal structure of IDH2^R140Q^ (PDB: 5I96). Visualisation of compounds interactions was achieved *via* Discovery Studio (version 20.1.019295, San Diego) and MOE, where a high resolution (1.55 Å) X-ray crystal structure of IDH2^R140Q^ mutant (PDB: 5I96) was selected^24^. The crystal structure of IDH2^R140Q^ (PDB: 5I96), consists of homodimer bound to NADPH, Ca^2+^ and enasidenib. Within the homodimer interface, the allosteric binding site is located^23^. The pocket is encapsulated by four helices (α9, α10, α9’, α10’) lining the sides, two loops (L1 and L1’), and the Tyr311–Asp312 interaction pairs capping the ends. The binding scores (S) and amino acids in the allosteric site of IDH2^R140Q^ that interact with inhibitors **6c**, **6e**, **7c**, and enasidenib are illustrated in Table 6. Enasidenib, the co-crystallised drug, is a non-competitive inhibitor^24^. Enasidenib binds by multiple H-bonds and hydrophobic interactions. The 2-methyl-2-propanol moiety donates a H-bond to Gln316, while Gln316’ accepts a H-bond from linker amine, in addition to forming a H-bond with *s*-triazine core nitrogen. Additional H-bond between enasidenib’s trifluoromethyl moiety and Asp312’ is observed as shown in Figure 11(A). Other hydrophobic interactions from surrounding hydrophobic residues include, Trp164’, Val294’, Val297’, Try311, Try311’, Asp312’, Val315, Val315’, Gln316’, Ile319’, and Leu320’.

**Table 6.** Docking results and interacting residues for inhibitors, **6c**, **6e**, **7c**, and enasidenib in IDH2^R140Q^ allosteric site (PDB ID: 5I96).

Cpd.	Score (Kcal/mol)	Amino acids that interact with			
		Head	Central core	Linker	Tail
**6c**	−8.03	Asp312, Val315, Val 315’,	Val294’, Gln316’, Ile319’	Gln316, Gln316’	Leu160, Leu160’, Trp164’, Val294, Val297, Val297’, Leu298’, Trp306, Ile319, Ile319’, Leu320
**6e**	−8.12	Val294, Val297, Leu298, Ile319, Gln316	Val294’, Gln316’, Ile319, Ile319’	Val315, Gln316, Ile319’	Leu160’, Trp164’, Asp312’, Val315’, Val316, Ile319’, Leu320’, Gly323’
**7c**	−8.08	Val315, Val315’	Val294’, Gln316’, Ile319, Ile319’	Gln316, Gln316’, Val294’	Val297’, Gln316’, Ile319, Ile319’, Leu320’
**En.** ^a^	−11.13	Gln316	Val294’, Gln316’, Ile319, Ile319’	Gln316’	Trp164’, Val294’, Val297’, Try311, Try311’, Asp312’, Val315, Val315’, Gln316’, Ile319’, Leu320’

^a^En: Enasidenib.

Molecular docking of **6c** verified three H-bond between **6c** and IDH2^R140Q^ where the *s*-triazine core and one NH group of the linker formed two H-bonds with amide and carbonyl of Gln316’ and Gln316, respectively, while H-bond between **6c** imine carbon and carbonyl of Gln316’ was recognised. The aromatic tails formed van der Waals attraction forces with Leu160, Trp164, Val294, Val297, Leu198, Ile319, and Trp306. Moreover, morpholine head established hydrophobic interaction with Val315 as demonstrated in Figure 11(B). Docking mode of **6e** revealed 3H-bonds with Gln316 and Val315 where two H-bonds with NH group and *s*-triazine nitrogen were observed. The third H-bond was established with NH group of the second linker. While 3-fluorophenyl group of compound **6e** formed halogen bond with Ileu319. In addition, compound **6e** displayed hydrophobic attraction forces between the aromatic tails and amino acid residues, Leu160’, Trp164’, Val315’, and Ile319’. Another hydrophobic interaction between morpholine carbons and Val297, Leu298, and Ile319, is reported as demonstrated in Figure 11(C).

**Figure 12.**
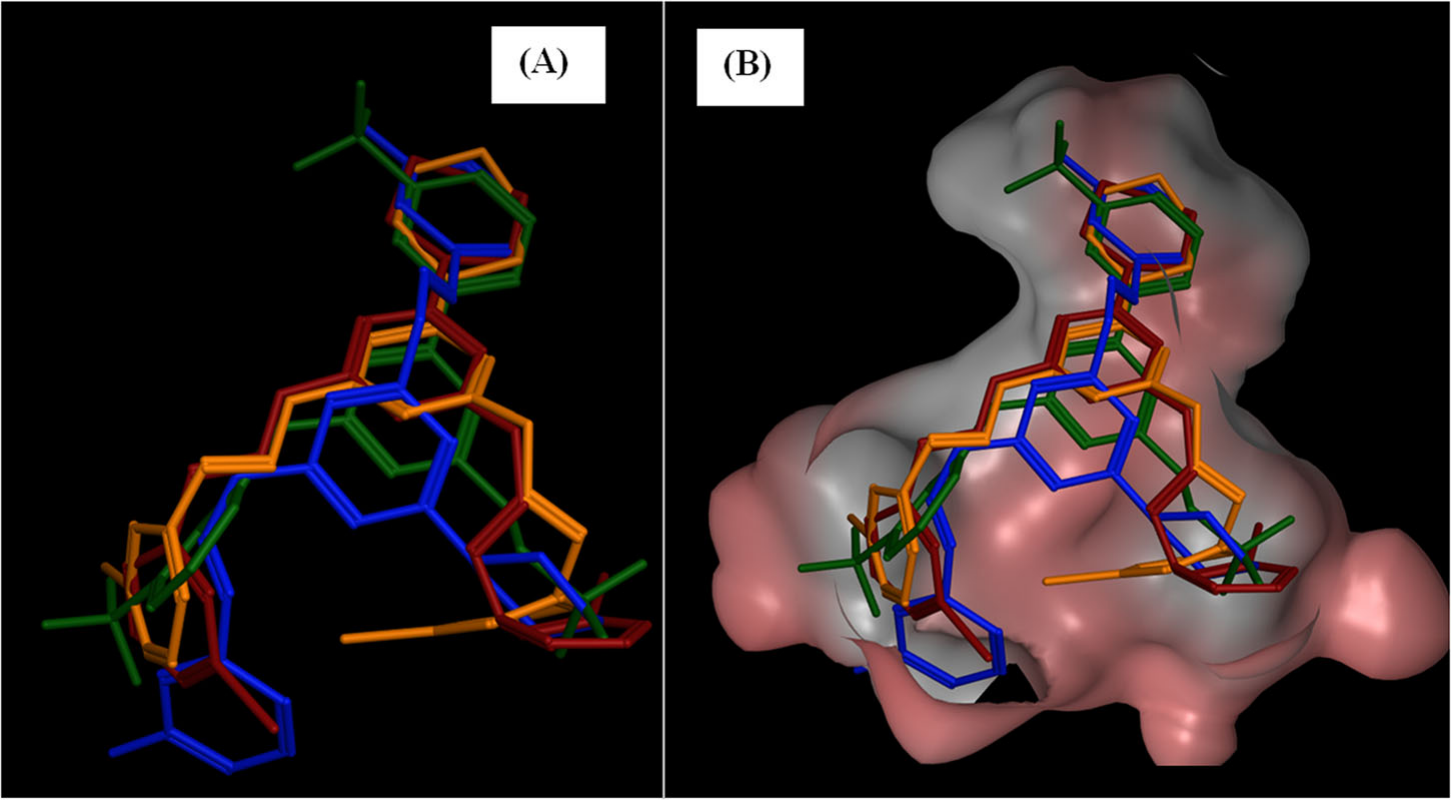
Alignment of compounds **6c** (dark red), **6e** (blue), and **7c** (orange) and enasidenib (green) in the IDH2^R140Q^ allosteric site viewing parallel layout.

While compound **7c** adapted two more polar interactions than **6c** which were one H-bond between Gln316’ and NH group while the second was halogen bond between bromine and two surrounding residues, Gln316’ and Leu320’ as exposed in Figure 11(D). Analogues **6c**, **6e**, and **7c** disclosed parallel layout upon alignment with enasidenib in the allosteric site of IDH2^R140Q^ as illustrated in Figure 12.

#### In silico physicochemical, pharmacokinetic prediction, and PAINS filters

**Figure 13.**
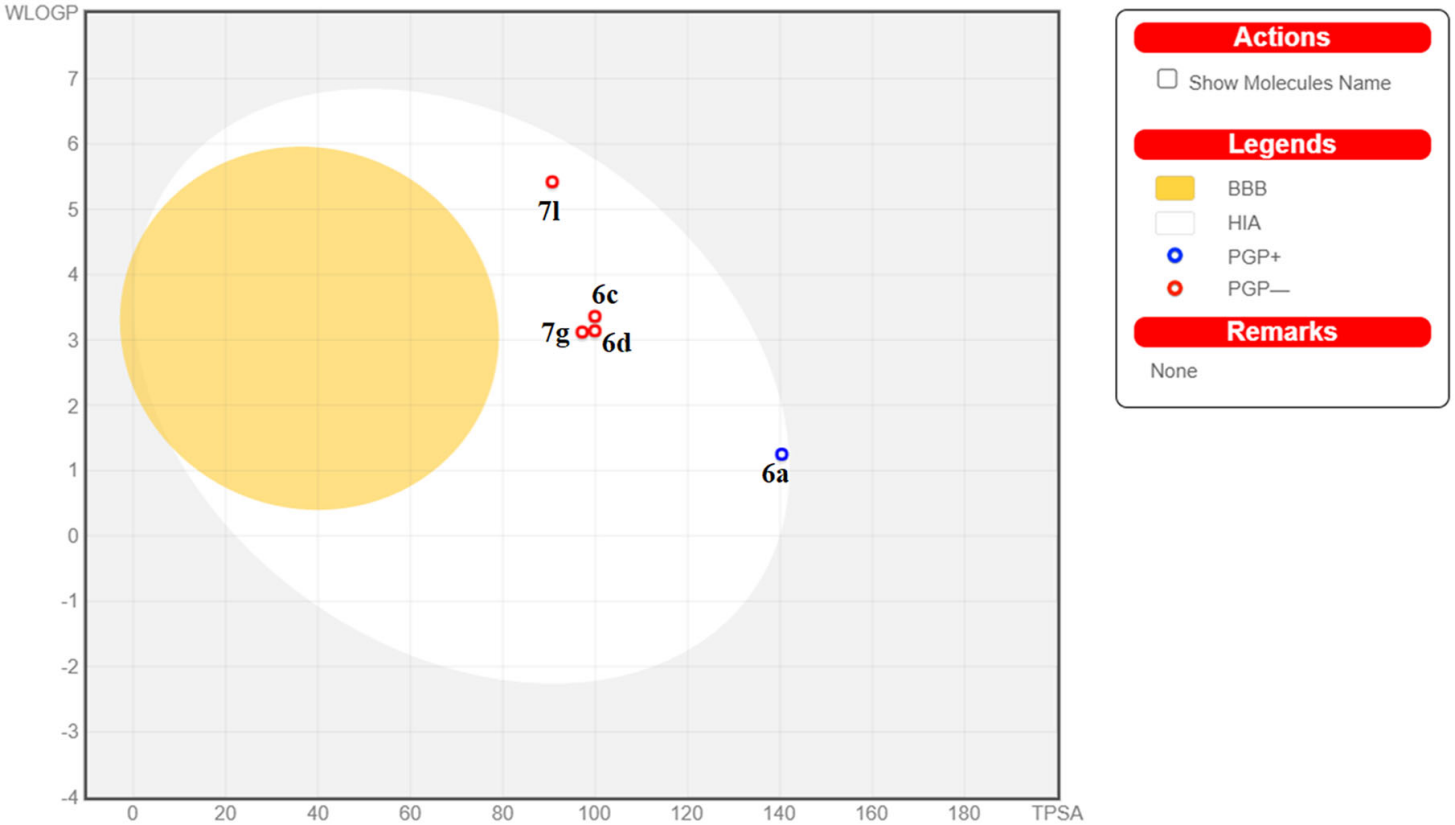
Predicted BOILED-Egg for compounds **6a**, **6c**, **6d, 7g**, and **7l**. BBB: blood–brain barrier; HIА; human intestinal absorption; PGP+: P-glycoprotein substrate; PGP-: not P-glycoprotein substrate.

To predict the physicochemical and drug-likeness properties of the most potent compounds **6a**, **6c**, **6d**, **7g**, and **7l** based on NCI *in vitro* results, SwissADME free online web tool was applied (http://www.swissadme.ch/, accessed on 24 July 2022). The brain or intestinal estimated permeation (BOILED-Egg) model was developed by calculating both lipophilicity using the Wildman log P method (WLOGP) and polarity expressed in topological polar surface area (TPSA), followed by plotting the relationship between them in a BOILED-Egg diagram^47^. Therefore, we can predict both gastrointestinal absorption and BBB permeability for the tested compounds. The obtained results predicted that physicochemical and pharmacokinetics of the five compounds were in acceptable ranges. All five compounds appeared in the zone of human intestinal absorption (HIA) with no blood–brain barrier (BBB) permeability. Hence, they have high oral bioavailability with a privilege of having a good CNS safety profile. BOILED-Egg was assembled for the tested compounds as illustrated in Figure 13.

In addition, compounds **6c**, **6d**, **7g**, **and 7l** were not P-glycoprotein substrates (Pgp–); thus, they are not susceptible to the efflux mechanism by the Pgp transporter, which is a mechanism that emerged by some tumour cells as a drug resistance strategy^48^. Compound **6a** was predicted to be a P-glycoprotein substrate. Furthermore, SwissADME revealed that compounds **6c**, **6d**, and **7g** fulfilled Lipinski (Pfizer), Veber (GSK), and Egan (Pharmacia) filters predicting that these compounds have promising drug-likeness profiles (Table 7).

**Table 7.** Drug-likeness profiles for compounds **6a, 6c, 6d, 7g,** and **7l** and number of rules they fulfilled.

Drug-likeness					
	6a	6c	6d	7g	7l
Lipinski	Pass	Pass	Pass	Pass	Failed
Ghose	Pass	Failed	Failed	Failed	Failed
Veber	Failed	Pass	Pass	Pass	Pass
Egan	Failed	Pass	Pass	Pass	Pass
Muegge	Pass	Failed	Failed	Failed	Failed

**Figure 14.**
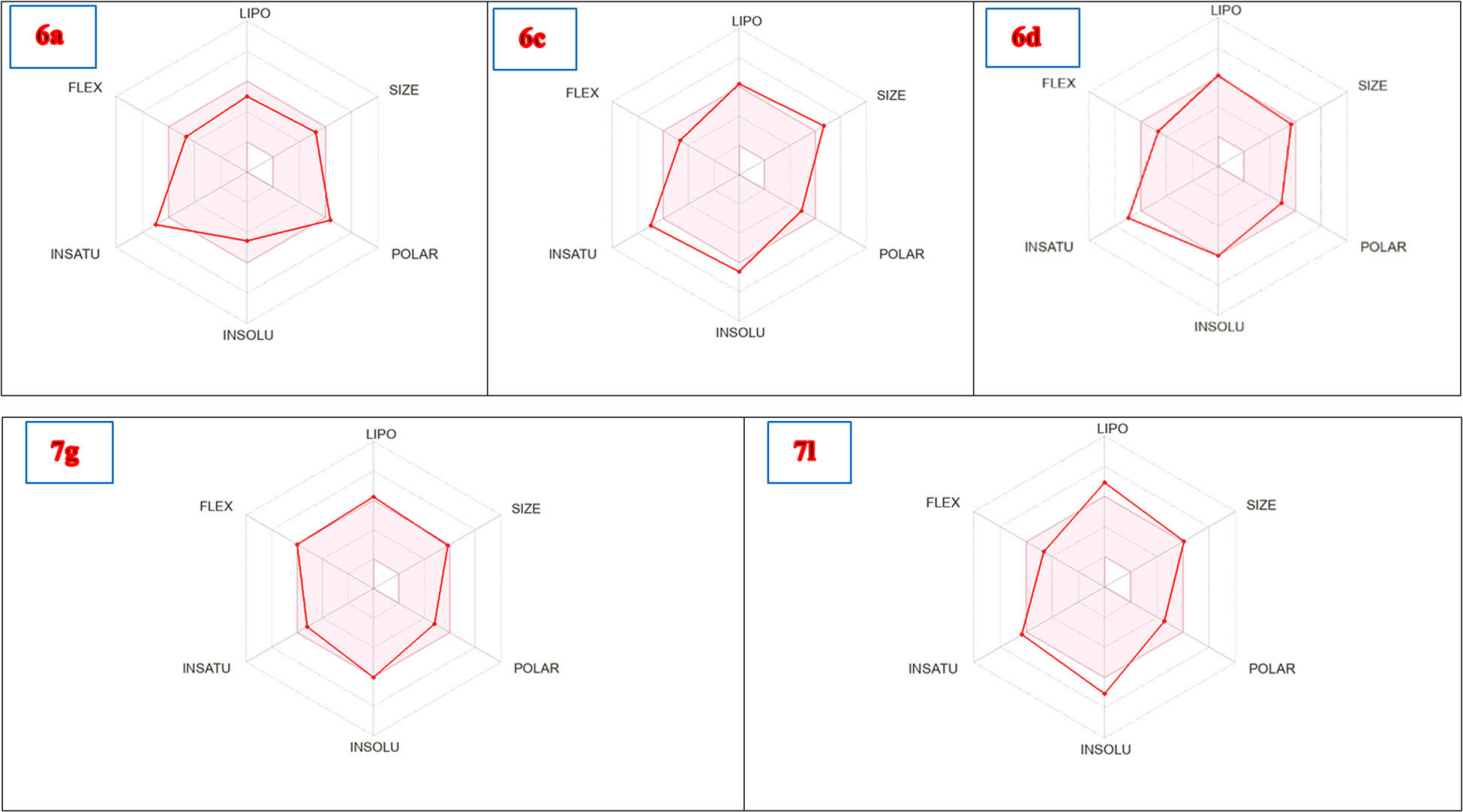
Radar charts for prediction of oral bioavailability profiles of compounds **6a**, **6c**, **6d**, **7g**, and **7l** represented by red line, and the range of optimal property values are shown in pink.

Moreover, the bioavailability radar which composed of the calculation of six parameters including size, lipophilicity, polarity, saturation, flexibility, and solubility showed that these compounds (represented by red lines) are almost predicting acceptable oral bioavailability with **7g** revealed the best fit among these compounds (Figure 14).

What’s more, swissADME-achieved data classified compounds **6c**, **6d**, and **7l** as non-PAINS (pan-assay interference compounds), signifying the high selectivity of our target compounds.

## Conclusion

Three novel series of *s*-triazines were designed and synthesised as inhibitors of IDH2^R140Q^ relying on the structural futures of the marketed Enasidenib and applying the symmetric dual-tail approach. In series (**I**), 12 new compounds **6a–l** were constructed with a central *s*-triazine scaffold with morpholine, as the heterocyclic head, and two groups of substituted aromatic rings as the lipophilic symmetric tails connected to the s-triazine core with a methylene hydrazine linker. Regarding series **(II),** 12 novel compounds, **7a–l,** were designed as analogues to series **(I)** with piperidine as the heterocyclic head. In series **(III)**, 4 new target compounds, **8a–d**, were assembled with *s*-triazine scaffold, morpholine head, and two symmetric lipophilic tails connected to *s*-triazine ring with diverse manipulated linkers. Target compounds were assessed as anticancer agents against a panel of sixty cancer cell lines, following the US-NCI protocol. Relying on NCI single-dose screening outcomes, compounds in series **(I)** and their corresponding analogues in series **(II)** demonstrated an overall comparable activity, while Target compounds in series **(III)** disclosed lower potency. The most active analogue, **6c** displayed high antiproliferative activity against NCI leukaemia subpanels with GI_50_ in nanomolar range and very good activity in inhibiting IDH2^R140Q^ enzyme (IC_50_ = 101.7 nM) while minimal inhibition was reported against wild type (IC_50_ = 2928 nM) *in vitro*. Moreover, **6c** showed better safety as a cytotoxic agent towards normal embryonic kidney cells (HEK 293), IC_50_ = 53.69 μM in comparison to staurosporine, IC_50_ = 35.33 μM. Cell cycle analysis was performed to measure the effect of compound **6c** on induction of cell cycle for leukaemia HL-60(TB) cancer cells. It arrested cell growth in G0–G1 and S phases with alteration of the pre-G1 phase. Moreover, compound **6c** induced necrosis by 7 times more than the control (DMSO) against HL-60(TB) cancer cells. Treatment of HL-60(TB) cancer cells with compound **6c** significantly increased the expression levels of the apoptotic markers, active Caspases **3** and **9** by about 3- and 4-fold, respectively, in comparison to the control. Computational docking study for selected compounds demonstrated strong binding interactions with variable amino acid residues in the allosteric site of IDH2^R140Q^. Moreover, pharmacokinetic, and physicochemical properties of the selected inhibitors revealed high predicted oral bioavailability and promising drug-likeness profiles.

## Materials and methods

### Chemistry

#### General

All organic reagents used were obtained from Alfa Aesar, Sigma-Aldrich, or Merck Company and were used without any further purification. Melting points were determined on an electrothermal melting point apparatus (Stuart SMP10) by the open capillary method and were reported uncorrected. Reactions were monitored by TLC using pre-coated sheet (Fastman Kodak Co., Rochester, NY; Silica 60 F_254_) using developing systems, chloroform: ethanol and *n*-hexane: ethyl acetate and were visualised using UV lamp at 254 nm. Elemental analysis was carried out by Perkin–Elmer 2400 CHNS analyser and the results were obtained within ± 0.40 of the theoretical values. Elemental analysis was performed by the regional centre for mycology and biotechnology, Al-Azhar University, Cairo, Egypt. ^1^H, ^13^C NMR, NOESY spectra were recorded on Bruker FT-NMR spectrometer at (400 MHz) and (100 or 125 MHz), respectively using DMSO-*d*_6_ as a solvent. Values of chemical shift, coupling constants, *J*, and multiplicity (s = singlet, d = doublet, t = triplet, m = multiplet, *br* = broad, *br*. s = broad singlet) were reported in ppm and in Hertz (Hz), respectively. ^1^H and ^13^C NMR experiments were performed by Faculty of Pharmacy and Faculty of Science, Mansoura University, Mansoura, Egypt. Electron ionisation mass spectra (EI-MS) were recorded on Thermo Scientific, ISQ Single Quadruple MS, with ionisation energy of 70 eV, and Helium gas was used as the carrier gas at a constant flow rate of 1 mL/min. Mass analysis was performed by the regional centre for mycology and biotechnology, Al-Azhar University, Cairo, Egypt.

#### *General procedure for preparation of compounds 3a*,*b*

A solution of morpholine, **2a** (0.871 g, 10 mmol) or piperidine, **2b** (0.851 g, 10 mmol) in acetone was added dropwise over a period of 1 h to a stirred solution of cyanuric chloride **1** (1.845 g, 10 mmol) in 30 ml acetone at 0–5 °C. Sodium carbonate solution was added after 4 h with vigorous stirring until pH reached 7–8. The slowly precipitated crystals were filtered off and dried^49^^,^^50^.

#### 2,4-Dichloro-6-(morpholin-4-yl)-1,3,5-triazine (3a)

An off-white powder, yield: (1.88 g, 80%). Mp: 159–160 °C. [Lit.^51^. Mp: 157–158 °C].

#### 2,4-Dichloro-6-(piperidin-1-yl)-1,3,5-triazine (3b)

An off-white powder, yield: (1.92 g, 82.5%). Mp: 90–91 °C. [Lit.^52^. Mp: 90–91 °C].

#### *General procedure for preparation of compounds 4a*,*b*

In a 250 mL round bottom flask, hydrazine hydrate (99%, 10 mL) was stirred with acetonitrile (25 mL) at room temperature. Then suspension of compound **3a** (2.35 g, 10 mmol) or **3b** (2.33 g, 10 mmol) in acetonitrile (25 mL) was added in portions. When the addition completed, the reaction mixture was heated under reflux for 6 h and then, cooled to room temperature. The precipitated powder was filtered off, washed several times with acetonitrile and finally with diether ether then dried to afford the pure products^51^^,^^52^.

#### 2,4‐Dihydrazinyl‐6‐(morpholin‐4‐yl)‐1,3,5‐triazine (4a)

White powder, yield (2.05 g, 91%). Mp: 205–207 °C [Lit.^51^. Mp: 215–217 °C].

#### 2,4‐Dihydrazinyl‐6‐(piperidin‐1‐yl)‐1,3,5‐triazine (4b)

White powder, yield: (1.97 g, 88%). Mp: 160–162 °C [Lit.^51^^,^^52^. Mp: 160–162 °C].

#### *General procedure for preparation of compounds, 6a–l, 7a–l*, *and 8a*,*b*

2,4-Dihydrazino-6-substituted-1,3,5-triazine derivatives 4a-b (1.3 mmol) were added portion-wise to a hot solution of ethanol (30 mL) containing aldehydes, **5a–l**, 4-nitroacetophenone, or 5-chloroisatin (2.6 mmol), and 2–3 drops of *glacial* acetic acid. The reaction mixture was heated under reflux for 4–8 h and the progress of the reaction was followed by TLC using ethyl acetate: *n-*hexane (2:1) as eluent. The reaction was left to cool down to room temperature and then the product was filtered off and recrystallised from acetone^53^.

##### 2-[(1e)-(2-{4-[(E)-2-[(2-Hydroxyphenyl)methylidene]hydrazin-1-yl]-6-(morpholin-4-yl)-1,3,5-triazin-2-yl}hydrazin-1-ylidene)methyl]phenol (6a)

Yield: (0.32 g, 56.40%) as a white powder with Mp: 258–259 °C. ^1^H NMR (400 MHz, DMSO-d_6_) δ (ppm): 3.69 (s, 4H, (CH_2_)_2_N), 3.81 (s, 4H, (CH_2_)_2_O), 6.89–6.94 (m, 4H, 3,5-H_2_, 2XAr-H), 7.26 (t, 2H, *J* = 7.60 Hz, 4-H, 2XAr-H), 7.40 (s, 2H, 6-H, 2XAr-H), 8.32 (s, 2H, 2XHC = N), 11.31 (*br.* s, 2H, 2XOH), 12.04 (s, 2H, 2XNH). ^13^C NMR (125 MHz, DMSO) δ (ppm): 43.29, 65.95, 116.40, 118.81, 119.18, 129.86, 130.39, 144.19, 157.44, 163.83, 164.66. EI-MS: *m*/*z*: 434.57 [M^+^]. Anal. Calcd. For C_21_H_22_N_8_O_3_: C, 58.06; H, 5.1; N, 25.79. Found: C, 58.23; H, 5.3; N, 25.99.

##### 2,4-Bis[(E)-2-[(2-bromophenyl)methylidene]hydrazin-1-yl]-6-(morpholin-4-yl)-1,3,5-triazine (6b)

Yield: (0.37 g, 50.40%) as an off-white powder with Mp: 286–288 °C. ^1^H NMR (400 MHz, DMSO-d_6_) δ (ppm): 3.72 (s, 4H, (CH_2_)_2_N), 3.85 (s, 4H, (CH_2_)_2_O), 7.43–7.73 (m, 6H, 3,4,5-H_3_, 2XAr-H), 8.13 (*br*. s, 2H, 6-H, 2XAr-H), 8.63 (s, 2H, 2XCH = N), 11.34 (*br.* s, 1H, NH). ^13^C NMR (125 MHz, DMSO) δ (ppm): 44.20, 65.79, 123.86, 127.36, 128.02, 131.71, 132.22, 132.66, 133.32, 145.73. EI-MS: *m*/*z*: 560.47 [M^+^]. Anal. Calcd. For C_21_H_20_Br_2_N_8_O: C, 45.02; H, 3.6; N, 20. Found: C, 45.30; H, 3.8; N, 20.21.

##### 2,4-Bis[(E)-2-[(3-bromophenyl)methylidene]hydrazin-1-yl]-6-(morpholin-4-yl)-1,3,5-triazine (6c)

Yield (0.55 g, 74%) as a white powder with Mp: 186–188 °C. ^1^H NMR (400 MHz, DMSO-d_6_) δ (ppm): 3.68 (s, 4H, (CH_2_)_2_N), 3.80 (s, 4H, (CH_2_)_2_O), 7.41 (t, 2H, *J* = 7.20 Hz, 5-H, 2XAr-H), 7.59 (d, 2H, *J* = 7.2 Hz, 4-H, 2XAr-H), 7.68 (s, 2H, 6-H, 2XAr-H), 7.90 (s, 2H, 2-H, 2XAr-H), 8.14 (s, 2H, 2XHC = N), 11.31 (*br*. s, 2H, 2XNH). ^13^C NMR (125 MHz, DMSO) δ (ppm): 43.50, 66.02, 122.19, 125.75, 128.99, 130.96, 131.89, 137.25, 141.27, 163.61, 164.54. EI-MS: *m*/*z*: 560.44 [M^+^]. Anal. Calcd. For C_21_H_20_Br_2_N_8_O: C, 45.02; H, 3.6; N, 20. Found: C, 45.21; H, 3.7; N, 20.19.

##### 2,4-Bis[(E)-2-[(3-chlorophenyl)methylidene]hydrazin-1-yl]-6-(morpholin-4-yl)-1,3,5-triazine (6d)

Yield (0.25 g, 40%) as a white powder with Mp: 260–262 °C. ^1^H NMR (400 MHz, DMSO-d_6_) δ (ppm): 3.71 (s, 4H, (CH_2_)_2_N), 3.85 (s, 4H, (CH_2_)_2_O), 7.53 (s, 4H, 4,5-H_2_, 2XAr-H), 7.82 (s, 2H, 6-H, 2XAr-H), 7.99 (s, 2H, 2-H, 2XAr-H), 8.25 (s, 2H, 2XHC = N), 13.30 (*br.* s, 2H, 2XNH). ^13^C NMR (125 MHz, DMSO) δ (ppm): 44.30, 65.74, 125.69, 127.19, 127.87, 130.07, 130.73, 133.76, 135.38, 135.90, 146.37, 161.08, 161.45. EI-MS: *m*/*z*: 471.49 [M^+^]. Anal. Calcd. For C_21_H_20_Cl_2_N_8_O: C, 53.51; H, 4.28; N, 23.77. Found: C, 53.61; H, 4.21; N, 23.51.

##### 2,4-Bis[(E)-2-[(3-Fluorophenyl)methylidene]hydrazin-1-yl]-6-(morpholin-4-yl)-1,3,5-triazine (6e)

Yield (0.2 g, 34.4%) as a white powder with Mp: 284–286 °C. ^1^H NMR (400 MHz, DMSO-d_6_) δ (ppm): 3.72 (s, 4H, (CH_2_)_2_N), 3.86 (s, 4H, (CH_2_)_2_O), 7.37 (s, 2H, 4-H, 2XAr-H), 7.54–7.77 (m, 6H, 2,5,6-H_3_, 2XAr-H), 8.30 (s, 2H, 2XHC = N), 13.24 (*br.* s, 1H, NH). ^13^C NMR (125 MHz, DMSO) δ (ppm): 44.39, 65.74, 113.35, 113.50, 117.18, 117.69, 123.87, 130.94, 135.76, 146.53, 161.45, 163.40 EI-MS: *m*/*z*: 438.54 [M^+^]. Anal. Calcd. For C_21_H_20_F_2_N_8_O: C, 57.53; H, 4.6; N, 25.56. Found: C, 57.74; H, 4.85; N, 25.82.

##### 2-(Morpholin-4-yl)-4,6-bis[(E)-2-[(3-nitrophenyl)methylidene]hydrazin-1-yl]-1,3,5-triazine (6f)

Yield (0.37 g, 56.60%) as a yellow powder with Mp: 275–277 °C. ^1^H NMR (400 MHz, DMSO-*d*_6_) δ (ppm): 3.69 (s, 4H, (CH_2_)_2_N), 3.81 (s, 4H, (CH_2_)_2_O), 7.74 (t, 2H, *J* = 8.00 Hz, 5-H, 2XAr-H), 8.10 (s, 2H, 4-H, 2XAr-H), 8.22 (d, 2H, *J* = 8.00 Hz, 6-H, 2XAr-H), 8.27 (s, 2H, 2XCH = N), 8.50 (s, 2H, 2-H, 2XAr-H), 11.33 (s, 2H, 2XNH). ^13^C NMR (100 MHz, DMSO) δ (ppm): 43.81, 66.49, 120.83, 123.78, 130.79, 133.10, 137.33, 140.55, 148.69, 164.92, 165.23. EI-MS: *m*/*z*: 492.12 [M^+^]. Anal. Calcd. For C_21_H_20_N_10_O_5_: C, 51.22; H, 4.09; N, 28.44. Found: C, 51.01; H, 4.26; N, 28.22.

##### 4-[(1e)-(2-{4-[(E)-2-{[4-(dimethylamino)phenyl]methylidene}hydrazin-1-yl]-6-(morpholin-4-yl)-1,3,5-triazin-2-yl}hydrazin-1-ylidene)methyl]-N,N-dimethylaniline (6g)

Yield (0.24 g, 37.80%) as a yellow powder crystallised from acetone: ethanol mixture (1:1) with Mp: 228–229 °C. ^1^H NMR (400 MHz, DMSO-d_6_) δ (ppm): 3.02 (s, 12H, 4XCH_3_), 3.71 (s, 4H, (CH_2_)_2_N), 3.83 (s, 4H, (CH_2_)_2_O), 6.77 (d, 4H, *J* = 8.40 Hz, 3,5-H_2_, 2XAr-H), 7.63 (d, 4H, *J* = 8.40 Hz, 2,6-H_2_, 2XAr-H), 8.15 (s, 2H, 2XHC = N), 13.24 (*br.* s, 2H, 2XNH). ^13^C NMR (100 MHz, DMSO) δ (ppm): 40.38, 44.85, 66.20, 112.03, 120.46, 129.17, 148.88, 152.44, 153.02, 161.73. EI-MS: *m*/*z*: 488.25 [M^+^]. Anal. Calcd. For C_25_H_32_N_10_O: C, 61.46; H, 6.6; N, 28.67. Found: C, 61.71; H, 6.81; N, 28.88.

##### 2,4-Bis[(E)-2-[(2,3-Dichlorophenyl)methylidene]hydrazin-1-yl]-6-(morpholin-4-yl)-1,3,5-triazine (6h)

Yield (0.20 g, 28.10%) as a white powder with Mp: 229–230 °C. ^1^H NMR (400 MHz, DMSO-d_6_) δ (ppm): 3.70 (s, 4H, (CH_2_)_2_N), 3.84 (s, 4H, (CH_2_)_2_O), 7.49 (s, 2H, 5-H, 2XAr-H), 7.73 (s, 2H, 4-H, 2XAr-H), 8.11 (s, 2H, 6-H, 2XAr-H), 8.63 (s, 2H, 2XHC = N), 11.97 (*br.* s, 2H, 2XNH). ^13^C NMR (125 MHz, DMSO) δ (ppm): 44.13, 65.84, 125.95, 128.37, 132.45. EI-MS: *m*/*z*: 540.94 [M^+^]. Anal. Calcd. For C_21_H_18_Cl_4_N_8_O: C, 46.69; H, 3.36; N, 20.74. Found: C, 46.87; H, 3.62; N, 20.46.

##### 2,4-Bis[(E)-2-[(4-Chloro-3-nitrophenyl)methylidene]hydrazin-1-yl]-6-(morpholin-4-yl)-1,3,5-triazine (6i)

Yield (0.40 g, 53.70%) as a yellow powder with Mp: 293–295 °C. ^1^H NMR (400 MHz, DMSO-d_6_) δ (ppm): 3.68 (s, 4H, (CH_2_)_2_N), 3.80 (s, 4H, (CH_2_)_2_O), 7.82–7.98 (m, 4H, 5,6-H_2_, 2XAr-H), 8.19 (s, 2H, 2XHC = N), 8.30 (s, 2H, 2-H, 2XAr-H), 11.40 (s, 2H, 2XNH). ^13^C NMR (100 MHz, DMSO) δ (ppm): 43.81, 66.48, 123.41, 125.18, 131.29, 132.55, 136.13, 139.51, 148.31, 164.85, 165.21 EI-MS: *m*/*z*: 561.53 [M^+^]. Anal. Calcd. For C_21_H_18_Cl_2_N_10_O_5_: C, 44.93; H, 3.23; N, 24.95. Found: C, 45.1; H, 3.50; N, 24.89.

##### 4-[(1e)-(2-{4-[(E)-2-[(3,4-Dihydroxyphenyl)methylidene]hydrazin-1-yl]-6-(morpholin-4-yl)-1,3,5-triazin-2-yl}hydrazin-1-ylidene)methyl]benzene-1,2-diol (6j)

Yield (0.48 g, 78.40%) as a buff powder with Mp: 272 °C (decom.). ^1^H NMR (400 MHz, DMSO-d_6_) δ (ppm): 3.67 (s, 4H, (CH_2_)_2_N), 3.78 (s, 4H, (CH_2_)_2_O), 6.77 (d, 2H, *J* = 8.00 Hz, 5-H, 2XAr-H), 6.87 (d, 2H, *J* = 8.00 Hz, 6-H, 2XAr-H), 7.13 (s, 2H, 2-H, 2XAr-H), 7.96 (s, 2H, 2XHC = N), 9.15 (s, 2H, 2XOH), 9.30 (s, 2H, 2XOH), 10.70 (*br.* s, 2H, 2XNH). ^13^C NMR (100 MHz, DMSO) δ (ppm): 43.76, 66.57, 113.14, 116.03, 119.96, 127.00, 143.65, 146.01, 147.52, 164.62, 165.31. EI-MS: *m*/*z*: 466.24 [M^+^]. Anal. Calcd. For C_21_H_22_N_8_O_5_: C, 54.07; H, 4.75; N, 24.02. Found C, 54.31; H, 5.04; N, 24.31.

##### 2,4-Bis[(E)-2-(2H-1,3-Benzodioxol-5-ylmethylidene)hydrazin-1-yl]-6-(morpholin-4-yl)-1,3,5-triazine (6k)

Yield (0.38 g, 58.40%) as a white powder with Mp: 272 °C (decom.). ^1^H NMR (400 MHz, DMSO-d_6_) δ (ppm): 3.71 (s, 4H, (CH_2_)_2_N), 3.84 (s, 4H, (CH_2_)_2_O), 6.12–6.15 (m, 4H, 2XOCH_2_O), 7.05–7.40 (m, 6H, 2,5,6-H_3_, 2XAr-H), 8.14 (s, 1H, HC = N), 8.22 (s, 1H, HC = N’), 13.08 (*br.* s, 2H, 2XNH). ^13^C NMR (125 MHz, DMSO) δ (ppm): 44.38, 65.71, 101.59, 101.89, 105.61, 108.61, 123.35, 123.50, 127.40, 147.59, 148.16, 149.86. EI-MS: *m*/*z*: 490.15 [M^+^]. Anal. Calcd. For C_23_H_22_N_8_O_5_: C; 56.32, H; 4.52, N; 22.85. Found: C, 56.04; H, 4.46; N, 22.99.

##### 2,4-Bis[(E)-2-[(2-Chloro-6-fluorophenyl)methylidene]hydrazin-1-yl]-6-(morpholin-4-yl)-1,3,5-triazine (6l)

Yield (0.32 g, 47.50%) as a white powder with Mp: 242–244 °C. ^1^H NMR (400 MHz, DMSO-d_6_) δ (ppm): 3.65 (s, 4H, (CH_2_)_2_N), 3.77 (s, 4H, (CH_2_)_2_O), 7.30–7.44 (m, 6H, 3,4,5-H_6_, 2XAr-H), 8.36 (s, 2H, 2XHC = N), 11.31 (*br.* s, 2H, 2XNH). ^13^C NMR (100 MHz, DMSO) δ (ppm): 43.75, 66.45, 115.80, 116.02, 121.71, 121.85, 126.41, 131.13, 131.23, 133.85, 133.89, 135.98, 159.19, 161.73, 164.83, 165.31 EI-MS: *m*/*z*: 507.73 [M^+^]. Anal. Calcd. For C_21_H_18_Cl_2_F_2_N_8_O: C, 49.72; H, 3.58; N, 22.09. Found: C, 49.65; H, 3.37; N, 22.28.

##### 2-[(1e)-(2-{4-[(E)-2-[(2-Hydroxyphenyl)methylidene]hydrazin-1-yl]-6-(piperidin-1-yl)-1,3,5-triazin-2-yl}hydrazin-1-ylidene) methyl] phenol (7a)

Yield: (0.42 g, 74.10%) as a white powder with Mp: 278–280 °C. ^1^H NMR (400 MHz, DMSO-d_6_) δ (ppm): 1.55 (s, 4H, 2XCH_2_), 1.66 (s, 2H, CH_2_), 3.82 (s, 4H, (CH_2_)_2_N), 6.90–6.94 (m, 4H, 3,5-H_2_, 2XAr-H), 7.26 (t, 2H, *J* = 8.00 Hz, 4-H, 2XAr-H), 7.40 (s, 2H, 6-H, 2XAr-H), 8.30 (s, 2H, 2XHC = N), 11.23 (s, 2H, 2XOH), 12.07 (s, 1H, NH). ^13^C NMR (125 MHz, DMSO) δ (ppm): 24.37, 25.46, 43.63, 116.40, 118.84, 119.13, 129.84, 130.28, 143.96, 157.43, 163.90, 164.22. EI-MS: *m*/*z*: 432.68 [M^+^]. Anal. Calcd. For C_22_H_24_N_8_O_2_: C, 61.1; H, 5.59; N, 25.91. Found: C, 61.25; H, 5.78; N, 25.99.

##### 2,4-Bis[(E)-2-[(2-Bromophenyl)methylidene]hydrazin-1-yl]-6-(piperidin-1-yl)-1,3,5-triazine (7b)

Yield (0.55 g, 74.20%) as a white powder with Mp: 260–262 °C. ^1^H NMR (400 MHz, DMSO-d_6_) δ (ppm): 1.54 (s, 4H, 2XCH_2_), 1.64 (s, 2H, CH_2_), 3.81 (s, 4H, (CH_2_)_2_N), 7.32 (t, 2H, *J* = 8.00 Hz, 4-H, 2XAr-H), 7.47 (t, 2H, *J* = 8.00 Hz, 5-H, 2XAr-H), 7.67 (d, 2H, *J =* 8.00 Hz, 3-H, 2XAr-H), 7.97 (d, 2H, *J* = 8.00 Hz, 6-H, 2XAr-H), 8.51 (s, 2H, 2XHC = N), 11.29 (s, 2H, 2XNH). ^13^C NMR (125 MHz, DMSO) δ (ppm): 24.36, 25.50, 43.58, 122.78, 126.84,127.93, 130.74, 133.09, 133.78, 140.56, 164.38. EI-MS: *m*/*z*: 558.50 [M^+^]. Anal. Calcd. For C_22_H_22_Br_2_N_8_: C, 47.33; H, 3.97; N, 20.07. Found: C, 47.54; H, 4.09; N, 20.22.

##### 2,4-Bis[(E)-2-[(3-Bromophenyl)methylidene]hydrazin-1-yl]-6-(piperidin-1-yl)-1,3,5-triazine (7c)

Yield (0.41 g, 55.30%) as a white powder with Mp: 210–212 °C. ^1^H NMR (400 MHz, DMSO-d_6_) δ (ppm): 1.54 (s, 4H, 2XCH_2_), 1.65 (s, 2H, CH_2_), 3.81 (s, 4H, (CH_2_)_2_N), 7.41–7.64 (m, 6H, 4,5,6-H_3_, 2XAr-H), 7.87 (s, 2H, 2-H, 2XAr-H), 8.11 (s, 2H, 2XHC = N), 11.13 (s, 2H, 2XNH). ^13^C NMR (125 MHz, DMSO) δ (ppm): 24.36, 25.49, 43.55, 122.15, 125.58, 128.39, 130.94, 131.53, 137.56, 140.26, 164.31, 164.45 EI-MS: *m*/*z*: 558.57 [M^+^]. Anal. Calcd. For C_22_H_22_Br_2_N_8_: C, 47.33; H, 3.97; N, 20.07. Found: C, 47.52; H, 4.08; N, 20.13.

##### 2,4-Bis[(E)-2-[(3-Chlorophenyl)methylidene]hydrazin-1-yl]-6-(piperidin-1-yl)-1,3,5-triazine (7d)

Yield (0.20 g, 32.10%) as a white powder with Mp: 269–271 °C. ^1^H NMR (400 MHz, DMSO-d_6_) δ (ppm): 1.61 (s, 4H, 2x CH_2_), 1.68 (s, 2H, CH_2_), 3.86 (s, 4H, (CH_2_)_2_N), 7.55 (s, 4H, 4,5-H_2_, 2XAr-H), 7.84 (s, 2H, 6-H, 2XAr-H), 7.98 (s, 2H, 2-H, 2XAr-H), 8.29 (s, 2H, 2XHC = N), 13.12 (*br*. s, 1H, NH). ^13^C NMR (125 MHz, DMSO) δ (ppm): 23.80, 25.45, 44.90, 125.80, 127.19, 130.76, 133.77, 146.26. EI-MS: *m*/*z*: 469.40 [M^+^]. Anal. Calcd. For C_22_H_22_Cl_2_N_8_: C, 56.3; H, 4.72; N, 23.87. Found: C, 56.51; H, 4.91; N, 23.98.

##### 2,4-Bis[(E)-2-[(3-Fluorophenyl)methylidene]hydrazin-1-yl]-6-(piperidin-1-yl)-1,3,5-triazine (7e)

Yield (0.23 g, 41.40%) as a white powder with Mp: 274–276 °C. ^1^H NMR (400 MHz, DMSO-d_6_) δ (ppm): 1.62 (s, 6H, 3CH_2_), 3.87 (s, 4H, (CH_2_)_2_N), 7.37 (s, 2H, 4-H, 2XAr-H), 7.56–7.70 (m, 6H, 2,5,6-H_3_, 2XAr-H), 8.33 (s, 2H, 2XHC = N), 13.21 (s, 2H, 2XNH). ^13^C NMR (125 MHz, DMSO) δ (ppm): 23.77, 25.45, 44.99, 113.28, 113.47, 117.77, 123.74, 130.91, 135.75, 146.11, 153.55, 160.48, 161.45, 163.39. EI-MS: *m*/*z*: 436.43 [M^+^]. Anal. Calcd. For C_22_H_22_F_2_N_8_: C, 60.54; H, 5.08; N, 25.67. Found: C, 60.32; H, 5.26; N, 25.49.

##### 2,4-Bis[(E)-2-[(3-Nitrophenyl)methylidene]hydrazin-1-yl]-6-(piperidin-1-yl)-1,3,5-triazine (7f)

Yield (0.36 g, 56.20%) as a white powder with Mp: 278–279 °C. ^1^H NMR (400 MHz, DMSO-d_6_) δ (ppm): 1.62 (s, 4H, 2XCH_2_), 1.69 (s, 2H, CH_2_), 3.88 (s, 4H, (CH_2_)_2_N), 7.80 (s, 2H, 5-H, 2XAr-H) 8.32 (s, 4H, 4,6-H_2_, 2XAr-H), 8.43 (s, 2H, 2H, HC = N), 8.72 (s, 2H, 2-H, 2XAr-H), 13.11 (*br.* s, 2H, 2XNH). ^13^C NMR (125 MHz, DMSO) δ (ppm): 23.79, 25.43, 44.88, 121.72, 124.83, 130.49, 133.48, 134.96, 145.57, 148.11, 153.83, 160.31. EI-MS: *m*/*z*: 490.01 [M^+^]. Anal. Calcd. For C_22_H_22_N_10_O_4_: C, 53.87; H, 4.52; N, 28.56. Found: C, 53.97; H, 4.37; N, 28.30.

#### 4-[(1e)-(2-{4-[(E)-2-{[4-(dimethylamino)phenyl]methylidene}chydrazin-1-yl]-6-(piperidin-1-yl)-1,3,5-triazin-2-yl}hydrazin-1-ylidene)methyl]-N,N-dimethylaniline (7g)

Yield (0.31 g, 48.00%) as a faint yellow powder with Mp: 267–269 °C. ^1^H NMR (400 MHz, DMSO-d_6_) δ (ppm): 1.53 (s, 4H, 2XCH_2_), 1.64 (s, 2H, CH_2_), 2.97 (s, 12H, 2XN(CH_3_)_2_), 3.78 (s, 4H, (CH_2_)_2_N), 6.76 (d, 4H, *J* = 8.00 Hz, 3,5-H_2_, 2XAr-H), 7.47 (d, 4H, *J* = 8.00 Hz, 2,6-H_2_, 2XAr-H) 8.01 (s, 2H, 2XHC = N), 10.53 (*br*. s, 2H, 2XNH). ^13^C NMR (100 MHz, DMSO) δ (ppm): 24.92, 26.03, 40.32, 43.93, 112.39, 123.16, 123.48, 128.12, 143.41, 151.33, 164.58, 164.83. EI-MS: *m*/*z*: 486.72 [M^+^]. Anal. Calcd. For C_26_H_34_N_10_: C, 64.17; H, 7.04; N, 28.78. Found: C, 64.36; H, 7.28; N, 28.91.

##### 2,4-Bis[(E)-2-[(2,3-Dichlorophenyl)methylidene]hydrazin-1-yl]-6-(piperidin-1-yl)-1,3,5-triazine (7h)

Yield (0.54 g, 75.60%) as a white powder with Mp: 275–277 °C. ^1^H NMR (400 MHz, DMSO-d_6_) δ (ppm): 1.56 (s, 4H, 2x CH_2_), 1.65 (s, 2H, CH_2_), 3.82 (s, 4H, (CH_2_)_2_N), 7.47 (t, 2H, *J* = 8.00 Hz, Ar-H), 7.68 (d, 2H, *J* = 8.00 Hz, Ar-H) 7.97 (d, 2H, *J* = 8.00 Hz, Ar-H), 8.59 (s, 2H, HC = N), 11.47 (*br.* s, 2H, NH). ^13^C NMR (125 MHz, DMSO) δ (ppm): 24.24, 25.46, 43.78, 125.09, 128.29, 130.27, 130.74, 132.31, 134.53, 138.16, 164.08. EI-MS: *m*/*z*: 538.57 [M^+^]. Anal. Calcd. For C_22_H_20_Cl_4_N_8_: C, 49.09; H, 3.75; N, 20.82. Found: C, 49.23; H, 3.96; N, 20.61.

##### 2,4-Bis[(E)-2-[(4-Chloro-3-nitrophenyl)methylidene]hydrazin-1-yl]-6-(piperidin-1-yl)-1,3,5-triazine (7i)

Yield (0.40 g, 53.90%) as a greenish white powder with Mp: 274–276 °C. ^1^H NMR (400 MHz, DMSO-d_6_) δ (ppm): 1.61 (s, 6H, 3XCH_2_), 3.85 (s, 4H, (CH_2_)_2_N), 7.89 (s, 2H, 5-H, 2XAr-H) 8.25–8.61 (m, 6H, 2,6-H_2_, 2XAr-H, 2XHC = N), 13.07 (s, 2H, 2XNH). ^13^C NMR (125 MHz, DMSO) δ (ppm): 23.83, 25.45, 44.72, 124.49, 126.16, 131.94, 132.17, 134.32, 144.17, 147.84. EI-MS: *m*/*z*: 559.75 [M^+^]. Anal. Calcd. For C_22_H_20_Cl_2_N_10_O_4_: C, 47.24; H, 3.6; N, 25.04. Found: C, 47.35; H, 3.8; N, 25.39.

##### 4-[(1e)-(2-{4-[(E)-2-[(3,4-Dihydroxyphenyl)methylidene]hydrazin-1-yl]-6-(piperidin-1-yl)-1,3,5-triazin-2-yl}hydrazin-1-ylidene)methyl]benzene-1,2-diol (7j)

Yield (0.48 g, 78.70%) as a khaki powder with Mp: 248 °C (Decom.). ^1^H NMR (400 MHz, DMSO-d_6_) δ (ppm): 1.54 (s, 4H, 2x CH_2_), 1.65 (s, 2H, CH_2_), 3.80 (s, 4H, (CH_2_)_2_N), 6.77 (s, 2H, 5-H, 2XAr-H), 6.86 (s, 2H, 6-H, 2XAr-H) 7.14 (s, 2H, 2-H, 2XAr-H), 7.96 (s, 2H, 2XHC = N), 9.17 (s, H, 2XOH), 9.30 (s, H, 2XOH), 10.58 (*br.* s, 2H, 2XNH). ^13^C NMR (100 MHz, DMSO) δ (ppm): 24.90, 26.03, 43.94, 113.08, 116.04, 119.93, 127.08, 143.36, 146.01, 147.46, 164.87. EI-MS: *m*/*z*: 464.45 [M^+^]. Anal. Calcd. For C_22_H_24_N_8_O_4_: C, 56.89; H, 5.21; N, 24.12. Found: C, 56.71; H, 5.19; N, 24.23.

##### 2,4-Bis[(E)-2-(2H-1,3-Benzodioxol-5-ylmethylidene)hydrazin-1-yl]-6-(piperidin-1-yl)-1,3,5-triazine (7k)

Yield (0.38 g, 58.60%) as white powder with Mp: 230–232 °C. ^1^H NMR (400 MHz, DMSO-d_6_) δ (ppm): 1.53 (s, 4H, 2XCH_2_), 1.64 (s, 2H, CH_2_), 3.79 (s, 4H, (CH_2_)_2_N), 6.08 (s, 4H, 2XOCH_2_O), 6.97 (d, 2H, *J* = 8.00 Hz, 5-H, 2Ar-H), 7.07 (d, 2H, *J* = 8.00 Hz, 6-H, 2XAr-H), 7.24 (s, 2H, 2-H, 2XAr-H), 8.04 (s, 2H, 2XHC = N), 10.79 (*br.* s, 2H, 2XNH). ^13^C NMR (125 MHz, DMSO) δ (ppm): 24.42, 25.52, 43.49, 101.39, 104.73, 108.45, 122.09, 129.62, 141.75, 147.87, 148.23, 164.36. EI-MS: *m*/*z*: 488.96 [M^+^]. Anal. Calcd. For C_24_H_24_N_8_O_4_: C, 59.01; H, 4.95; N, 22.94. Found: C, 59.22; H, 4.81; N, 22.76.

##### 2,4-Bis[(E)-2-[(2-Chloro-6-fluorophenyl)methylidene]hydrazin-1-yl]-6-(piperidin-1-yl)-1,3,5-triazine (7l)

Yield (0.32 g, 47.70%) as a white powder with Mp: 223–225 °C. ^1^H NMR (400 MHz, DMSO-d_6_) δ (ppm): 1.52 (s, 2H, CH_2_), 1.63 (s, 4H, 2XCH_2_), 3.78 (s, 4H, (CH_2_)_2_N), 7.29–7.34 (m, 2H, 5-H, 2XAr-H), 7.0–7.46 (m, 4H, 3,4-H_2_, 2XAr-H), 8.35 (s, 2H, 2XHC = N), 11.25 (*br*. s, 2H, 2XNH). ^13^C NMR (100 MHz, DMSO) δ (ppm): 24.80, 25.92, 43.98, 115.80, 116.02, 121.78, 121.91, 126.44, 131.06, 131.16, 133.81, 133.85, 135.62, 159.17, 161.71, 164.88 EI-MS: *m*/*z*: 505.62 [M^+^]. Anal. Calcd. For C_22_H_20_Cl_2_F_2_N_8_: C, 52.29; H, 3.99; N, 22.17. Found: C, 52.44; H, 4.1; N, 22.19.

##### 2-(Morpholin-4-yl)-4,6-bis[(E)-2-[1–(4-nitrophenyl)ethylidene]hydrazin-1-yl]-1,3,5-triazine (8a)

Yield (0.53 g, 77.60%) as a yellow powder with Mp: 199–200 °C. ^1^H NMR (400 MHz, DMSO-d_6_) δ (ppm): 2.38 (s, 6H, 2XCH_3_-C = N), 3.69 (s, 4H, (CH_2_)_2_N), 3.85 (s, 4H, (CH_2_)_2_O), 8.09 (d, 4H, *J* = 6.00 Hz, 2,6-H_2_, 2XAr-H), 8.27 (d, 4H, *J* = 6.00 Hz, 3,5-H_2_, 2XAr-H), 10.21 (s, 2H, 2XNH). ^13^C NMR (125 MHz, DMSO) δ (ppm): 13.56, 43.43, 66.09, 123.52, 126.95, 144.87, 147.02, 162.32, 164.71, 165.16 EI-MS: *m*/*z*: 520.95 [M^+^]. Anal. Calcd. For C_23_H_24_N_10_O_5_: C, 53.07; H, 4.65; N, 26.91. Found: C, 53.23; H, 4.46; N, 26.81.

##### (3E)-5-Chloro-3-[2–(4-{2-[(3E)-5-chloro-2-oxo-2,3-dihydro-1H-indol-3-ylidene]hydrazin-1-yl}6-(morpholin-4-yl)-1,3,5-triazin-2-yl)hydrazin-1-ylidene]-2,3-dihydro-1H-indol-2-one (8b)

Yield (0.33 g, 45.90%) as a dark yellow powder crystallised from DMF with Mp: >300 °C. ^1^H NMR (400 MHz, DMSO-d_6_) δ (ppm): 3.68 (s, 4H, (CH_2_)_2_N), 3.85 (s, 4H, (CH_2_)_2_O), 6.88 (d, 1H, *J* = 8.00 Hz, 6-H, Ar-H), 6.95 (d, 1H, *J* = 9.00 Hz, 6′-H, Ar-H), 7.37 (d, 1H, *J* = 8.00 Hz, 7-H, Ar-H), 7.39 (d, 1H, *J* = 9.00 Hz, 7′-H, Ar-H), 7.51 (s, 1H, 4-H, Ar-H), 8.16 (s,1H, 4′-H, Ar-H), 10.88 (s, 1H, NH-isatin), 11.25 (*br.* s, 1H, NH). 11.37 (s, 1H, NH-isatin’), 12.86 (s, 1H, NH’). ^13^C NMR (125 MHz, DMSO) δ (ppm): 43.59, 65.99, 111.63, 112.43, 116.80, 119.78, 121.89, 125.52, 126.61, 130.03, 131.21, 132.72, 132.86, 135.76, 140.20, 141.90, 162.81, 162.87, 164.17, 164.71, 165.89. EI-MS: *m*/*z*: 552.99 [M^+^]. Anal. Calcd. For C_23_H_18_Cl_2_N_10_O_3_: C, 49.92; H, 3.28; N, 25.31. Found: C, 49.81; H, 3.49; N, 25.52.

##### Methyl 2-({4-[(1-methoxy-1-oxo-3-phenylpropan-2-yl)amino]-6-(morpholin-4-yl)-1,3,5-triazin-2-yl}amino)-3-phenylpropanoate (8c)

Following general procedure for preparation of **4a**,**b**, **3a** (1.30 mmol) was reacted with L-phenylalanine methyl ester hydrochloride (2.60 mmol) and aqueous solution of sodium carbonate (4 mmol, 0.40 M) The reaction mixture was heated under reflux for additional 96 h. Yield (0.15 g, 21.70%) as a white powder with Mp: 142–144 °C. ^1^H NMR (400 MHz, DMSO-d_6_) δ (ppm): 2.98–3.10 (m, 4H, 2XCH_2_-Ph), 3.57–3.65 (m, 14H, 2XCH_3_O, (CH_2_)_2_N, and (CH_2_)_2_O), 4.38–4.59 (m, 2H, 2XCH), 7.10–7.42 (m, 10H, 2XAr-H), 8.49 (d, 1H, *J* = 7.2 Hz, NH). ^13^C NMR (100 MHz, DMSO) δ (ppm): 36.66, 36.75, 43.90, 52.27, 52.55, 55.72, 56.42, 66.04, 66.23, 127.07, 128.73, 128.77, 129.51, 129.59, 137.71, 137.92, 164.00, 165.33, 165.89, 168.86, 169.03, 172.65, 172.72. EI-MS: *m*/*z*: 520.84 [M^+^]. Anal. Calcd. For C_27_H_32_N_6_O_5_: C, 62.29; H, 6.2; N, 16.14. Found: C, 62.38; H, 6.5; N, 16.36.

##### N'-[4-(morpholin-4-yl)-6-(phenylhydrazido)-1,3,5-triazin-2-yl]benzohydrazide (8d)

Following general procedure for preparation of **4a**,**b**, **3a** (1.30 mmol) was reacted with benzohydrazide (2.60 mmol). The reaction mixture was heated under reflux for 6 h. Yield (0.45 g, 78.10%) as a white powder with Mp: 245–246 °C. ^1^H NMR (400 MHz, DMSO-d_6_) δ (ppm): 3.56 (s, 8H, (CH_2_)_2_N, (CH_2_)_2_O), 7.27–7.97 (m, 10H, 2XAr-H), 8.89 (s, 2H, 2XNH), 10.26 (br. s, 2H, 2XNHCO). ^13^C NMR (125 MHz, DMSO) δ (ppm): 43.12, 65.97, 127.37, 127.61, 128.12, 128.49, 131.15, 131.59, 132.99, 133.31, 164.73, 165.80, 166.31, 167.74, 168.18. EI-MS: *m*/*z*: 434.38 [M^+^]. Anal. Calcd. For C_21_H_22_N_8_O_3_: C, 58.06; H, 5.1; N, 25.79. Found: C, 58.19; H, 5.31; N, 25.97.

### Biological evaluation

#### Procedure for in vitro antitumor screening in national cancer institute “NCI”

The antitumor screening was performed for 60 human cancer cell lines under the protocol of NCI (www.dtp.nci.nih.gov), the Drug Evaluation Branch, Bethesda, MD. All submitted compounds were nominated based on the degree of structural diversity and computer modelling techniques for evaluation of their anticancer activity. The screening process for antineoplastic activity passes through two successive levels, starting with the assessment of target compounds at a single dose of 10 μM against 60 cancer cell lines on nine types of human tumours. These experiments were done on a triplicate following^54^. The output from the single‐dose screening was covered as a mean graph, and then, analysed by the COMPARE program. Finally, the compounds which disclosed subpanel cancer cell growth (G %) equal to or less than 10% in at least eight cancer cell lines get to pass the assessment against the 60 cell lines at five doses (0.01–100 μM). By using GraphPad Prism version 9.0 (Graphpad Software Inc., La Jolla, CA), GI_50_, TGI, as well as LC_50_ dose‐response parameters were computed and presented for each compound (Supplementary information)^55–57^.

#### Assay of IDH2 enzyme inhibition

Assay of IDH2^R140Q^ enzyme inhibition was performed using the fluorimetry-based assay according to manufacturer’s procedure of BPS Biosciences IDH2 (R140Q) Assay Kit, catalogue #79309 and wild type IDH Assay Kit, catalogue #71074.

#### In vitro cytotoxicity against human normal cells (human embryonic kidney)

Assay of HEK-293 cell GI was performed as previously described by Tim Mosmann^58^. Data was measured in triplicate, and IC_50_ values are given as mean values ± SD.

#### Assay of Annexin V-FITC apoptosis

Annexin V-fluorescein isothiocyanate (Annexin V-FITC) has high affinity to phosphatidylserine (PS). This affinity enables detection of PS by Annexin V-FITC staining as previously described^59^.

#### Cell cycle analysis

HL-60(TB) cells were treated for 24 h with the GI_50_ concentration of compound **6c**. Following that, two runs of wash with ice-cold phosphate-buffered saline (PBS) were performed. And then, the treated HL-60(TB) cells were centrifuged and frozen in ethanol (70%, ice-cold) then washed in PBS, resuspended with RNase (100 mg/mL), stained with PI (40 mg/mL), finally analysed by flow cytometry using a FACS Calibur (Becton Dickinson, BD; Franklin Lakes, NJ). To calculate the cell cycle distributions, CellQuest software version 5.1 (Becton Dickinson) was used.

#### Western blot analysis

Compound **6c** was incubated with the seeded cells following the procedure described by W. Neal Burnette^60^.

## *In silico* studies

### Molecular docking study

The crystal structure of IDH2 was downloaded as a PDB file from the research Collaboratory for structural bioinformatics (RCSB) Protein Data Bank (PDB ID: 5I96). The PDB file of the protein was opened in MOE version 2020.09 and all non-standard atoms and bonds were removed. Then the protein was prepared using the preparation options available in the program as well as energy minimised. Data base of the selected inhibitors was prepared by MOE. Docking was done and the results were visualised by Discovery Studio version 20.1.019295.

## *In silico* physicochemical and pharmacokinetic study

We applied SwissADME model which is a free web tool developed by the Swiss Institute of Bioinformatics (SIB) (http://www.swissadme.ch) to predict physicochemical and pharmacokinetic properties of our compounds. A Boiled-Egg plot provides a supportive guidance and a statistical plot to foreshow the two passive prediction of small molecules, i.e. gastrointestinal absorption and brain-permeant. This model supplies a superior optimisation method. Gastrointestinal absorption and Brain access are two crucial pharmacokinetic parameters necessary in estimating the stages of the drug discovery processes. This computational tool also provides parameters; MLOGP, TPSA, and MW. Moreover, SwissADME tool confers a prediction to drug-likeness using the physicochemical properties and by applying number of rules as Lipinski, Ghose, Veber, Egan, and Muegge rules.

## Supplementary Material

Supplemental Material
