# Supplementary material for: Discovery of novel enasidenib analogues targeting inhibition of mutant isocitrate dehydrogenase 2 as antileukaemic agents
Source: J Enzyme Inhib Med Chem. 2023 Jan 11;38(1):2157411. doi: 10.1080/14756366.2022.2157411 (PMC9848300; doi:10.1080/14756366.2022.2157411)
Supplement: Supplemental Material [file IENZ_A_2157411_SM0930.pdf]

Discovery of novel enasidenib analogues targeting inhibition of mutant isocitrate dehydrogenase 2 as antileukemic agents

Ahmed F. Khalil[a*], Tarek F. El-Moselhy[a], Eman A. El-Bastawissy[a], Rasha Abdelhady[b], Nancy S. Younis[c], Mervat H. El-Hamamsy[a]

[a]*Department of Pharmaceutical Chemistry, Faculty of Pharmacy, Tanta University, Tanta, 31527, Egypt*

[b]*Department of Pharmacology and Toxicology, Faculty of Pharmacy, Fayoum University, Fayoum 63514, Egypt*

[c]*Department of Pharmaceutical Sciences, College of Clinical Pharmacy, King Faisal University, Al Hofuf 31982, Al-Ahsa, Saudi Arabia*

Corresponding author: Ahmed F. Khalil, E-mail: ahmed.faisal@pharm.tanta.edu.eg

Phone +201015806836

SUPPORTING INFORMATION

Spectral data section ……………………………..…. P. 2-P.45
Biological section ………………………………..…. P. 46-P.93

Information Classification: General

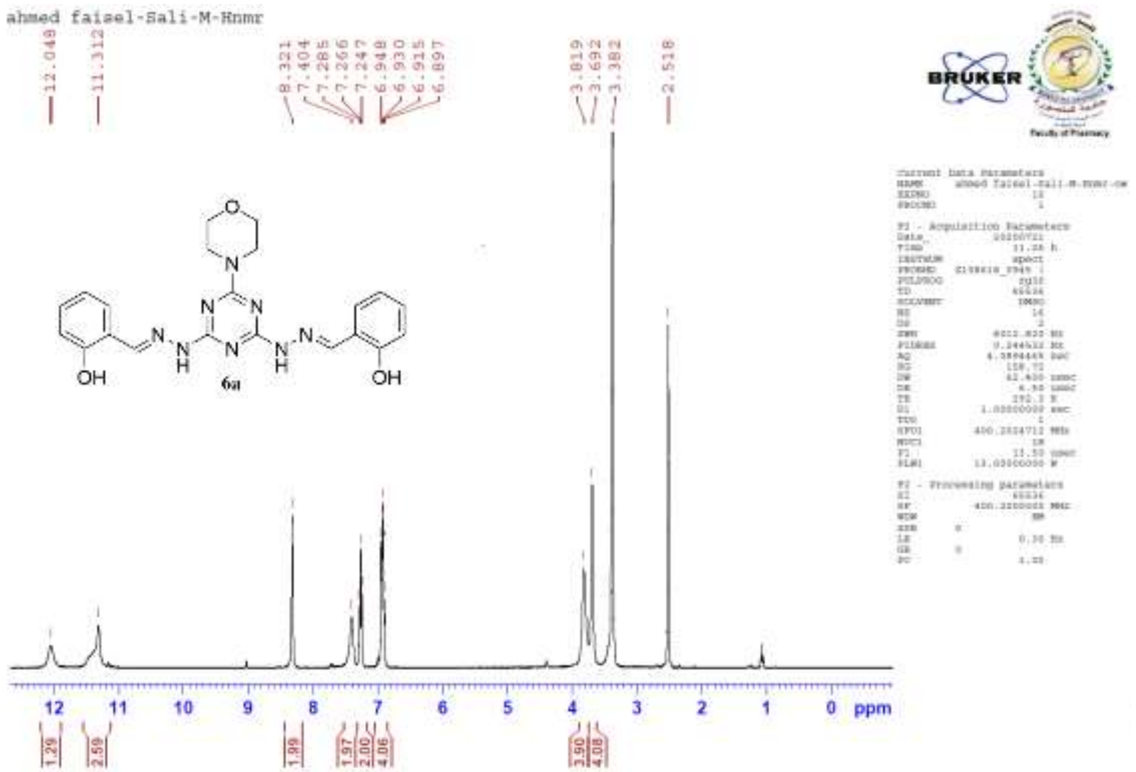

Figure S1. **^{1}H NMR (400 MHz, DMSO-d_6) spectrum of compound 6a**

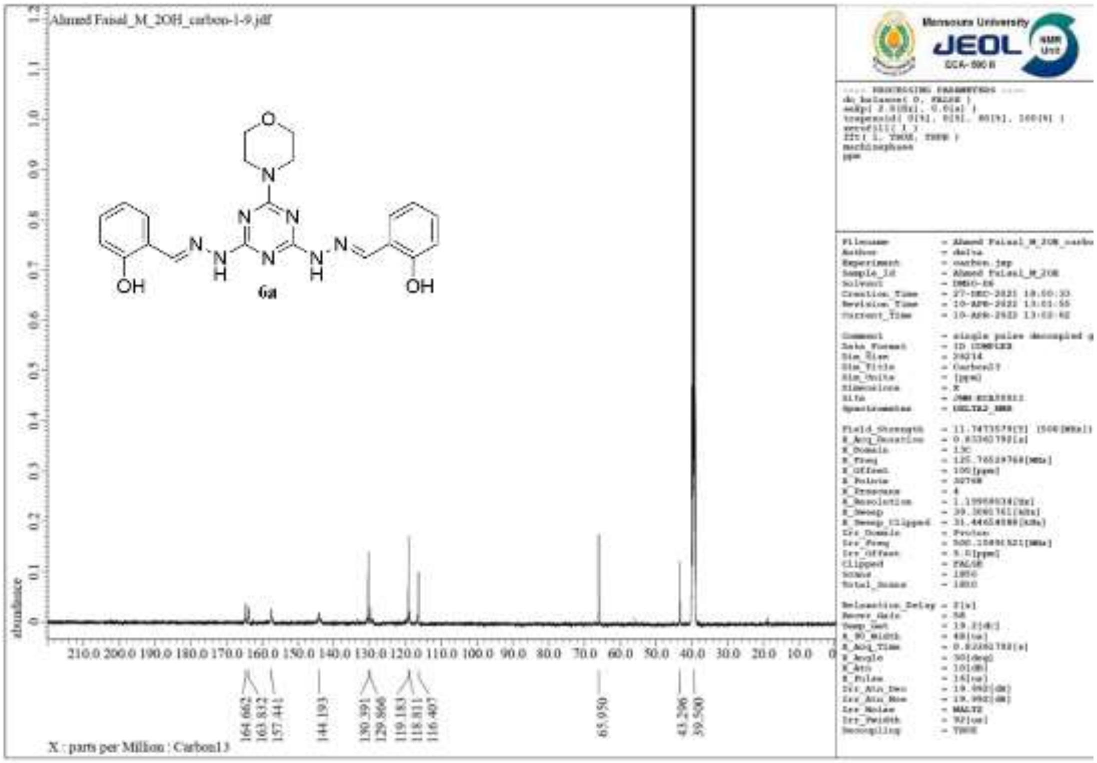

Figure S2. **^{13}C NMR (125 MHz, DMSO) spectrum of compound 6a**

Information Classification: General

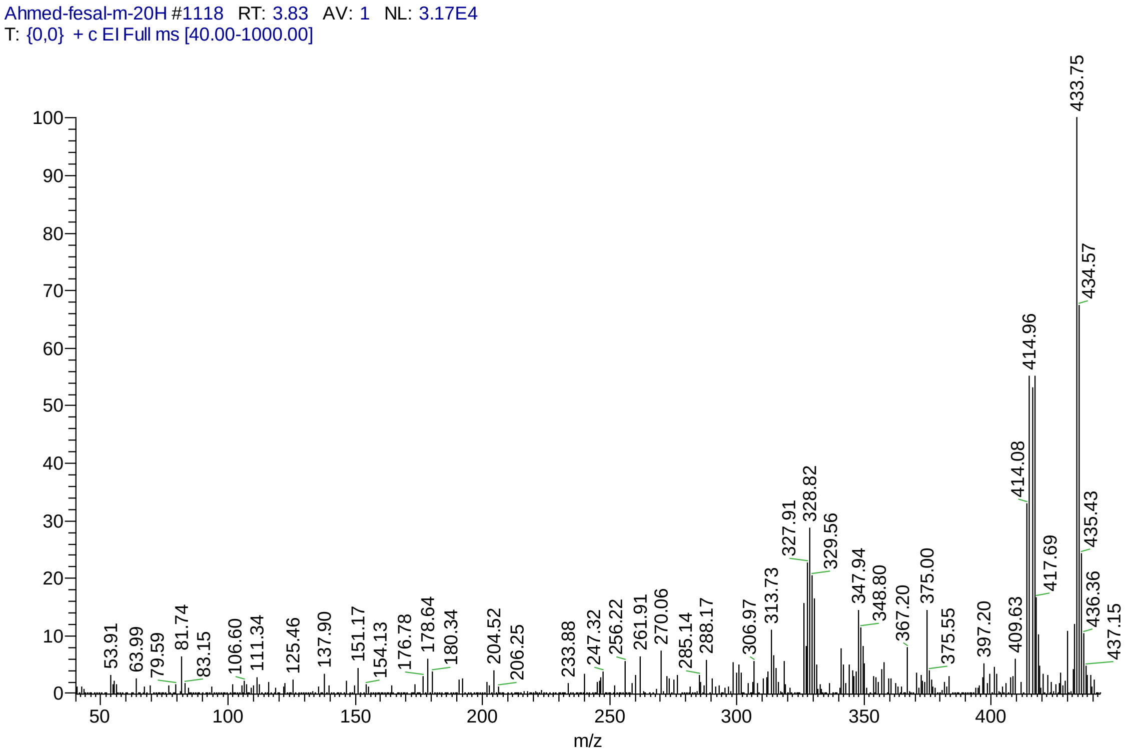

Figure S3. Mass spectrum of compound 6a

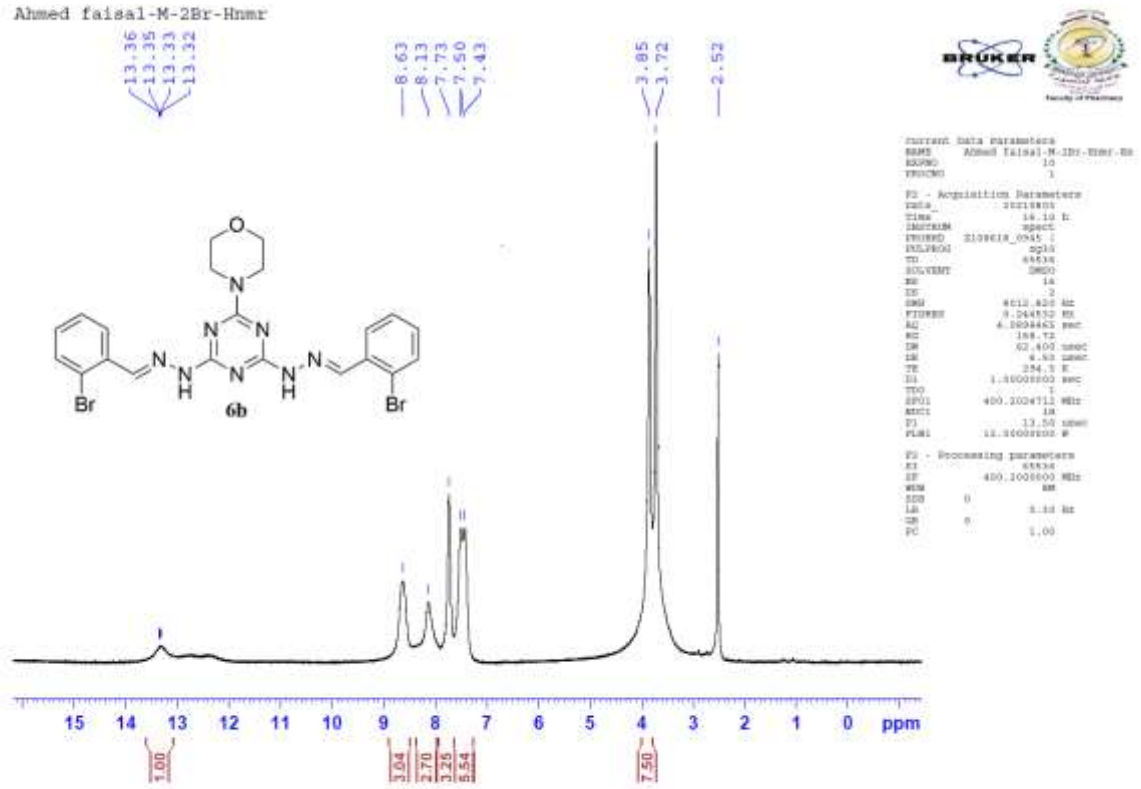

Information Classification: General

Figure S4. ^{1}**H NMR (400 MHz, DMSO-**d_6**) spectrum of compound 6b**

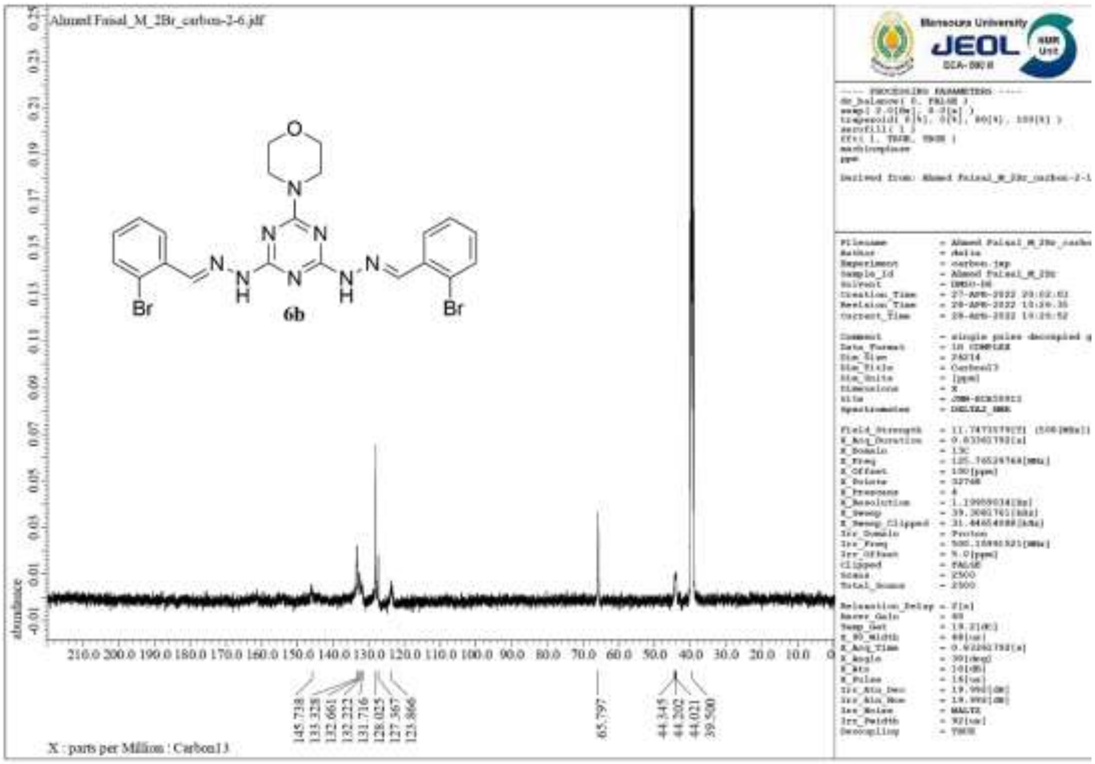

Figure S5. ^{13}**C NMR (125 MHz, DMSO) spectrum of compound 6b**

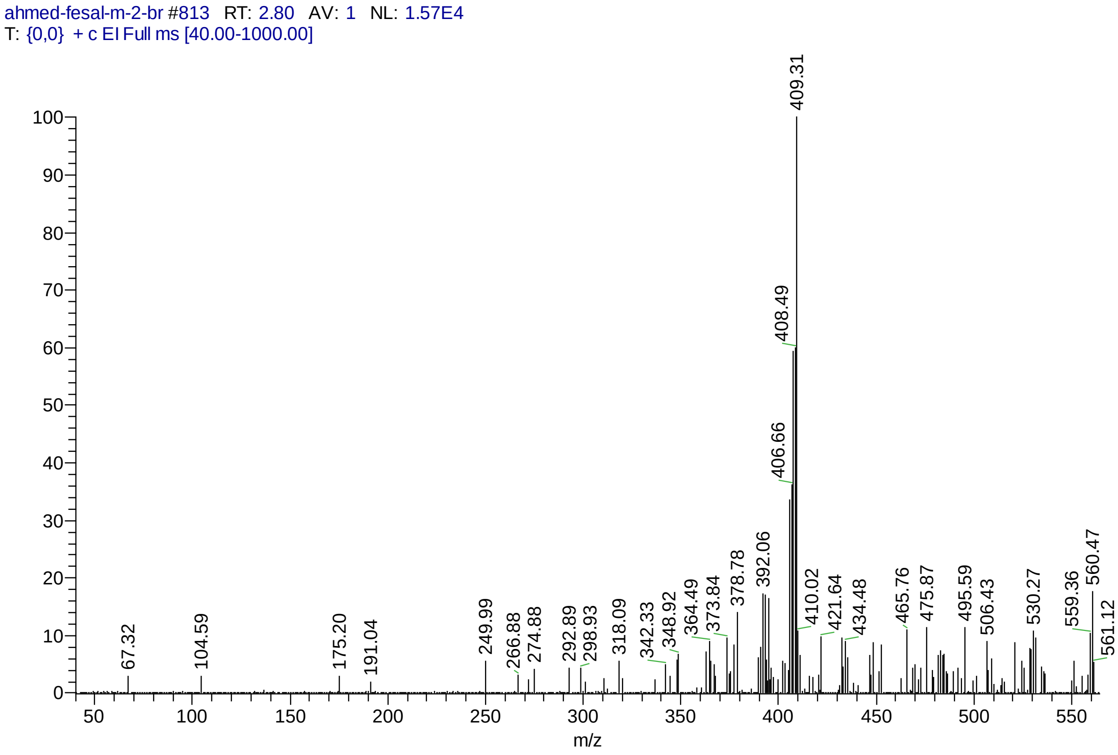

Figure S6. Mass spectrum of compound 6b

Information Classification: General

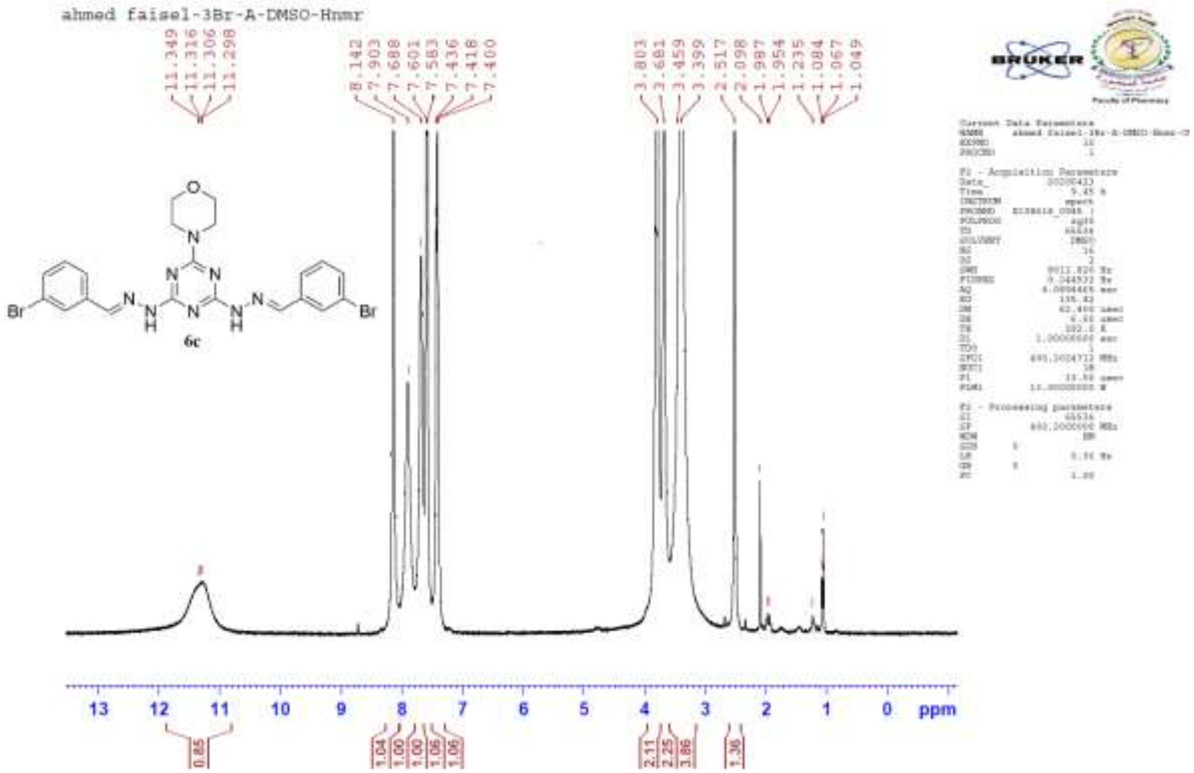

Figure S7. ^{1}H NMR (400 MHz, DMSO-d_6) spectrum of compound 6c

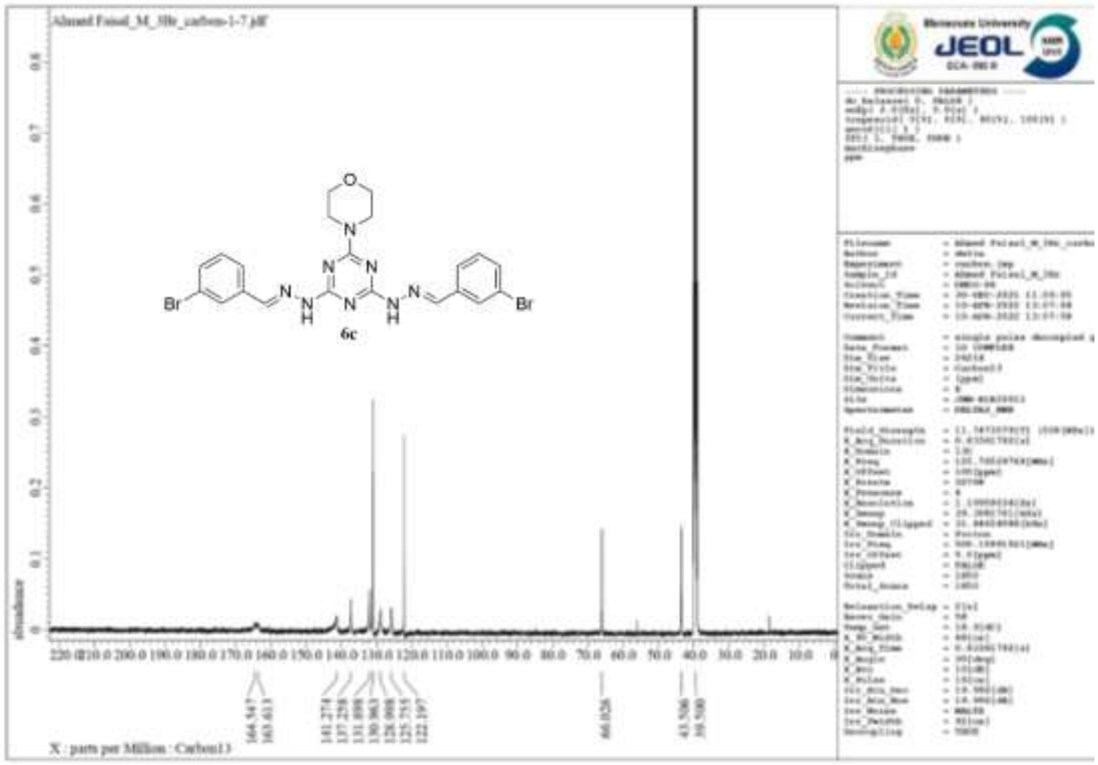

Figure S8. ^{13}C NMR (125 MHz, DMSO) spectrum of compound 6c

Information Classification: General

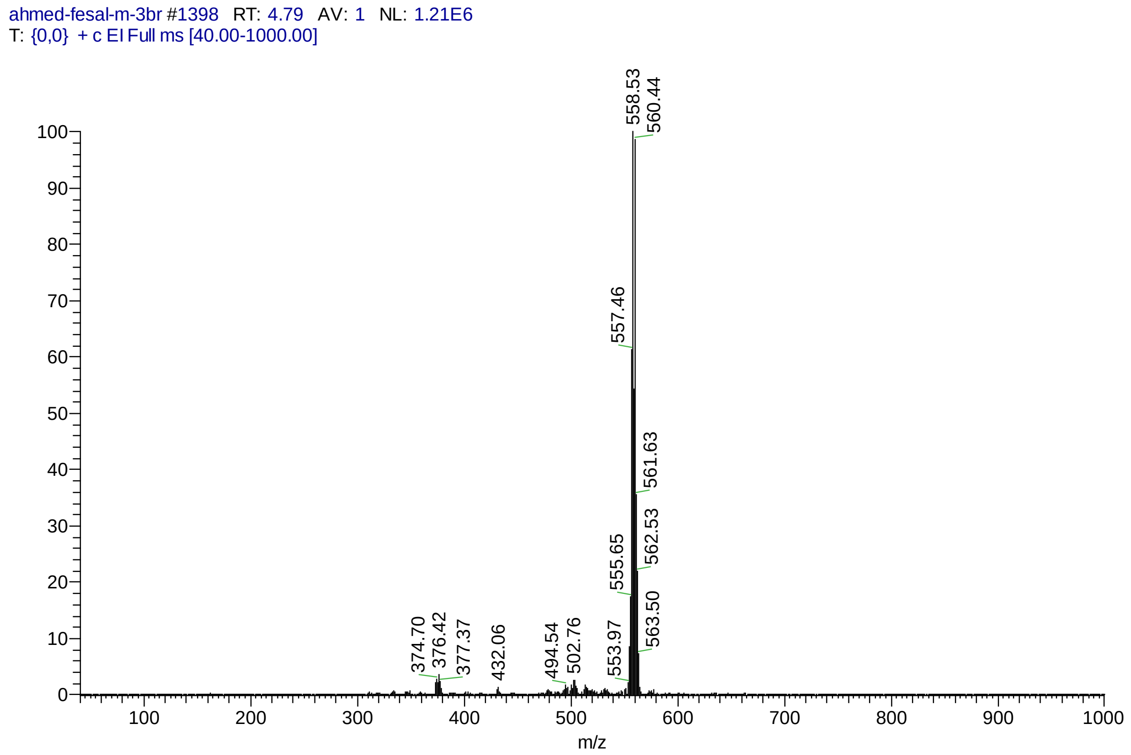

Figure S9. Mass spectrum of compound 6c

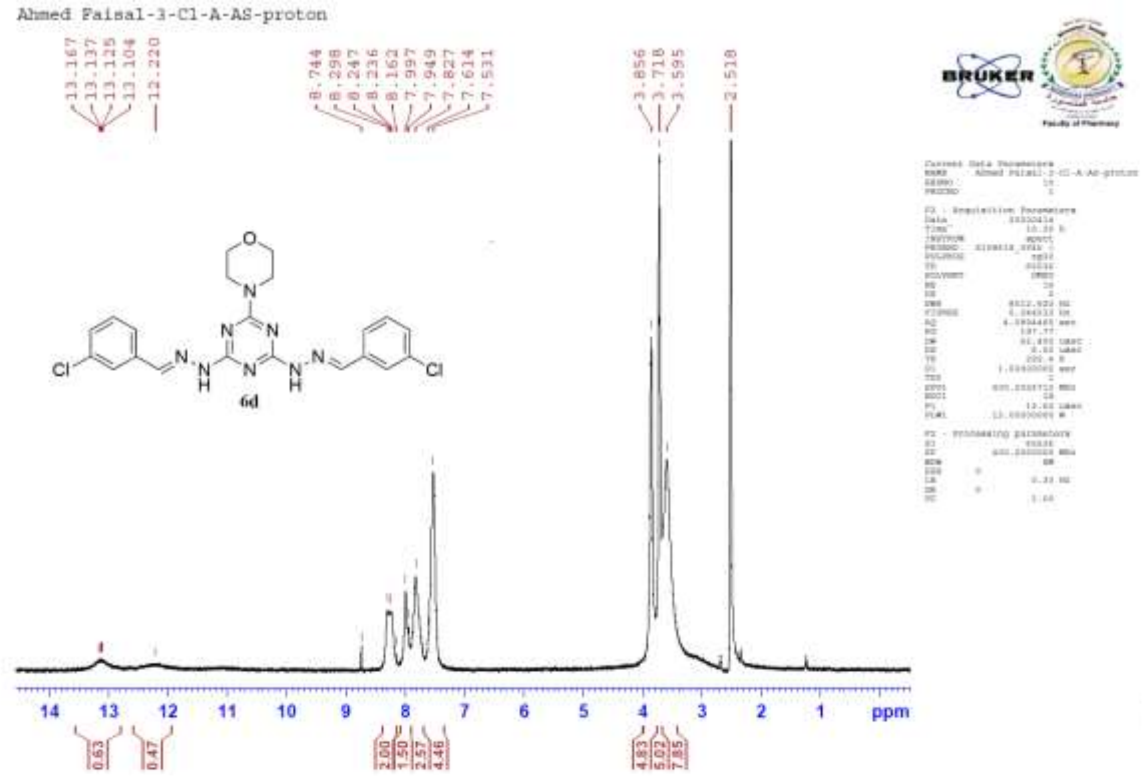

Figure S10. **^{1}H NMR (400 MHz, DMSO-*d_6*) spectrum of compound 6d**

Information Classification: General

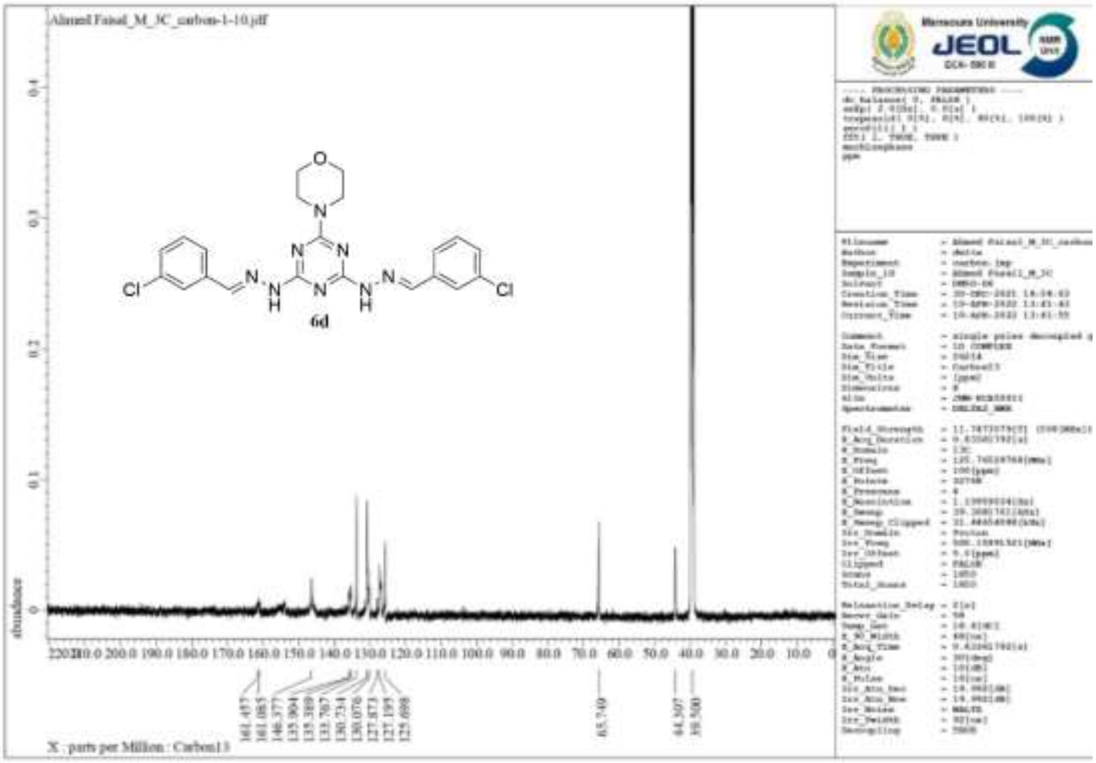

Figure S11. ^{13}**C NMR (125 MHz, DMSO) spectrum of compound 6d**

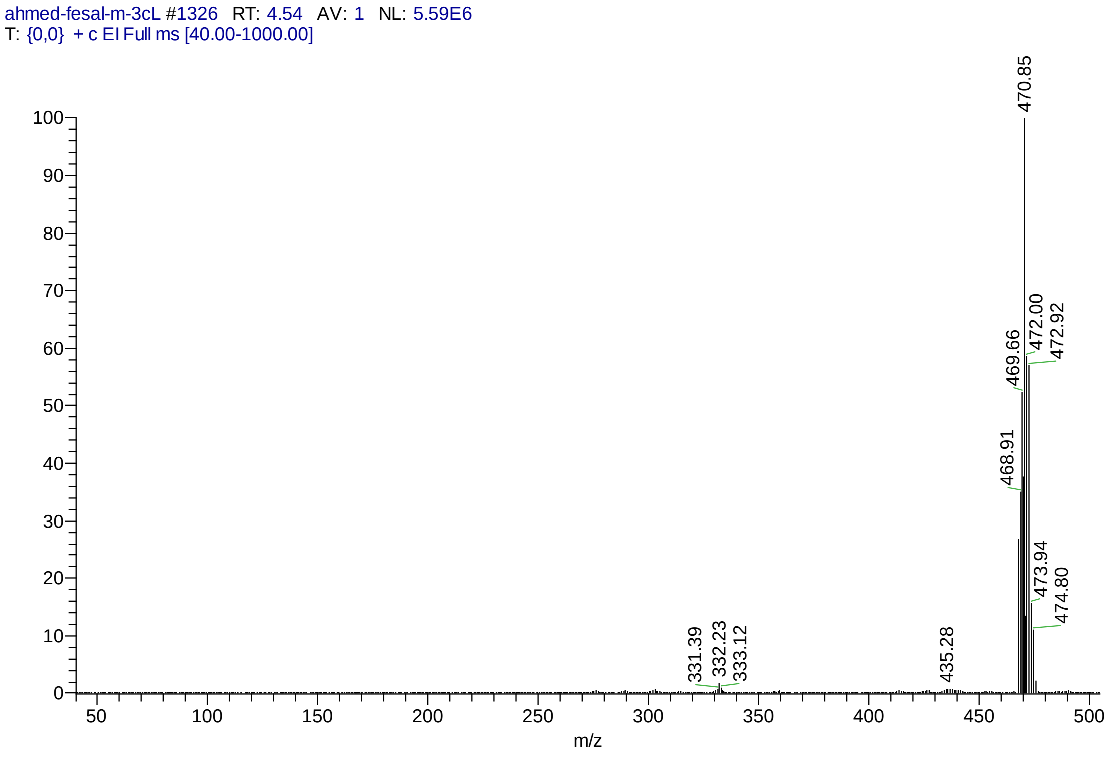

Figure S12. Mass spectrum of compound 6d

Information Classification: General

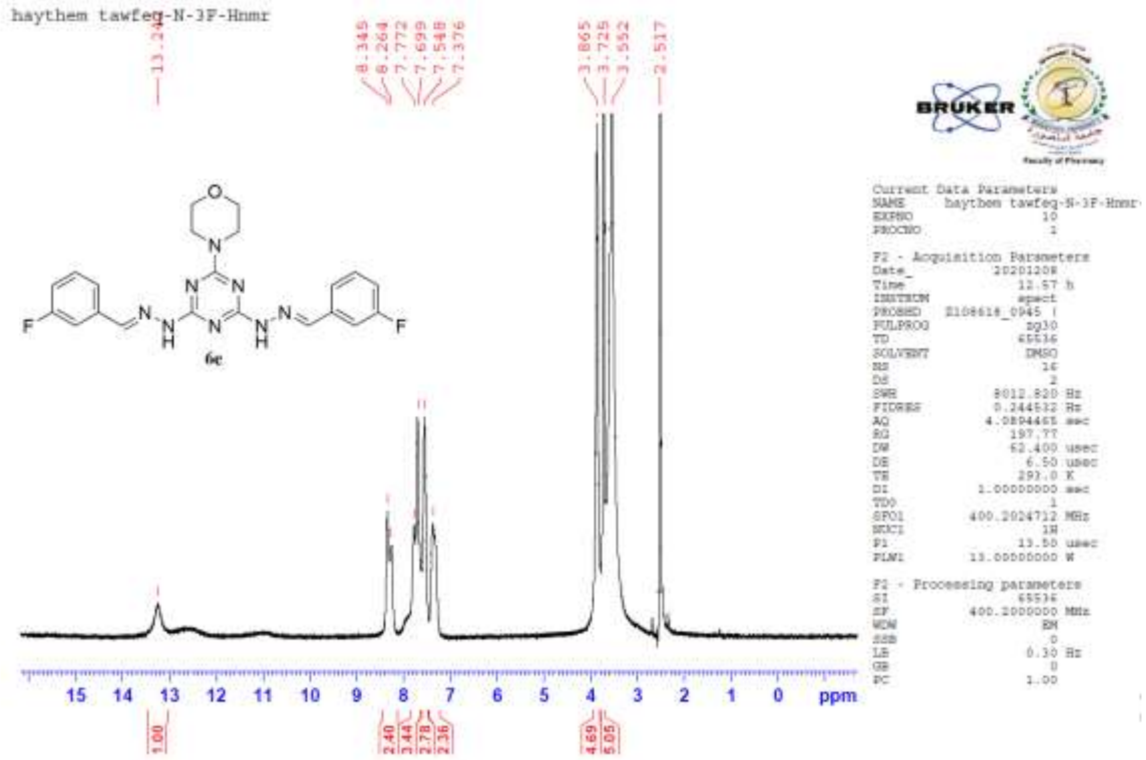

Figure S13. **^{1}H NMR (400 MHz, DMSO-d_6) spectrum of compound 6e**

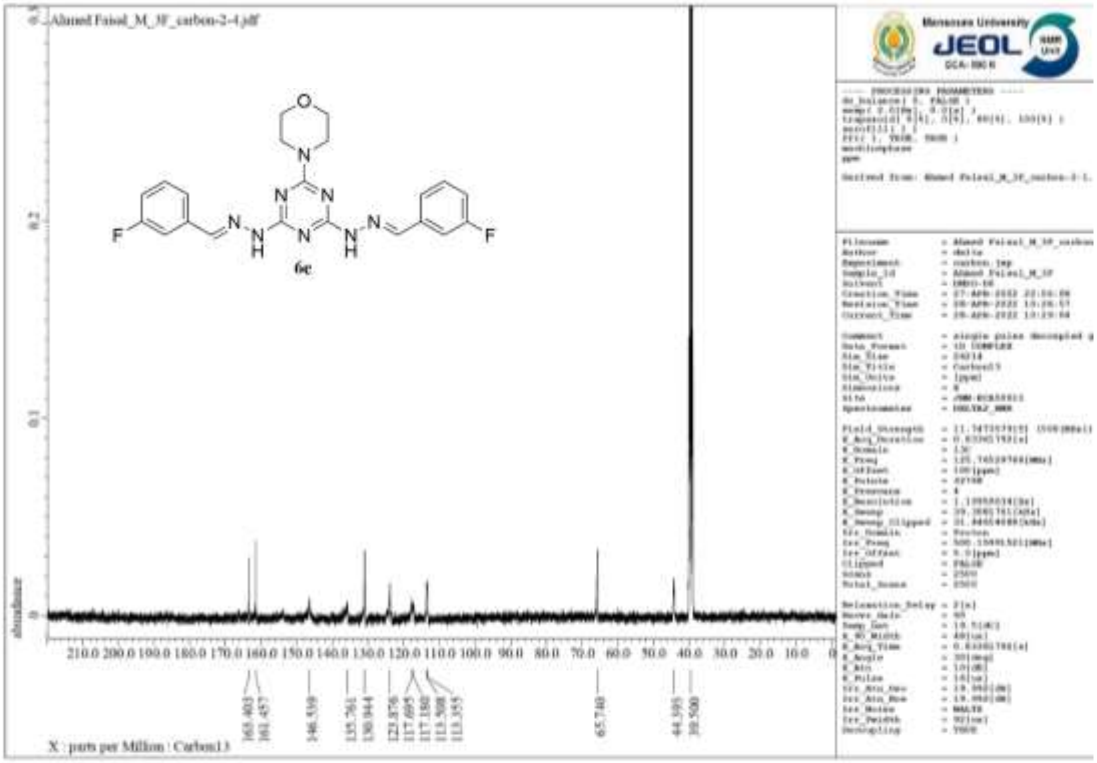

Figure S14. **^{13}C NMR (125 MHz, DMSO) spectrum of compound 6e**

Information Classification: General

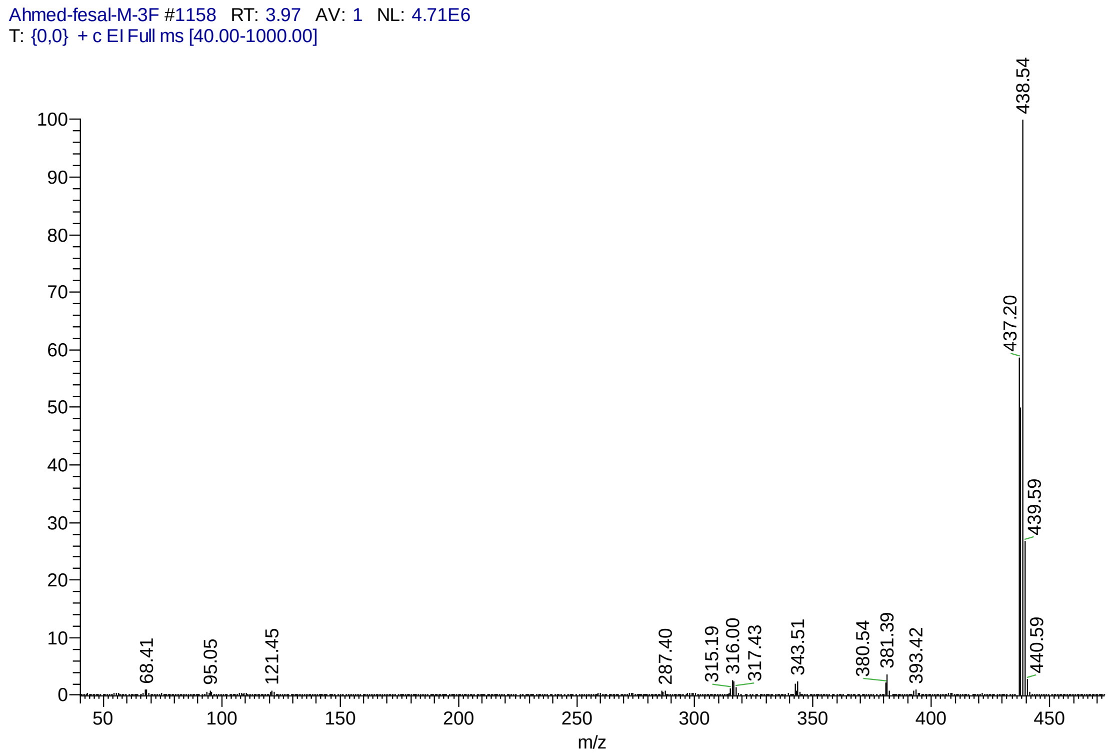

Figure S15. Mass spectrum of compound 6e

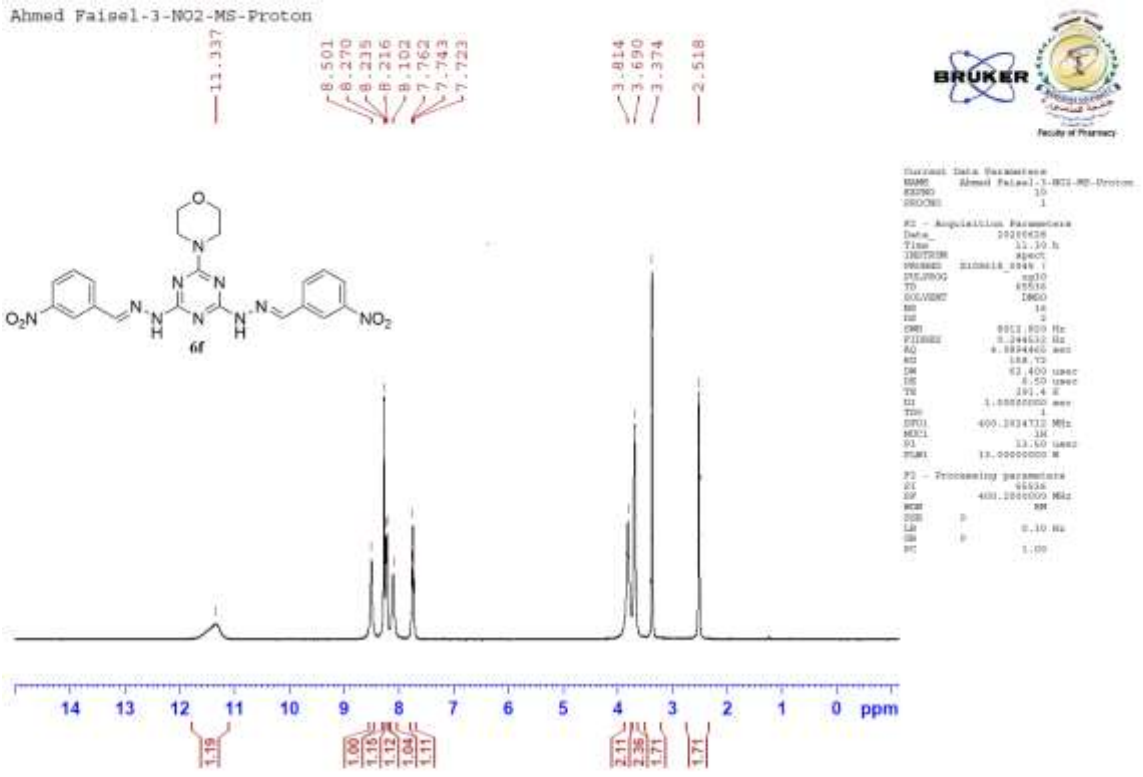

Figure S16. ^{1}H NMR (400 MHz, DMSO-d_6) spectrum of compound 6f

Information Classification: General

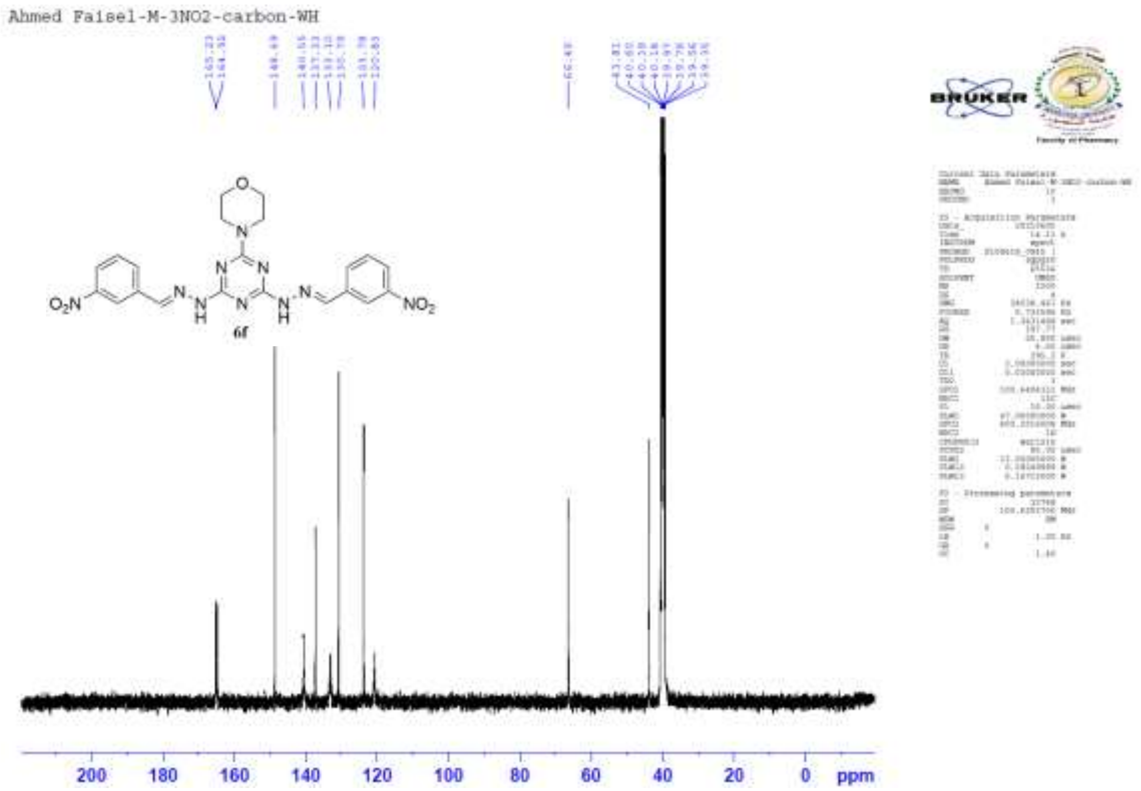

Figure S17. ^{13}**C NMR (125 MHz, DMSO) spectrum of compound 6f**

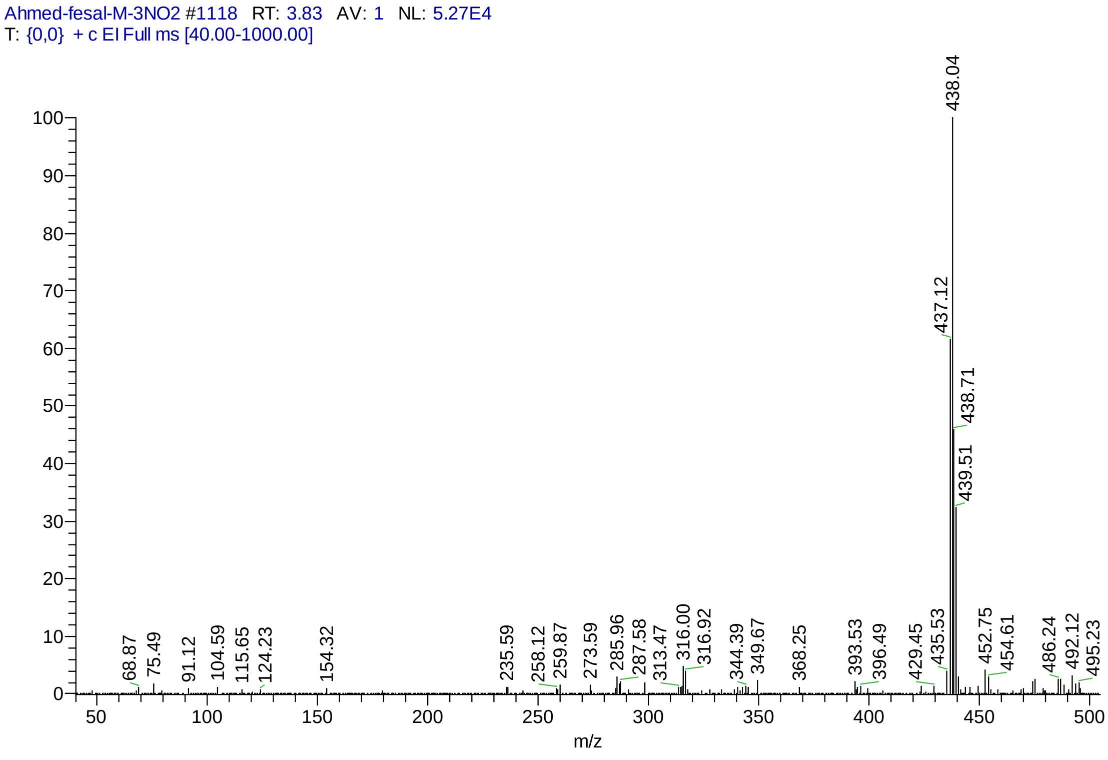

Figure S18. Mass spectrum of compound 6f

Information Classification: General

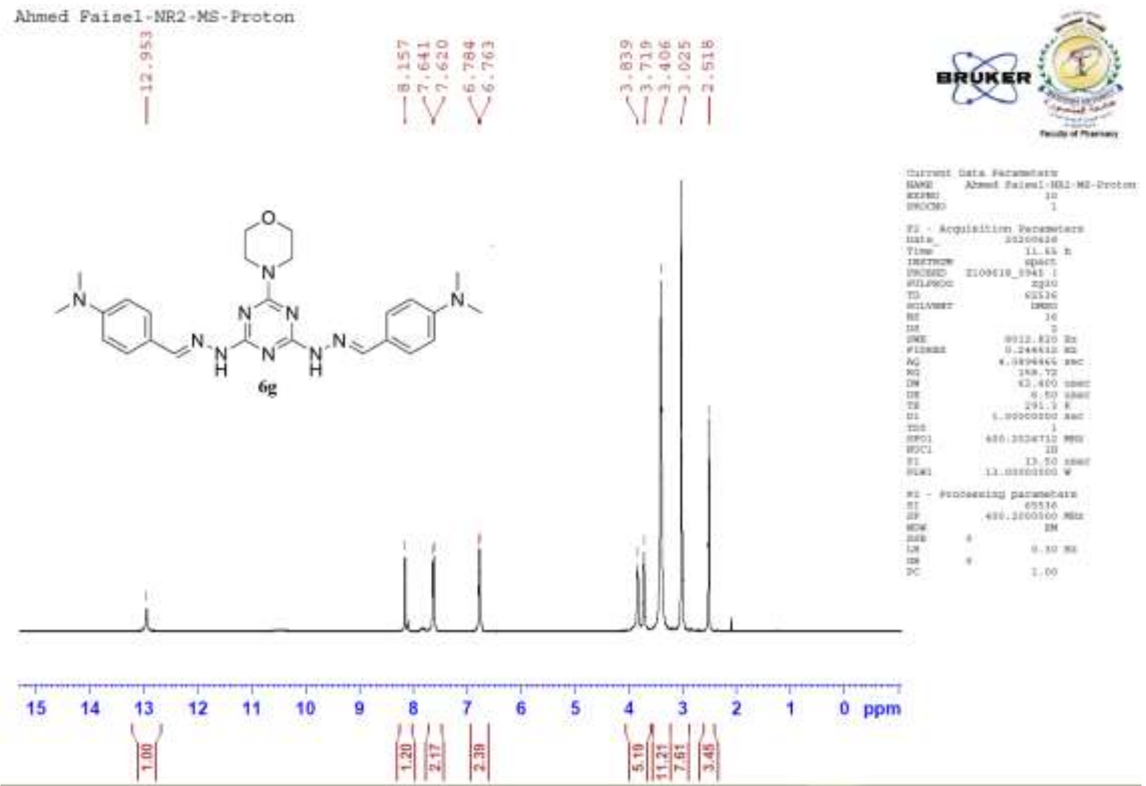

Figure S19. ^{1}H NMR (400 MHz, DMSO-d_6) spectrum of compound 6g

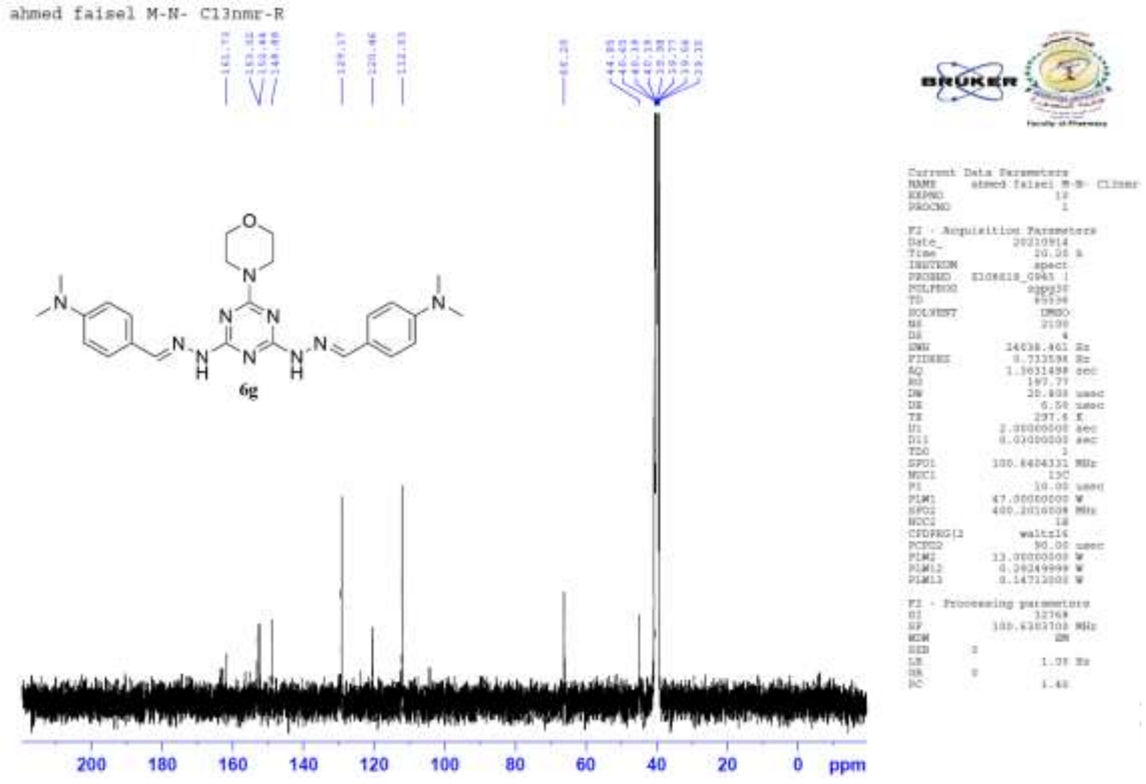

Figure S20. ^{13}C NMR (100 MHz, DMSO) spectrum of compound 6g

Information Classification: General

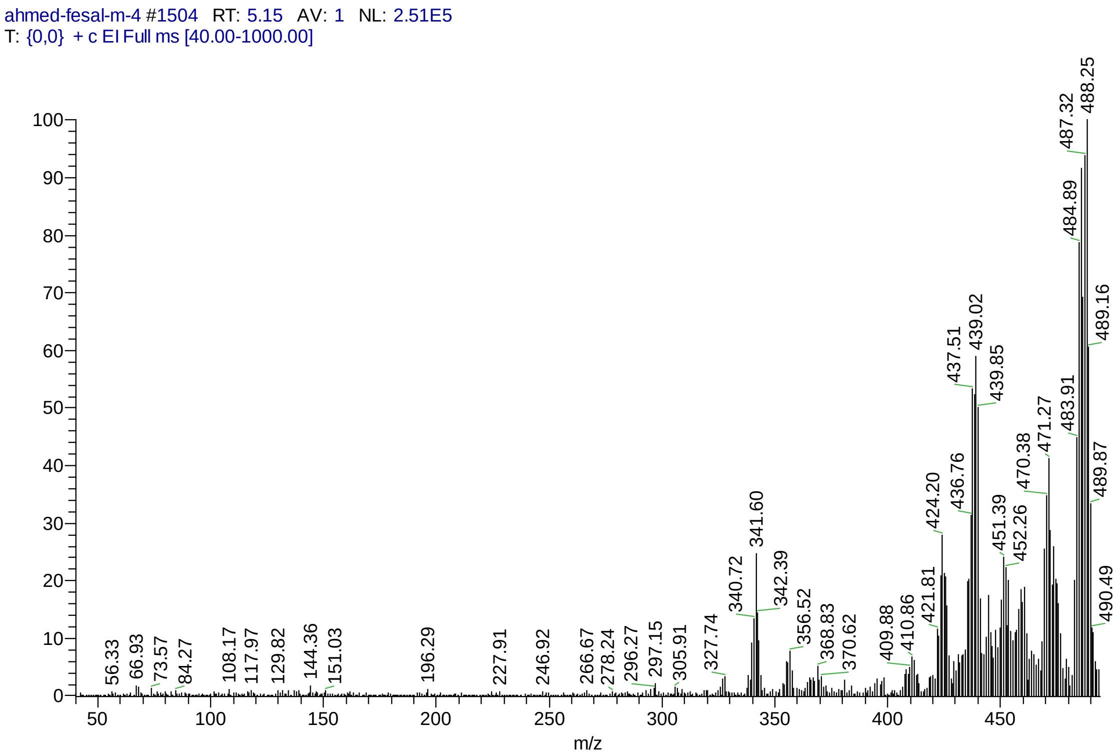

Figure S21. Mass spectrum of compound 6g

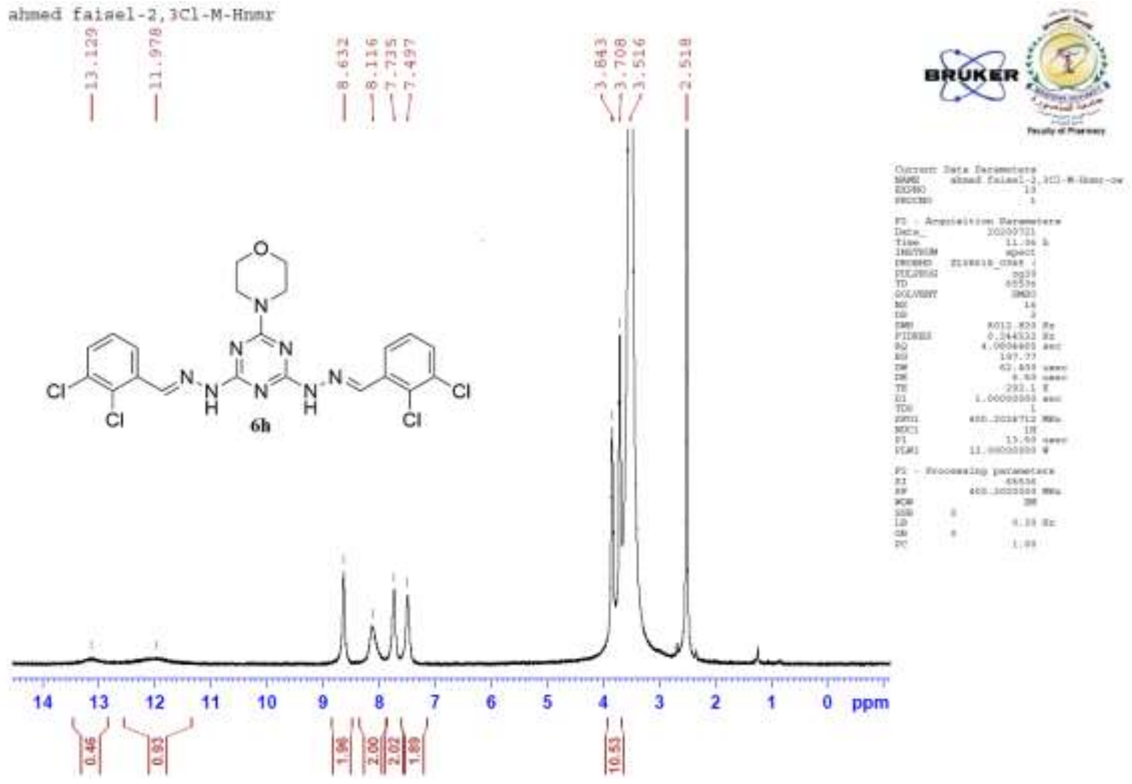

Figure S22. ^{1}H NMR (400 MHz, DMSO-d_6) spectrum of compound 6h

Information Classification: General

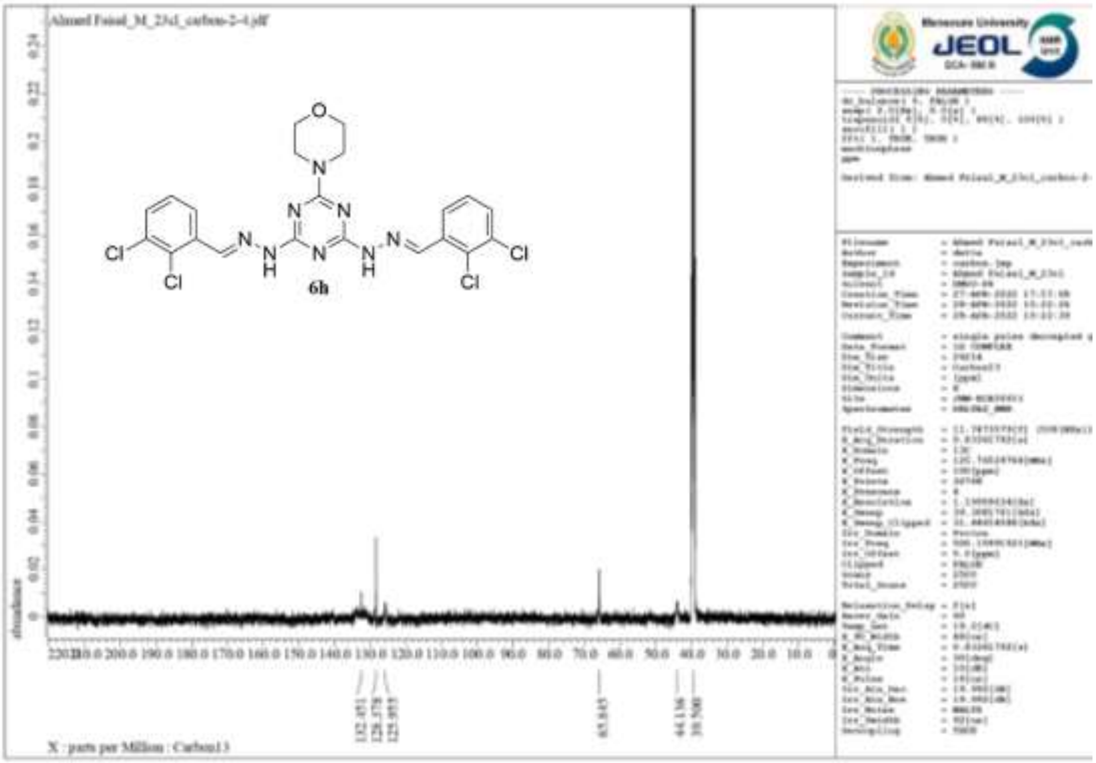

Figure S23. ^{13}C **NMR (125 MHz, DMSO) spectrum of compound 6h**

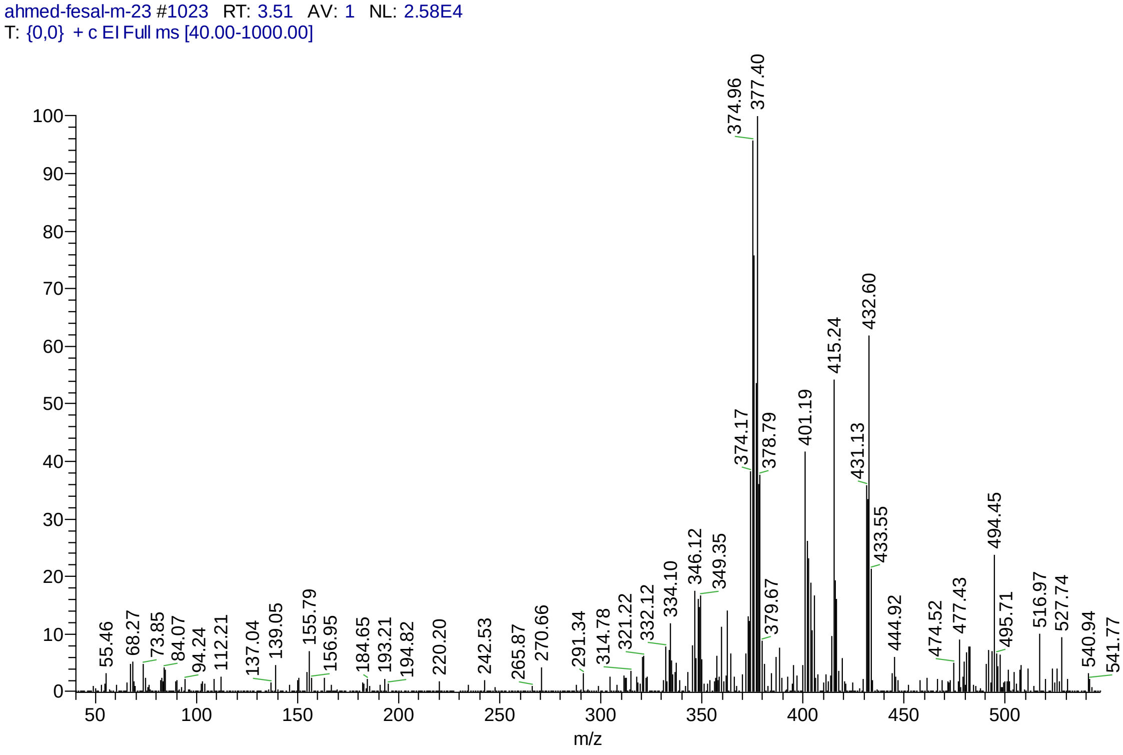

Figure S24. Mass spectrum of compound 6h

Information Classification: General

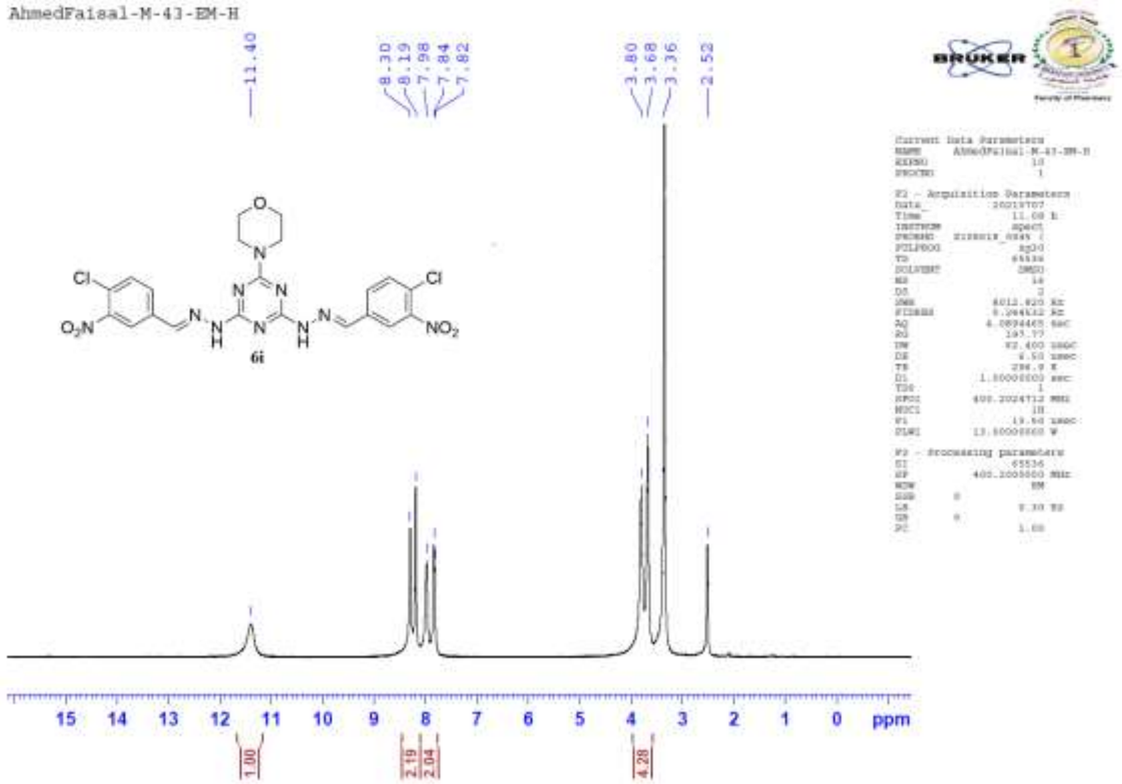

Figure S25. [1]**H NMR (400 MHz, DMSO-*d₆*) spectrum of compound 6i**

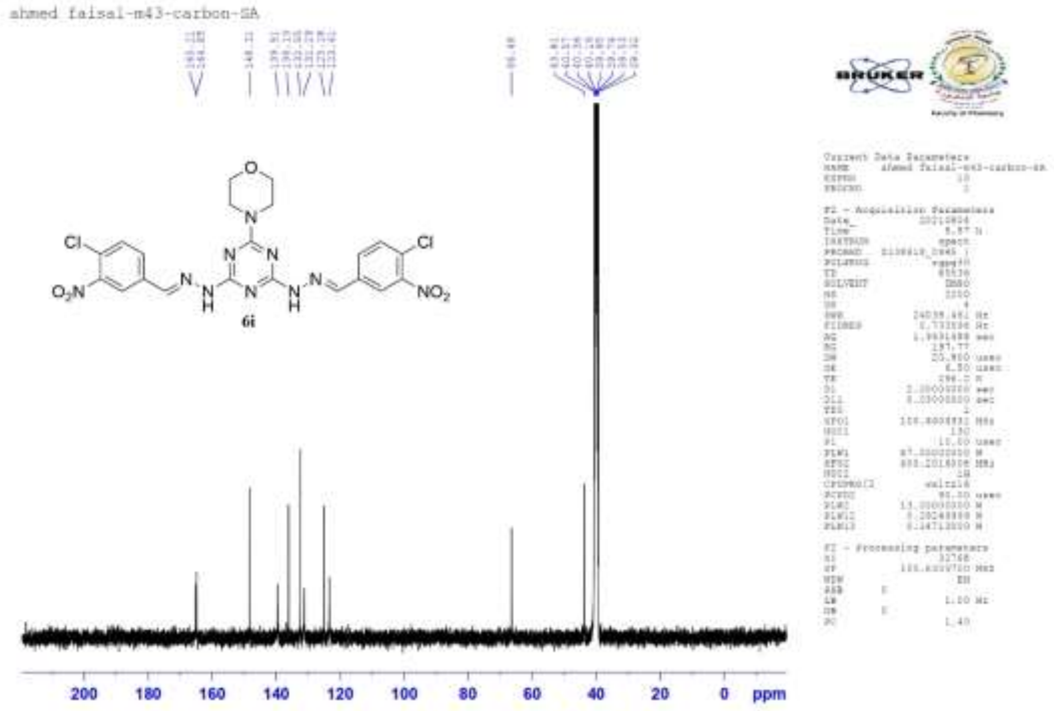

Figure S26. [13]**C NMR (100 MHz, DMSO) spectrum of compound 6i**

Information Classification: General

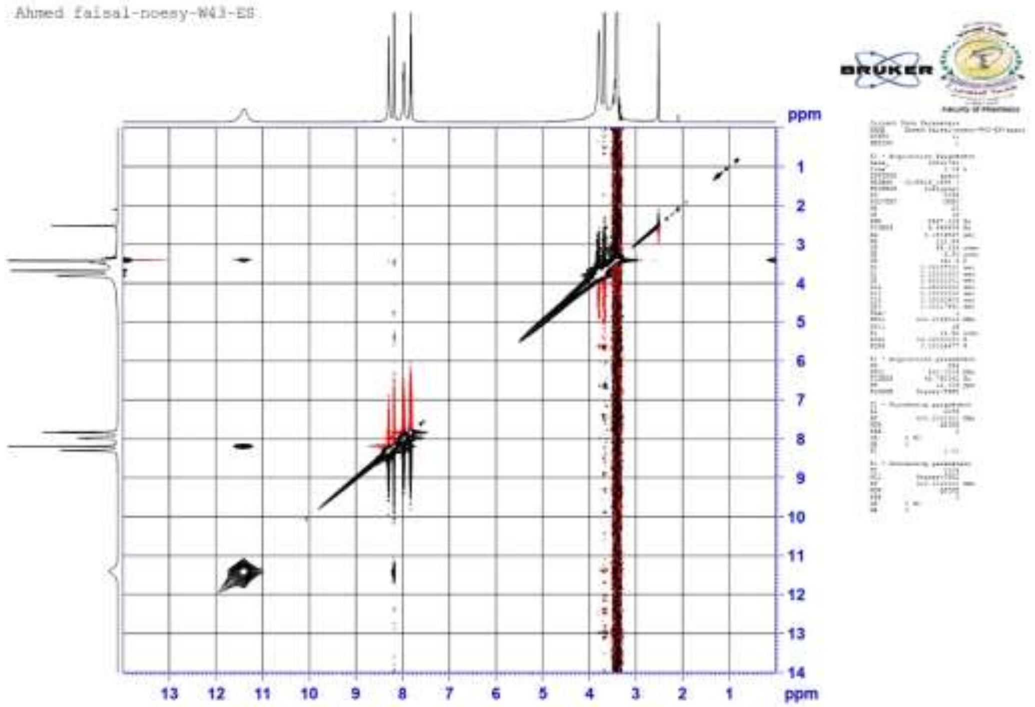

Figure S27. **2D ^{1}H-^{1}H homonuclear NOESY spectrum of compound 6i**

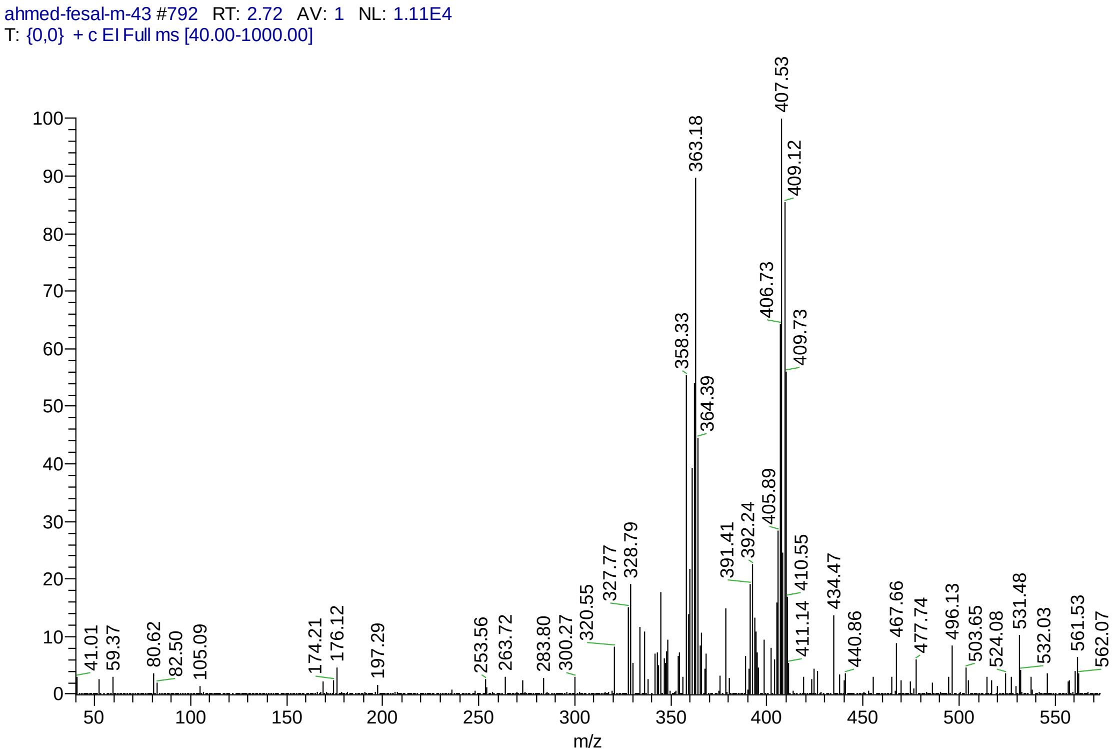

Figure S28. Mass spectrum of compound 6i

Information Classification: General

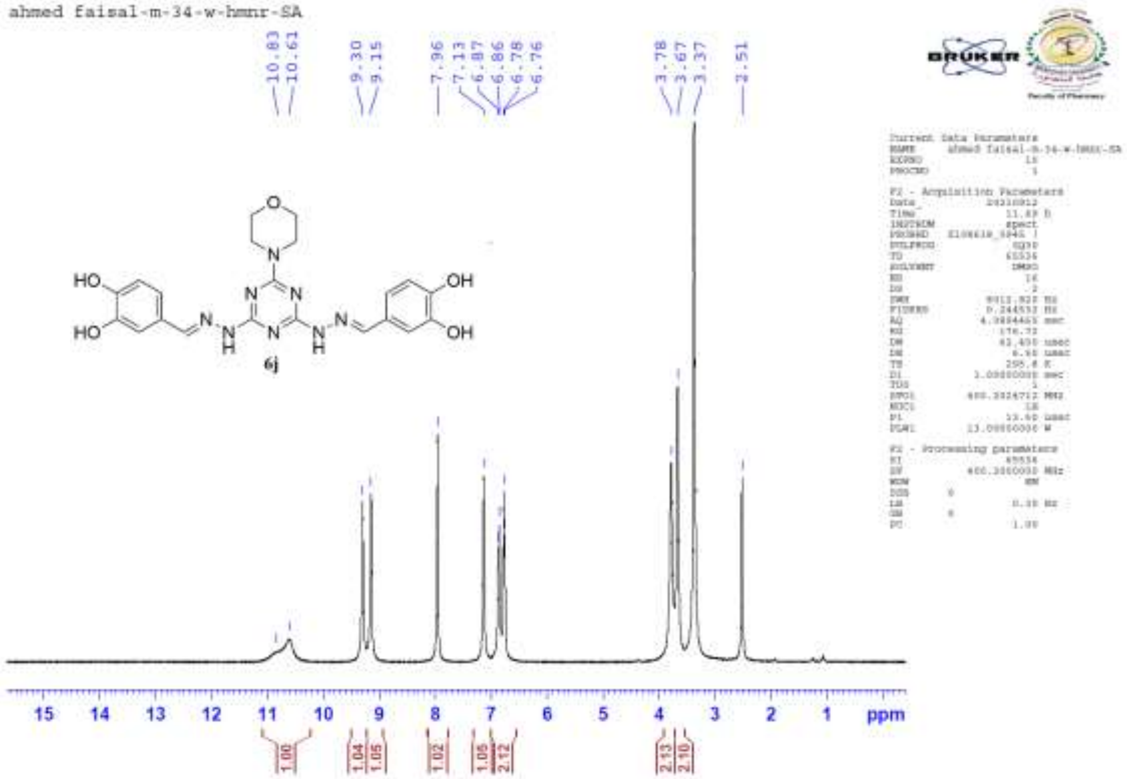

Figure S29. **^{1}H NMR (400 MHz, DMSO-d_6) spectrum of compound 6j**

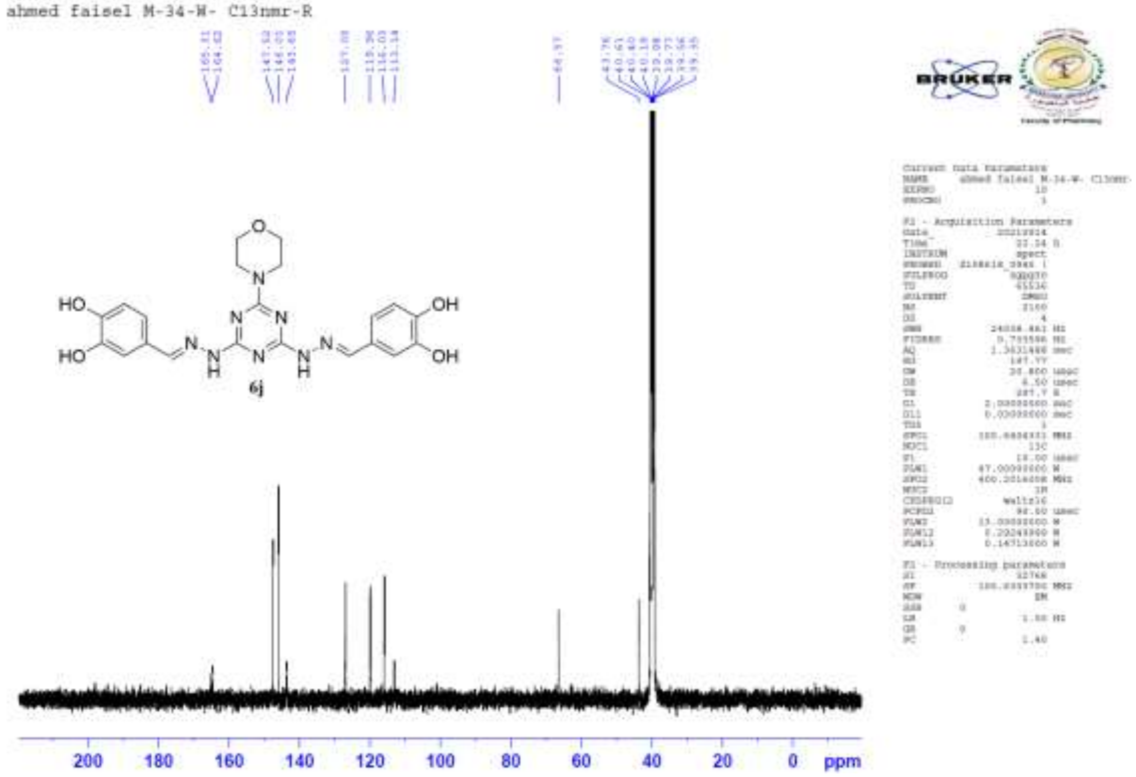

Figure S30. **^{13}C NMR (100 MHz, DMSO) spectrum of compound 6j**

Information Classification: General

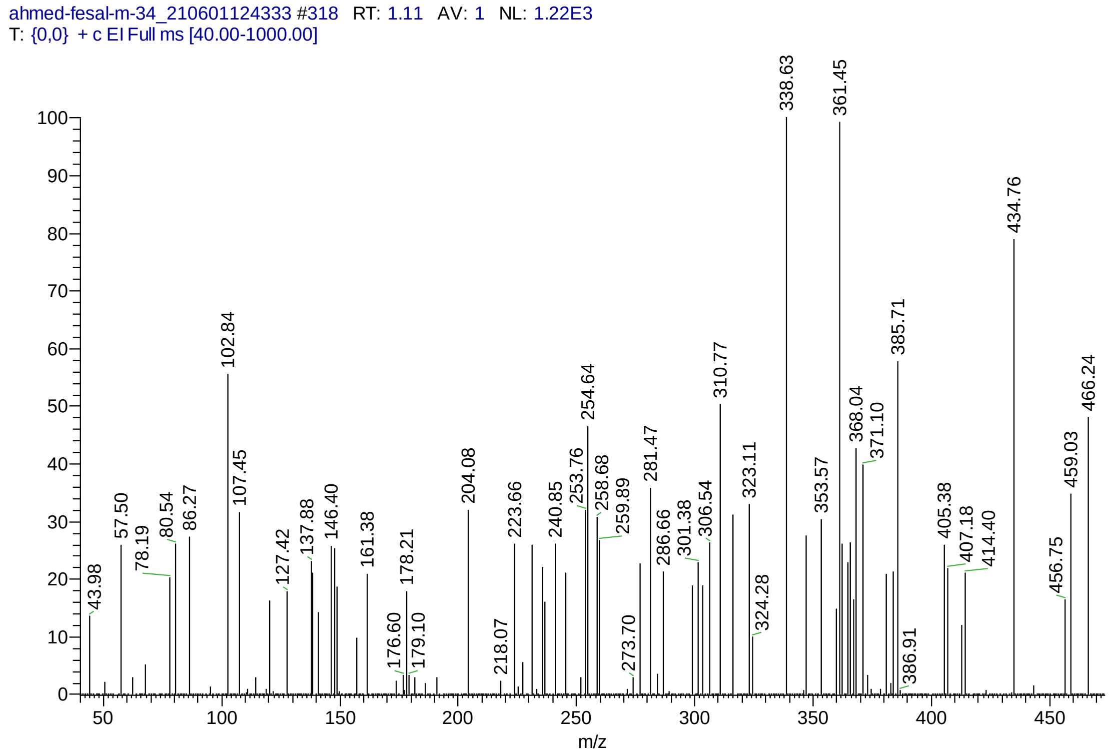

Figure S31. Mass spectrum of compound 6j

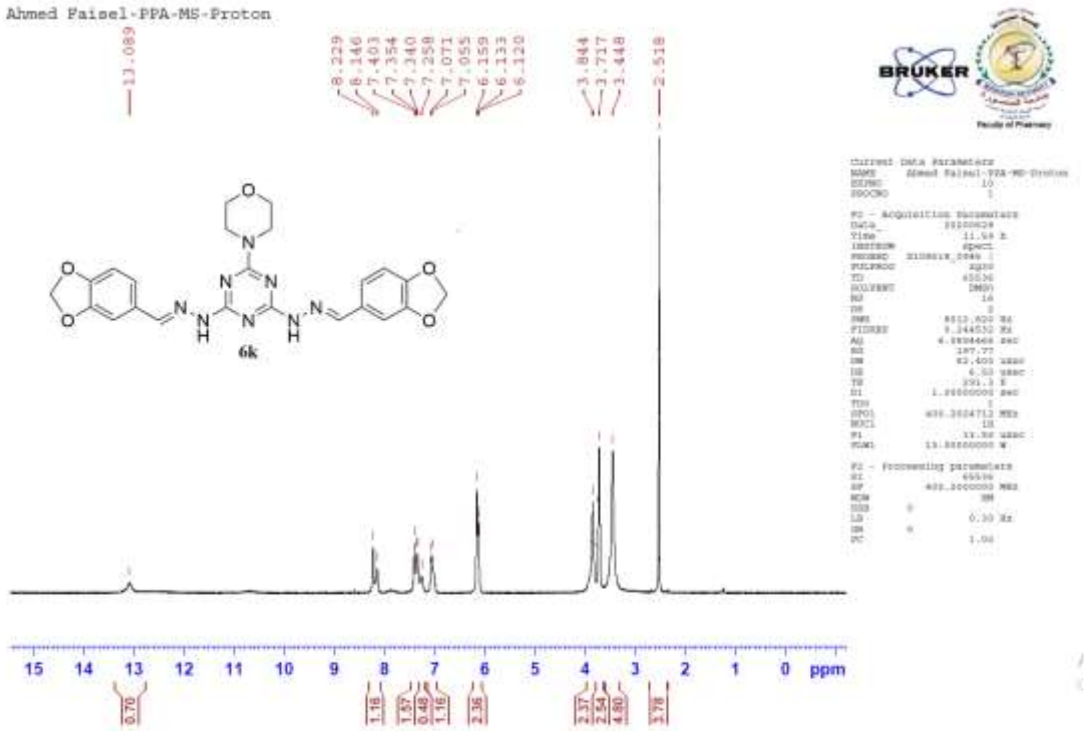

Figure S32. ^{1}H NMR (400 MHz, DMSO-d_6) spectrum of compound 6k

Information Classification: General

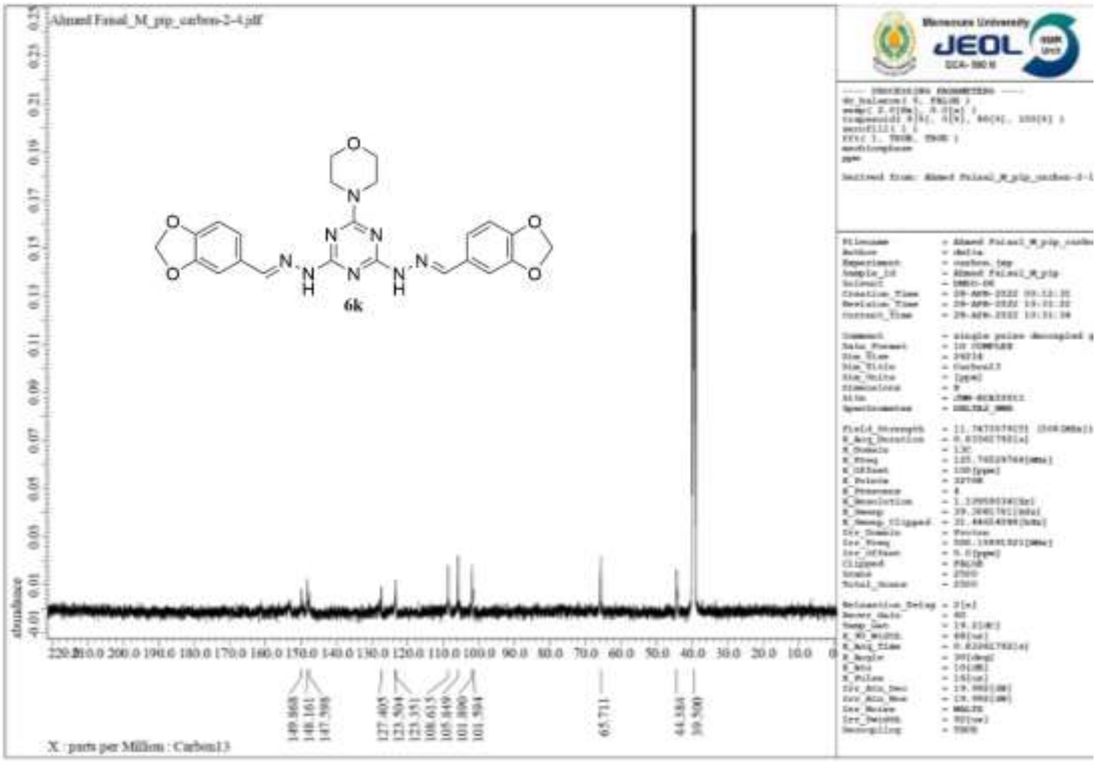

Figure S33. ^{13}**C NMR (125 MHz, DMSO) spectrum of compound 6k**

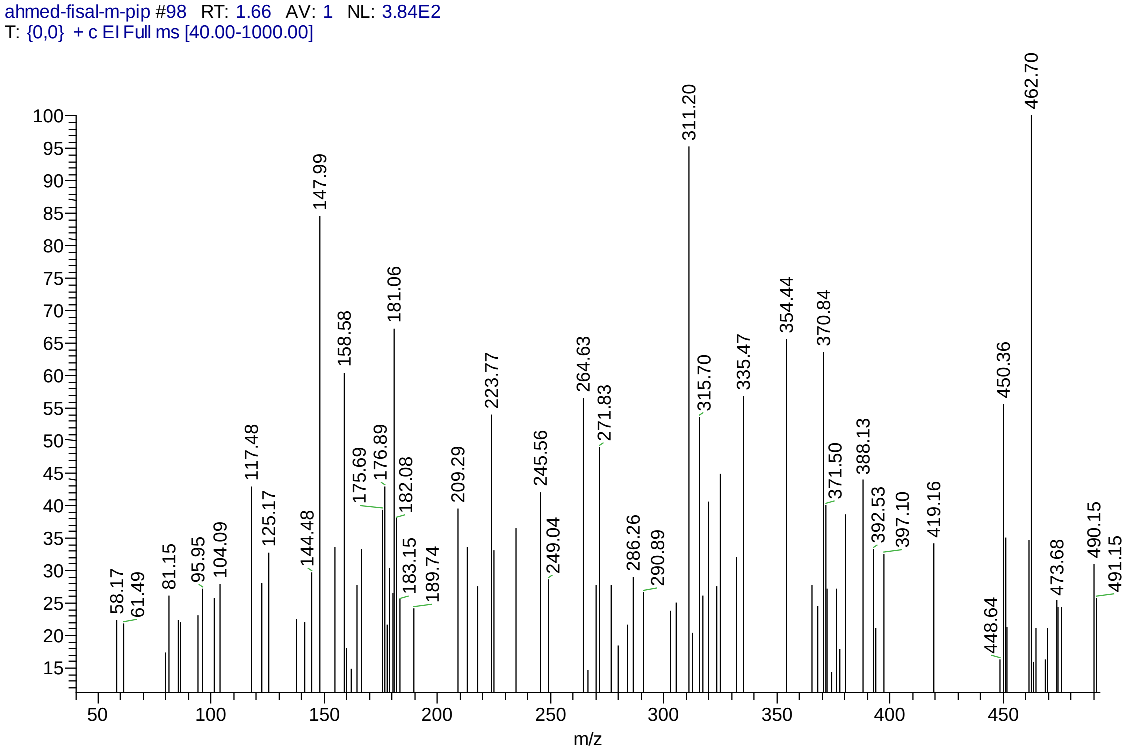

Figure S34. Mass spectrum of compound 6k

Information Classification: General

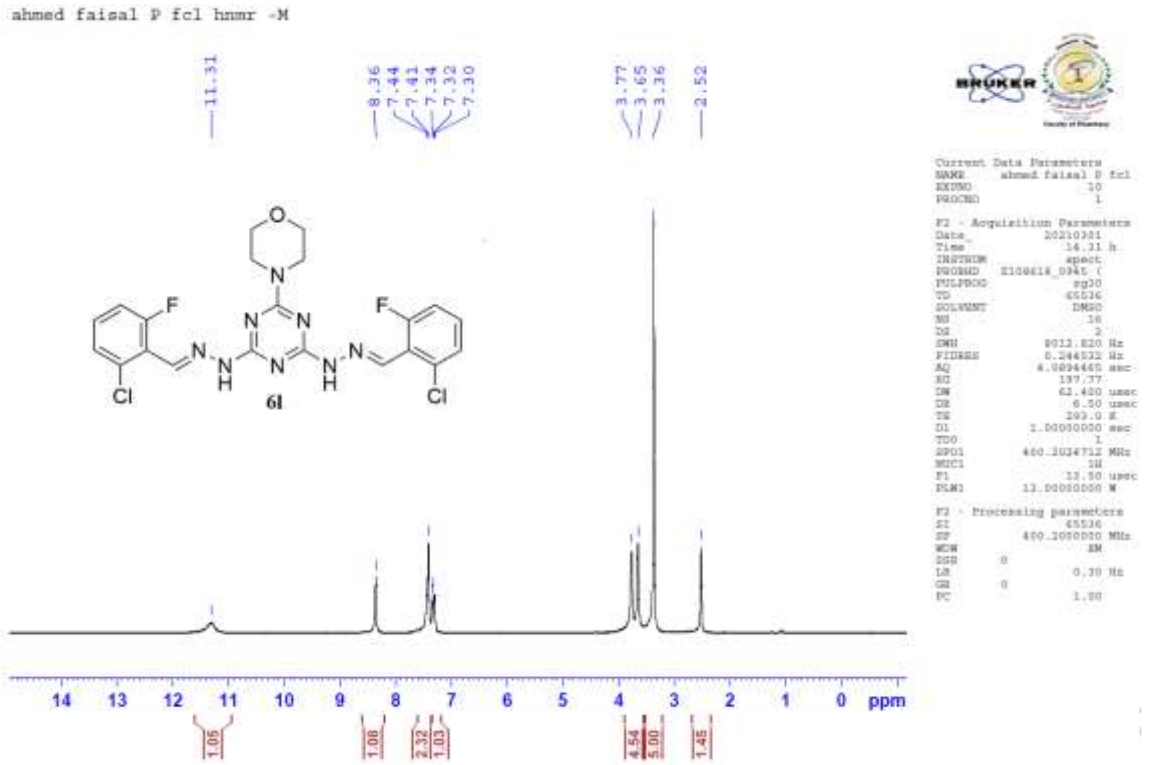

Figure S35. ^{1}H NMR (400 MHz, DMSO-d_6) spectrum of compound 6l

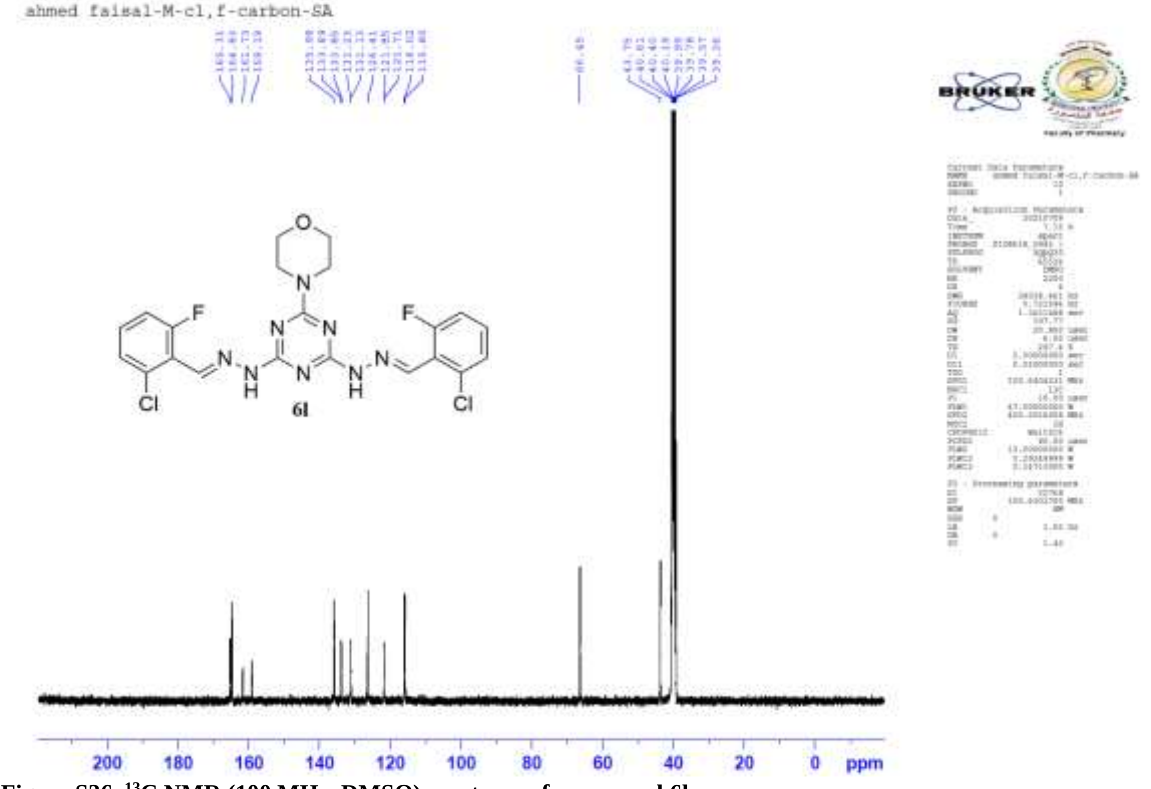

Figure S36. ^{13}C NMR (100 MHz, DMSO) spectrum of compound 6l

Information Classification: General

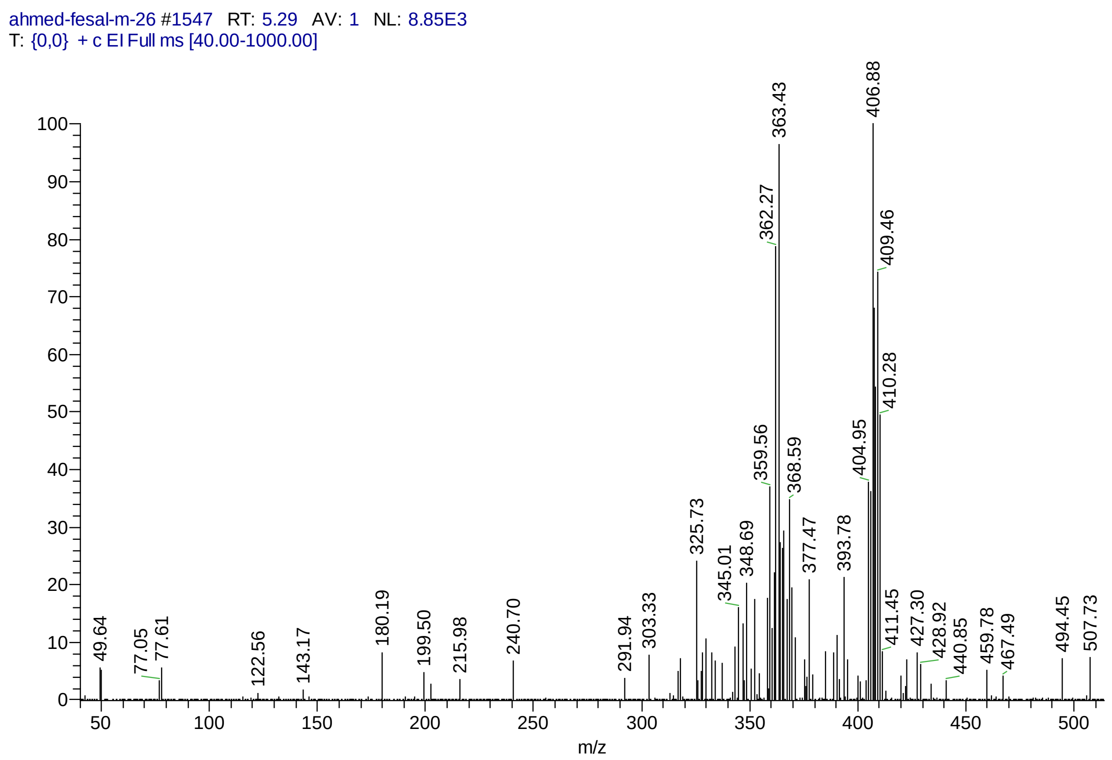

Figure S37. Mass spectrum of compound 6l

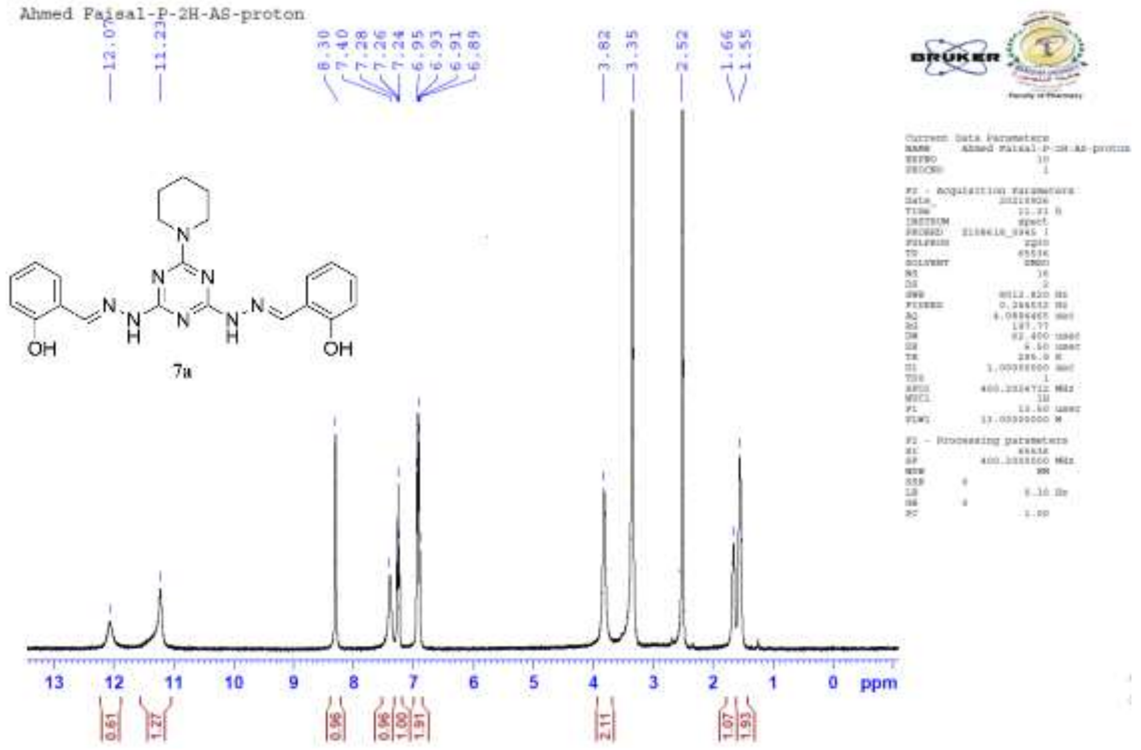

Figure S38. ^{1}H NMR (400 MHz, DMSO-d_6) spectrum of compound 7a

Information Classification: General

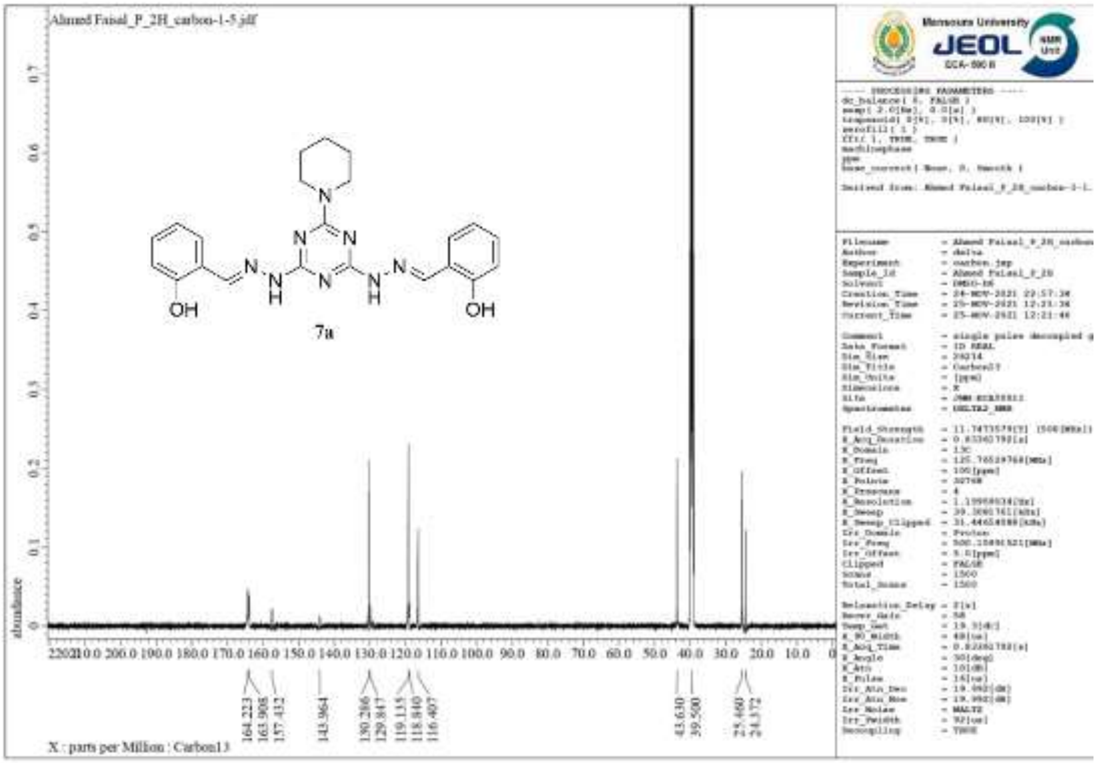

Figure S39. ^{13}**C NMR (125 MHz, DMSO) spectrum of compound 7a**

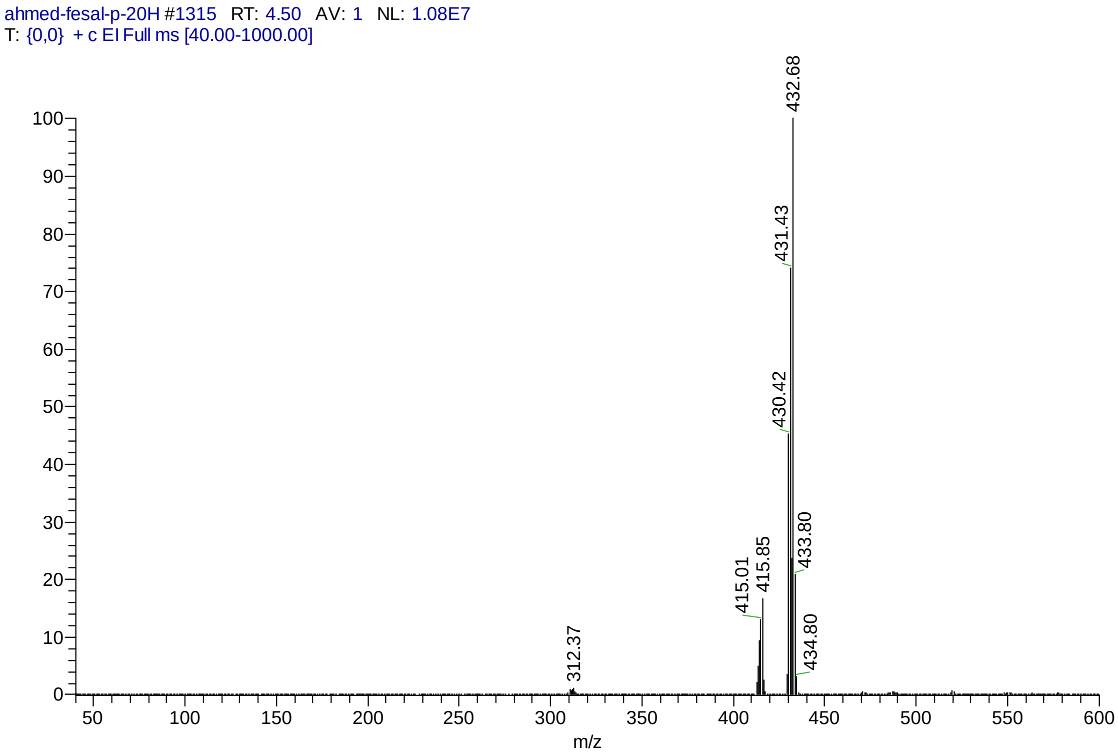

Figure S40. Mass spectrum of compound 7a

Information Classification: General

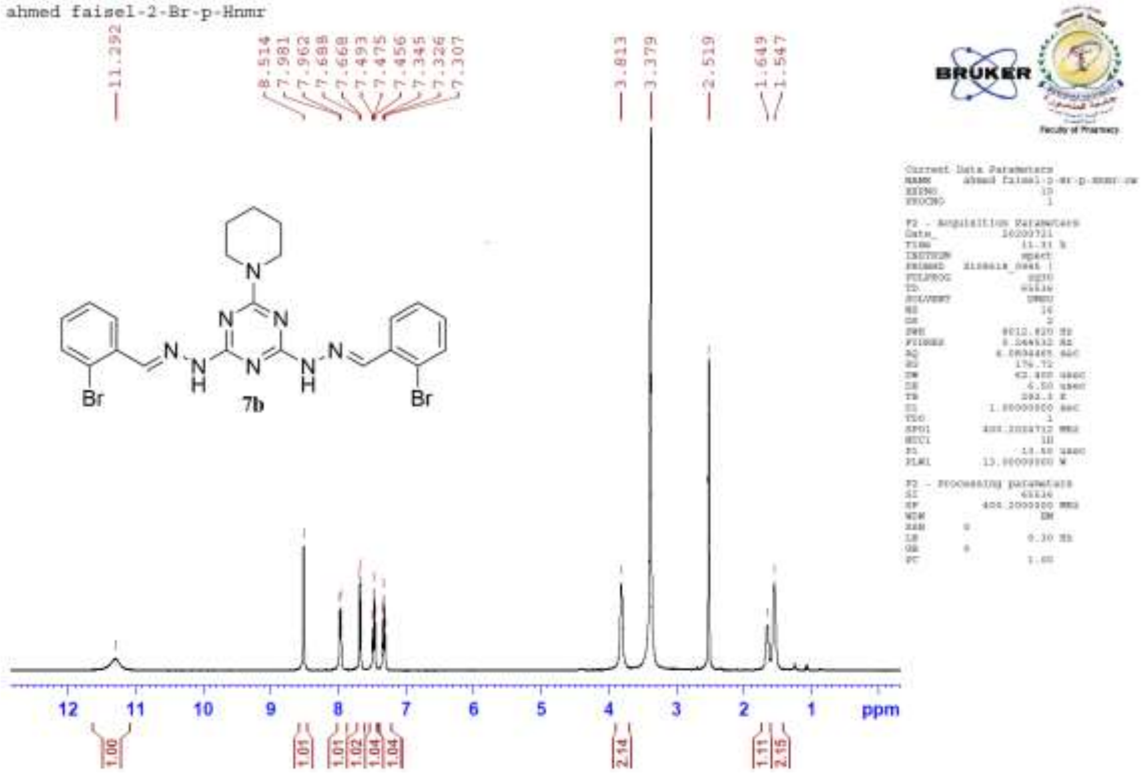

Figure S41. **^{1}H NMR (400 MHz, DMSO-d_6) spectrum of compound 7b**

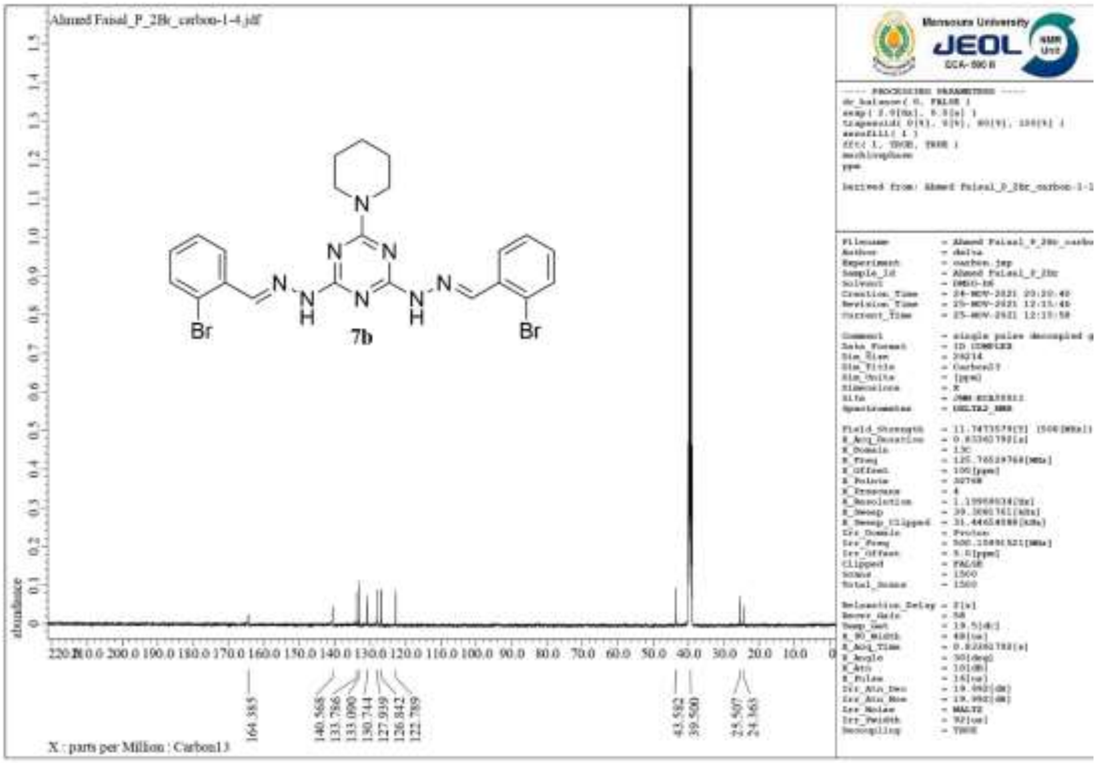

Figure S42. **^{13}C NMR (125 MHz, DMSO) spectrum of compound 7b**

Information Classification: General

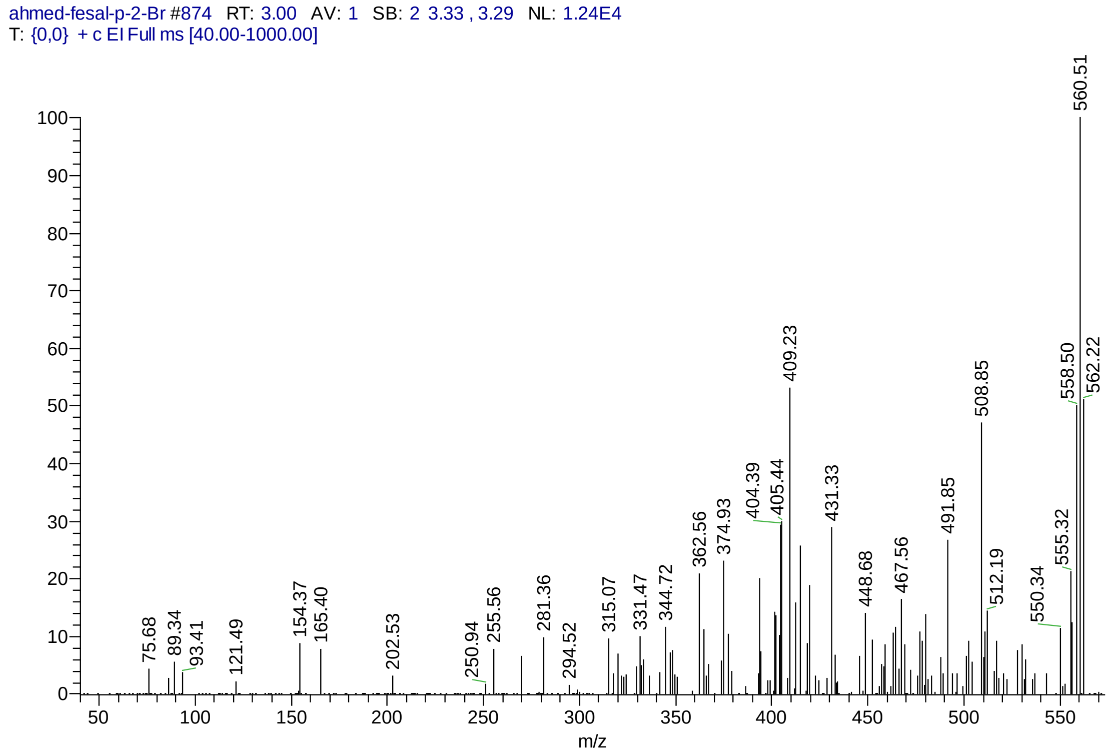

Figure S43. Mass spectrum of compound 7b

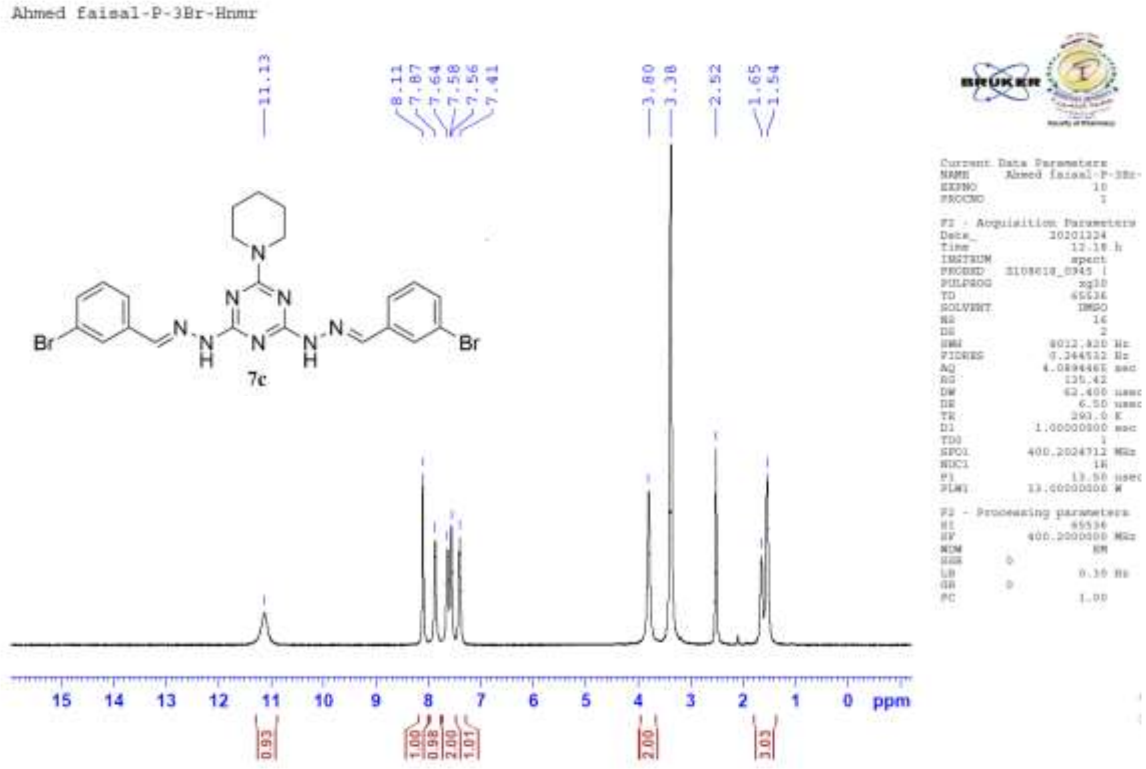

Figure S44. **^{1}H NMR (400 MHz, DMSO-d_6) spectrum of compound 7c**

Information Classification: General

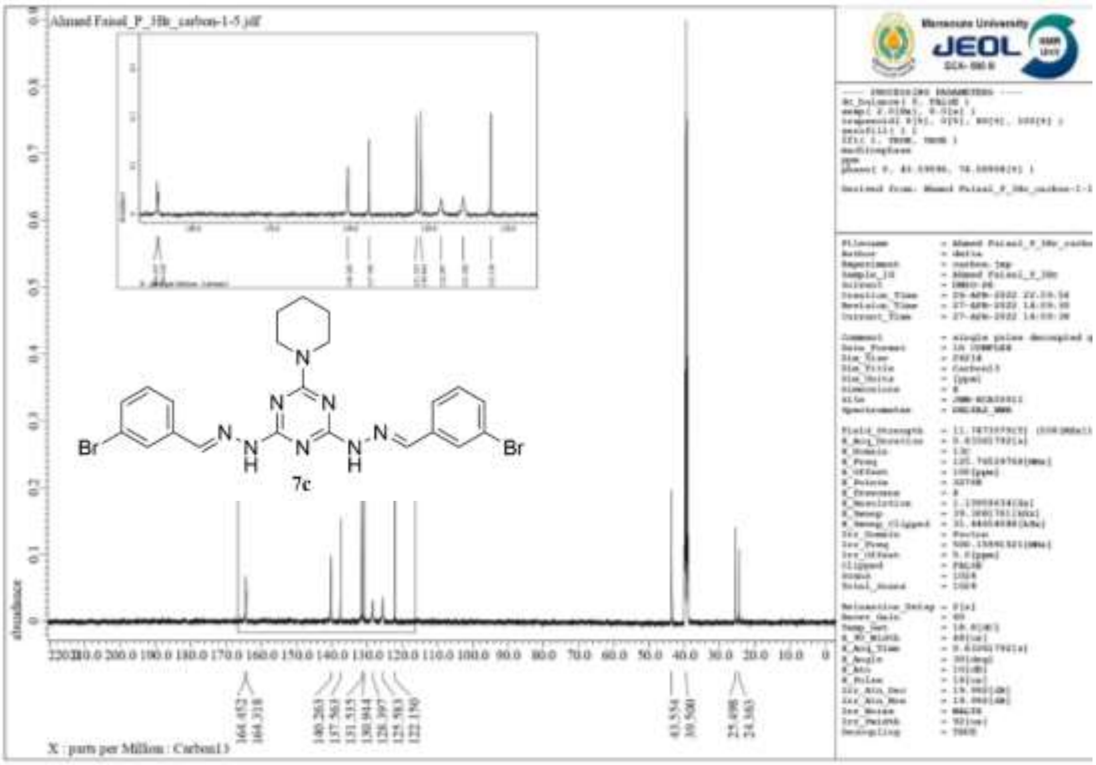

Figure S45. ^{13}C NMR (125 MHz, DMSO) spectrum of compound 7c

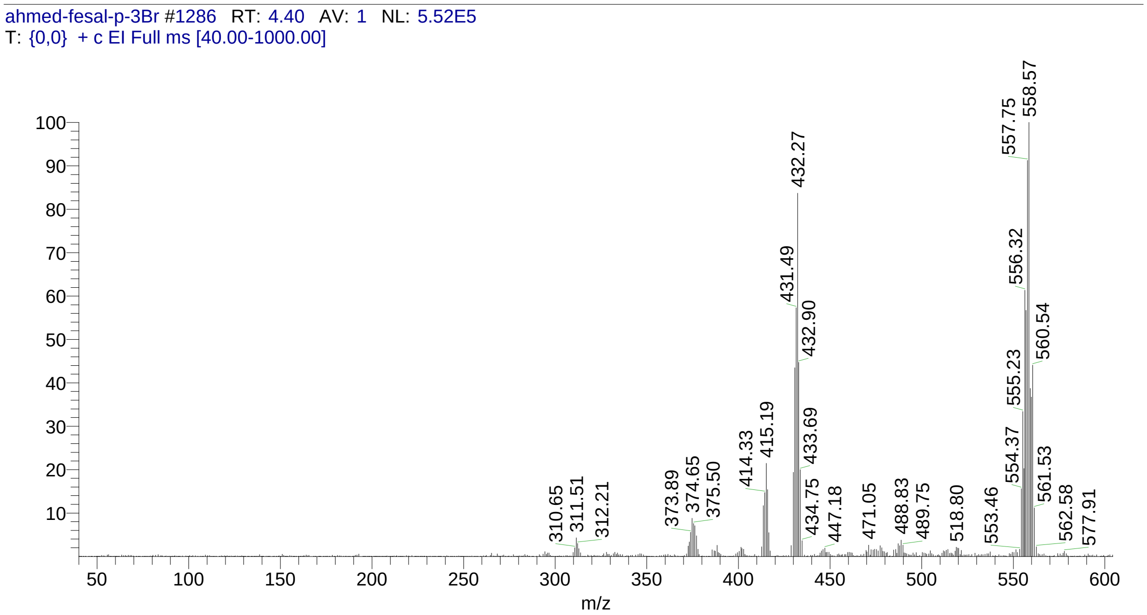

Figure S46. Mass spectrum of compound 7c

Information Classification: General

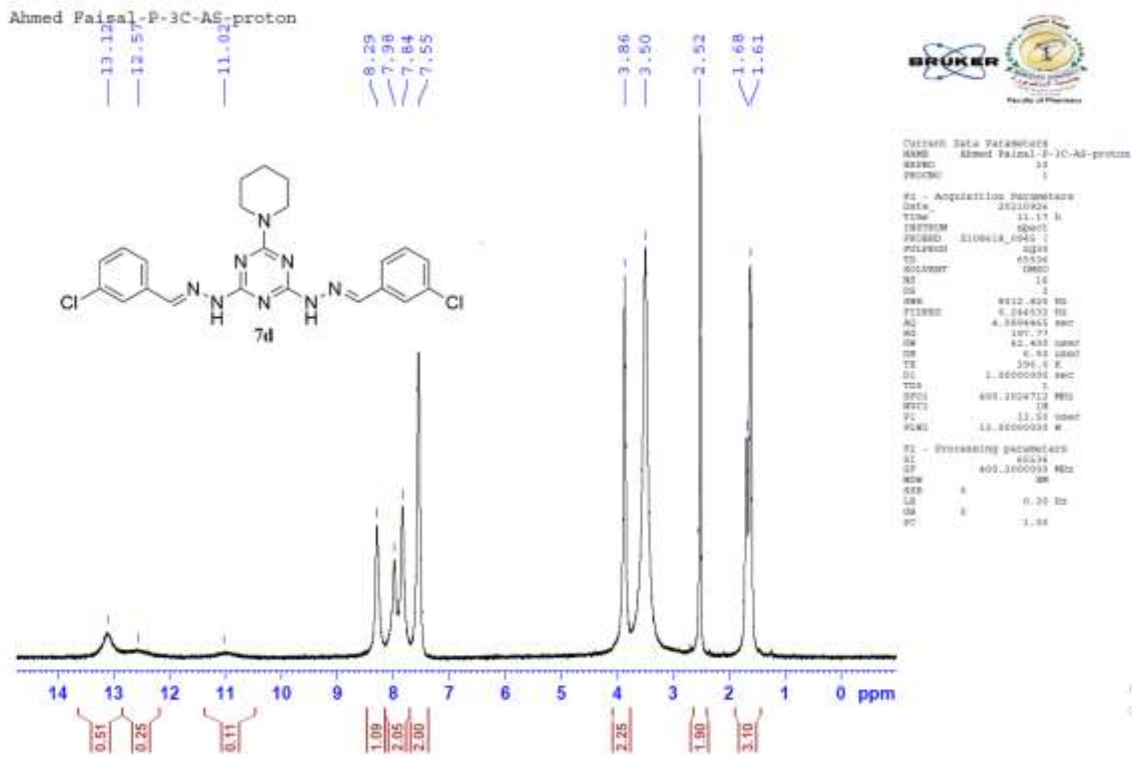

Figure S47. ^{1}**H NMR (400 MHz, DMSO-**d_6**) spectrum of compound 7d**

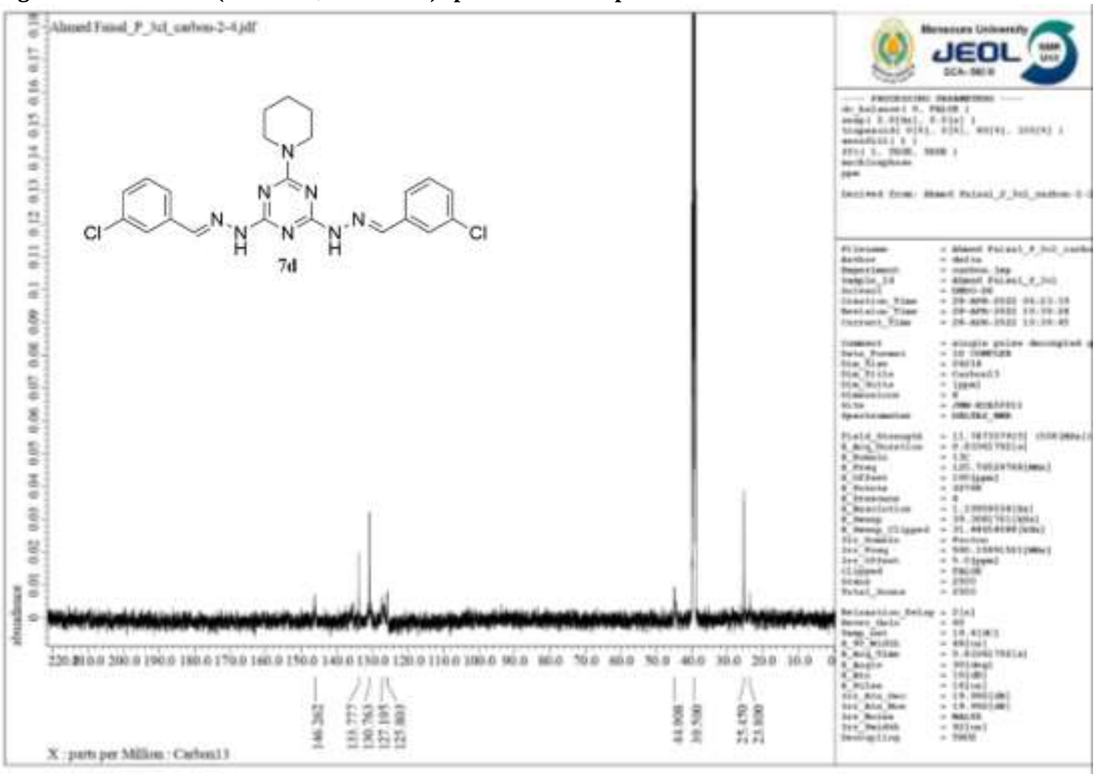

Figure S48. ^{13}**C NMR (125 MHz, DMSO) spectrum of compound 7d**

Information Classification: General

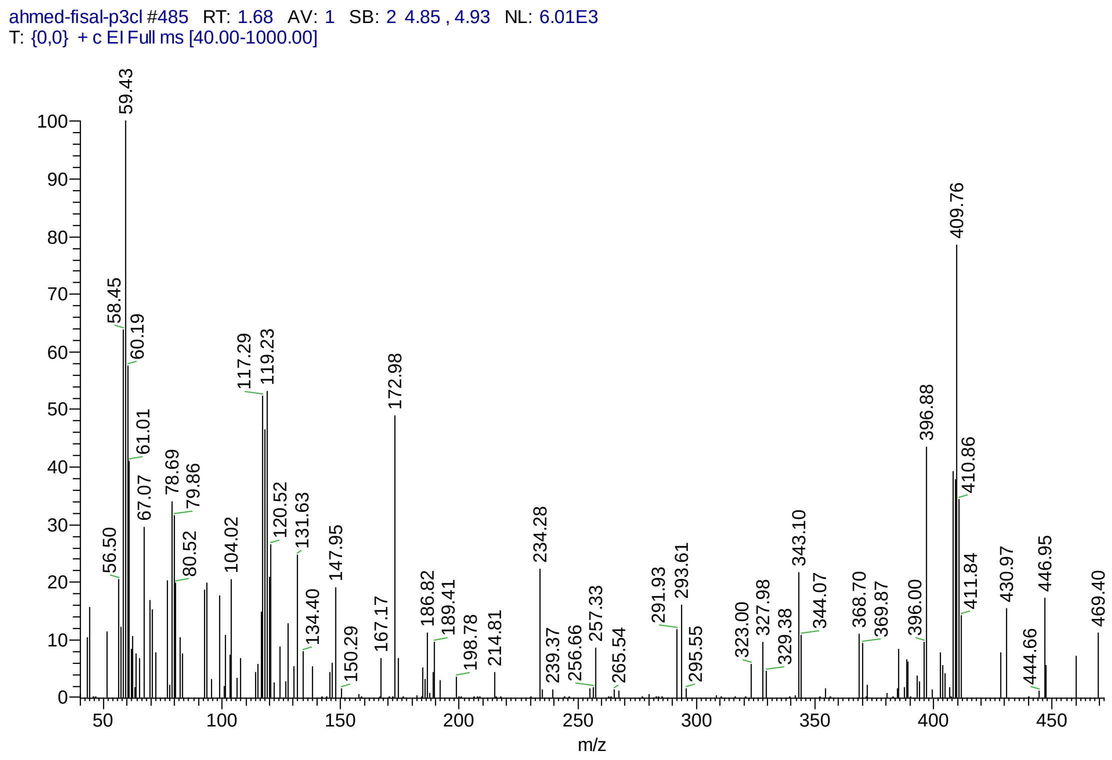

Figure S49. Mass spectrum of compound 7d

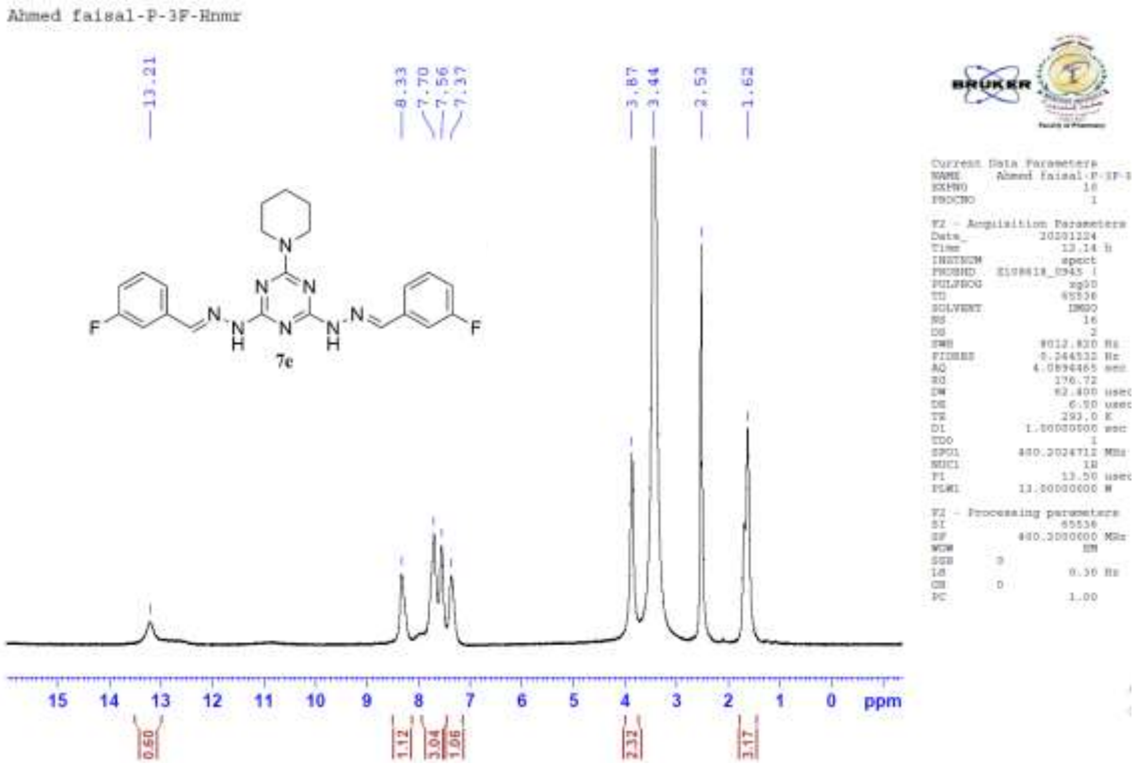

Figure S50. ^{1}H NMR (400 MHz, DMSO) spectrum of compound 7e

Information Classification: General

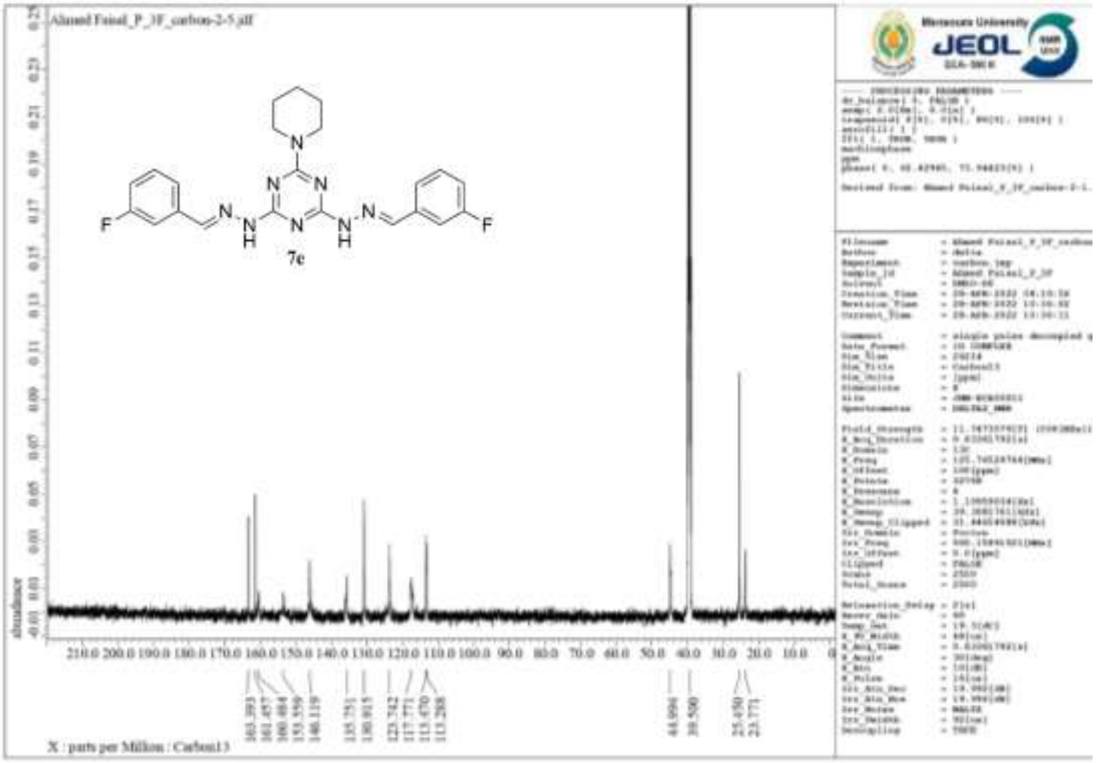

Figure S51. ^{13}**C NMR (125 MHz, DMSO) spectrum of compound 7e**

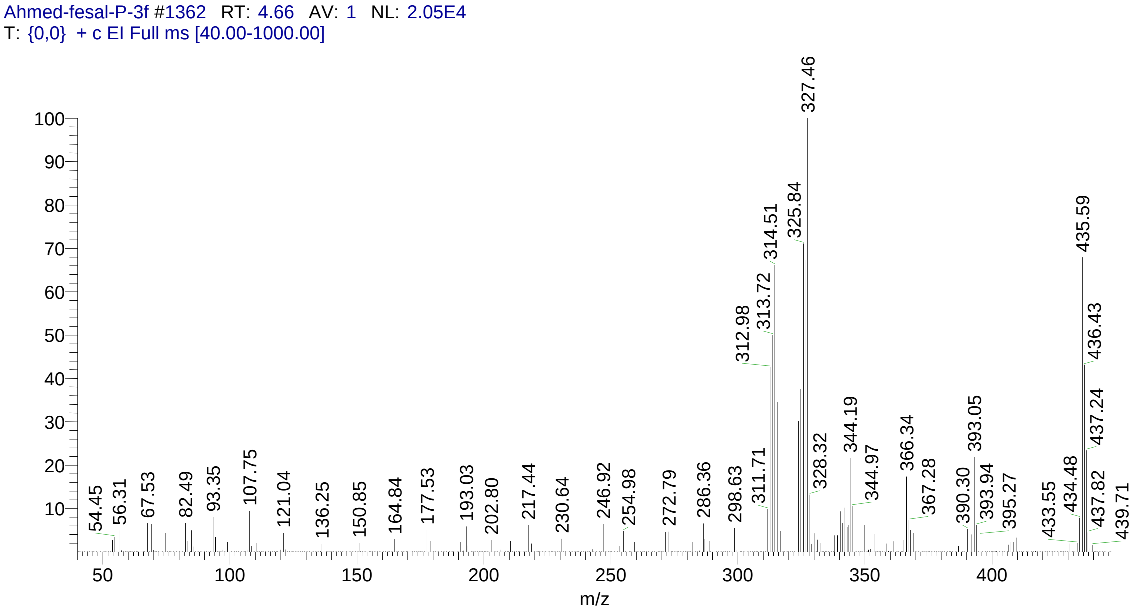

Figure S52. Mass spectrum of compound 7e

Information Classification: General

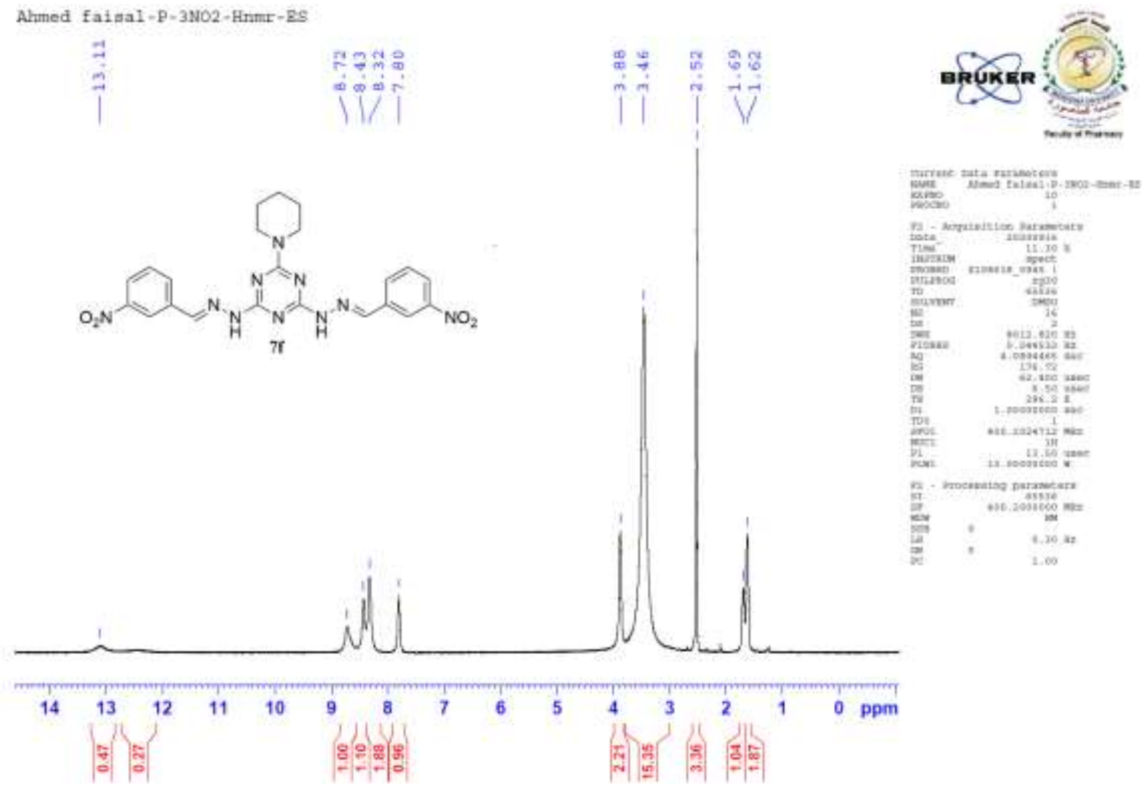

Figure S53. ^{1}H **NMR (400 MHz, DMSO-d_6) spectrum of compound 7f**

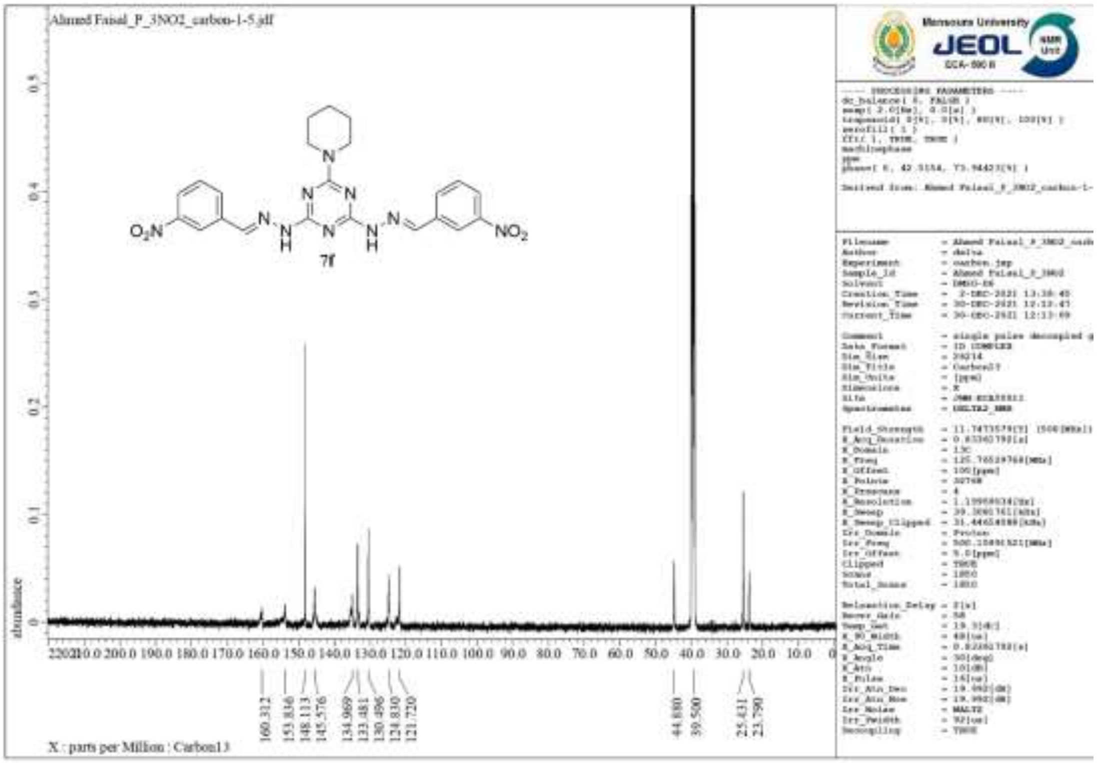

Figure S54. ^{13}C **NMR (125 MHz, DMSO) spectrum of compound 7f**

Information Classification: General

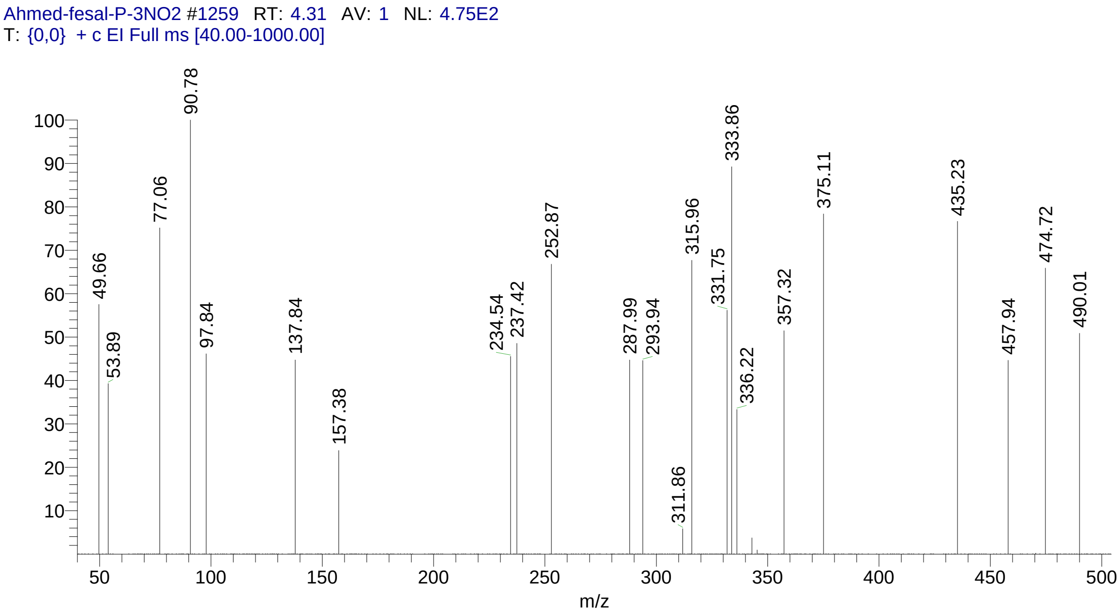

Figure S55. Mass spectrum of compound 7f

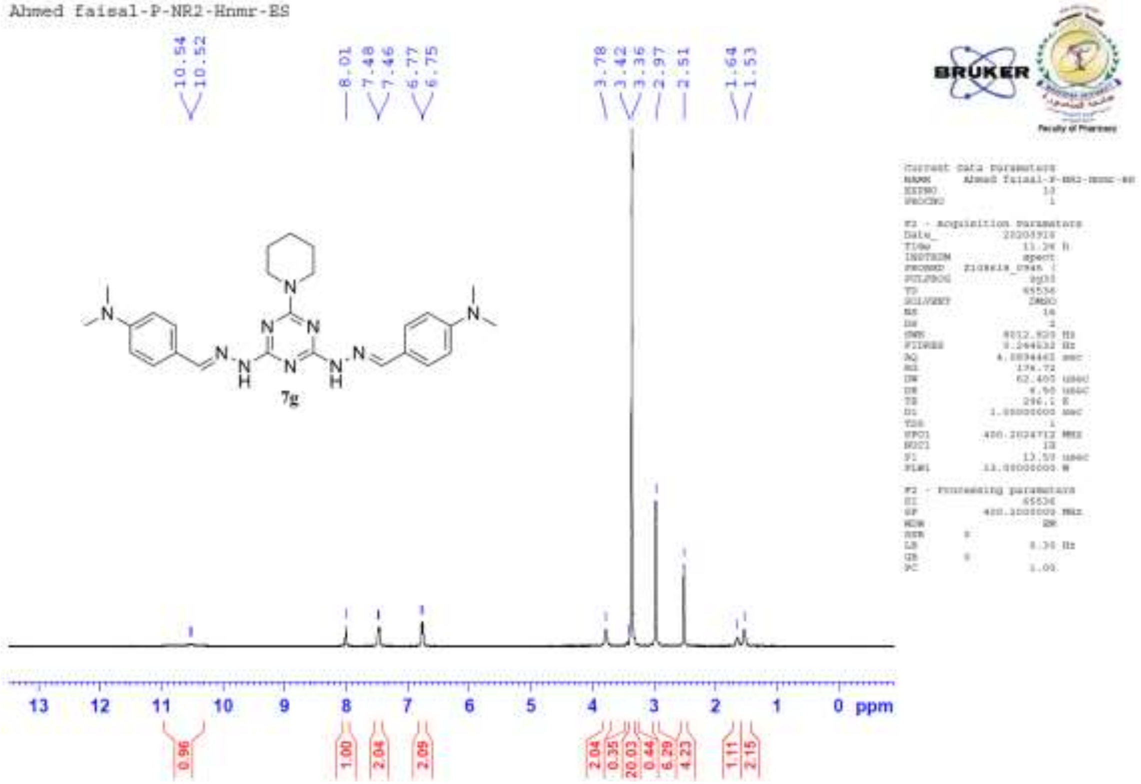

Figure S56. ¹H NMR (400 MHz, DMSO-d_6) spectrum of compound 7g

Information Classification: General

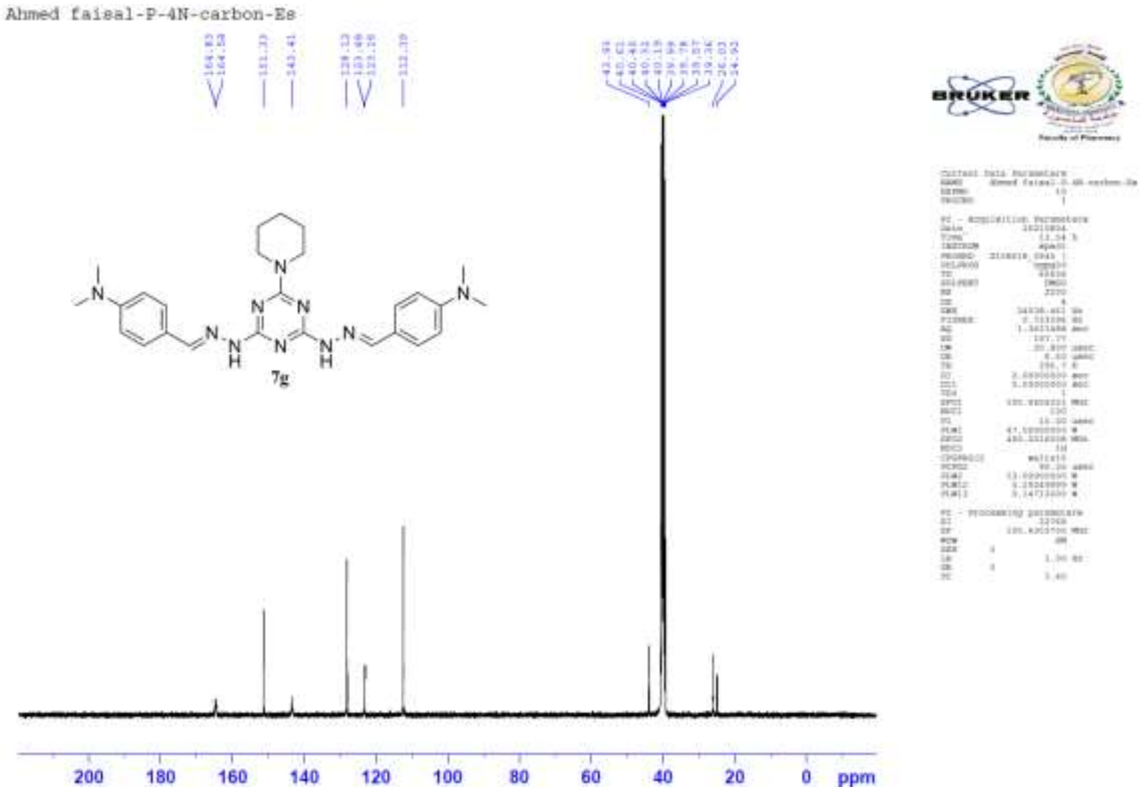

Figure S57. ^{13}**C NMR (100 MHz, DMSO) spectrum of compound 7g**

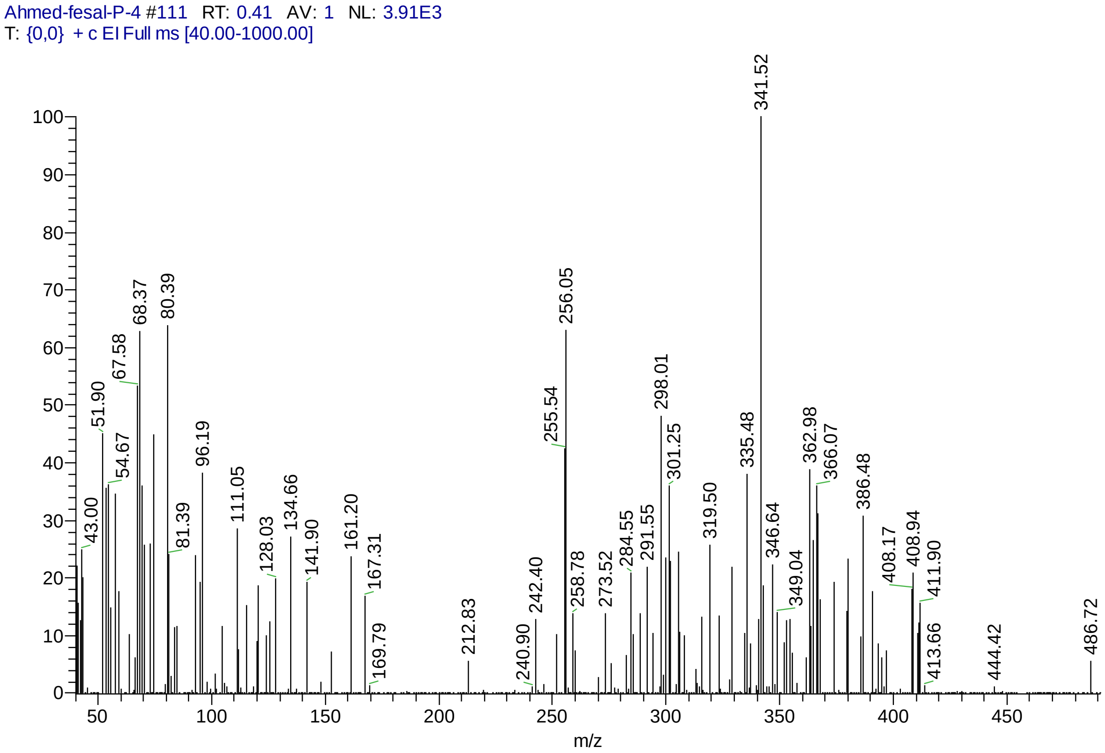

Figure S58. Mass spectrum of compound 7g

Information Classification: General

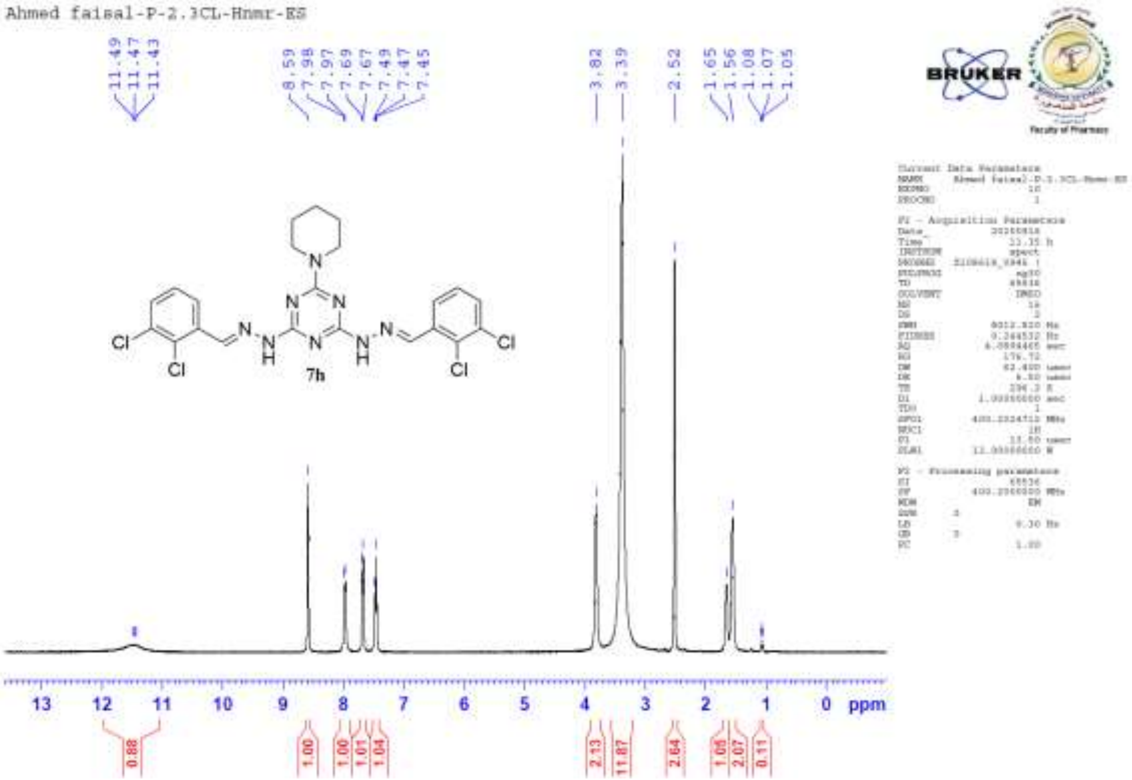

Figure S59. ^{1}H NMR (400 MHz, DMSO-d_6) spectrum of compound 7h

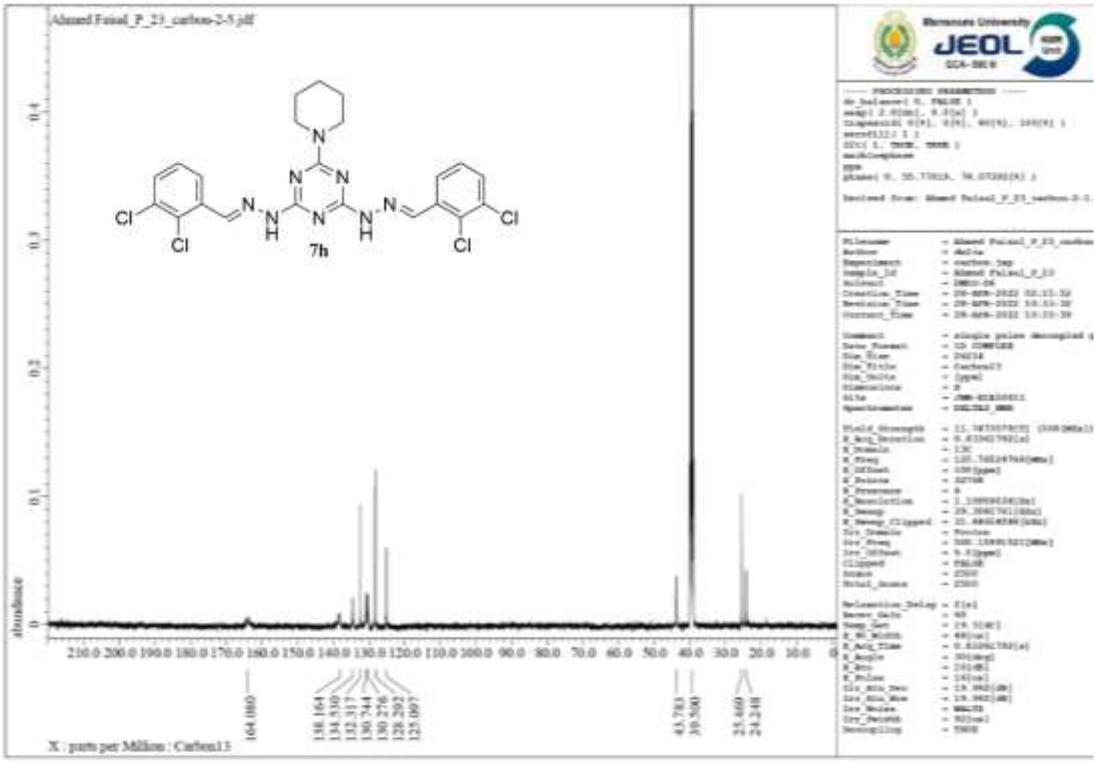

Figure S60. ^{13}C NMR (125 MHz, DMSO) spectrum of compound 7h

Information Classification: General

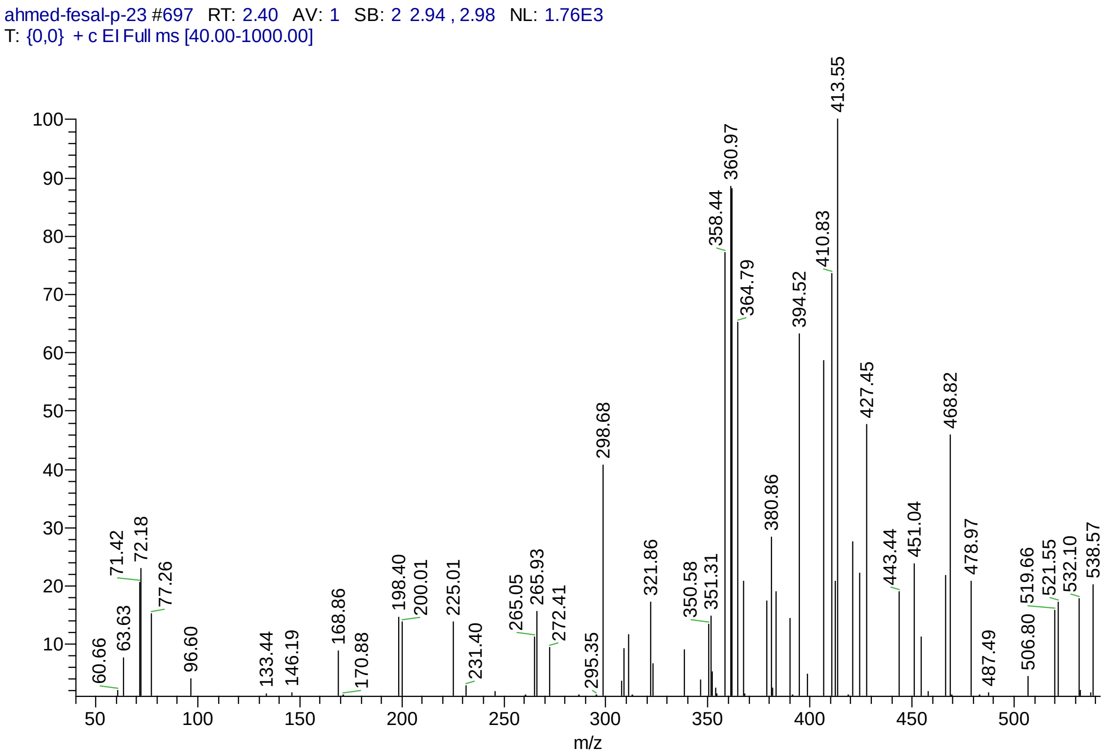

Figure S61. Mass spectrum of compound 7h

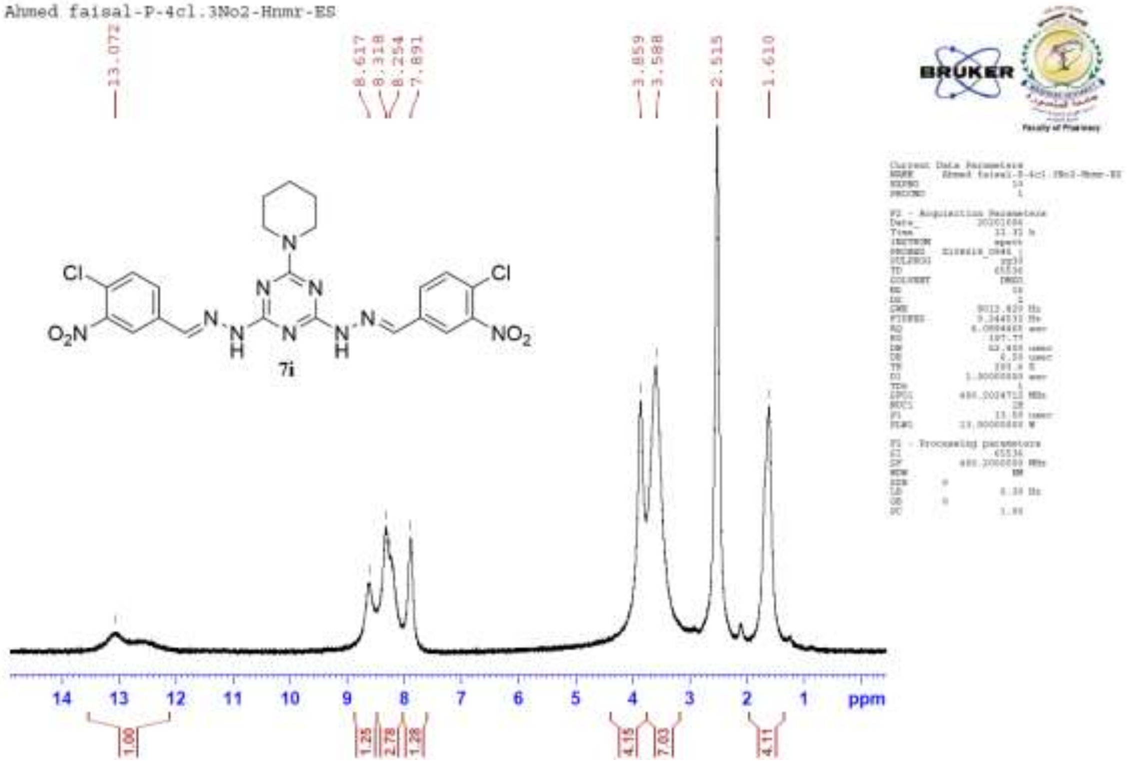

Figure S62. **^{1}H NMR (400 MHz, DMSO-*d*$_6$) spectrum of compound 7i**

Information Classification: General

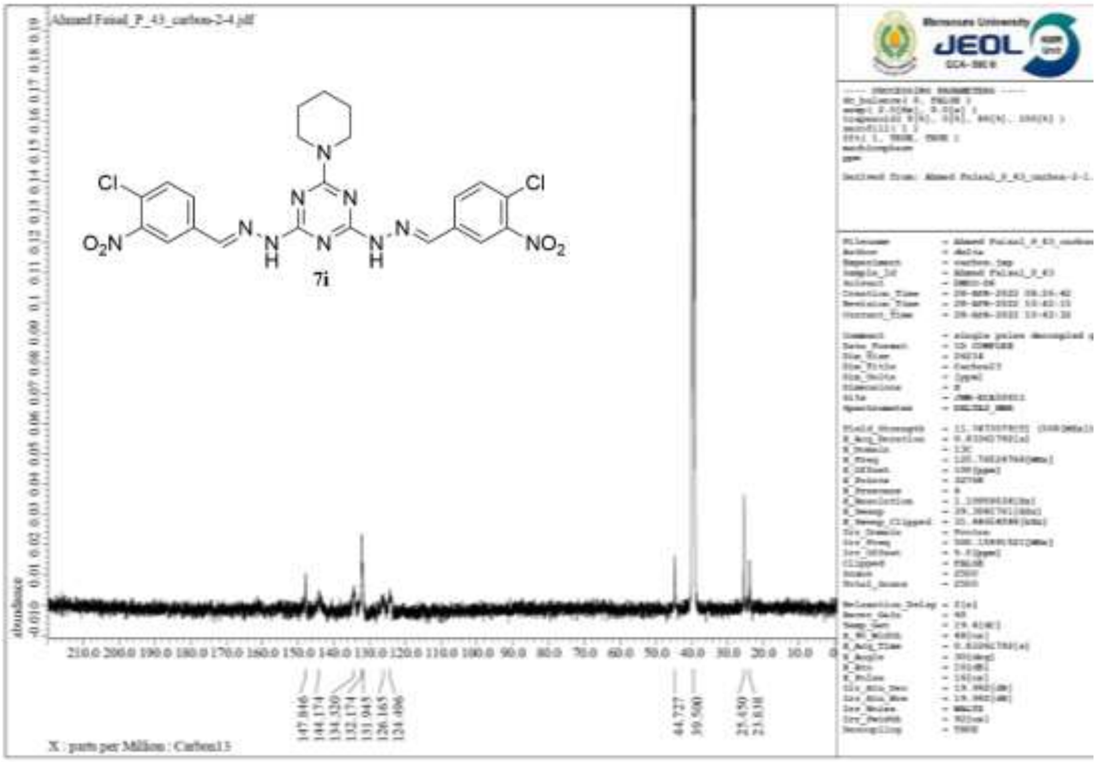

Figure S63. ^{13}**C NMR (125 MHz, DMSO) spectrum of compound 7i**

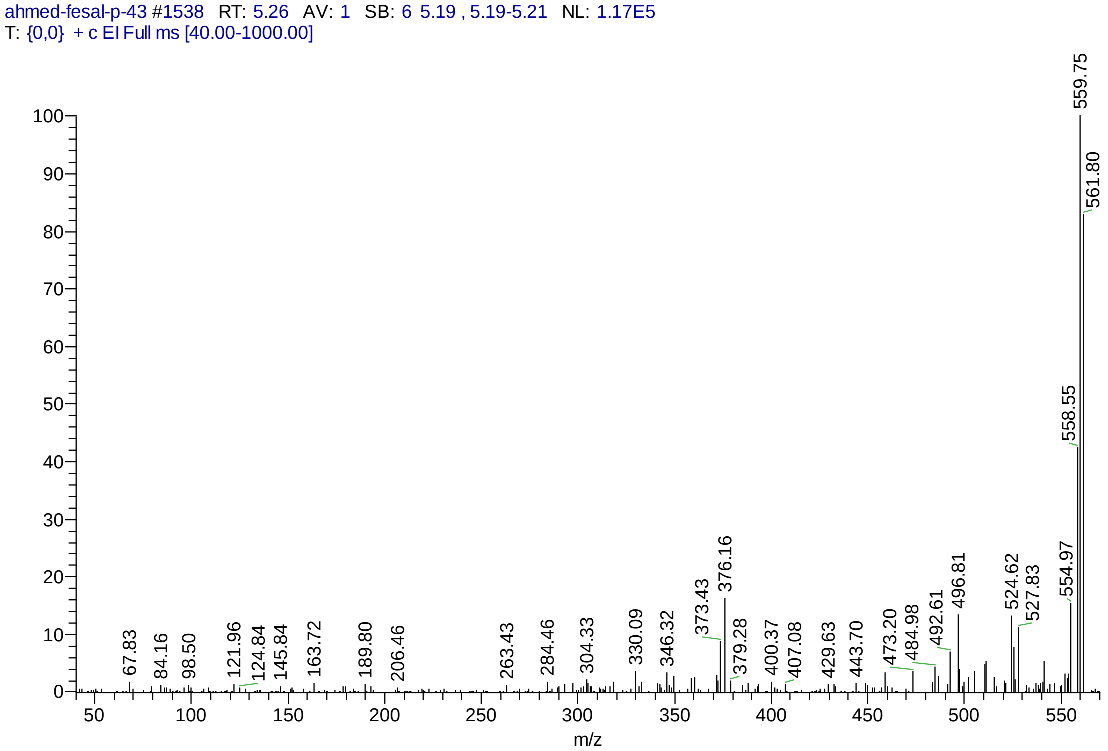

Figure S64. Mass spectrum of compound 7i

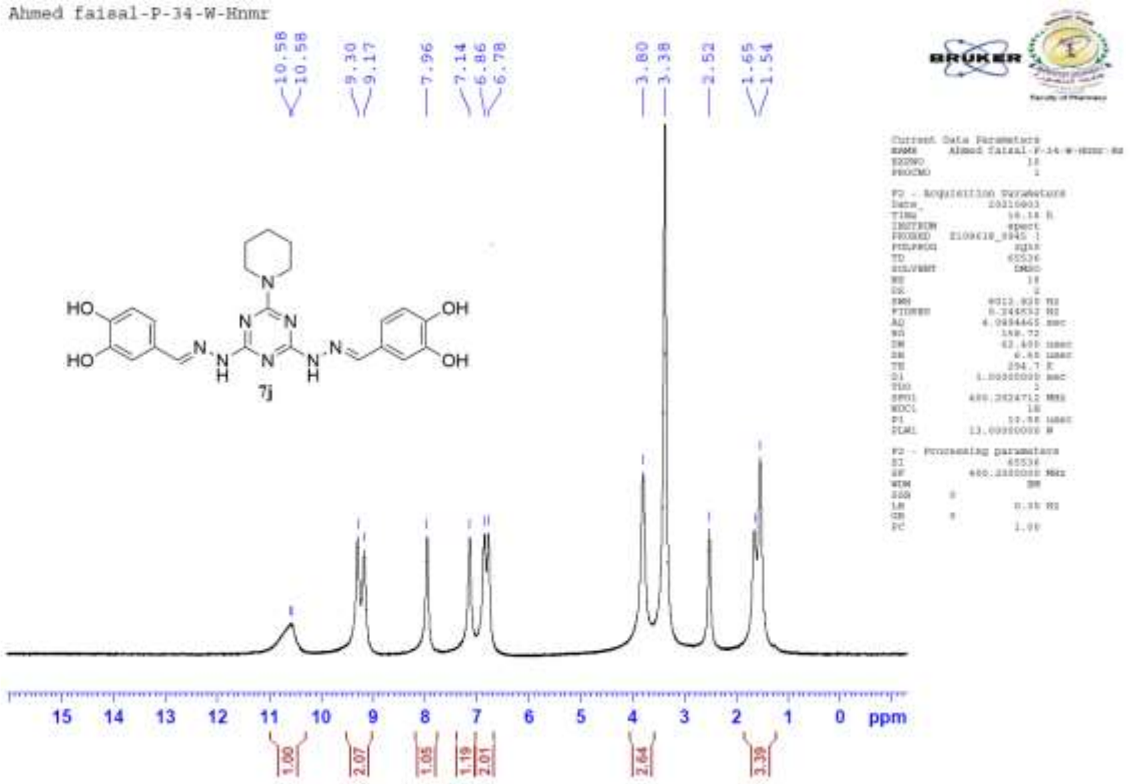

Figure S65. [1]**H NMR (400 MHz, DMSO-*d6*) spectrum of compound 7j**

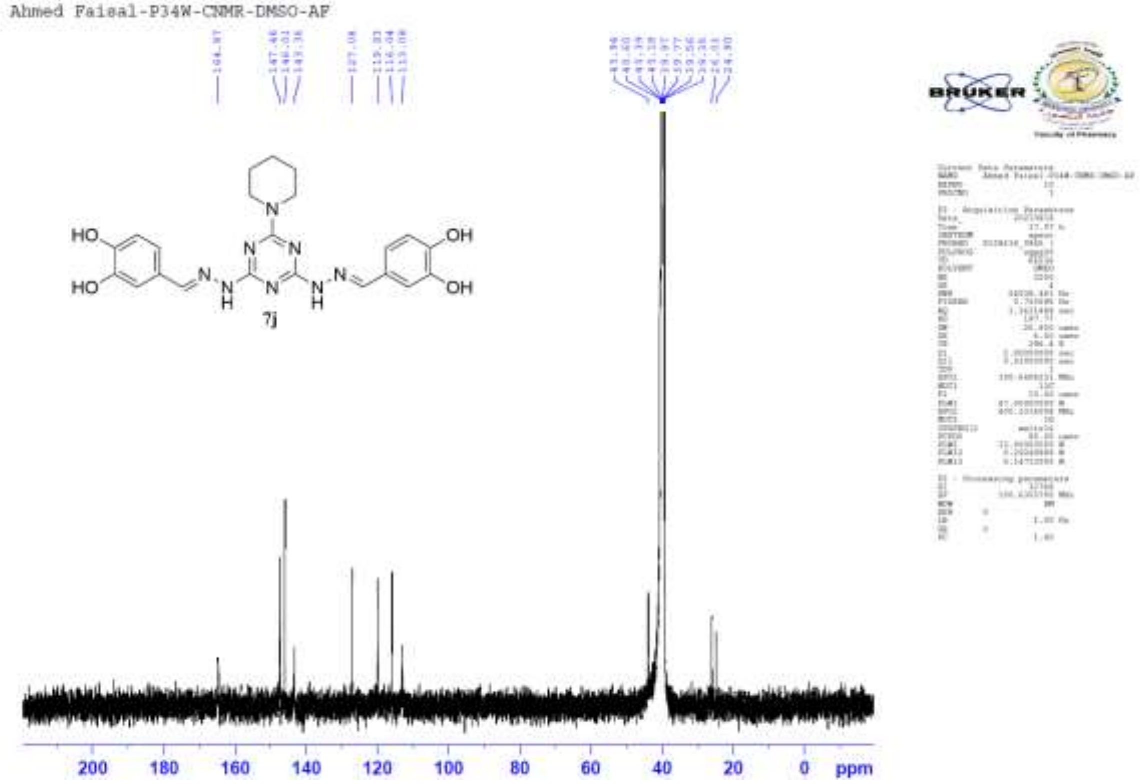

Figure S66. [13]**C NMR (100 MHz, DMSO) spectrum of compound 7j**

Information Classification: General

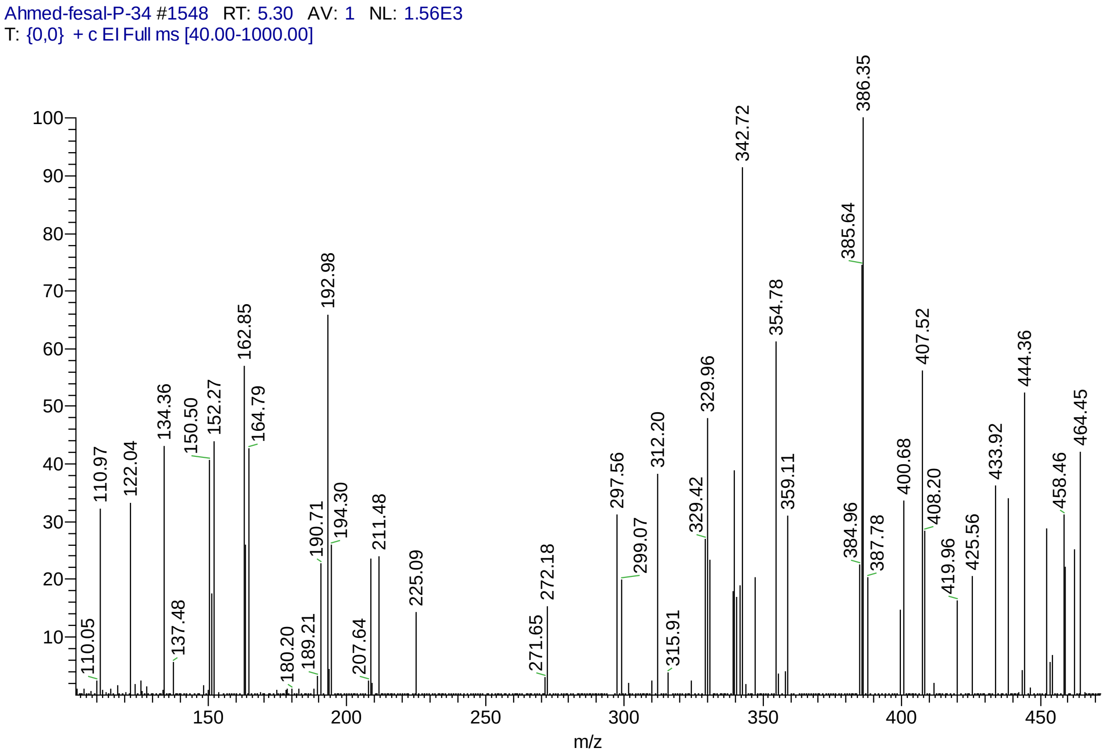

Figure S67. Mass spectrum of compound 7j

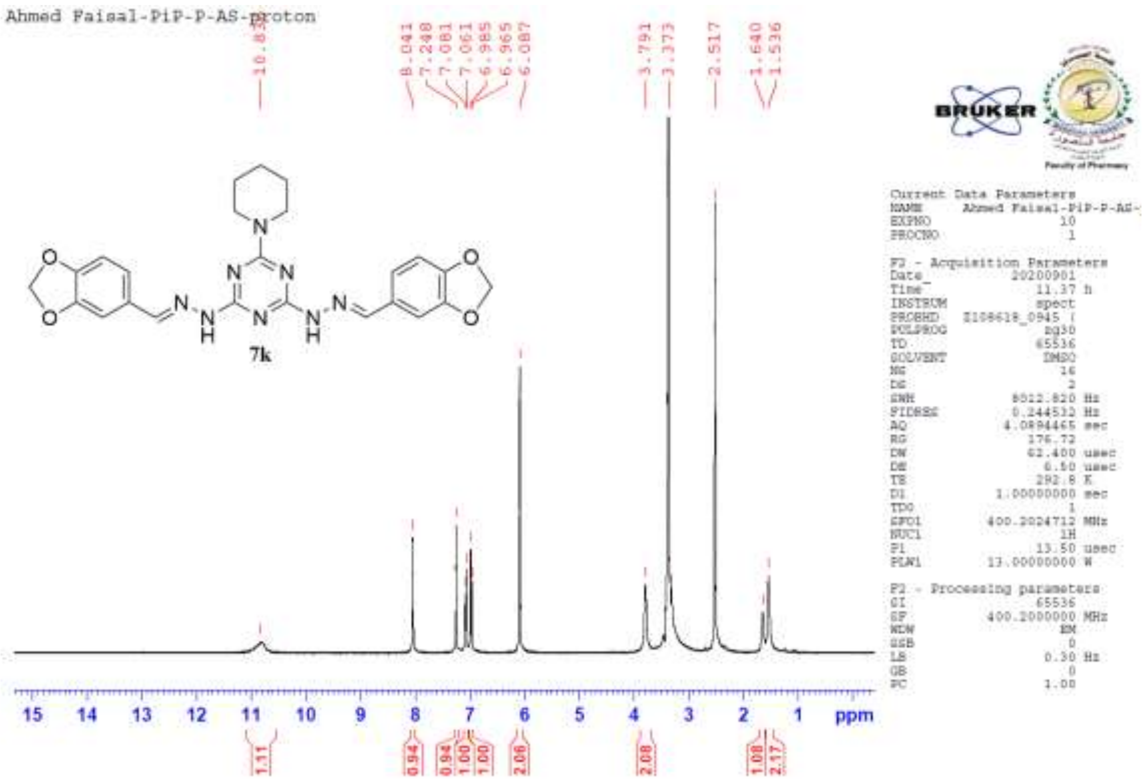

Information Classification: General

Figure S68. ^{1}H NMR (400 MHz, DMSO-d_6) spectrum of compound 7k

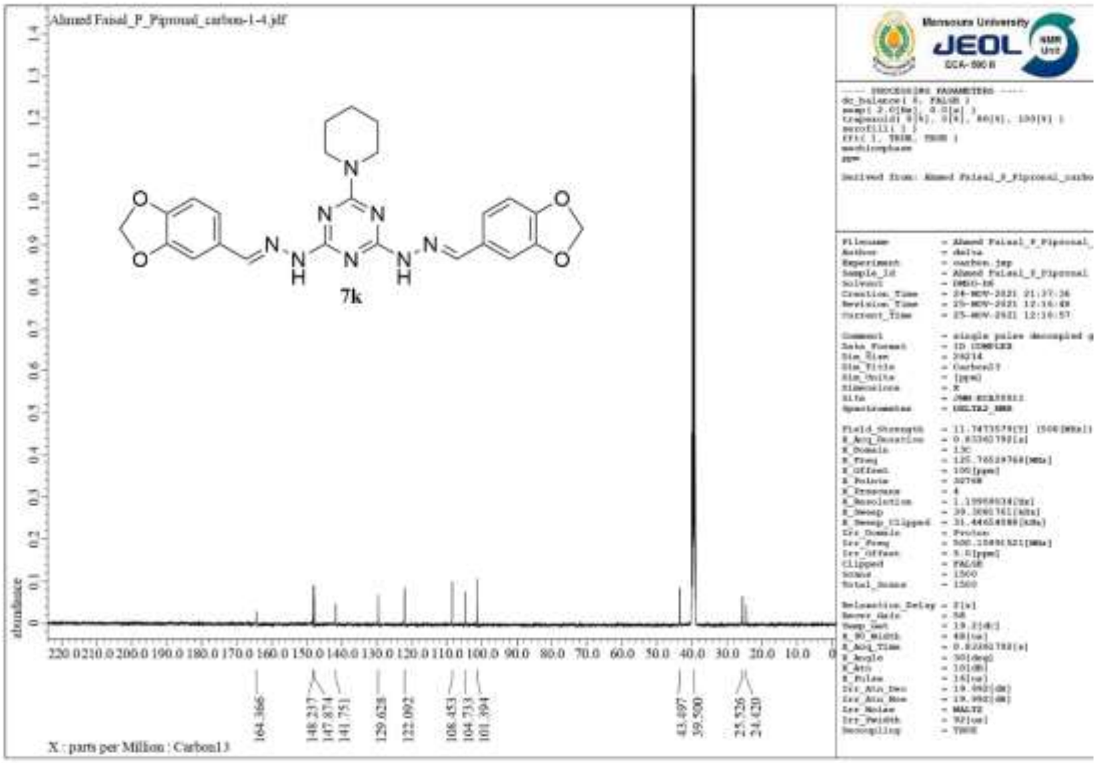

Figure S69. ^{13}C NMR (125 MHz, DMSO) spectrum of compound 7k

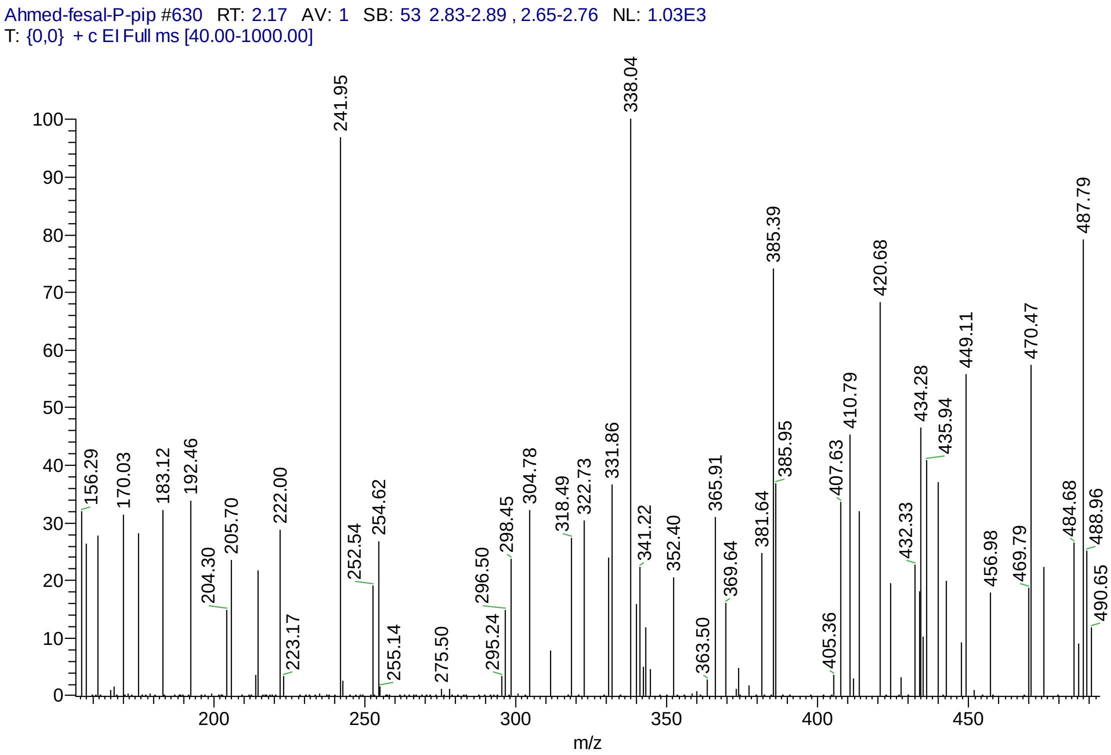

Figure S70. Mass spectrum of compound 7k

Information Classification: General

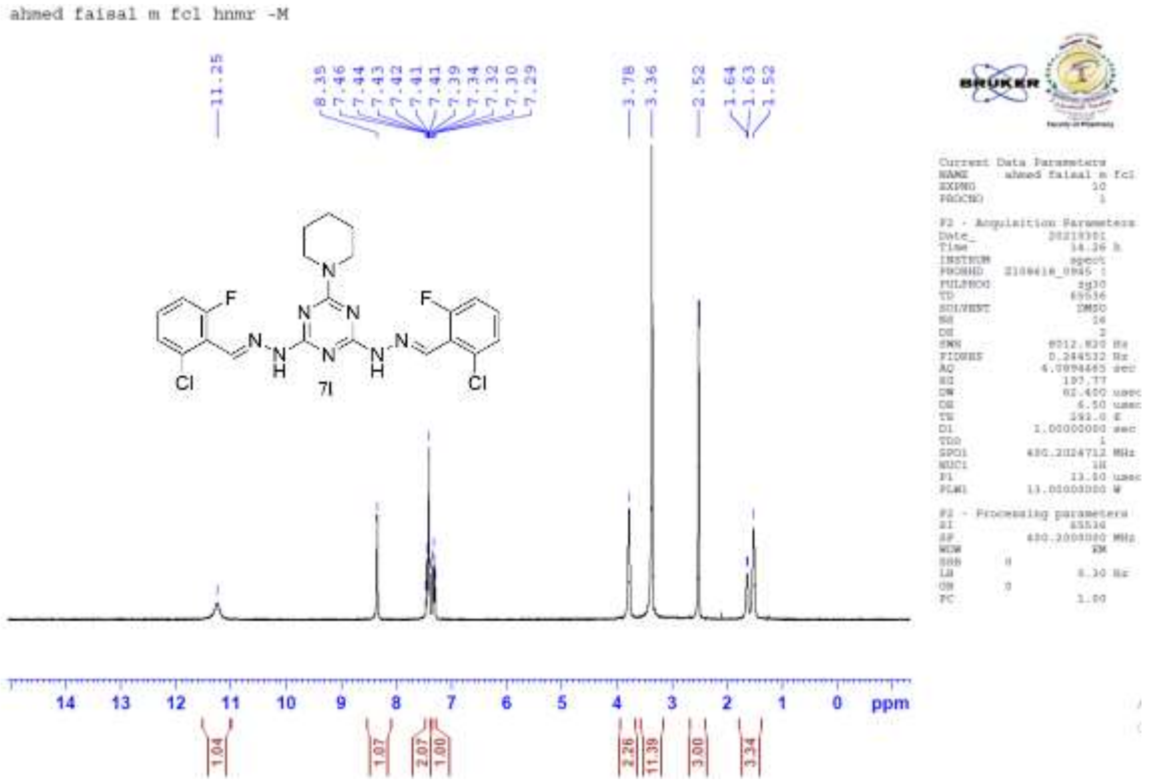

Figure S71. ^{1}H NMR (400 MHz, DMSO-d_6) spectrum of compound 7l

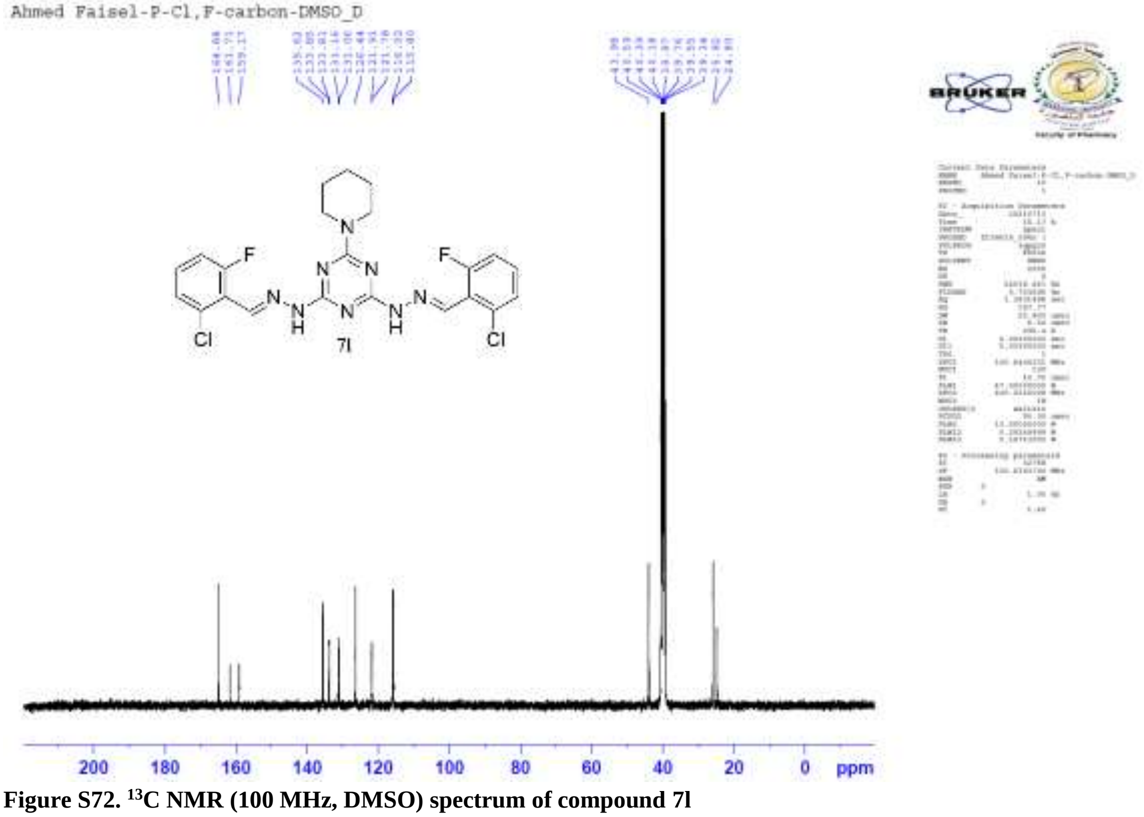

Figure S72. ^{13}C NMR (100 MHz, DMSO) spectrum of compound 7l

Information Classification: General

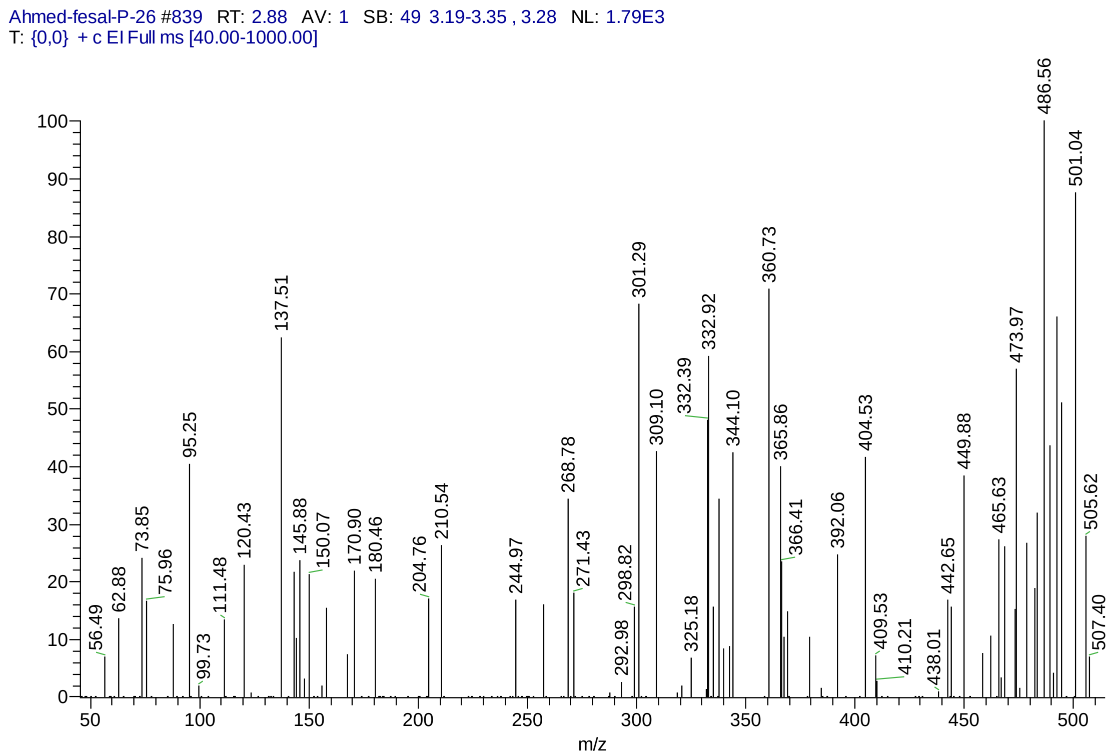

Figure S73. Mass spectrum of compound 7l

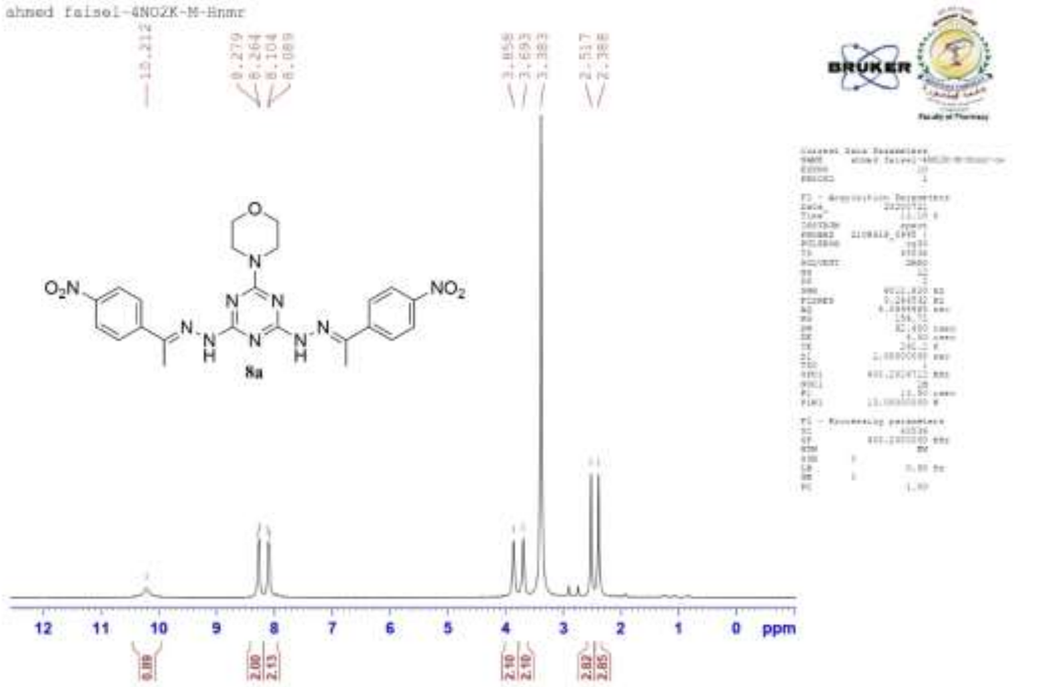

Figure S74. ^{1}H NMR (400 MHz, DMSO-d_6) spectrum of compound 8a

Information Classification: General

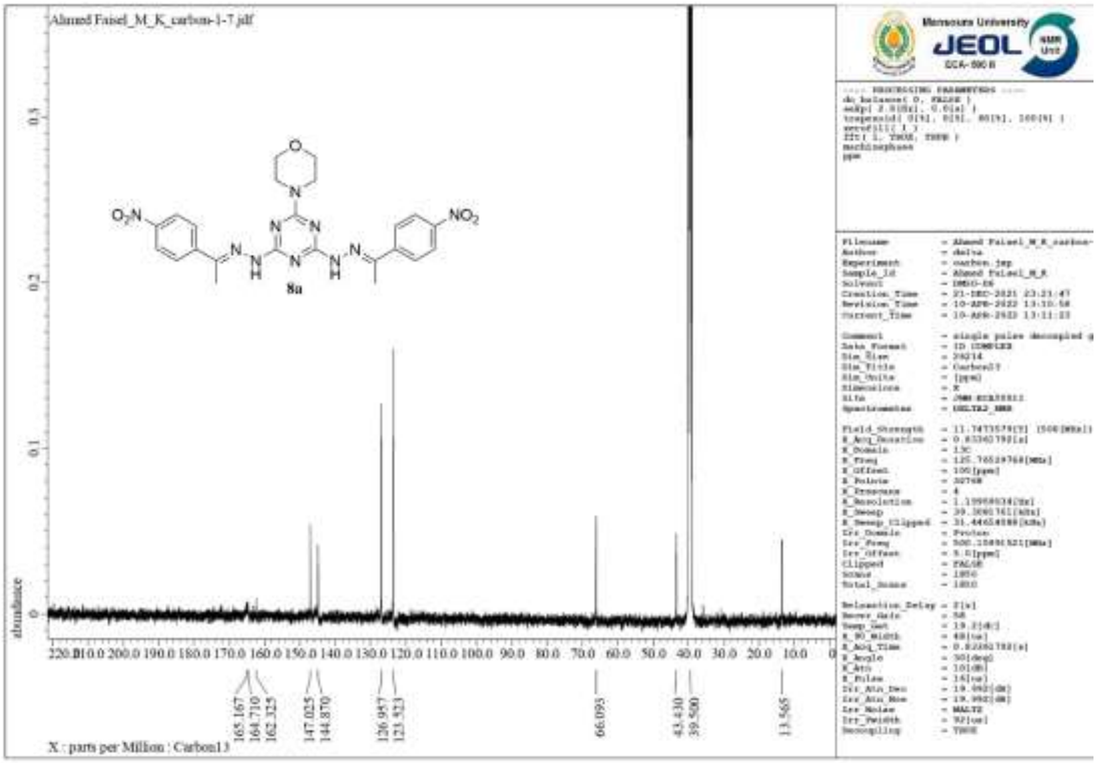

Figure S75. ^{13}**C NMR (125 MHz, DMSO) spectrum of compound 8a**

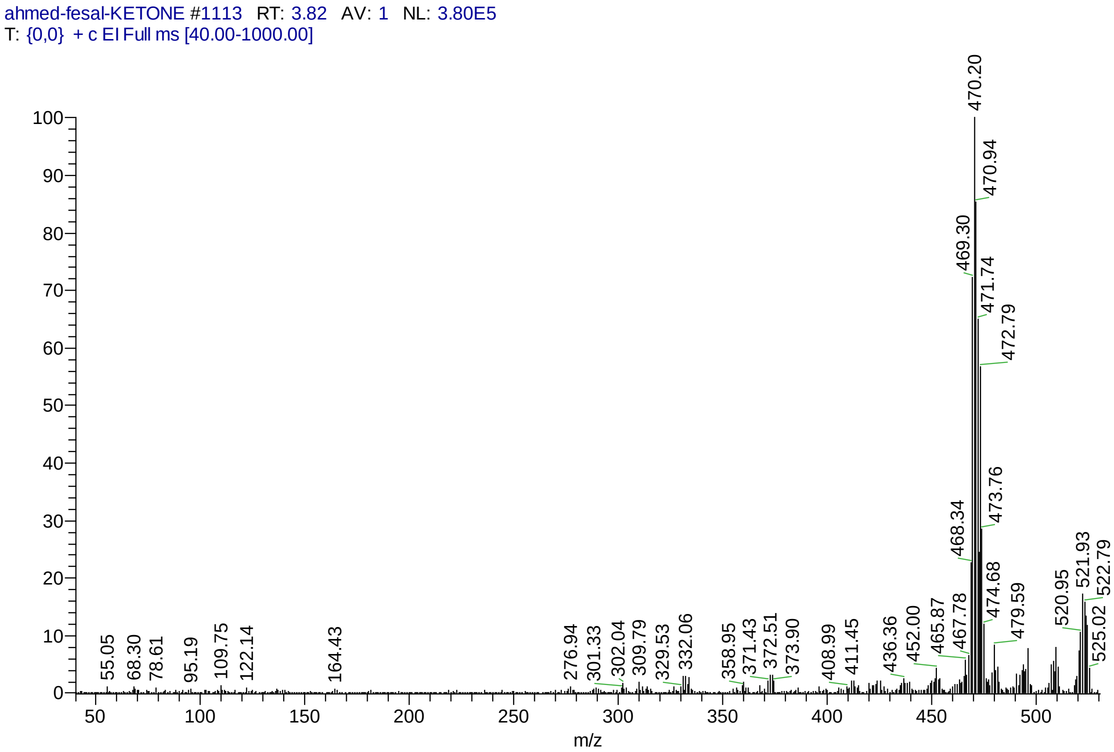

Figure S76. Mass spectrum of compound 8a

Information Classification: General

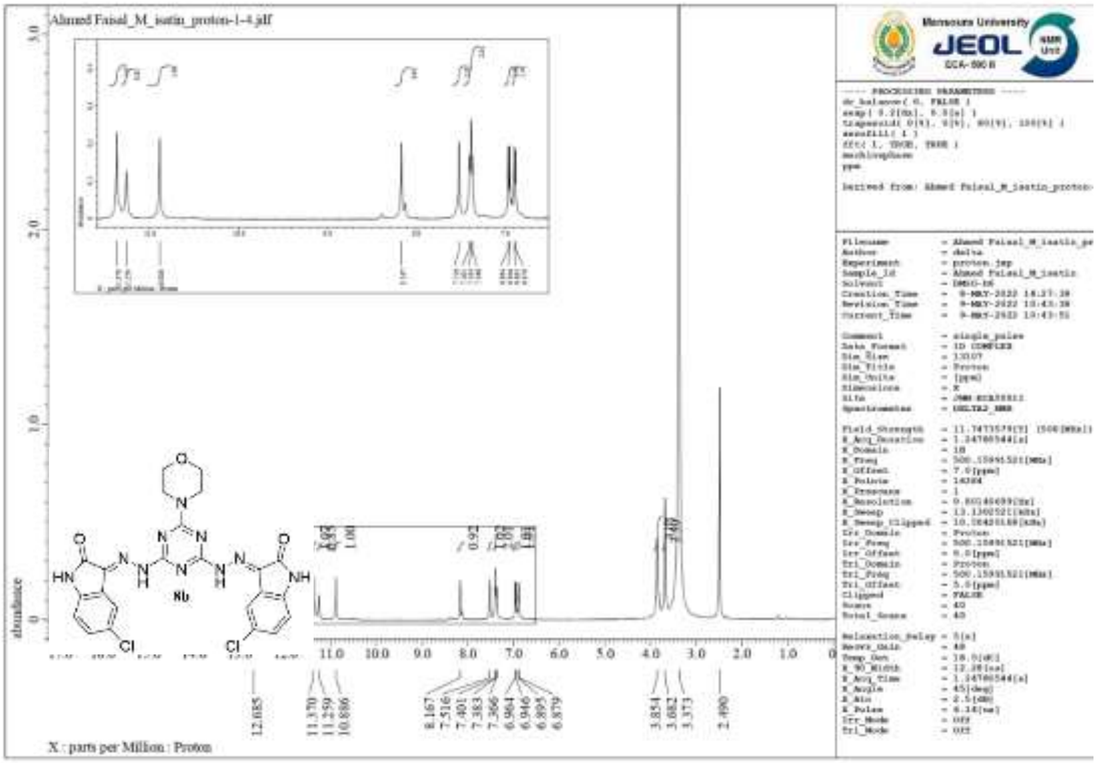

Figure S77. ^{1}**H NMR (500 MHz, DMSO-**d_6**) spectrum of compound 8b**

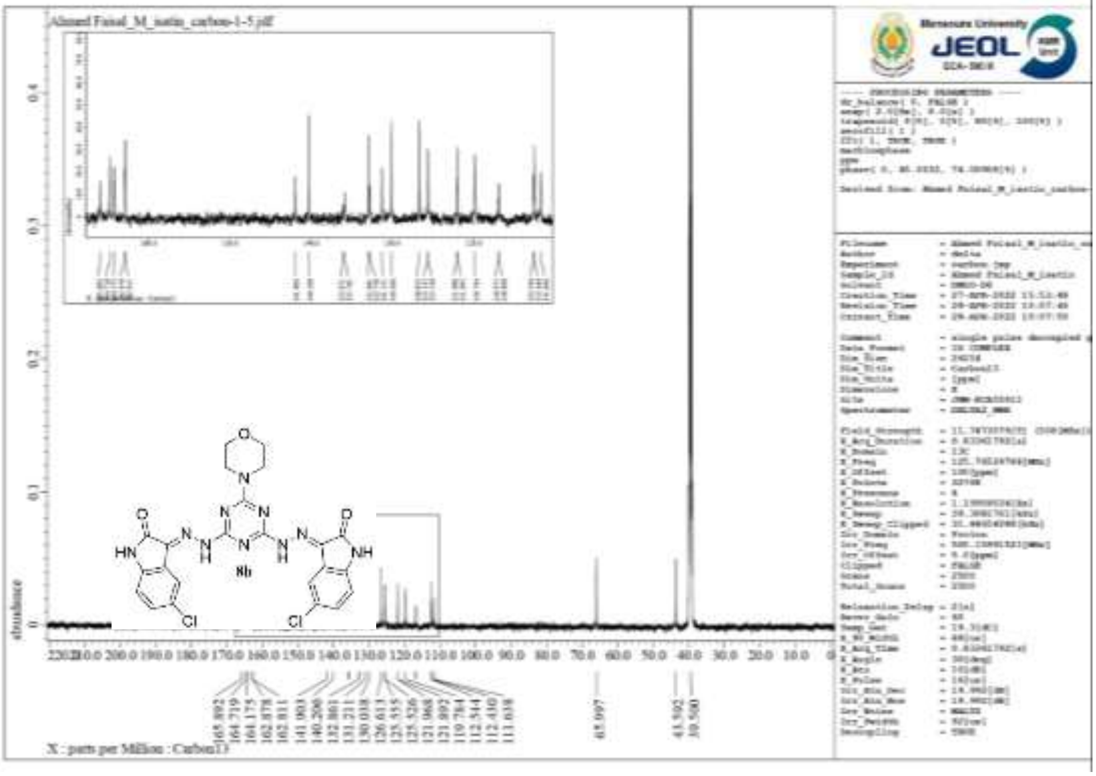

Figure S78. ^{13}**C NMR (125 MHz, DMSO) spectrum of compound 8b**

Information Classification: General

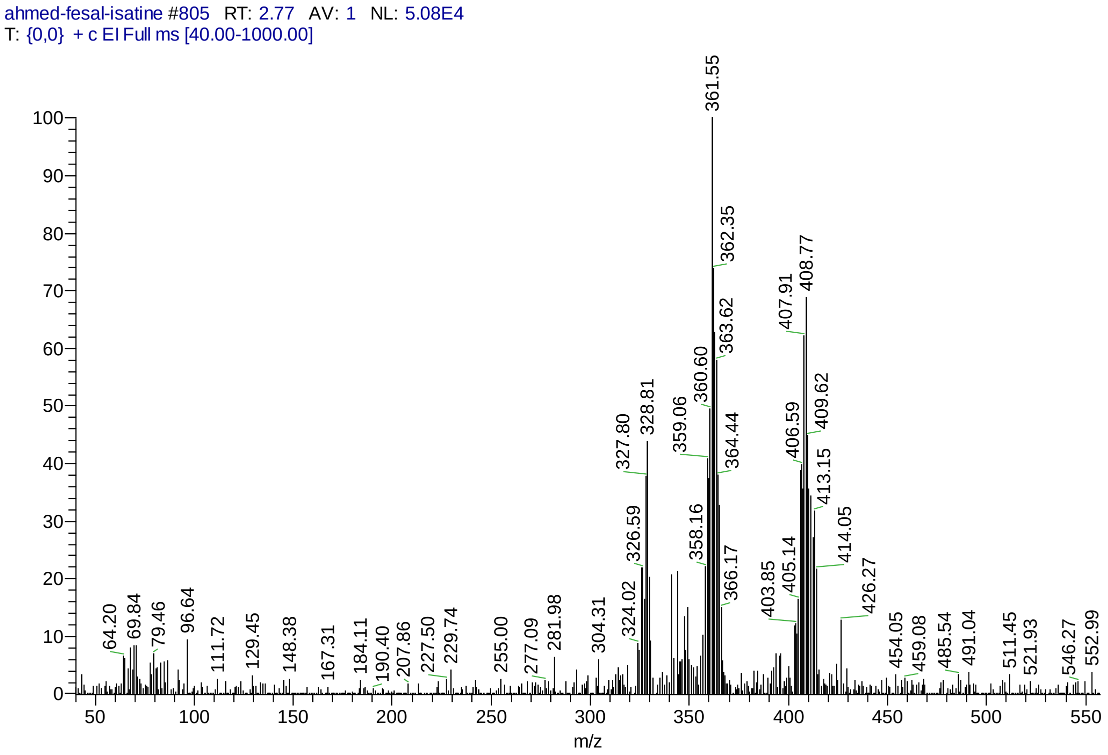

Figure S79. Mass spectrum of compound 8b

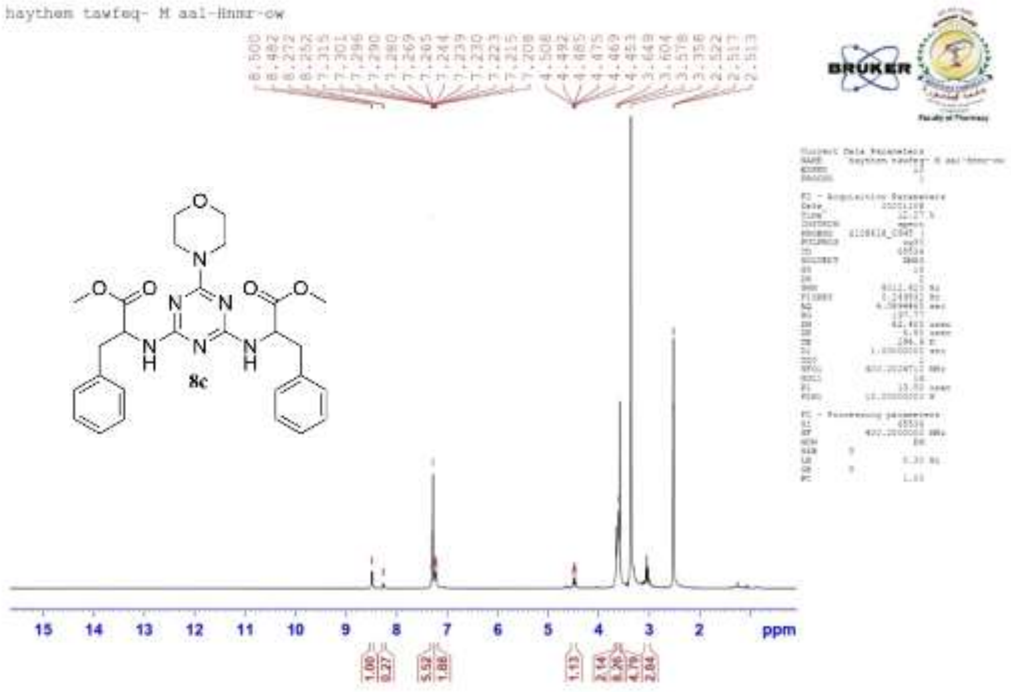

Figure S80. ^{1}H NMR (400 MHz, DMSO-d_6) spectrum of compound 8c

Information Classification: General

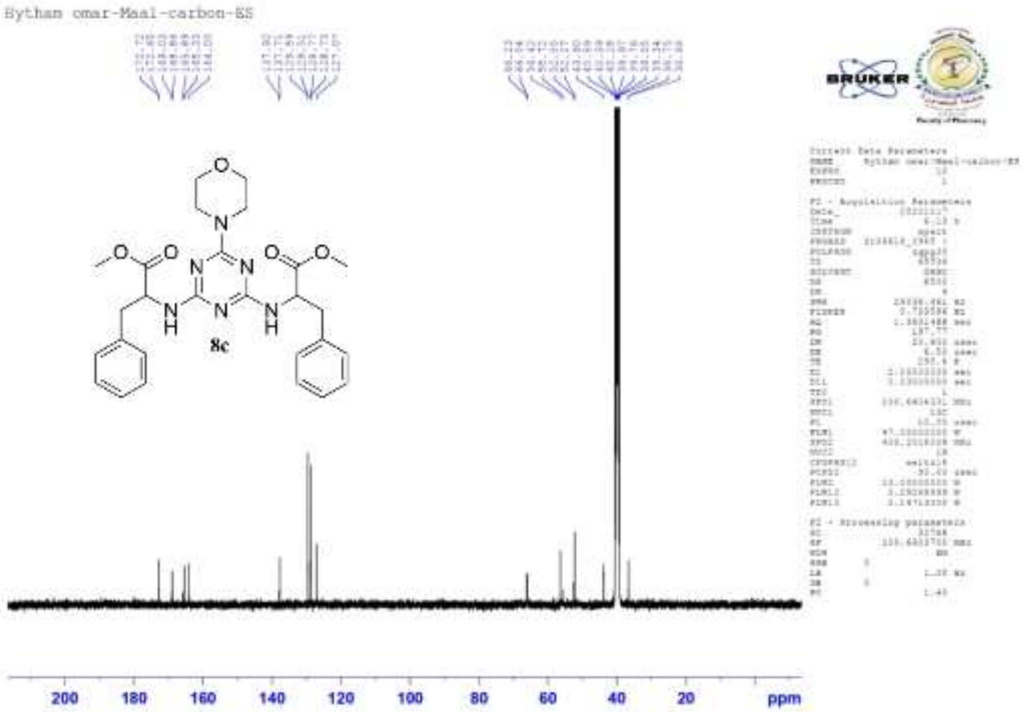

Figure S81. ^{13}C NMR (100 MHz, DMSO) spectrum of compound 8c

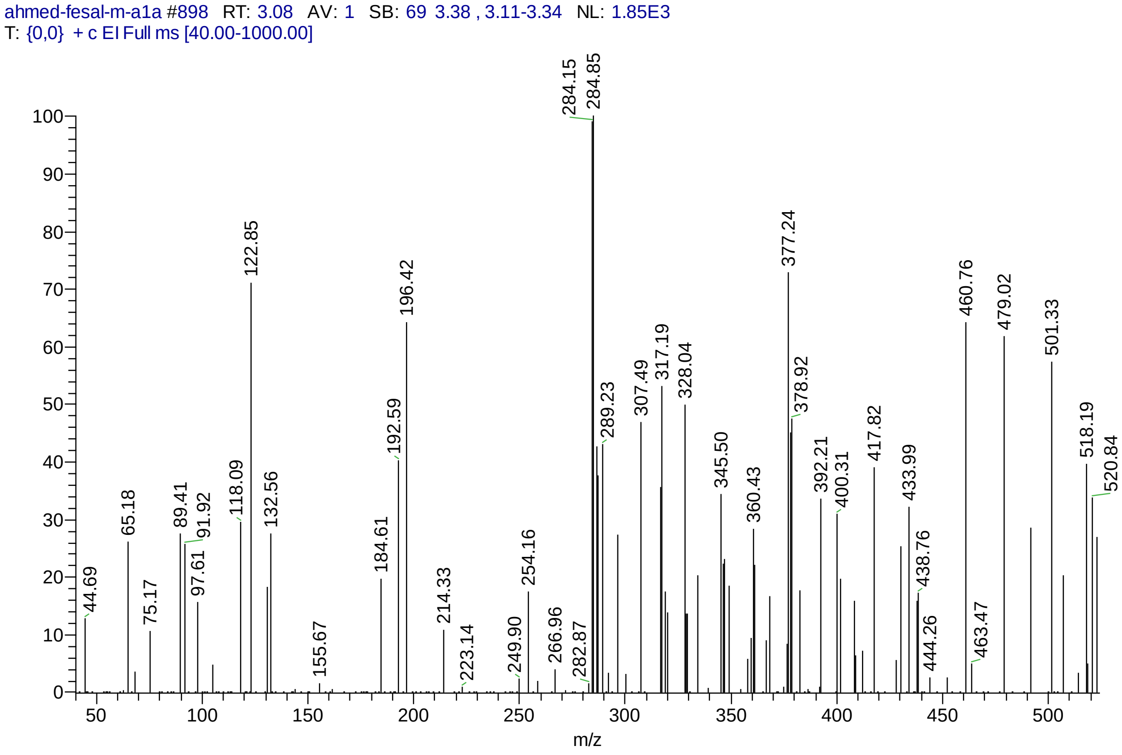

Figure S82. Mass spectrum of compound 8c

Information Classification: General

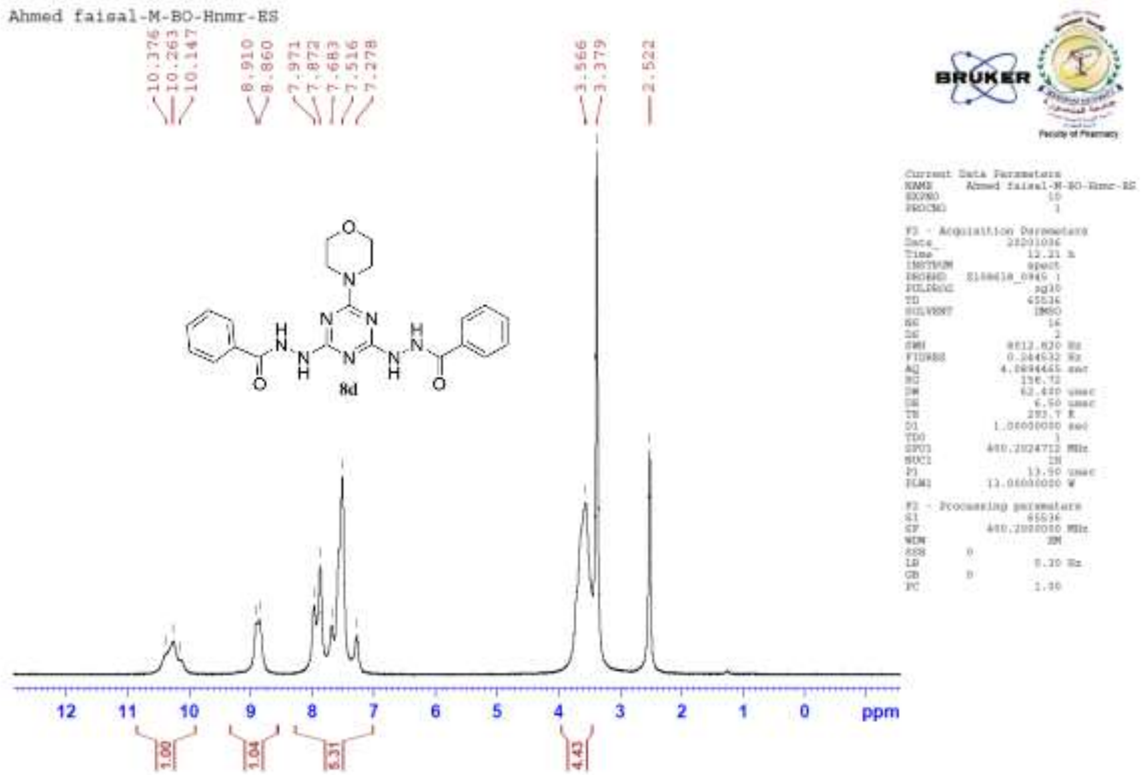

Figure S83. ^{1}H NMR (400 MHz, DMSO-d_6) spectrum of compound 8d

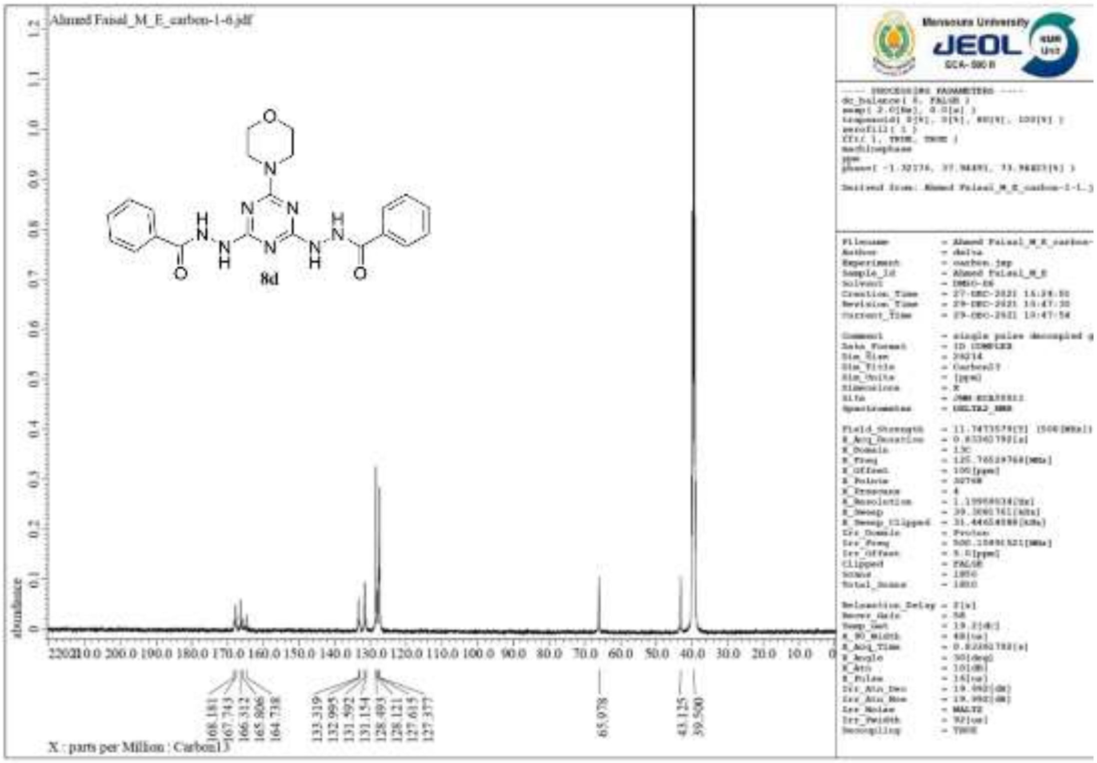

Figure S84. ^{13}C NMR (125 MHz, DMSO) spectrum of compound 8d

Information Classification: General

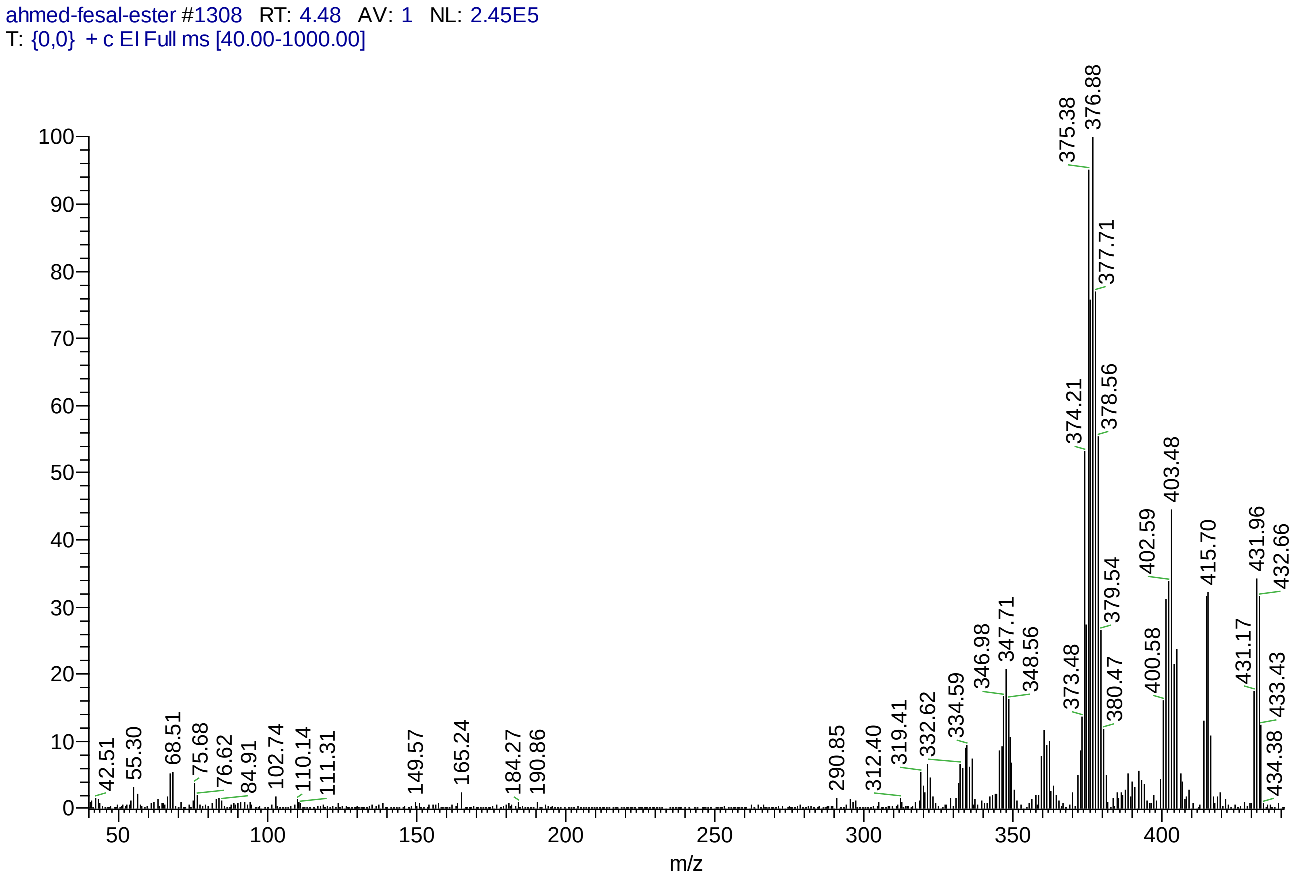

Figure S85. Mass spectrum of compound 8d

Information Classification: General

Table 1. Preliminary anticancer effects of single dose (10 μM) of target compounds in, series **(I) 6a-l** and series **(II) 7a-l, (III) 8a-d** against 60 human subpanel cancer cell lines declared as the percentage cell growth inhibition (GI%)

Subpanel cell lines	Growth Inhibition Percentage (GI %)									
	**6a**	**6b**	**6c**	**6d**	**6e**	**6f**	**6g**	**6h**	**6i**	**6j**
**Leukemia (L)**										
**CCRF-CEM**	57.65	19.92	79.05	75.18	32.77	58.42	86.74	16.46	12.24	-2.09
**HL-60(TB)**	96.26	19.54	110.2	109.74	50.94	47.75	3.26	-3.95	13.71	0.38
**K-562**	74.64	5.94	85.49	88.23	35.27	63.32	93.65	38.57	31.41	-5.6
**MOLT-4**	54.86	27.39	89.8	93.96	33.86	52.67	3.27	16.06	13.33	-1.68
**RPMI-8226**	46.36	35.02	65.83	61.19	33.05	66.38	65	12.12	42.76	-3.09
**SR**	84.03	36.14	92.07	92.30	60.18	66.01	113.07	51.85	18.46	8.3
**Non-Small Cell Lung Cancer (NSCLC)**										
**A549/ATCC**	87.04	2.89	57.4	53.38	6.5	28.63	25.12	3.4	-8.71	1.02
**EKVX**	77.82	19.76	50.98	27.82	10.87	11.01	4.62	24.44	0.07	5.31
**HOP-62**	74.28	-8.36	47.72	-9.83	-5.63	26.44	-5.52	-3.7	-5.02	-5.33
**HOP-92**	87.15	-12.31	36.22	34.55	8.68	14.19	-8.78	-5.67	-20.34	-7.35
**NCI-H226**	76.72	4.87	56.72	39.65	5.56	26.4	6.53	14.6	-3.53	1.59
NCI-H23	58.46	-4.52	56.93	29.37	-1.55	27.55	0.65	25.46	3.66	0.57
**NCI-H322M**	55.47	3.22	47.23	46.16	3.82	5.64	-0.33	11.87	2.28	9.4
**NCI-H460**	67.51	-2.92	58.82	21.79	5.44	16.97	83.28	-0.07	-1.58	-7.99
**NCI-H522**	39.06	13.39	66.45	86.08	4.23	34.8	1.08	21.4	0.32	3.1
**Colon Cancer (CC)**										
**COLO 205**	52.04	13.91	76.23	63.11	37.37	44.2	-8.37	7.98	-4.66	-7.11
**HCC-2998**	41.13	29.05	68.49	41.04	-2.3	-2.57	-2.9	-6.13	-5.78	-6.4
**HCT-116**	80.81	2.53	78.76	77.2	24.6	35.65	64.26	19.28	7.64	-6.01
**HCT-15**	96.13	18.94	108.94	111.07	52.5	53.17	77.62	15.24	8.38	-2.93
**HT29**	65.76	25.98	93.39	91.79	53.47	42.19	-1.91	12.87	-2.29	-12
**KM 12**	47.32	10.86	69.7	69.79	0.3	15.01	-3.9	8.19	6.79	-1.91
**SW-620**	61.5	3.31	75.3	81.91	33.97	18.44	16.24	-6.25	-6.94	-3.6
**CNS Cancer (CNSC)**										
**SF-268**	27.74	-2.5	31.58	34.13	4.91	6.36	2.19	12.99	0.39	-2.53
**SF-295**	80.42	14.47	70.86	42.35	-8.45	13.72	86.19	18.01	-7.07	-3.97
**SF-539**	48.19	-4.64	43.61	56.4	12.56	6.2	3.33	44.25	6.38	-3.18
**SNB-19**	58.32	-1.04	22.05	12.05	1.85	4.49	8.7	12.31	0.85	-4.37
**SNB-75**	104.09	-31.72	-7.5	-2.64	18.29	11.21	12.62	-19.58	-7.64	-1.54
**U251**	78.04	3.53	57.3	39.61	23.82	17.18	55.74	20.97	-5.95	-3.1
**Melanoma**										
**LOX IMVI**	95.96	-2.7	94.77	67.4	0.67	39.52	12.34	31.55	9.76	-5.85

Information Classification: General

Subpanel cell lines	Growth Inhibition Percentage (GI %)									
	**6a**	**6b**	**6c**	**6d**	**6e**	**6f**	**6g**	**6h**	**6i**	**6j**
**MALME-3M**	47.37	-42.88	153.96	143.74	-44.01	6.12	-14.15	2.72	-29.9	-4.04
**M14**	62.38	NT	68.29	64.84	100	14.02	-2.62	-4.08	-11.52	-4.69
**MDA-MB-435**	41.07	2.29	66.57	50.74	1.89	9.84	-4.43	-6.86	-7.52	-3.12
**SK-MEL-2**	27.99	3.71	97.36	101.89	2.51	4.93	1.45	13.82	-2.6	3.04
**SK-MEL-28**	71.96	0.72	82.88	70.18	10.12	17.85	-5.75	2.23	-4.35	-2.58
**SK-MEL-5**	34.2	30.36	79.26	55.87	27.89	36.91	-0.23	15.5	3.31	2.65
**UACC-257**	46.66	-3.93	64.12	60.86	6.59	9.17	-2.69	-9.57	-2.78	-3.12
**UACC-62**	11.18	-2.17	43.96	8.33	-5.24	21.08	2.25	11.35	9.59	2.19
**Ovarian Cancer**										
**IGROV1**	63	10.33	57.18	49.35	18.43	42.63	5.41	11.17	7.34	-6.08
**OVCAR-3**	66.39	14.84	57.55	43.71	19.31	19.02	-9.22	-2.72	-10	-4.79
**OVCAR-4**	88.25	3.79	45.82	33.6	0.54	18.4	-3.79	3.18	-12.46	-1.23
**OVCAR-5**	63.03	-11.22	27.6	4.53	-3.81	-16.96	-10.41	-10.24	-9.28	-3.8
**OVCAR-8**	87.26	3.39	62.58	50.51	8.56	39.91	1.04	44.56	3.42	-0.99
**NCI/ADR-RES**	77.06	8.51	58.24	38.19	8.29	30.89	0.17	34.18	-0.3	-5.32
**SK-OV-3**	88.13	-13.88	7.13	-29.62	-17.71	-28.96	-9.91	-15.13	-22.51	-7.04
**Renal Cancer**										
**786-0**	69.66	38.71	75.48	69.44	54.04	30.4	15.4	1.86	-8.76	-3.75
**A498**	NT	49.78	NT	95.69	51.99	13.11	NT	-10.47	-10.29	2.87
**ACHN**	71.9	6.36	73.69	74.23	20.86	30.16	-4.23	-7.98	-2.76	-1.32
**CAKI-1**	95.44	-8.66	84.64	62.4	11.29	29.52	4.03	-1.35	-3.02	0.69
**RXF 393**	100.65	30.35	168.01	82.55	79.3	28.15	6.9	5.06	-20.66	-20.44
**SN 12C**	72.81	1.16	55.8	40.07	8.98	24.45	10.78	20.19	9.02	0.16
**TK-10**	52.05	0.58	33.24	-0.21	-28.12	-45.74	-10.17	-29.23	-24.42	-9.62
**UO-31**	80.18	21.44	74.5	73.15	30.31	50.72	9.35	16.05	22.28	15.45
**Prostate Cancer**										
**PC-3**	75.9	16.01	63.47	58.52	24.5	37.85	12.32	14.52	-0.63	-5.8
**DU-145**	64.49	1.72	72.68	57.48	40.47	31.14	7.27	-2.63	-11.34	-5.37
**Breast Cancer**										
**MCF7**	59.97	14.77	77.94	57.05	19.26	34.71	45.15	25.08	56.71	5.79
**MDA-MB-231 /ATCC**	63.72	-3.30	33.87	28.72	3.49	2.27	0.47	13.77	3.16	1.27
**HS 578T**	53.04	-14.37	33.8	32.54	-0.4	16.7	2.43	-8.95	-5.27	-5.42
**BT-549**	23.67	-6.54	47.32	7.26	11.59	11.7	-5.53	0.16	-0.85	-9.12
**T-47D**	53.94	21.02	43.55	31.48	10.28	30.86	5.31	44.66	0.85	7.67

Information Classification: General

Subpanel cell lines	Growth Inhibition Percentage (GI %)									
	6a	6b	6c	6d	6e	6f	6g	6h	6i	6j
**MDA-MB-468**	60.84	9.74	98.91	69.24	11.54	1.3	2.17	32.81	27.06	6.49
**Mean GI %**	66.07	7.16	66.00	53.69	15.60	23.39	14.28	9.97	0.67	-2.12

Table 1. Preliminary anticancer effects of single dose (10 μM) of target compounds in, series **(I) 6a-l** and series **(II) 7a-l, (III) 8a-d** against 60 human subpanel cancer cell lines declared as the percentage cell growth inhibition (GI%, (Cont.)

Subpanel cell lines	Growth Inhibition Percentage (GI %)									
	6k	6l	7a	7b	7c	7d	7e	7f	7g	7h
Leukemia (L)										
**CCRF-CEM**	40.95	18.85	78.34	0.06	18.7	24.99	41.31	65.13	74.2	28.74
**HL-60(TB)**	61.08	26.15	52.57	-3.87	7.75	3.01	31.95	42.8	137.97	-6.32
**K-562**	45.61	42.96	61.84	4.67	11.15	16.97	38.56	67.63	96.92	21.88
**MOLT-4**	47.32	18.16	59.08	-12.28	31.6	31.33	40.44	57.24	87.95	15.65
**RPMI-8226**	40.29	41.11	57.37	-0.33	11.79	27.61	38.83	69.34	67.36	6.61
**SR**	85.03	27.82	60.18	-1.34	12.93	25.36	31.85	53.33	114.07	66.63
Non-Small Cell Lung Cancer (NSCLC)										
**A549/ATCC**	17.71	21.03	52.18	-2.25	10.57	13.69	10.55	39.29	21.55	44.36
**EKVX**	4.47	45.54	69.87	4.52	22.43	21.65	29.33	34.89	2.99	10.98
**HOP-62**	13.6	6.23	44.19	1.14	-8.46	-4.1	-7.48	41.53	18.25	1.81
**HOP-92**	8.47	26.96	21.61	-32.99	-12.84	-7.89	-16.04	20.94	16.02	13.8
**NCI-H226**	21.99	20.45	18.2	10.92	8.12	17.97	17.95	42.56	-1.93	2.51
NCI-H23	16.98	27.25	44.22	4.71	-5.83	2.24	9.62	37.81	3.27	0.28
**NCI-H322M**	11.76	16.23	41.08	3.65	-3.53	-1.33	-8.36	13.8	-0.12	10.02
**NCI-H460**	12.21	23.11	60.5	0.96	-2.41	3.48	8.63	31.28	29.08	66.25
**NCI-H522**	12.54	14.37	52.53	6.02	8.55	11.68	8.08	58.08	3.84	23.39
Colon Cancer (CC)										
**COLO 205**	40.7	29.62	75.48	-5.18	-5.18	-1.88	-0.08	24.11	16.53	9.72
**HCC-2998**	8.26	5.25	60.43	-7.41	-2.01	-8.18	0.77	5.6	-3.1	2.64
**HCT-116**	37.14	16.36	22.01	-2.25	13.47	2.75	9.65	46.98	85.13	51.22
**HCT-15**	41.52	22.08	22.55	1.2	16.1	20.19	29.19	53.55	99.88	34.49
**HT29**	51.69	30.63	54.71	-1.89	3.98	14.36	8.7	36.31	104.67	15.66
**KM 12**	17.4	18.91	52.48	2	-6.72	8.95	17.88	35.17	6.98	32.83
**SW-620**	29.02	9.73	32.18	-0.46	-8.28	-2.95	-7.5	16.52	35.82	57.44
CNS Cancer										
**SF-268**	8.94	11.15	45.67	-1.64	-5.8	8.03	0.36	24.27	-5.33	13.59
**SF-295**	9.09	49.51	47.41	-3.22	2.91	7.02	15.59	28.34	-6.74	7.34
**SF-539**	18.19	7.53	40.62	2.62	-6.24	-5.25	-3.16	7.37	112.27	15.69
**SNB-19**	5.57	12.9	44.05	3.76	-2.24	1.37	3.96	11.5	13.95	4.59
**SNB-75**	13.41	9.55	25.6	-2.35	-17.28	-2.48	-14.56	-0.71	-1.39	-4.05

Information Classification: General

Subpanel cell lines	Growth Inhibition Percentage (GI %)									
	**6k**	**6l**	**7a**	**7b**	**7c**	**7d**	**7e**	**7f**	**7g**	**7h**
**U251**	24.06	11.8	49.59	-2.15	3.1	8.35	5.46	28.63	49.97	47.15
**Melanoma**										
**LOX IMVI**	30.12	5.3	60.31	1.94	-5.74	-4.97	14.22	49.07	173.29	31.71
**MALME-3M**	-3.77	16.01	33.08	-6.25	15.07	-9.06	-0.5	7.94	167.33	7.49
**M14**	5.09	27.79	53.57	-4.12	NT	4.75	NT	41.23	27.17	31.19
**MDA-MB-435**	4.07	19.39	30.87	-1.23	4.57	9.61	8.23	26.05	39.61	-13.78
**SK-MEL-2**	1.68	17.11	30.78	-1.28	-2.13	13.92	0.55	36.03	-10.79	2.27
**SK-MEL-28**	12.83	10.03	36.56	-0.81	0.82	3.79	9.32	31.36	58.34	5.78
**SK-MEL-5**	14.01	44.91	25.6	5.12	28.57	19.35	32.15	52.38	13.27	0.02
**UACC-257**	-2.23	17	20.85	-8.22	-4.98	-11.08	-0.08	4.25	-4.45	10.25
**UACC-62**	-4.28	19.83	29.72	12.24	14.54	20.83	25.76	43.66	-0.61	31.71
**Ovarian Cancer**										
**IGROV1**	24.3	41.44	54.81	2.38	-0.7	-4.79	9.61	51.28	6.23	20.37
**OVCAR-3**	6.73	22.93	80.77	-6.1	0.95	8.59	10.61	44.38	34.42	91.81
**OVCAR-4**	8.16	32.75	70.94	0.53	11.63	9.83	13.72	32.27	-6.05	28.55
**OVCAR-5**	-1.78	-12.43	28.44	0.1	-15.89	-10.44	-8.1	-12.99	3.87	6.05
**OVCAR-8**	26.04	15.51	50.41	-1.21	2.62	10.79	14.62	48.92	43.4	27.07
**NCI/ADR-RES**	23.29	16.83	80.97	-2.41	9.29	4.8	23.85	32.83	41.7	32.64
**SK-OV-3**	-11.76	7.59	12.04	-0.76	-9	-9.25	-14.55	-14.33	-31.8	-5.17
**Renal Cancer**										
**786-0**	36.61	3.32	26.78	1.09	4.36	4.76	10.52	-6.21	11.81	-1.71
**A498**	NT	26.93	17.02		12.24	7.8	15.71	18.67	-15.11	7.46
**ACHN**	24.7	7.67	63.13	4.56	-5.5	-1.44	8.6	37.83	67.57	16.3
**CAKI-1**	21.13	17.48	24.47	3.19	0.79	7.63	9.66	27.61	10.06	15.86
**RXF 393**	30.47	17.81	16.96	2.13	-16.12	6.56	-12.51	-3.98	6.71	-11.79
**SN 12C**	24.14	15.33	40.48	5.69	-1.41	7.63	5.08	31.93	49.57	19.31
**TK-10**	-3.24	-16.51	2.29	-16.5	-27.65	-6.48	-20.65	-9.28	-18.38	8.78
**UO-31**	46.35	33.49	51.67	7.3	20.22	23.25	22.11	48.38	19.69	6.76
**Prostate Cancer**										
**PC-3**	30.13	19.26	55.25	5.07	19.75	24.03	31.37	51.41	15.19	29.94
**DU-145**	23.91	4.76	45.22	-4.47	-1.2	5.38	2.92	29.76	22.48	26.65
**Breast Cancer**										
**MCF7**	30.94	28.98	64.28	13.21	20.24	16.93	26.71	38.72	68.89	28.84
**MDA-MB-231 /ATCC**	0.11	10.73	34.54	2.05	-8.36	-16.08	-0.43	0.87	31.42	15.8
**HS 578T**	16.69	5.00	23.41	-5.25	-21.18	-4.65	-13.24	20.78	-6.05	14.98
**BT-549**	5.57	-1.45	NT	-5.26	-13.14	5.13	-5.92	28.23	-10.63	2.70
**T-47D**	23.77	29.48	51.76	11.2	12.30	24.04	30.64	47.48	53.97	51.71
**MDA-MB-468**	32.09	24.58	36.2	12.37	9.99	23.66	9.17	26.39	63.73	7.19

Information Classification: General

Subpanel cell lines	Growth Inhibition Percentage (GI %)									
	6k	6l	7a	7b	7c	7d	7e	7f	7g	7h
**Mean GI %**	20.69	19.00	45.09	-0.12	2.56	7.03	10.18	30.94	35.10	18.81

Table 1. Preliminary anticancer effects of single dose (10 μM) of target compounds in, series **(I) 6a-l** and series **(II) 7a-l,** series **(III) 8a-d** against 60 human subpanel cancer cell lines declared as the percentage cell growth inhibition (GI%, (Cont.)

Subpanel cell lines	Growth Inhibition Percentage (GI %)							
	7i	7j	7k	7l	8a	8b	8c	8d
**Leukemia (L)**								
**CCRF-CEM**	38.67	9.46	79.63	77.73	38.39	79.75	-4.54	-6.05
**HL-60(TB)**	58.10	-1.96	39.56	98.73	7.40	27.78	-2.51	-15.29
**K-562**	68.28	6.41	58.85	67.73	20.31	67.14	8.42	-6.16
**MOLT-4**	57.66	6.30	43.66	86.29	21.09	37.48	-1.03	-8.90
**RPMI-8226**	81.72	-7.60	43.82	96.73	35.26	63.6	1.47	-9.42
**SR**	36.83	25.85	99.89	83.92	-13.1	70.79	-0.37	2.07
**Non-Small Cell Lung Cancer (NSCLC)**								
**A549/ATCC**	-11.2	-0.07	8.56	78.36	26.13	-1.37	-8.33	1.66
**EKVX**	22.00	1.58	6.27	86.19	31.88	14.84	-4.54	3.90
**HOP-62**	56.32	-10.50	-22.53	74.13	13.83	62.44	2.66	-11.84
**HOP-92**	-23.07	-9.47	4.01	101.32	35.93	73.99	-18.54	-12.82
**NCI-H226**	38.57	4.47	-0.61	46.14	58.10	42.69	-1.61	4.42
NCI-H23	18.20	0.50	-1.44	55.17	42.14	82.84	-6.51	5.69
**NCI-H322M**	28.75	-2.30	1.53	64.24	13.28	21.94	2.57	4.42
**NCI-H460**	1.33	0.77	9.41	80.13	21.87	11.84	-6.88	-1.86
**NCI-H522**	48.08	6.61	8.07	89.64	38.34	94.91	9.02	-2.83
**Colon Cancer (CC)**								
**COLO 205**	-0.27	-6.39	14.12	116.72	5.21	51.30	-4.56	-12.92
**HCC-2998**	32.58	-5.00	16.23	75.65	3.95	24.46	-9.86	-5.88
**HCT-116**	78.03	23.74	52.1	80.49	29.43	54.64	0.81	-3.34
**HCT-15**	50.33	16.09	66.19	96.06	33.90	8.40	-6.16	-2.67
**HT29**	92.57	-13.41	61.82	96.50	19.45	8.90	-0.61	-12.01
**KM 12**	94.55	-0.29	13.82	89.24	17.85	18.8	0.30	-5.40
**SW-620**	36.29	6.35	17.59	82.81	4.48	25.67	-11.66	-4.44
**CNS Cancer**								
**SF-268**	35.13	-2.56	-0.46	63.34	3.96	50.89	-2.56	-1.5
**SF-295**	6.78	0.36	22.23	97.43	33.5	54.35	-7.74	-0.06
**SF-539**	63.69	1.16	12.44	43.75	84.53	83.97	1.99	2.56
**SNB-19**	27.44	13.7	19.73	74.23	36.94	109.43	-3.58	3.32
**SNB-75**	-36.06	-4.00	-13.08	62.51	19.38	100.67	9.51	9.11
**U251**	81.5	9.07	18.64	73.21	7.36	96.19	-5.23	-0.30
**Melanoma**								
**LOX IMVI**	34.01	6.65	52.13	80.37	61.08	76.78	-5.03	2.21

Information Classification: General

Subpanel cell lines	Growth Inhibition Percentage (GI %)							
	7i	7j	7k	7l	8a	8b	8c	8d
**MALME-3M**	-1.47	6.88	-20.19	95.81	30.63	43.58	-4.87	-7.11
**M14**	7.70	-3.18	2.72	88.1	25.54	22.51	NT	-0.8
**MDA-MB-435**	19.28	-1.68	1.52	96.25	14.99	36.49	-4.67	-2.63
**SK-MEL-2**	37.80	9.62	-2.84	125.78	3.61	34.71	-10.96	-13.42
**SK-MEL-28**	22.22	-7.04	-8.67	55.11	16.23	44.95	-8.72	-19.49
**SK-MEL-5**	48.87	4.85	3.15	40.95	32.89	53.94	1.32	0.18
**UACC-257**	56.87	-8.89	-7.99	88.28	0.81	18.02	-3.35	1.13
**UACC-62**	34.01	3.56	-2.37	81.67	14.73	48.43	9.82	4.98
**Ovarian Cancer**								
**IGROV1**	47.25	5.35	23.14	82.32	6.22	45.01	1.38	-2.38
**OVCAR-3**	107.45	5.01	-5.21	77.35	11.56	47.29	-11.08	-5.45
**OVCAR-4**	13.14	-0.74	-9.55	78.95	22.39	21.34	-6.53	-10.62
**OVCAR-5**	-8.09	-13.14	-18.17	72.15	4.46	4.93	-6.25	-4.08
**OVCAR-8**	68.96	1.94	9.89	74.19	36.36	49.91	0.4	3.15
**NCI/ADR-RES**	69.76	-3.16	14.52	84.77	31.31	1.85	-10.56	1.24
**SK-OV-3**	1.24	-24.79	-39.55	60.86	-12.96	44.55	-2.71	-18.5
**Renal Cancer**								
**786-0**	-8.84	-1.22	13.36	71.74	33.09	26.29	-0.43	-3.15
**A498**	-8.06	-2.84	48.28	47.2	33.87	78.37	-7.4	-10.99
**ACHN**	64.84	6.6	5.09	95.79	22.14	25.48	-8.95	-4.4
**CAKI-1**	27.58	13.4	1.07	85.87	30.84	27.98	13.22	3.15
**RXF 393**	0.13	-17.83	20.31	96.62	26.26	86.34	-12.71	0.99
**SN 12C**	27.66	4.32	20.28	76.81	-27.38	56.08	-3.62	-6.53
**TK-10**	6.2	-3.52	-20.99	72.77	19.22	-18.46	-3.4	-10.76
**UO-31**	52.54	15.28	25.61	90.98	NT	21.35	18.78	13.35
**Prostate Cancer**								
**PC-3**	46.6	9.42	26.35	81.33	23.41	43.85	-2.72	-7.68
**DU-145**	70.38	0.25	0.19	39.03	19	11.63	-11.6	-7.98
**Breast Cancer**								
**MCF7**	99.09	4.27	45.19	96.9	25.91	56.91	2.21	0.71
**MDA-MB-231 /ATCC**	51.67	12.75	9.3	57.89	56.54	63.69	-2.54	-3.73
**HS 578T**	46.16	-6.08	-4.09	66.74	21.34	107.82	-1.3	-6.14
**BT-549**	2.15	1.47	-21.87	31.62	32.71	106.36	-11.67	-9.45
**T-47D**	51.82	16.88	12.79	89.05	45.11	70.65	-0.53	-10.83
**MDA-MB-468**	31.23	10.15	18.37	114.15	20.35	76.37	-7.75	-15.09
**Mean GI %**	37.38	1.89	14.53	81.15	23.27	47.89	-2.92	-3.94

NT; Not tested. GI % less than 10 indicates low activity, GI % 10:60 indicates moderate activity, GI % 60:100 indicates strong activity, GI % more than 100 indicates very strong activity (lethal).

Information Classification: General

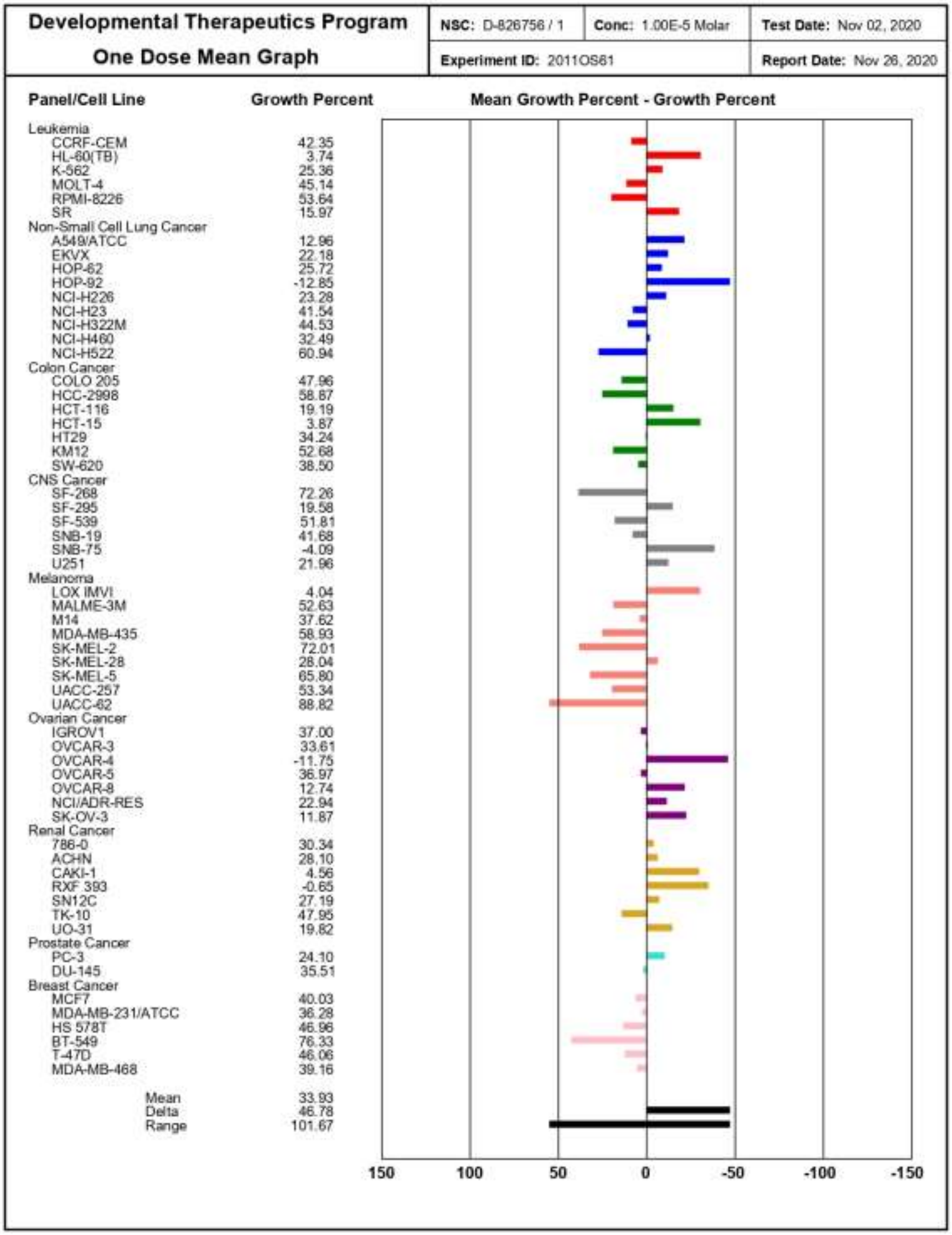

Figure S86. One dose mean graph for compound 6a (NSC 826756) at 10 µM

Information Classification: General

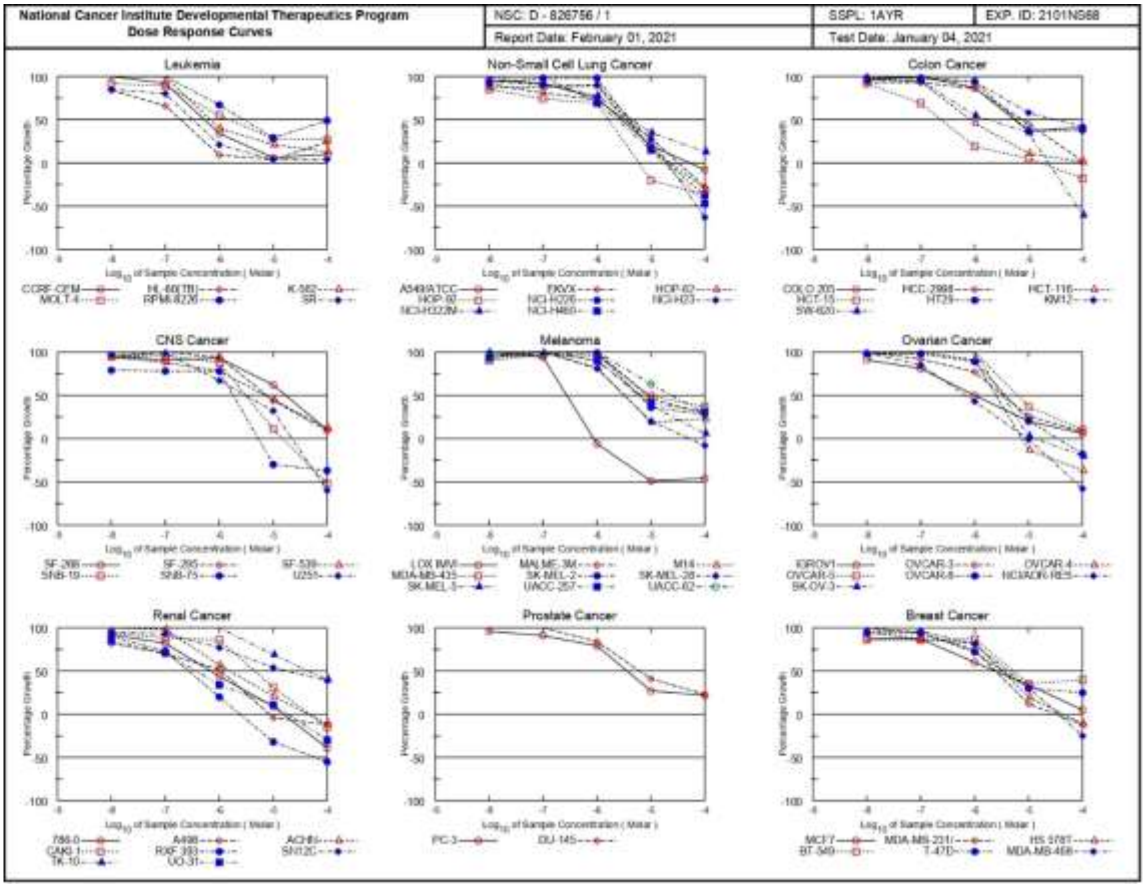

Figure S87. Dose-response curves (% growth versus sample concentration) for all cell lines with different subpanel obtained from the NCI's *in vitro* disease-oriented human cancer cells line for compound 6a on nine types of cancer

Information Classification: General

National Cancer Institute Developmental Therapeutics Program
In-Vitro Testing Results

NSC : D - 826756 / 1	Experiment ID : 2101NS68	Test Type : 08	Units : Molar
Report Date : February 01, 2021	Test Date : January 04, 2021	QNS :	MC :
COMI : AF_3	Stain Reagent : SRB Dual-Pass Related	SSPL : 1AYR	

			Log10 Concentration													
	Time		Mean Optical Densities					Percent Growth								
Panel/Cell Line	Zero	Ctrl	-8.0	-7.0	-6.0	-5.0	-4.0	-8.0	-7.0	-6.0	-5.0	-4.0	GI50	TGI	LC50
**Leukemia**															
CCRF-CEM	0.488	1.736	1.735	1.637	0.918	0.562	0.615	100	92	34	6	10	5.37E-7	> 1.00E-4	> 1.00E-4
HL-60(TB)	0.693	2.300	2.045	1.761	0.831	0.753	1.082	84	66	9	4	24	1.93E-7	> 1.00E-4	> 1.00E-4
K-562	0.282	2.154	2.176	2.154	1.033	0.678	0.520	101	100	40	21	13	6.83E-7	> 1.00E-4	> 1.00E-4
MOLT-4	0.721	2.487	2.354	2.294	1.695	1.220	1.201	92	89	55	28	27	1.55E-6	> 1.00E-4	> 1.00E-4
RPMI-8226	1.118	2.257	2.298	2.266	1.882	1.445	1.679	104	101	67	29	49	2.78E-6	> 1.00E-4	> 1.00E-4
SR	0.683	2.653	2.367	2.251	1.103	0.766	0.764	85	80	21	4	4	3.22E-7	> 1.00E-4	> 1.00E-4
**Non-Small Cell Lung Cancer**															
A549/ATCC	0.420	2.550	2.468	2.379	2.022	0.853	0.387	96	92	75	20	-8	2.88E-6	5.22E-5	> 1.00E-4
EKVX	0.568	1.544	1.455	1.356	1.278	0.848	0.409	91	81	73	29	-28	3.28E-6	3.20E-5	> 1.00E-4
HOP-62	0.895	2.655	2.435	2.452	2.470	1.195	0.626	87	88	89	17	-30	3.51E-6	2.30E-5	> 1.00E-4
HOP-92	1.491	2.121	2.029	1.956	1.928	1.198	0.935	85	74	69	-20	-37	1.65E-6	6.01E-6	> 1.00E-4
NCI-H226	1.047	2.229	2.129	2.210	2.206	1.250	0.646	92	98	98	17	-38	3.92E-6	2.04E-5	> 1.00E-4
NCI-H23	0.661	1.783	1.725	1.673	1.666	0.966	0.246	95	90	90	27	-63	4.31E-6	2.01E-5	7.21E-5
NCI-H322M	0.767	2.223	2.223	2.098	1.909	1.280	0.955	100	91	78	35	13	4.55E-6	> 1.00E-4	> 1.00E-4
NCI-H460	0.296	2.569	2.594	2.560	1.884	0.635	0.158	101	100	70	15	-47	2.30E-6	1.74E-5	> 1.00E-4
**Colon Cancer**															
COLO 205	0.637	2.661	2.590	2.659	2.391	1.393	1.463	96	100	87	37	41	5.53E-6	> 1.00E-4	> 1.00E-4
HCC-2998	0.633	1.904	1.819	1.815	1.720	1.209	0.657	93	93	86	45	2	7.65E-6	> 1.00E-4	> 1.00E-4
HCT-116	0.335	2.631	2.574	2.487	1.391	0.595	0.354	98	94	46	11	1	8.23E-7	> 1.00E-4	> 1.00E-4
HCT-15	0.296	1.904	1.772	1.404	0.608	0.372	0.244	92	69	19	5	-18	2.41E-7	1.62E-5	> 1.00E-4
HT29	0.442	2.648	2.671	2.581	2.508	1.258	1.288	101	97	94	37	38	5.89E-6	> 1.00E-4	> 1.00E-4
KM12	0.854	3.296	3.284	3.273	3.145	2.282	1.885	99	99	94	58	42	3.32E-5	> 1.00E-4	> 1.00E-4
SW-620	0.321	1.876	1.807	1.800	1.174	0.873	0.127	96	95	55	35	-60	1.78E-6	2.34E-5	7.78E-5
**CNS Cancer**															
SF-268	0.964	2.661	2.584	2.515	2.539	2.015	1.133	95	91	93	62	10	1.69E-5	> 1.00E-4	> 1.00E-4
SF-295	0.980	3.160	3.028	3.203	3.022	1.948	1.173	94	102	94	44	9	7.67E-6	> 1.00E-4	> 1.00E-4
SF-539	1.014	2.847	2.739	2.624	2.449	1.848	1.237	94	88	78	45	12	7.29E-6	> 1.00E-4	> 1.00E-4
SNB-19	0.546	1.880	1.809	1.829	1.726	0.694	0.260	95	96	88	11	-52	3.14E-6	1.49E-5	9.14E-5
SNB-75	1.164	2.086	1.889	1.881	1.885	0.812	0.737	79	78	78	-30	-37	1.82E-6	5.26E-6	> 1.00E-4
U251	0.429	2.088	2.044	2.077	1.545	0.956	0.173	97	99	67	32	-60	3.06E-6	2.23E-5	7.84E-5
**Melanoma**															
LOX IMVI	0.305	1.733	1.733	1.650	0.287	0.156	0.166	100	94	-6	-49	-46	2.76E-7	8.73E-7	> 1.00E-4
MALME-3M	0.832	2.318	2.328	2.432	2.403	1.521	1.259	101	108	106	46	29	8.69E-6	> 1.00E-4	> 1.00E-4
M14	0.545	1.952	1.858	1.988	1.704	0.808	0.875	93	103	82	19	23	3.22E-6	> 1.00E-4	> 1.00E-4
MDA-MB-435	0.575	2.548	2.342	2.487	2.465	1.542	1.300	90	97	96	49	37	9.53E-6	> 1.00E-4	> 1.00E-4
SK-MEL-2	1.298	2.532	2.464	2.544	2.524	1.746	1.646	95	101	99	36	28	6.06E-6	> 1.00E-4	> 1.00E-4
SK-MEL-28	0.553	1.714	1.702	1.749	1.493	0.781	0.506	99	103	81	20	-8	3.20E-6	4.99E-5	> 1.00E-4
SK-MEL-5	1.089	3.124	3.096	3.103	2.940	1.855	1.216	99	99	91	38	6	5.86E-6	> 1.00E-4	> 1.00E-4
UACC-257	1.013	2.514	2.415	2.531	2.362	1.628	1.486	93	101	90	41	31	6.53E-6	> 1.00E-4	> 1.00E-4
UACC-62	0.918	2.698	2.600	2.651	2.826	2.045	1.435	95	97	107	63	29	2.45E-5	> 1.00E-4	> 1.00E-4
**Ovarian Cancer**															
IGROV1	0.342	1.965	1.817	1.651	1.146	0.668	0.439	91	81	50	20	6	9.66E-7	> 1.00E-4	> 1.00E-4
OVCAR-3	0.626	1.974	2.039	1.860	1.669	0.979	0.731	105	92	77	26	8	3.42E-6	> 1.00E-4	> 1.00E-4
OVCAR-4	0.761	2.035	2.074	2.081	1.917	0.666	0.482	103	104	91	-13	-37	2.48E-6	7.56E-6	> 1.00E-4
OVCAR-5	0.491	1.409	1.415	1.459	1.447	0.831	0.584	101	105	104	37	10	6.41E-6	> 1.00E-4	> 1.00E-4
OVCAR-8	0.738	3.073	3.023	3.034	2.812	1.234	0.604	98	98	89	21	-18	3.75E-6	3.45E-5	> 1.00E-4
NCI/ADR-RES	0.514	1.682	1.676	1.503	1.019	0.508	0.215	99	85	43	-1	-58	6.85E-7	9.37E-6	7.18E-5
SK-OV-3	1.344	2.487	2.455	2.514	2.618	1.382	1.072	97	102	111	3	-20	3.70E-6	1.38E-5	> 1.00E-4
**Renal Cancer**															
786-0	0.678	2.678	2.516	2.320	1.566	0.854	0.415	92	82	44	9	-39	7.09E-7	1.53E-5	> 1.00E-4
A498	1.770	2.491	2.361	2.275	2.146	1.704	1.560	82	70	52	-4	-12	1.09E-6	8.58E-6	> 1.00E-4
ACHN	0.474	2.012	2.040	1.985	1.355	0.796	0.421	102	98	57	21	-11	1.58E-6	4.49E-5	> 1.00E-4
CAKI-1	0.918	2.959	2.835	2.714	2.676	1.551	0.777	94	88	86	31	-15	4.52E-6	4.65E-5	> 1.00E-4
RXF 393	1.070	1.479	1.438	1.368	1.153	0.732	0.487	90	73	20	-32	-55	2.72E-7	2.45E-6	6.35E-5
SN12C	1.323	3.329	3.231	3.179	2.863	2.408	2.096	95	93	77	54	39	1.83E-5	> 1.00E-4	> 1.00E-4
TK-10	1.107	1.958	1.988	2.233	2.109	1.694	1.447	103	132	118	69	40	4.49E-5	> 1.00E-4	> 1.00E-4
UO-31	0.716	2.403	2.147	1.916	1.292	0.903	0.504	85	71	34	11	-30	3.72E-7	1.87E-5	> 1.00E-4
**Prostate Cancer**															
PC-3	0.835	2.408	2.348	2.272	2.079	1.260	1.184	96	91	79	27	22	3.62E-6	> 1.00E-4	> 1.00E-4
DU-145	0.453	1.811	1.934	1.840	1.589	1.007	0.765	109	102	84	41	23	6.09E-6	> 1.00E-4	> 1.00E-4
**Breast Cancer**															
MCF7	0.480	2.280	2.062	2.051	1.554	1.095	0.569	88	87	60	34	5	2.39E-6	> 1.00E-4	> 1.00E-4
MDA-MB-231/ATCC	0.685	1.479	1.478	1.435	1.271	0.780	0.600	100	94	74	12	-12	2.42E-6	3.09E-5	> 1.00E-4
HS 578T	0.817	1.770	1.724	1.641	1.596	1.004	0.729	95	87	82	20	-11	3.24E-6	4.41E-5	> 1.00E-4
BT-549	1.257	2.136	2.012	2.010	2.020	1.568	1.610	86	86	87	35	40	5.19E-6	> 1.00E-4	> 1.00E-4
T-47D	1.008	2.519	2.428	2.433	2.099	1.458	1.393	94	94	72	30	25	3.34E-6	> 1.00E-4	> 1.00E-4
MDA-MB-468	0.703	1.761	1.721	1.720	1.559	1.027	0.531	96	96	81	31	-25	4.11E-6	3.59E-5	> 1.00E-4

Figure S88. Values of log molar concentration of response parameters (log_{10} GI_{50}, log_{10} TGI & log_{10} LC_{50}) for compound 6a

Information Classification: General

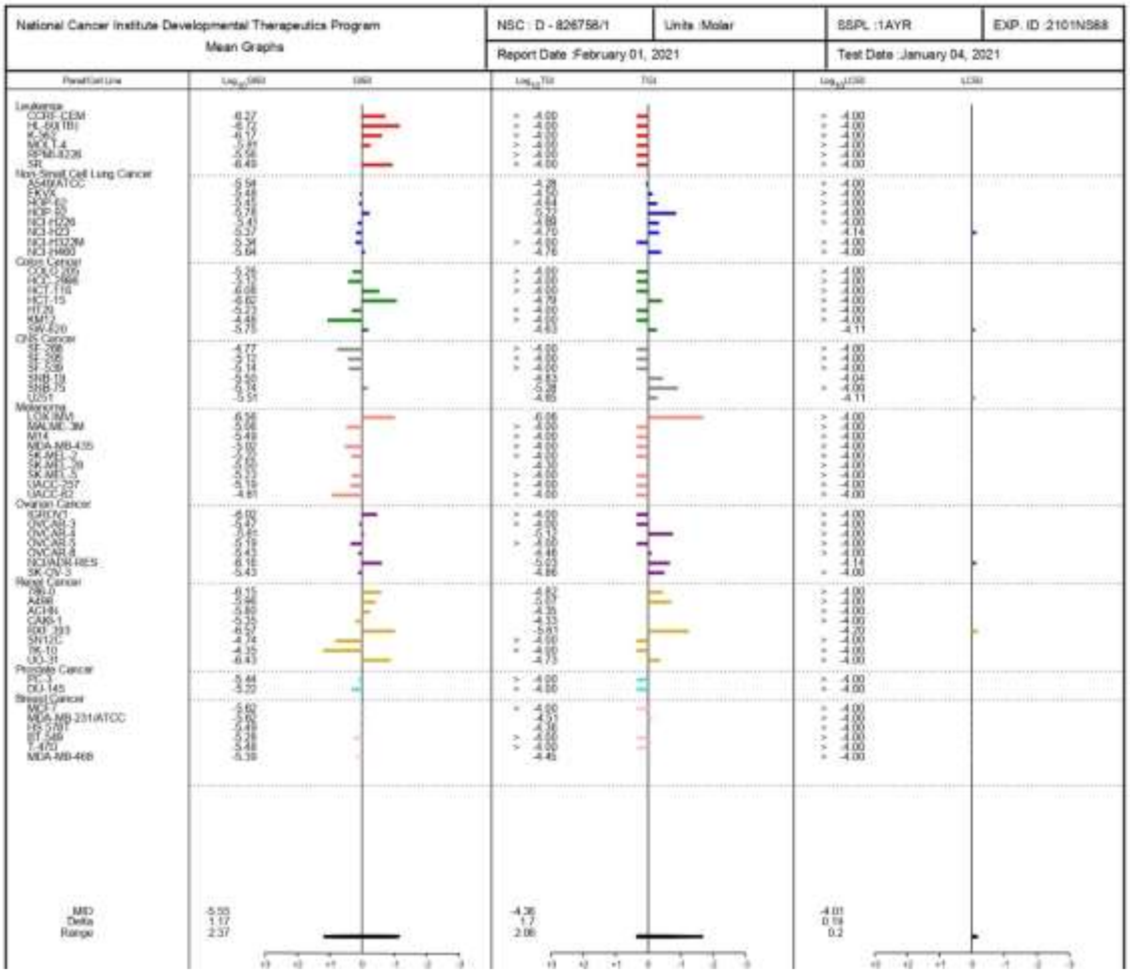

Figure S89. Mean Graphs of the log_{10} values (Molar) of GI_{50}, TGI, and LC_{50} obtained from the NCI 60 cell line experiments for compound 6a

Information Classification: General

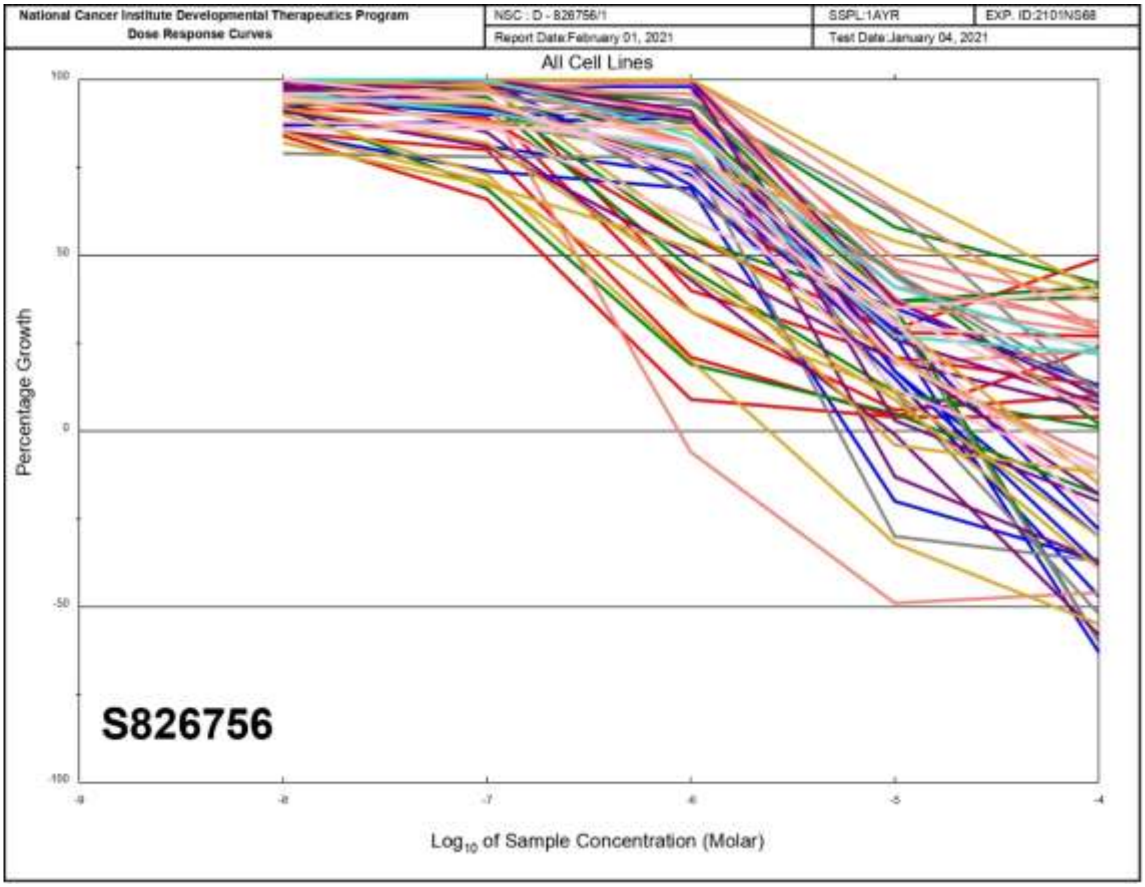

Figure S90. Dose-response curves for all cell lines in the NCI60 panel exposed compound 6a with tissue originated colors and shapes.

Information Classification: General

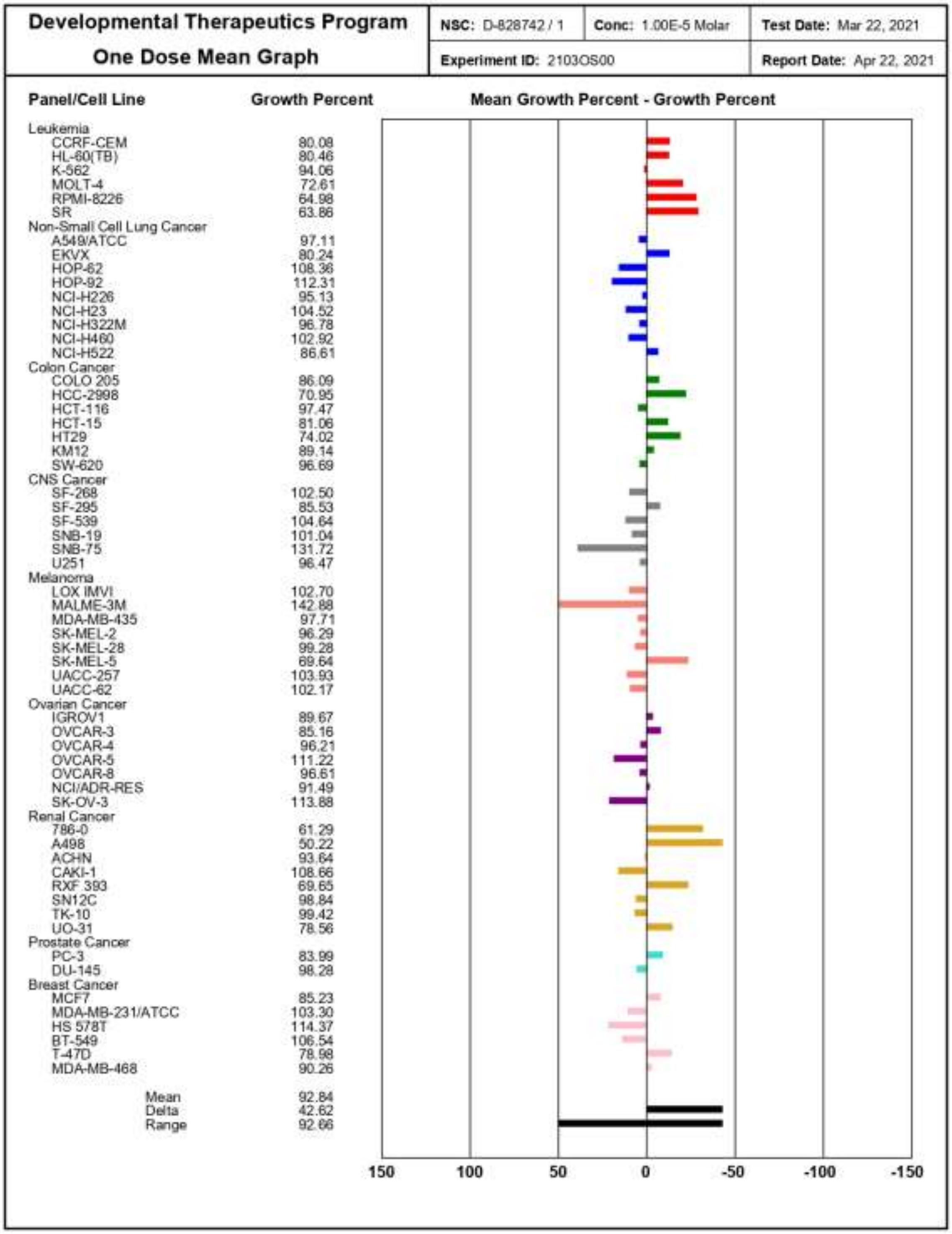

Figure S91. One dose mean graph for compound 6b (NSC 828742) at 10 µM

Information Classification: General

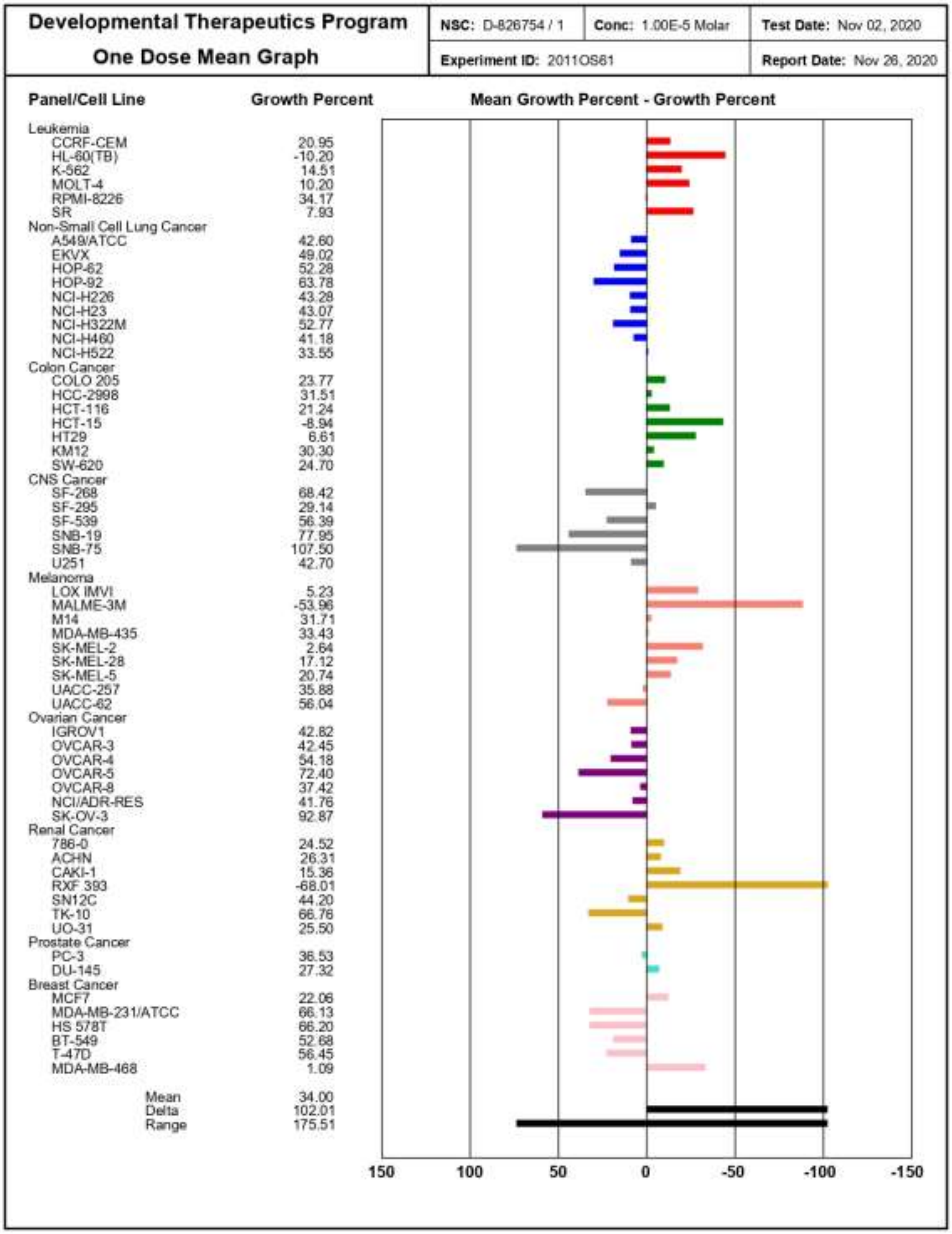

Figure S92. One dose mean graph for compound 6c (NSC 826754) at 10 μM

Information Classification: General

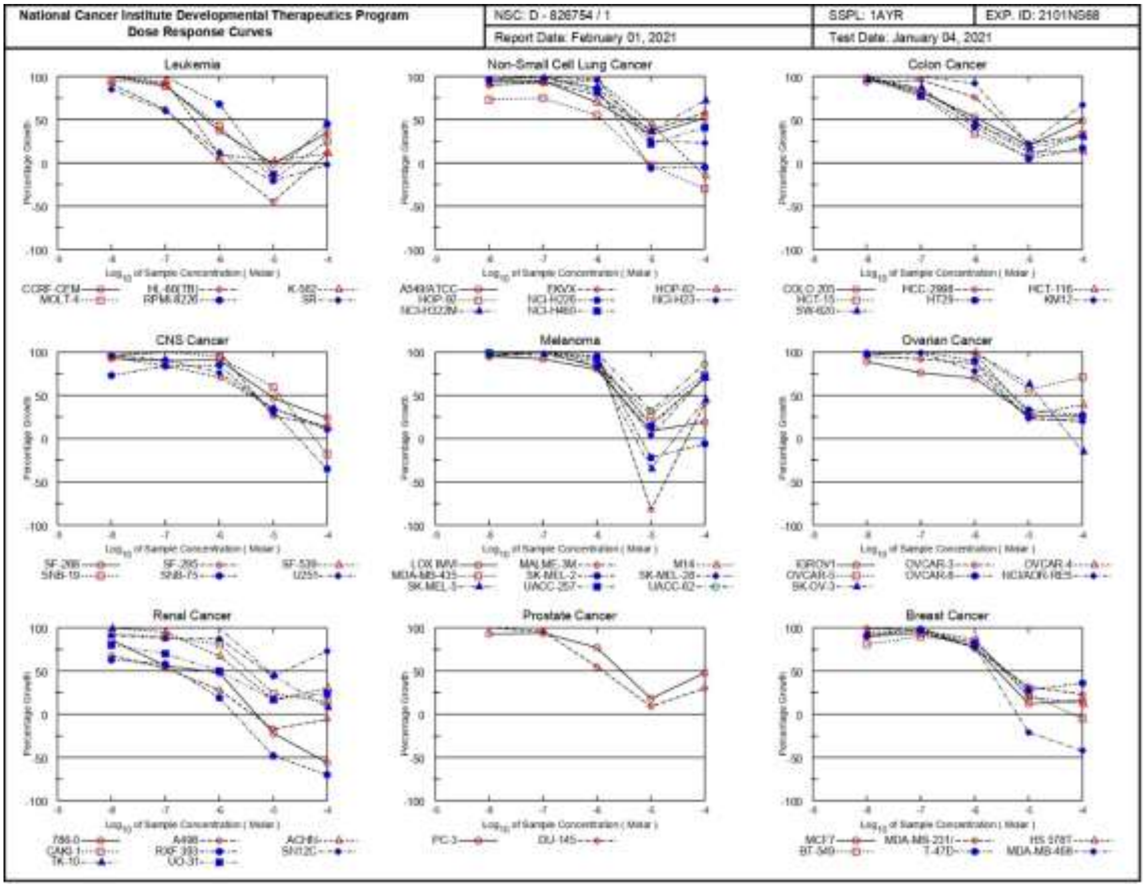

Figure S93. Dose-response curves (% growth versus sample concentration) for all cell lines with different subpanel obtained from the NCI's *in vitro* disease-oriented human cancer cells line for compound 6c on nine types of cancer

Information Classification: General

National Cancer Institute Developmental Therapeutics Program
In-Vitro Testing Results

NSC : D - 826754 / 1	Experiment ID : 2101NS68	Test Type : 08	Units : Molar
Report Date : February 01, 2021	Test Date : January 04, 2021	QNS :	MC :
COMI : AF_1	Stain Reagent : SRB Dual-Pass Related	SSPL : 1AYR	

	Log10 Concentration														
	Time		Mean Optical Densities					Percent Growth							
Panel/Cell Line	Zero	Ctrl	-8.0	-7.0	-6.0	-5.0	-4.0	-8.0	-7.0	-6.0	-5.0	-4.0	GI50	TGI	LC50
Leukemia															
CCRF-CEM	0.488	1.752	1.785	1.623	0.960	0.482	0.933	103	90	37	-1	35	5.74E-7		> 1.00E-4
HL-60(TB)	0.693	2.175	2.039	1.609	0.734	0.380	0.852	91	62	3	-45	11	1.58E-7		> 1.00E-4
K-562	0.282	2.096	2.140	1.995	0.464	0.327	0.486	102	94	10	2	11	3.36E-7	> 1.00E-4	> 1.00E-4
MOLT-4	0.721	2.424	2.355	2.230	1.446	0.584	1.140	96	89	43	-19	25	6.89E-7		> 1.00E-4
RPMI-8226	1.118	2.297	2.344	2.322	1.925	0.973	1.647	104	102	68	-13	45	1.68E-6		> 1.00E-4
SR	0.683	2.692	2.399	1.883	0.924	0.538	0.670	85	60	12	-21	-2	1.60E-7	2.29E-6	> 1.00E-4
Non-Small Cell Lung Cancer															
A549/ATCC	0.420	2.506	2.404	2.404	1.888	1.107	1.495	95	95	70	33	52		> 1.00E-4	> 1.00E-4
EKVX	0.568	1.508	1.415	1.456	1.314	0.916	1.112	90	94	79	37	58		> 1.00E-4	> 1.00E-4
HOP-62	0.895	2.637	2.484	2.495	2.568	1.684	0.757	91	92	96	45	-15	8.07E-6	5.56E-5	> 1.00E-4
HOP-92	1.491	2.142	1.968	1.982	1.852	1.448	1.047	73	75	55	-3	-30	1.24E-6	8.92E-6	> 1.00E-4
NCI-H226	1.047	2.175	2.129	2.262	2.021	0.979	0.995	96	108	86	-6	-5	2.46E-6	8.51E-6	> 1.00E-4
NCI-H23	0.661	1.817	1.769	1.811	1.766	0.960	0.923	96	100	96	26	23	4.50E-6	> 1.00E-4	> 1.00E-4
NCI-H322M	0.767	2.230	2.246	2.207	2.034	1.309	1.813	101	98	87	37	72		> 1.00E-4	> 1.00E-4
NCI-H460	0.296	2.503	2.566	2.619	2.103	0.790	1.200	103	105	82	22	41	3.43E-6	> 1.00E-4	> 1.00E-4
Colon Cancer															
COLO 205	0.637	2.668	2.626	2.313	1.715	1.040	1.635	98	82	53	20	49	1.24E-6	> 1.00E-4	> 1.00E-4
HCC-2998	0.633	1.946	1.853	1.898	1.637	0.921	1.051	93	96	76	22	32	3.06E-6	> 1.00E-4	> 1.00E-4
HCT-116	0.335	2.615	2.625	2.277	1.378	0.635	0.628	100	85	46	13	13	7.80E-7	> 1.00E-4	> 1.00E-4
HCT-15	0.296	1.805	1.756	1.452	0.812	0.400	0.788	97	77	34	7	33	4.23E-7	> 1.00E-4	> 1.00E-4
HT29	0.442	2.572	2.572	2.105	1.343	0.559	0.801	100	78	42	5	17	6.09E-7	> 1.00E-4	> 1.00E-4
KM12	0.854	3.255	3.253	3.256	3.063	1.392	2.466	100	100	92	22	67		> 1.00E-4	> 1.00E-4
SW-620	0.321	1.881	1.821	1.681	1.048	0.588	0.796	96	87	47	17	30	8.25E-7	> 1.00E-4	> 1.00E-4
CNS Cancer															
SF-268	0.964	2.607	2.499	2.437	2.466	1.730	1.357	93	90	91	47	24	8.40E-6	> 1.00E-4	> 1.00E-4
SF-295	0.980	3.116	3.032	3.213	3.187	1.548	1.270	96	105	103	26	14	4.94E-6	> 1.00E-4	> 1.00E-4
SF-539	1.014	2.864	2.757	2.576	2.332	1.658	1.252	94	84	71	35	13	3.83E-6	> 1.00E-4	> 1.00E-4
SNB-19	0.546	1.898	1.847	1.893	1.828	1.337	0.450	96	100	95	59	-18	1.29E-5	5.87E-5	> 1.00E-4
SNB-75	1.164	2.126	1.870	1.969	1.987	1.467	0.754	73	84	85	31	-35	4.54E-6	2.98E-5	> 1.00E-4
U251	0.429	2.078	2.009	1.922	1.678	1.000	0.592	96	91	76	35	10	4.22E-6	> 1.00E-4	> 1.00E-4
Melanoma															
LOX IMVI	0.305	1.748	1.675	1.630	1.464	0.434	0.572	95	92	80	9	19	2.66E-6	> 1.00E-4	> 1.00E-4
MALME-3M	0.832	2.285	2.206	2.610	2.279	0.150	1.435	95	122	100	-82	41	1.88E-6		
M14	0.545	1.971	1.991	2.184	1.817	0.783	1.538	101	115	89	17	70		> 1.00E-4	> 1.00E-4
MDA-MB-435	0.575	2.560	2.554	2.612	2.224	1.129	2.038	100	103	83	28	74		> 1.00E-4	> 1.00E-4
SK-MEL-2	1.298	2.516	2.507	2.665	2.459	1.010	1.221	99	112	95	-22	-6	2.43E-6	6.47E-6	> 1.00E-4
SK-MEL-28	0.553	1.716	1.704	1.790	1.516	0.594	1.380	99	106	83	4	71		> 1.00E-4	> 1.00E-4
SK-MEL-5	1.089	3.106	3.050	3.045	2.828	0.711	2.003	97	97	86	-35	45	1.99E-6		> 1.00E-4
UACC-257	1.013	2.520	2.503	2.692	2.414	1.217	2.077	99	111	93	14	71		> 1.00E-4	> 1.00E-4
UACC-62	0.918	2.664	2.617	2.829	2.870	1.478	2.422	97	109	112	32	86		> 1.00E-4	> 1.00E-4
Ovarian Cancer															
IGROV1	0.342	1.958	1.787	1.572	1.470	0.784	0.750	89	76	70	27	25	2.92E-6	> 1.00E-4	> 1.00E-4
OVCAR-3	0.626	1.917	1.849	1.814	1.753	0.925	0.891	95	92	87	23	20	3.81E-6	> 1.00E-4	> 1.00E-4
OVCAR-4	0.761	2.080	2.100	2.166	2.073	1.127	1.271	101	106	99	28	39	4.89E-6	> 1.00E-4	> 1.00E-4
OVCAR-5	0.491	1.389	1.387	1.521	1.402	1.002	1.131	100	115	102	57	71	> 1.00E-4	> 1.00E-4	> 1.00E-4
OVCAR-8	0.738	3.053	3.014	3.073	2.829	1.494	1.371	98	101	90	33	27	5.00E-6	> 1.00E-4	> 1.00E-4
NCI/ADR-RES	0.514	1.689	1.667	1.675	1.426	0.792	0.746	98	99	78	24	20	3.25E-6	> 1.00E-4	> 1.00E-4
SK-OV-3	1.344	2.535	2.500	2.675	2.655	2.096	1.141	97	112	110	63	-15	1.47E-5	6.40E-5	> 1.00E-4
Renal Cancer															
786-0	0.678	2.642	2.371	1.782	1.614	0.526	0.297	86	56	48	-22	-56	5.29E-7	4.78E-6	6.52E-5
A498	1.770	2.444	2.231	2.130	1.961	1.475	1.666	68	53	28	-17	-6	1.37E-7	4.26E-6	> 1.00E-4
ACHN	0.474	1.989	1.990	1.913	1.495	0.728	0.928	100	95	67	17	30	2.21E-6	> 1.00E-4	> 1.00E-4
CAKI-1	0.918	3.029	2.887	2.790	2.625	1.434	1.211	93	89	81	24	14	3.52E-6	> 1.00E-4	> 1.00E-4
RXF 393	1.070	1.457	1.316	1.294	1.145	0.562	0.317	63	58	19	-48	-70	1.58E-7	1.95E-6	1.28E-5
SN12C	1.323	3.335	3.147	3.098	3.068	2.208	2.798	91	88	87	44	73		> 1.00E-4	> 1.00E-4
TK-10	1.107	1.938	1.929	2.156	2.207	1.481	1.176	99	126	132	45	8	8.75E-6	> 1.00E-4	> 1.00E-4
UO-31	0.716	2.388	2.059	1.883	1.554	0.994	1.116	80	70	50	17	24	1.01E-6	> 1.00E-4	> 1.00E-4
Prostate Cancer															
PC-3	0.835	2.423	2.310	2.325	2.065	1.124	1.603	93	94	77	18	48	2.90E-6	> 1.00E-4	> 1.00E-4
DU-145	0.453	1.741	1.773	1.696	1.167	0.571	0.836	102	96	55	9	30	1.31E-6	> 1.00E-4	> 1.00E-4
Breast Cancer															
MCF7	0.480	2.198	2.025	2.095	1.814	0.689	0.754	90	94	78	12	16	2.64E-6	> 1.00E-4	> 1.00E-4
MDA-MB-231/ATCC	0.885	1.422	1.417	1.425	1.247	0.924	0.856	99	100	76	32	23	3.97E-6	> 1.00E-4	> 1.00E-4
HS 578T	0.817	1.738	1.695	1.703	1.618	1.005	0.924	95	96	87	20	12	3.60E-6	> 1.00E-4	> 1.00E-4
BT-549	1.257	2.262	2.072	2.161	2.090	1.488	1.196	81	90	83	23	-5	3.54E-6	6.69E-5	> 1.00E-4
T-47D	1.008	2.545	2.403	2.517	2.275	1.438	1.560	91	98	82	28	36	3.94E-6	> 1.00E-4	> 1.00E-4
MDA-MB-468	0.703	1.716	1.727	1.675	1.494	0.553	0.405	101	96	78	-21	-42	1.91E-6	6.09E-6	> 1.00E-4

Figure S94. Values of log molar concentration of response parameters (log_{10} GI_{50}, log_{10} TGI & log_{10} LC_{50}) for compound 6c

Information Classification: General

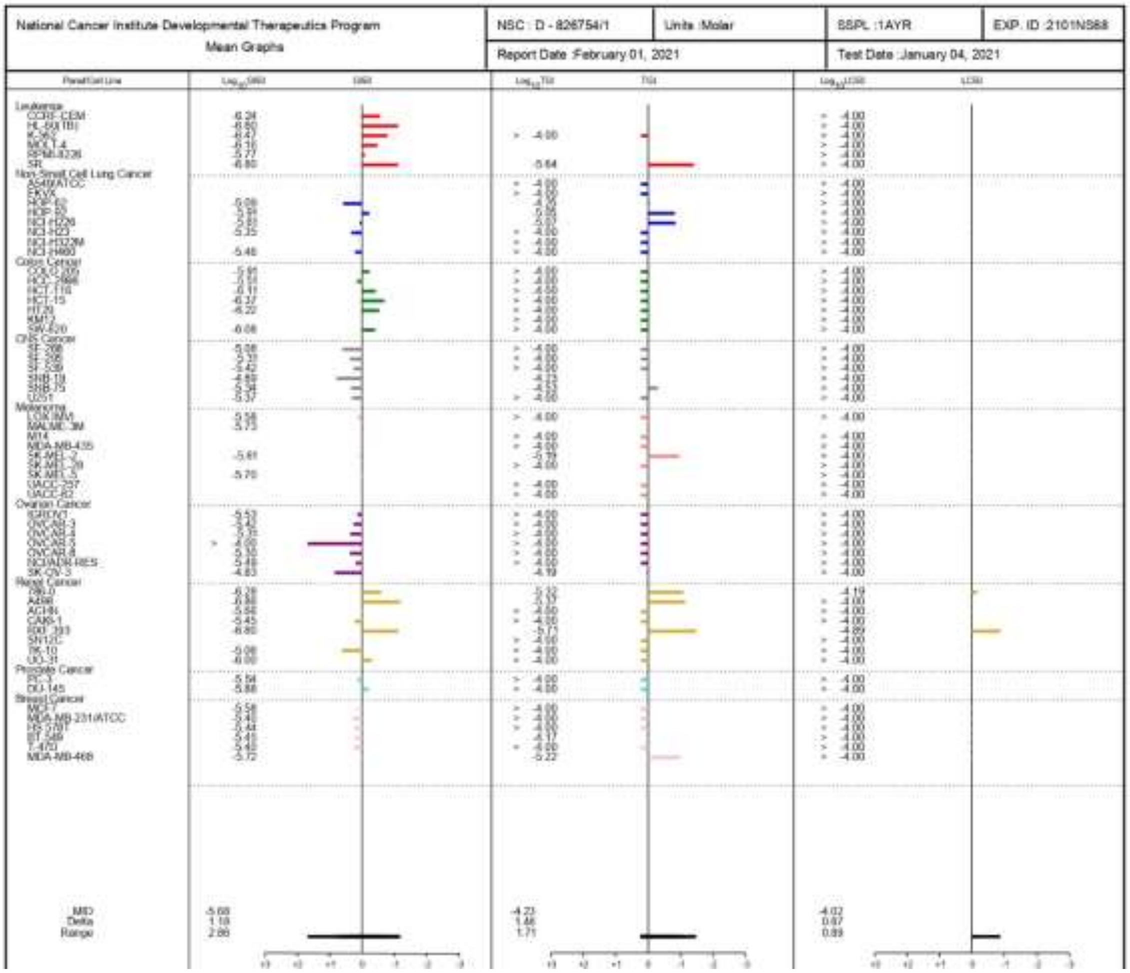

Figure S95. Mean Graphs of the log_{10} values (Molar) of GI_{50}, TGI, and LC_{50} obtained from the NCI 60 cell line experiments for compound 6c

Information Classification: General

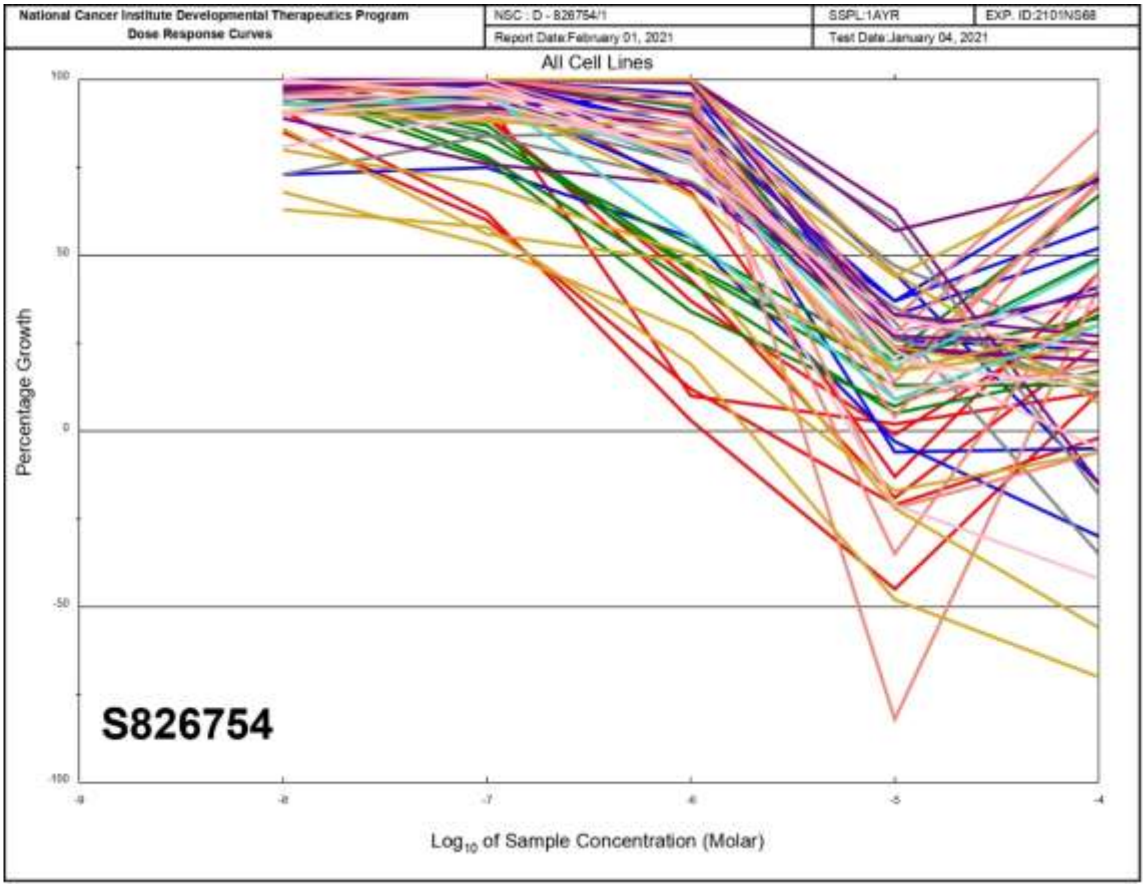

Figure S96. Dose-response curves for all cell lines in the NCI60 panel exposed compound 6c with tissue originated colors and shapes.

Information Classification: General

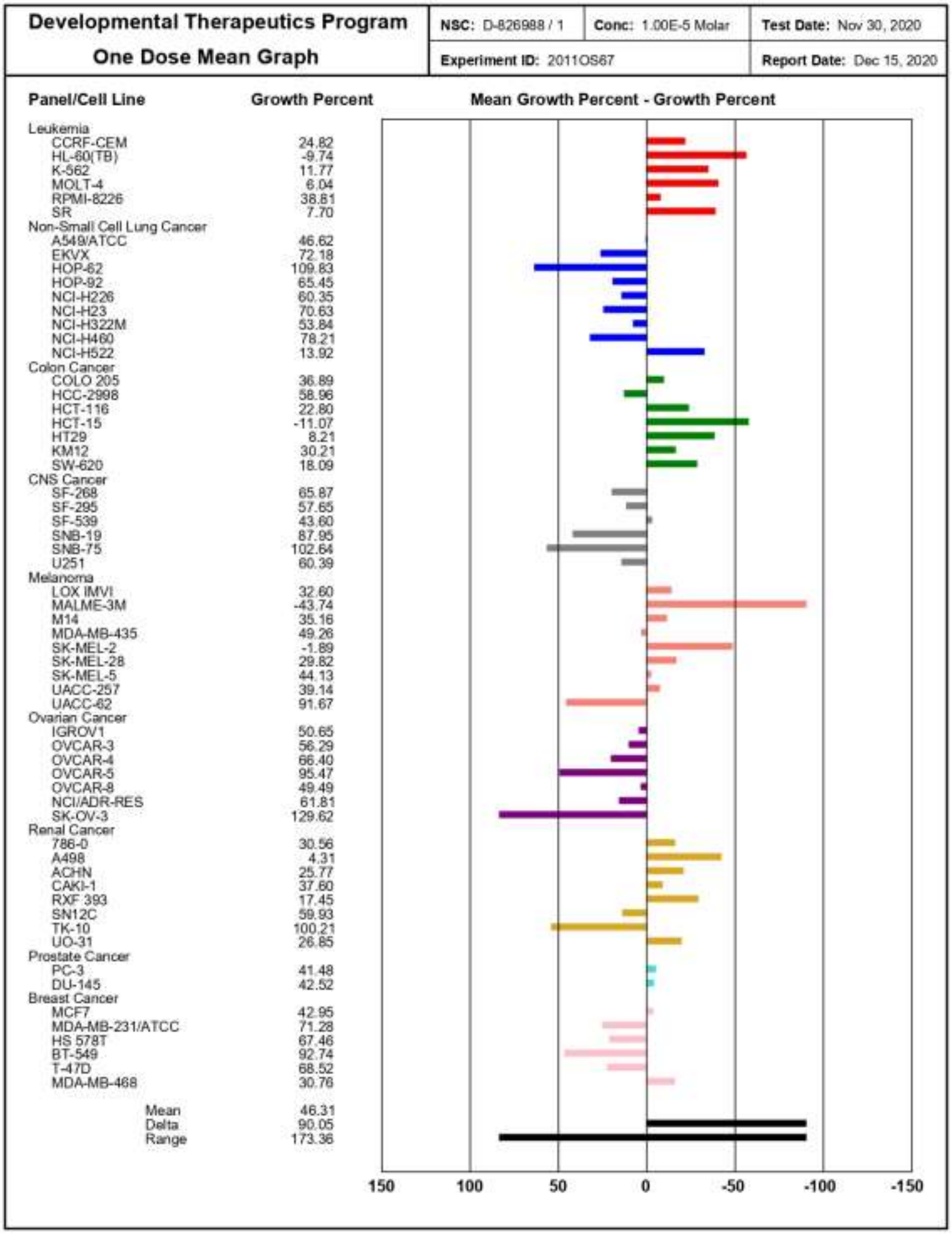

Figure S97. One dose mean graph for compound 6d (NSC 826988) at 10 µM

Information Classification: General

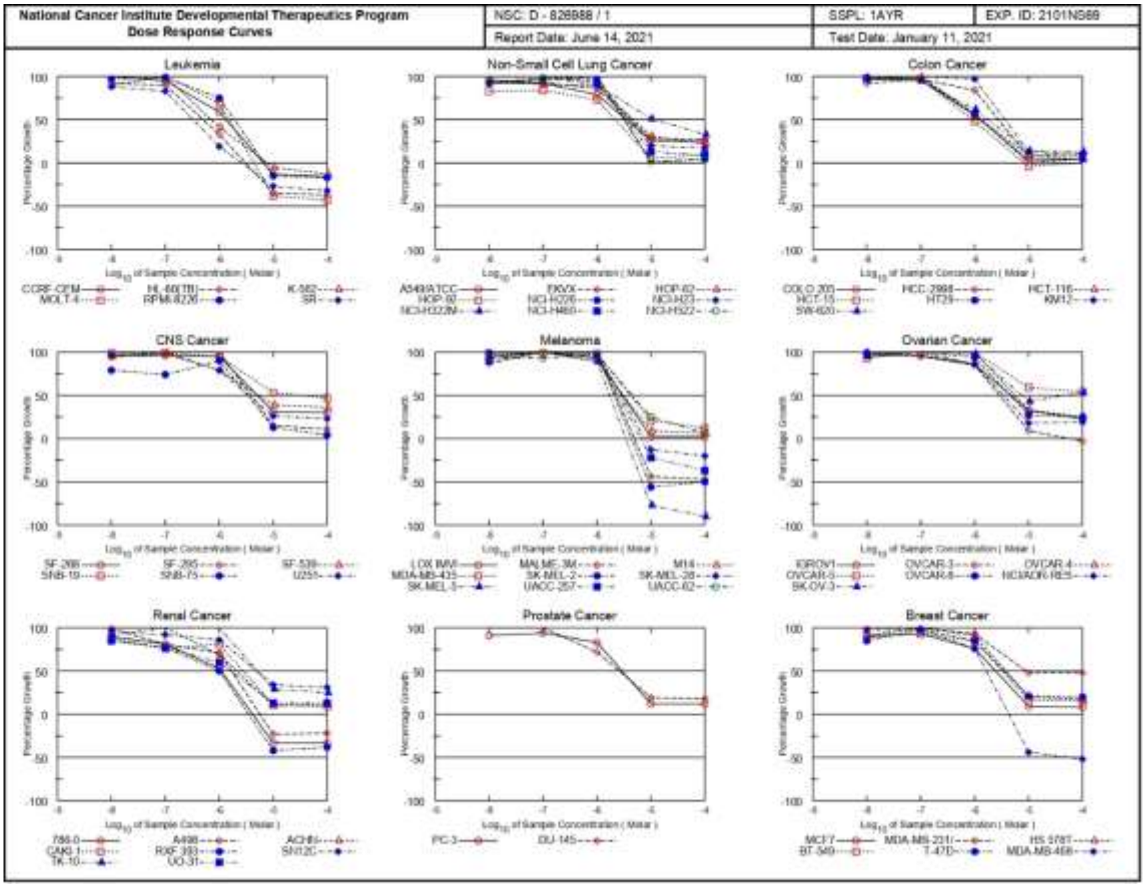

Figure S98. Dose-response curves (% growth versus sample concentration) for all cell lines with different subpanel obtained from the NCI's *in vitro* disease-oriented human cancer cells line for compound 6d on nine types of cancer

Information Classification: General

National Cancer Institute Developmental Therapeutics Program
In-Vitro Testing Results

NSC : D - 826988 / 1	Experiment ID : 2101NS69	Test Type : 08	Units : Molar
Report Date : June 14, 2021	Test Date : January 11, 2021	QNS :	MC :
COMI : AF_7	Stain Reagent : SRB Dual-Pass Related	SSPL : 1AYR	

			Log10 Concentration												
	Time		Mean Optical Densities					Percent Growth							
Panel/Cell Line	Zero	Ctrl	-8.0	-7.0	-6.0	-5.0	-4.0	-8.0	-7.0	-6.0	-5.0	-4.0	GI50	TGI	LC50
Leukemia															
CCRF-CEM	0.454	1.618	1.660	1.565	1.143	0.395	0.380	104	95	59	-13	-16	1.34E-6	6.60E-6	> 1.00E-4
HL-60(TB)	0.880	2.810	2.653	2.613	1.521	0.575	0.553	92	90	33	-35	-37	5.05E-7	3.08E-6	> 1.00E-4
K-562	0.252	1.469	1.342	1.469	0.748	0.230	0.220	90	100	41	-5	-13	6.99E-7	7.65E-6	> 1.00E-4
MOLT-4	0.517	1.732	1.779	1.873	1.350	0.321	0.293	104	112	69	-38	-43	1.49E-6	4.40E-6	> 1.00E-4
RPMI-8226	0.762	1.914	1.884	1.898	1.629	0.646	0.635	97	99	75	-15	-17	1.90E-6	6.79E-6	> 1.00E-4
SR	0.511	2.100	1.912	1.834	0.811	0.374	0.347	88	83	19	-27	-32	3.29E-7	2.58E-6	> 1.00E-4
Non-Small Cell Lung Cancer															
A549/ATCC	0.410	2.336	2.265	2.188	1.933	0.919	0.879	96	92	79	26	24	3.57E-6	> 1.00E-4	> 1.00E-4
EKVX	0.703	1.946	1.844	1.861	1.775	1.056	1.045	92	93	86	28	27	4.23E-6	> 1.00E-4	> 1.00E-4
HOP-62	0.753	2.495	2.376	2.333	2.300	1.291	1.132	93	91	89	31	22	4.67E-6	> 1.00E-4	> 1.00E-4
HOP-92	1.012	1.826	1.687	1.698	1.606	1.051	1.092	83	84	73	5	10	2.17E-6	> 1.00E-4	> 1.00E-4
NCI-H226	1.214	2.478	2.373	2.458	2.474	1.211	1.273	92	98	100	.	5	3.14E-6	.	> 1.00E-4
NCI-H23	0.779	2.128	2.021	2.073	2.090	1.043	1.008	92	96	97	20	17	4.05E-6	> 1.00E-4	> 1.00E-4
NCI-H322M	1.028	2.622	2.658	2.680	2.460	1.841	1.554	102	104	90	51	33	1.13E-5	> 1.00E-4	> 1.00E-4
NCI-H460	0.309	2.788	2.803	2.880	2.617	0.642	0.542	101	104	93	13	9	3.47E-6	> 1.00E-4	> 1.00E-4
NCI-H522	1.588	3.113	3.020	3.074	3.109	1.613	1.651	94	97	100	2	4	3.21E-6	> 1.00E-4	> 1.00E-4
Colon Cancer															
COLO 205	0.465	1.871	1.830	1.820	1.234	0.513	0.534	97	96	55	3	5	1.23E-6	> 1.00E-4	> 1.00E-4
HCC-2998	0.655	2.140	2.026	2.102	1.896	0.752	0.812	92	97	84	7	11	2.73E-6	> 1.00E-4	> 1.00E-4
HCT-116	0.127	1.194	1.262	1.186	0.711	0.222	0.168	106	99	55	9	4	1.27E-6	> 1.00E-4	> 1.00E-4
HCT-15	0.271	1.637	1.605	1.600	0.926	0.260	0.273	98	97	48	-4	.	9.08E-7	.	> 1.00E-4
HT29	0.431	2.526	2.455	2.550	1.629	0.438	0.538	97	101	57	.	5	1.34E-6	> 1.00E-4	> 1.00E-4
KM12	0.628	2.770	2.746	2.847	2.700	0.905	0.827	99	104	97	13	9	3.61E-6	> 1.00E-4	> 1.00E-4
SW-620	0.263	1.816	1.791	1.740	1.228	0.476	0.461	98	95	62	14	13	1.78E-6	> 1.00E-4	> 1.00E-4
CNS Cancer															
SF-268	0.721	2.275	2.210	2.214	2.198	1.209	1.181	96	96	95	31	30	5.10E-6	> 1.00E-4	> 1.00E-4
SF-295	0.731	2.745	2.626	2.907	2.852	1.042	0.950	94	108	105	15	11	4.13E-6	> 1.00E-4	> 1.00E-4
SF-539	1.011	2.916	2.824	2.885	2.523	1.758	1.700	95	98	79	39	36	5.39E-6	> 1.00E-4	> 1.00E-4
SNB-19	0.404	1.625	1.618	1.608	1.566	1.055	0.984	99	99	95	53	47	3.70E-5	> 1.00E-4	> 1.00E-4
SNB-75	1.522	2.496	2.296	2.245	2.400	1.652	1.564	79	74	90	13	4	3.33E-6	> 1.00E-4	> 1.00E-4
U251	0.374	1.874	1.832	1.914	1.558	0.759	0.714	97	103	79	26	23	3.49E-6	> 1.00E-4	> 1.00E-4
Melanoma															
LOX IMVI	0.209	1.370	1.306	1.365	1.292	0.238	0.230	94	100	93	2	2	2.99E-6	> 1.00E-4	> 1.00E-4
MALME-3M	0.774	1.361	1.287	1.423	1.530	0.434	0.410	87	111	129	-44	-47	2.86E-6	5.57E-6	> 1.00E-4
M14	0.439	1.882	1.808	1.885	1.723	0.573	0.530	95	100	89	9	6	3.08E-6	> 1.00E-4	> 1.00E-4
MDA-MB-435	0.723	3.108	3.051	3.083	3.064	1.235	1.015	98	99	98	21	12	4.24E-6	> 1.00E-4	> 1.00E-4
SK-MEL-2	1.374	2.581	2.573	2.670	2.627	0.611	0.689	99	107	104	-56	-50	2.18E-6	4.48E-6	.
SK-MEL-28	0.926	2.536	2.560	2.636	2.581	0.807	0.737	101	106	103	-13	-20	2.86E-6	7.74E-6	> 1.00E-4
SK-MEL-5	0.999	3.003	3.005	3.016	2.787	0.230	0.098	100	101	89	-77	-90	1.72E-6	3.44E-6	6.88E-6
UACC-257	1.046	2.584	2.438	2.609	2.529	0.816	0.658	90	102	96	-22	-37	2.47E-6	6.52E-6	> 1.00E-4
UACC-62	0.770	2.687	2.550	2.526	2.610	1.253	0.878	94	93	97	25	6	4.54E-6	> 1.00E-4	> 1.00E-4
Ovarian Cancer															
IGROV1	0.539	2.303	2.258	2.239	2.050	1.102	0.946	97	96	86	32	23	4.61E-6	> 1.00E-4	> 1.00E-4
OVCAR-3	0.769	2.253	2.251	2.185	2.026	0.901	0.750	100	95	85	9	-3	2.87E-6	5.99E-5	> 1.00E-4
OVCAR-4	1.016	2.402	2.382	2.486	2.482	1.472	1.363	99	106	106	33	25	5.82E-6	> 1.00E-4	> 1.00E-4
OVCAR-5	0.681	1.742	1.666	1.738	1.896	1.303	1.259	93	100	114	59	54	> 1.00E-4	> 1.00E-4	> 1.00E-4
OVCAR-8	0.638	2.903	2.879	2.968	2.774	1.256	1.208	99	103	94	27	25	4.58E-6	> 1.00E-4	> 1.00E-4
NCI/ADR-RES	0.524	1.766	1.742	1.776	1.596	0.746	0.756	98	101	86	18	19	3.39E-6	> 1.00E-4	> 1.00E-4
SK-OV-3	0.865	2.009	1.947	2.074	1.987	1.356	1.473	95	106	98	43	53		> 1.00E-4	> 1.00E-4
Renal Cancer															
786-0	0.480	2.365	2.175	2.009	1.472	0.322	0.322	90	81	53	-33	-33	1.07E-6	4.12E-6	> 1.00E-4
A498	1.315	2.035	2.030	1.895	1.826	1.007	1.024	99	81	71	-23	-22	1.67E-6	5.65E-6	> 1.00E-4
ACHN	0.560	2.231	2.266	2.303	1.719	0.721	0.741	102	104	69	10	11	2.11E-6	> 1.00E-4	> 1.00E-4
CAKI-1	0.536	2.105	1.913	1.727	1.804	0.713	0.674	88	76	81	11	9	2.77E-6	> 1.00E-4	> 1.00E-4
RXF 393	1.001	1.578	1.493	1.445	1.291	0.583	0.617	85	77	50	-42	-38	1.00E-6	3.51E-6	> 1.00E-4
SN12C	0.602	2.386	2.314	2.241	2.144	1.213	1.151	96	92	86	34	31	4.99E-6	> 1.00E-4	> 1.00E-4
TK-10	0.970	1.953	1.874	2.108	2.167	1.251	1.216	92	116	122	29	25	5.89E-6	> 1.00E-4	> 1.00E-4
UO-31	0.918	2.343	2.131	2.009	1.767	1.110	1.108	85	77	60	13	13	1.61E-6	> 1.00E-4	> 1.00E-4
Prostate Cancer															
PC-3	0.649	2.457	2.287	2.343	2.151	0.864	0.860	91	94	83	12	12	2.91E-6	> 1.00E-4	> 1.00E-4
DU-145	0.296	1.331	1.363	1.331	1.044	0.495	0.478	103	100	72	19	18	2.62E-6	> 1.00E-4	> 1.00E-4
Breast Cancer															
MCF7	0.406	2.011	1.859	1.904	1.633	0.554	0.527	90	93	76	9	8	2.47E-6	> 1.00E-4	> 1.00E-4
MDA-MB-231/ATCC	0.541	1.477	1.482	1.487	1.414	0.993	0.986	101	101	93	48	48	9.16E-6	> 1.00E-4	> 1.00E-4
HS 578T	1.250	2.426	2.325	2.429	2.320	1.482	1.447	91	100	91	20	17	3.76E-6	> 1.00E-4	> 1.00E-4
BT-549	0.876	1.753	1.674	1.693	1.623	1.016	1.017	91	93	85	16	16	3.22E-6	> 1.00E-4	> 1.00E-4
T-47D	1.069	2.652	2.420	2.612	2.415	1.406	1.381	85	98	85	21	20	3.54E-6	> 1.00E-4	> 1.00E-4
MDA-MB-468	0.666	1.701	1.676	1.676	1.454	0.371	0.321	98	98	76	-44	-52	1.65E-6	4.28E-6	5.62E-5

Figure S99. Values of log molar concentration of response parameters (log_{10} GI_{50}, log_{10} TGI & log_{10} LC_{50}) for compound 6d

Information Classification: General

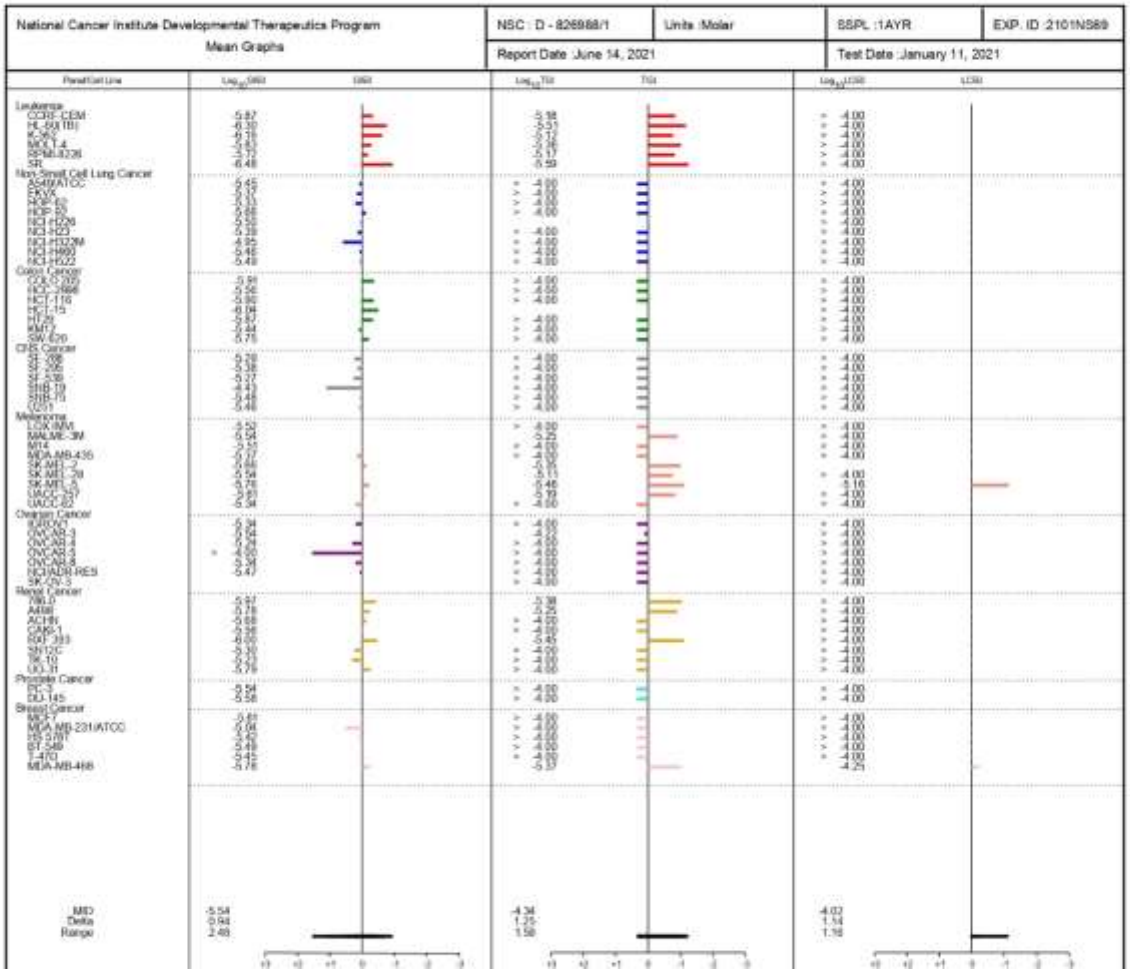

Figure S100. Mean Graphs of the $\log_{10}$ values (Molar) of GI_{50}, TGI, and LC_{50} obtained from the NCI 60 cell line experiments for compound 6d

Information Classification: General

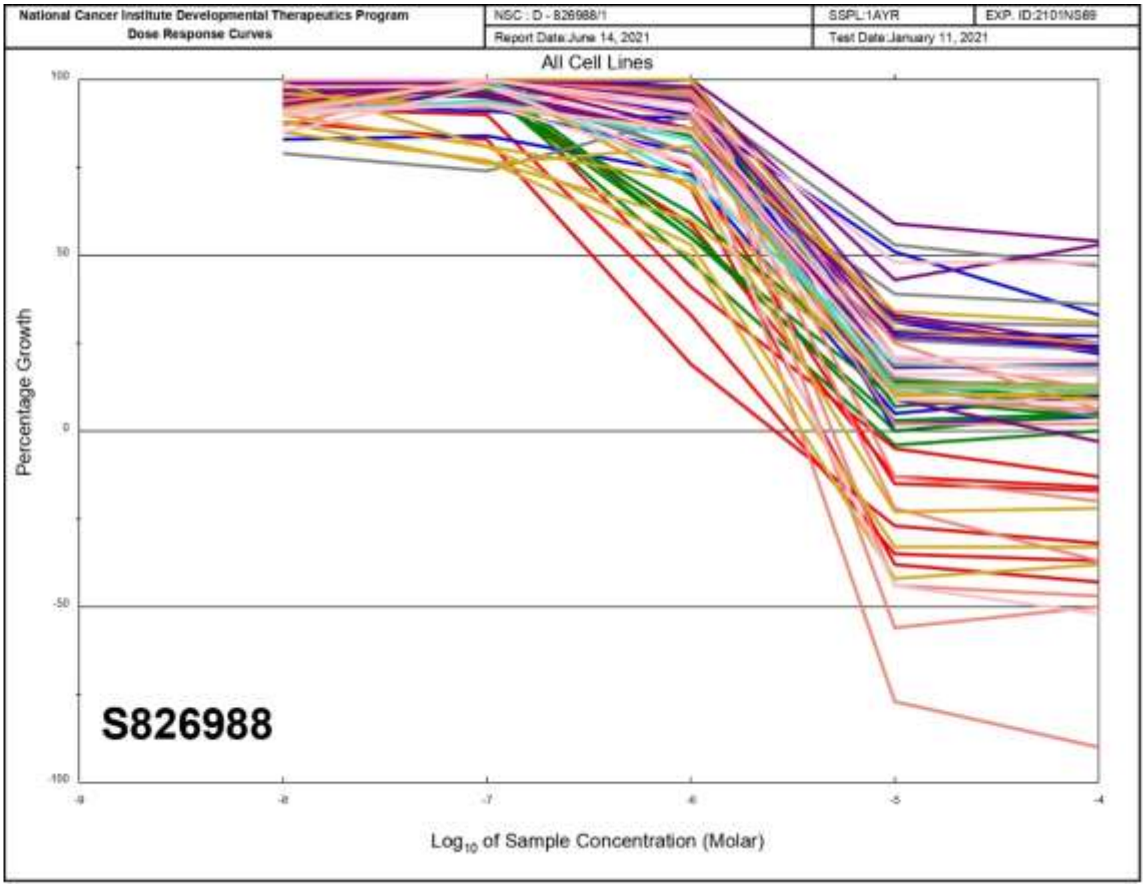

Figure S101. Dose-response curves for all cell lines in the NCI60 panel exposed compound 6d with tissue originated colors and shapes.

Information Classification: General

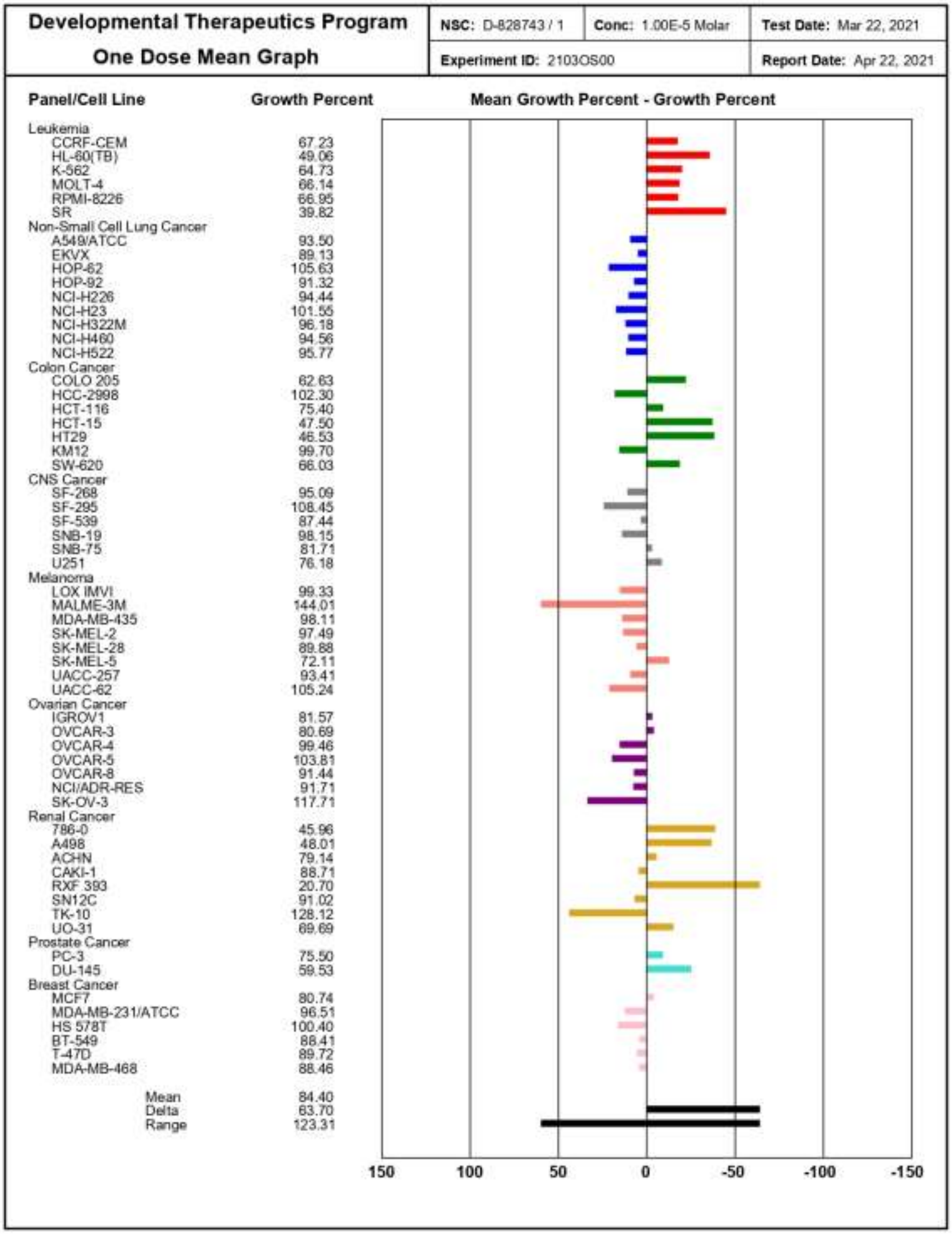

Figure S102. One dose mean graph for compound 6e (NSC 828743) at 10 μM

Information Classification: General

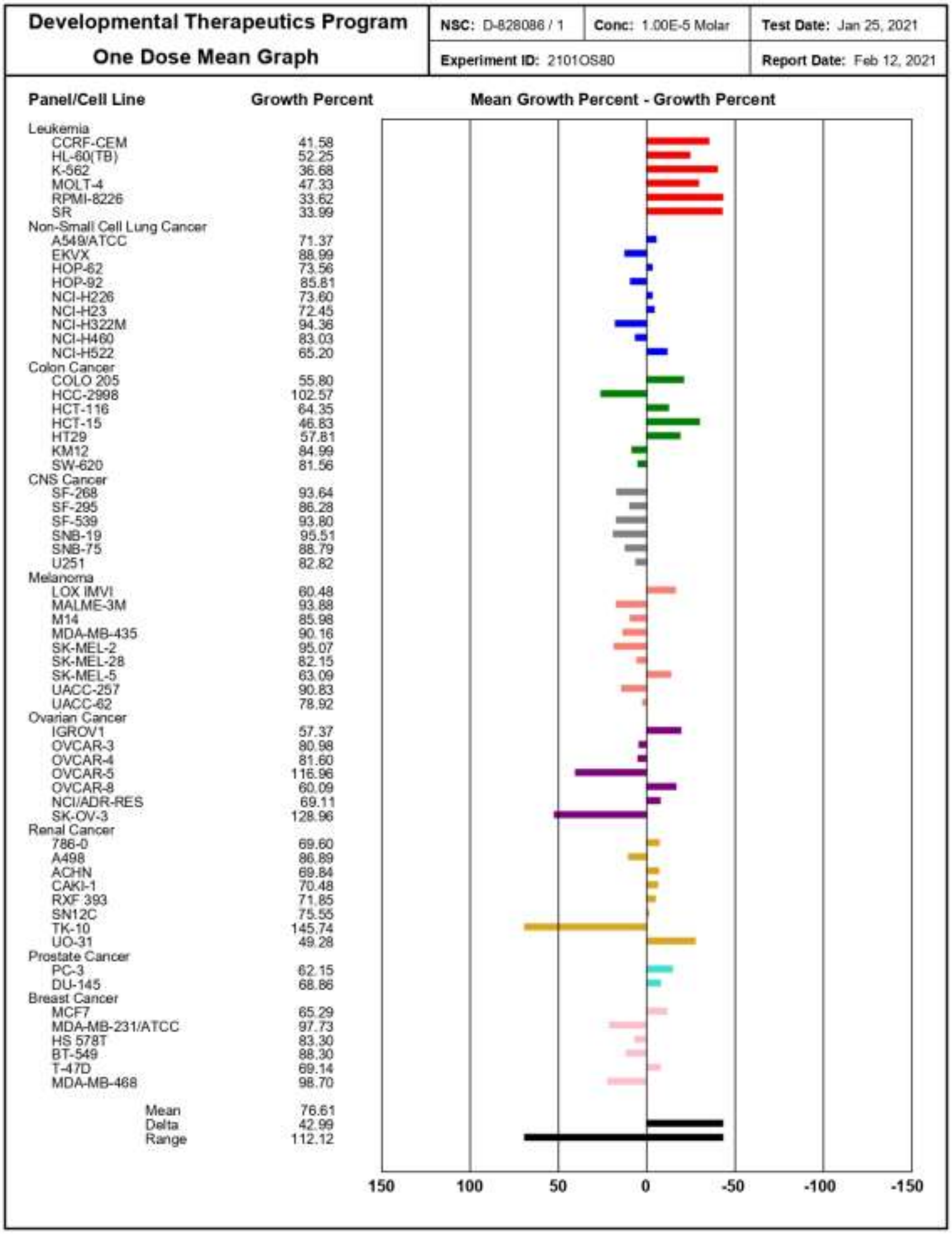

Figure S103. One dose mean graph for compound 6f (NSC 828086) at 10 µM

Information Classification: General

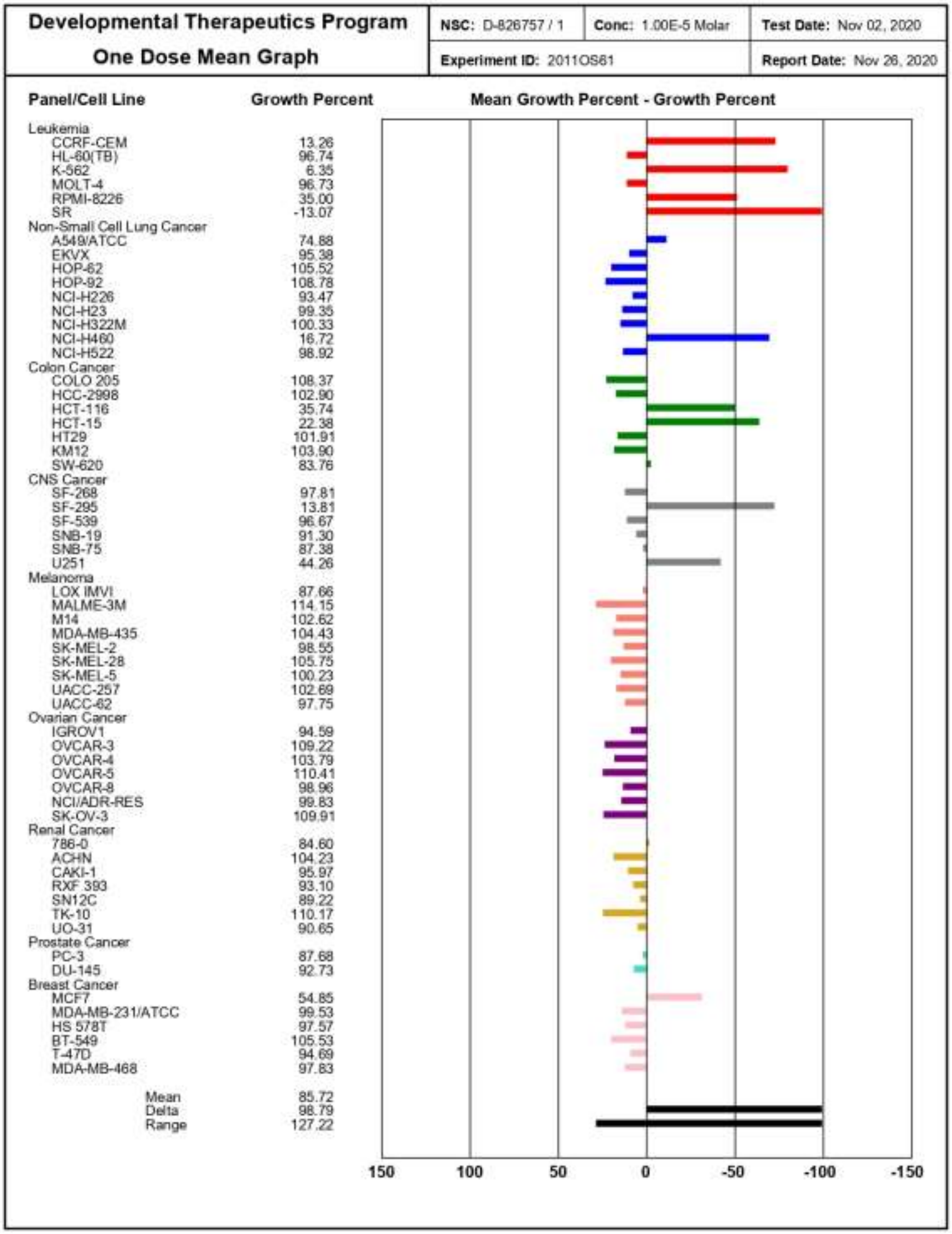

Figure S104. One dose mean graph for compound 6g (NSC 826757) at 10 μM

Information Classification: General

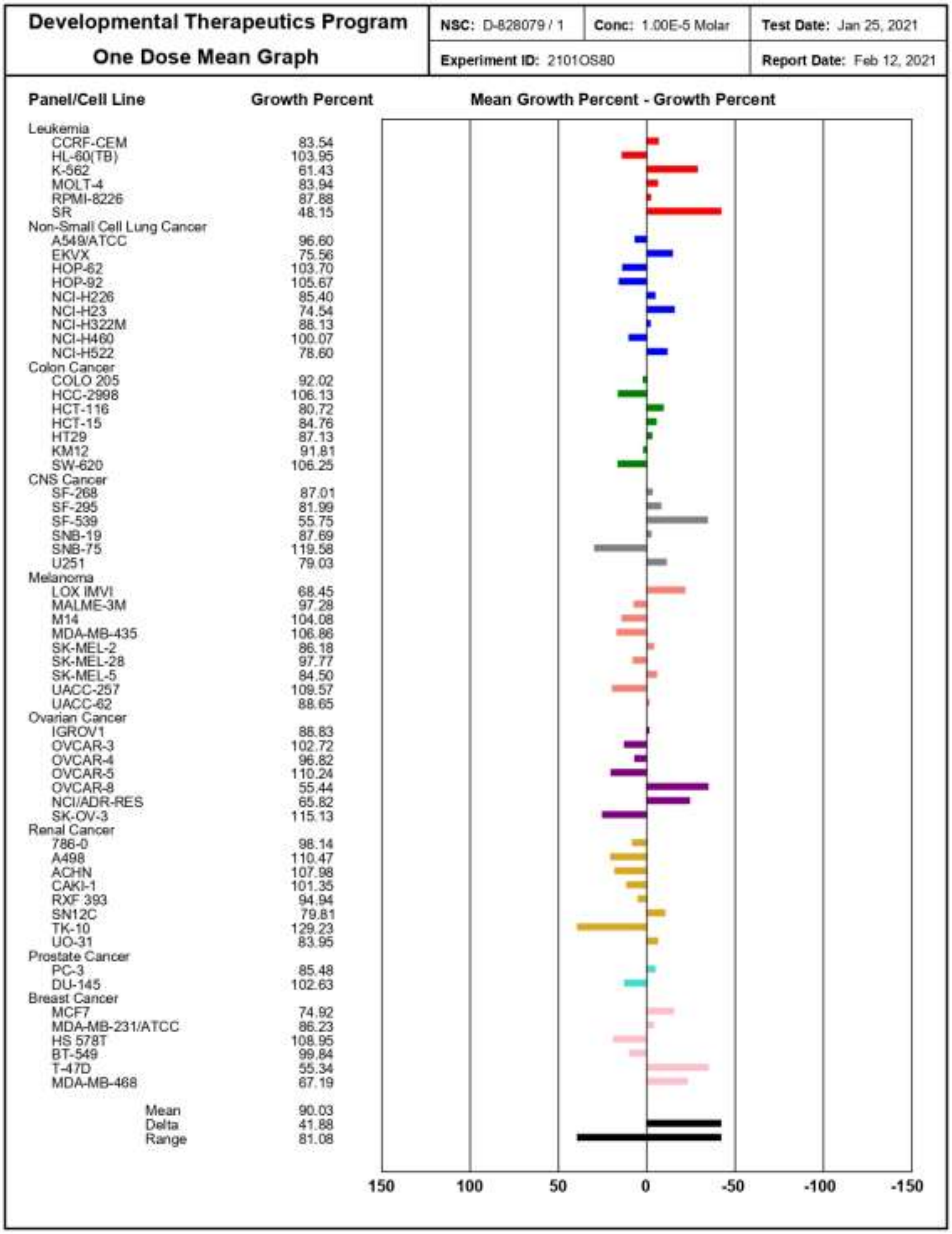

Figure S105. One dose mean graph for compound 6h (NSC 828079) at 10 µM

Information Classification: General

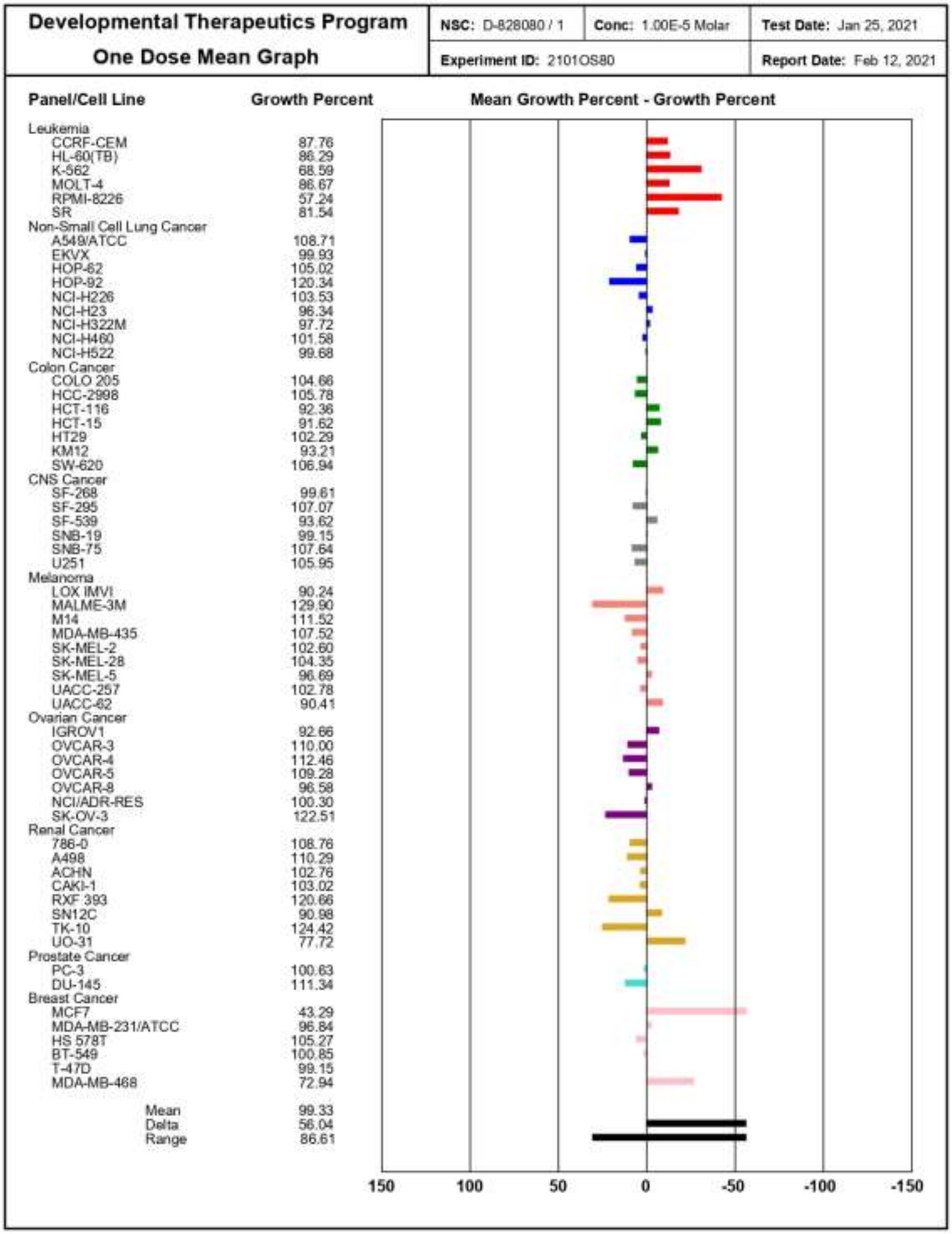

Figure S106. One dose mean graph for compound 6i (NSC 828080) at 10 μM

Information Classification: General

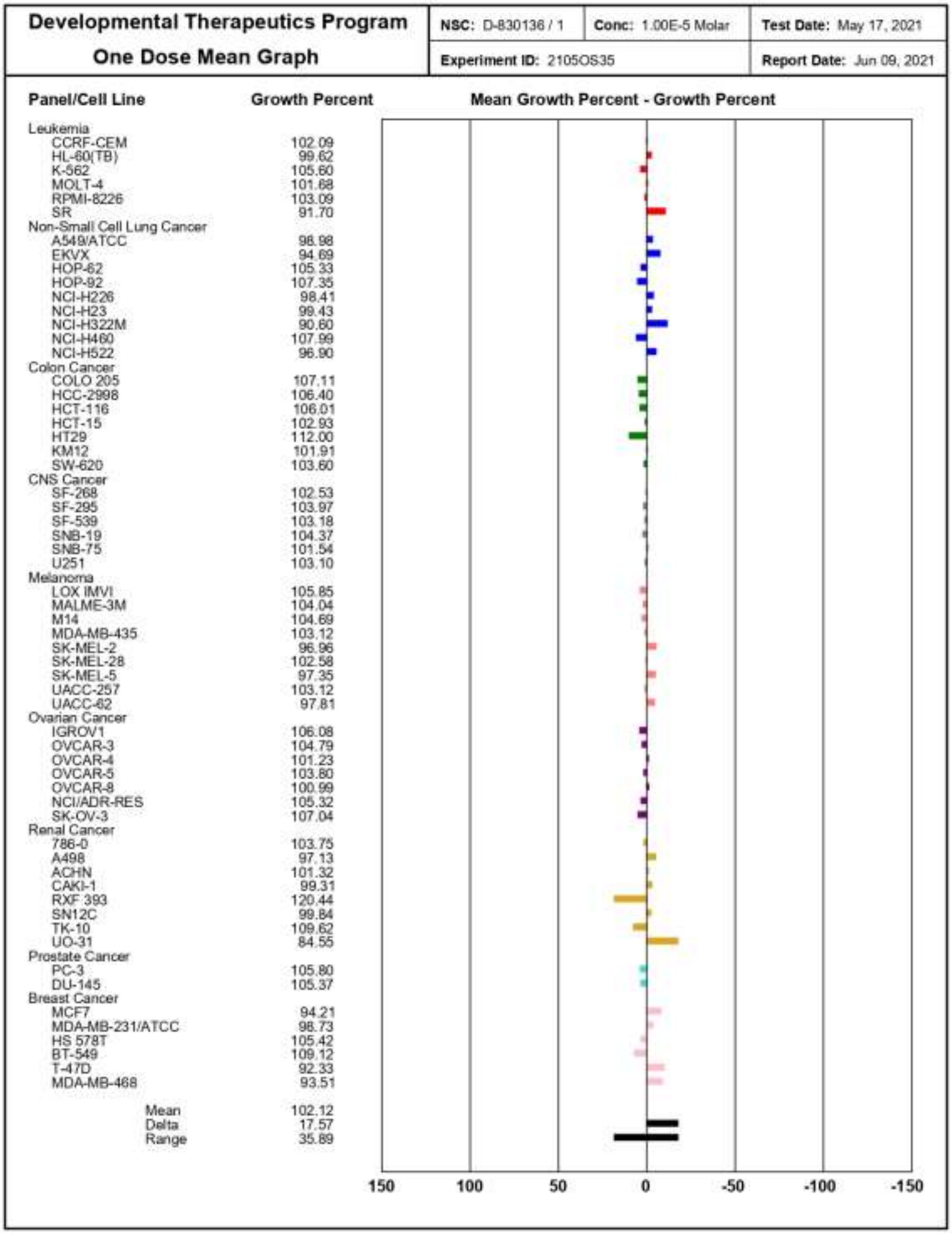

Figure S107. One dose mean graph for compound 6j (NSC 830136) at 10 µM

Information Classification: General

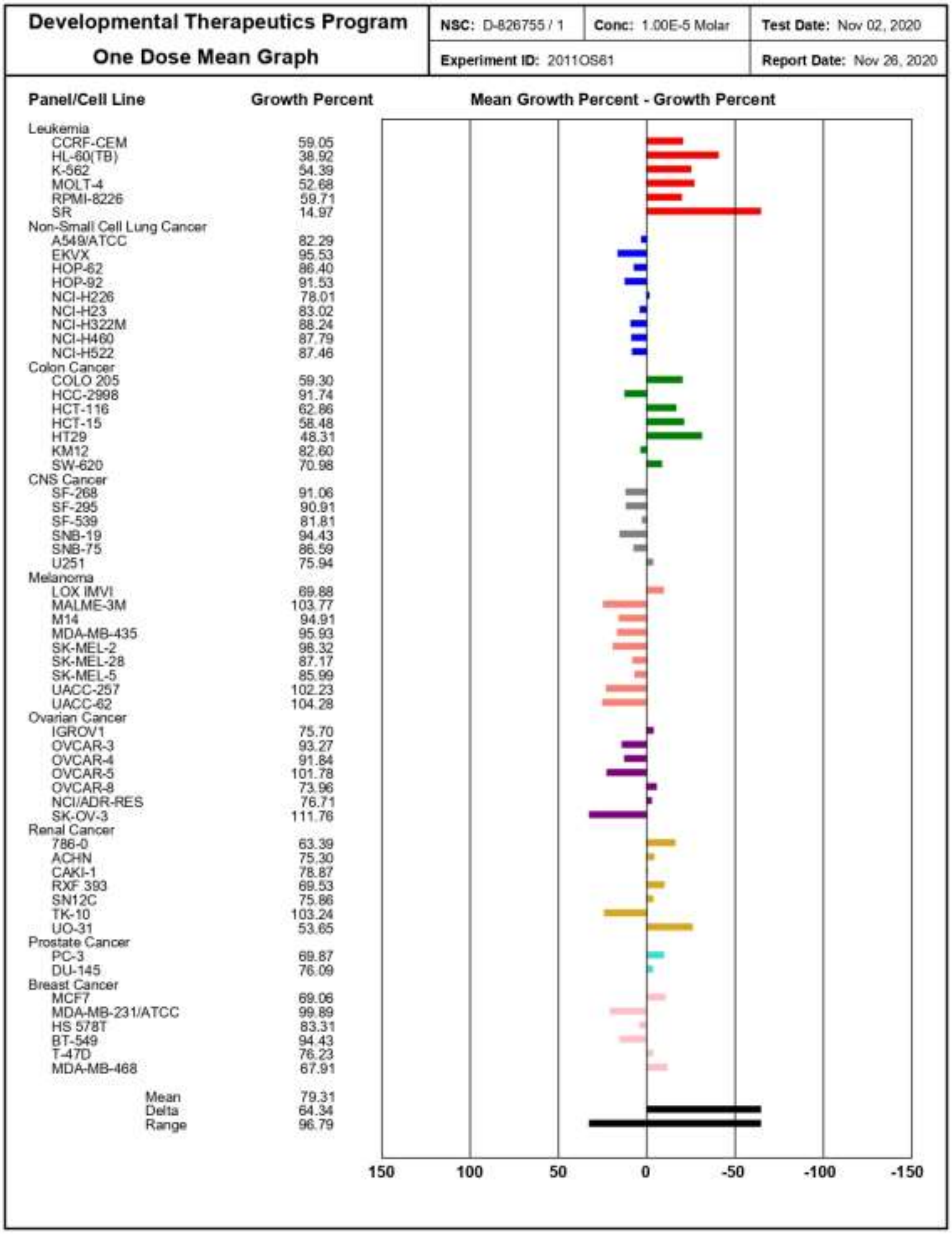

Figure S108. One dose mean graph for compound 6k (NSC 826755) at 10 μM

Information Classification: General

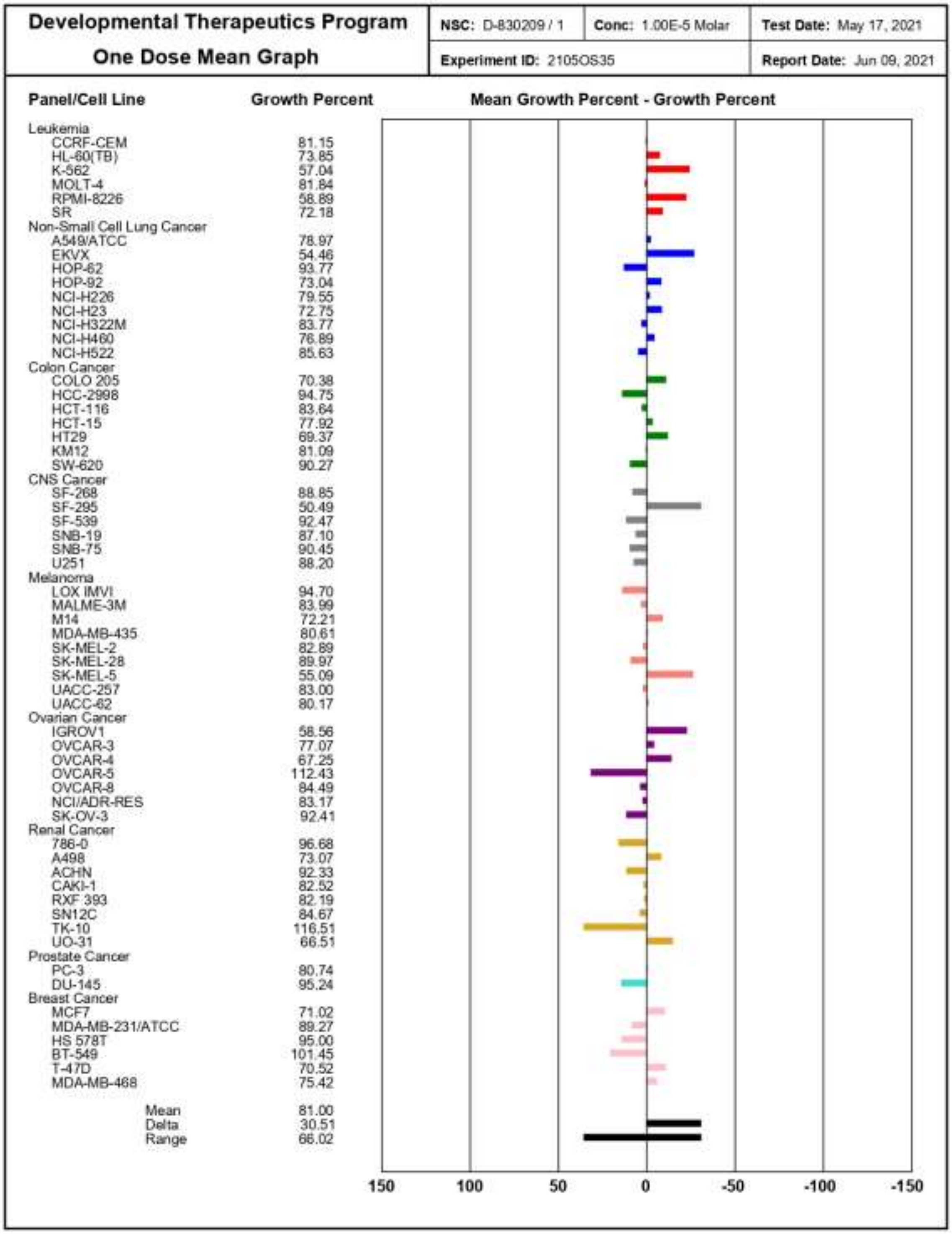

Figure S109. One dose mean graph for compound 6l (NSC 830209) at 10 µM

Information Classification: General

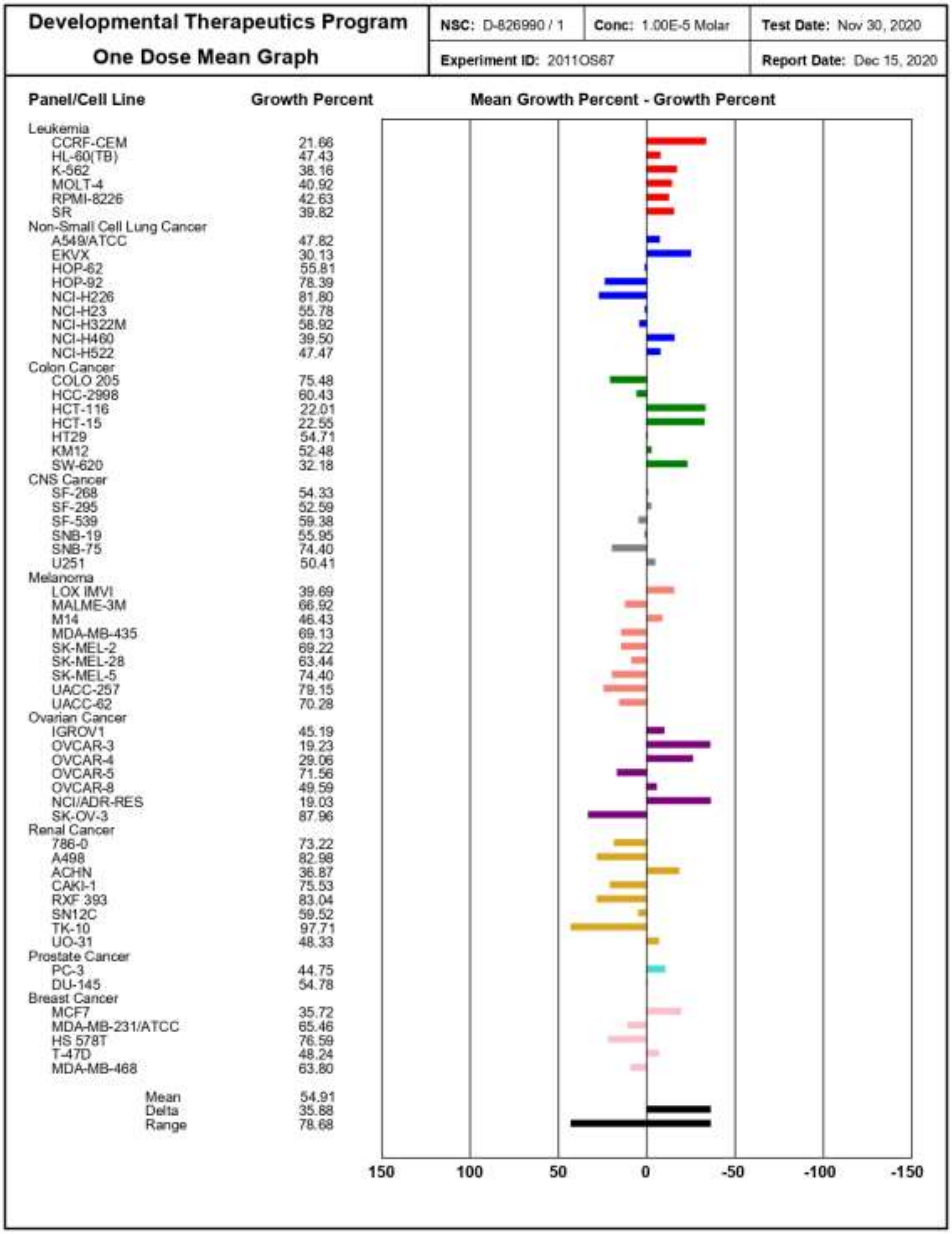

Figure S110. One dose mean graph for compound 7a (NSC 826990) at 10 μM

Information Classification: General

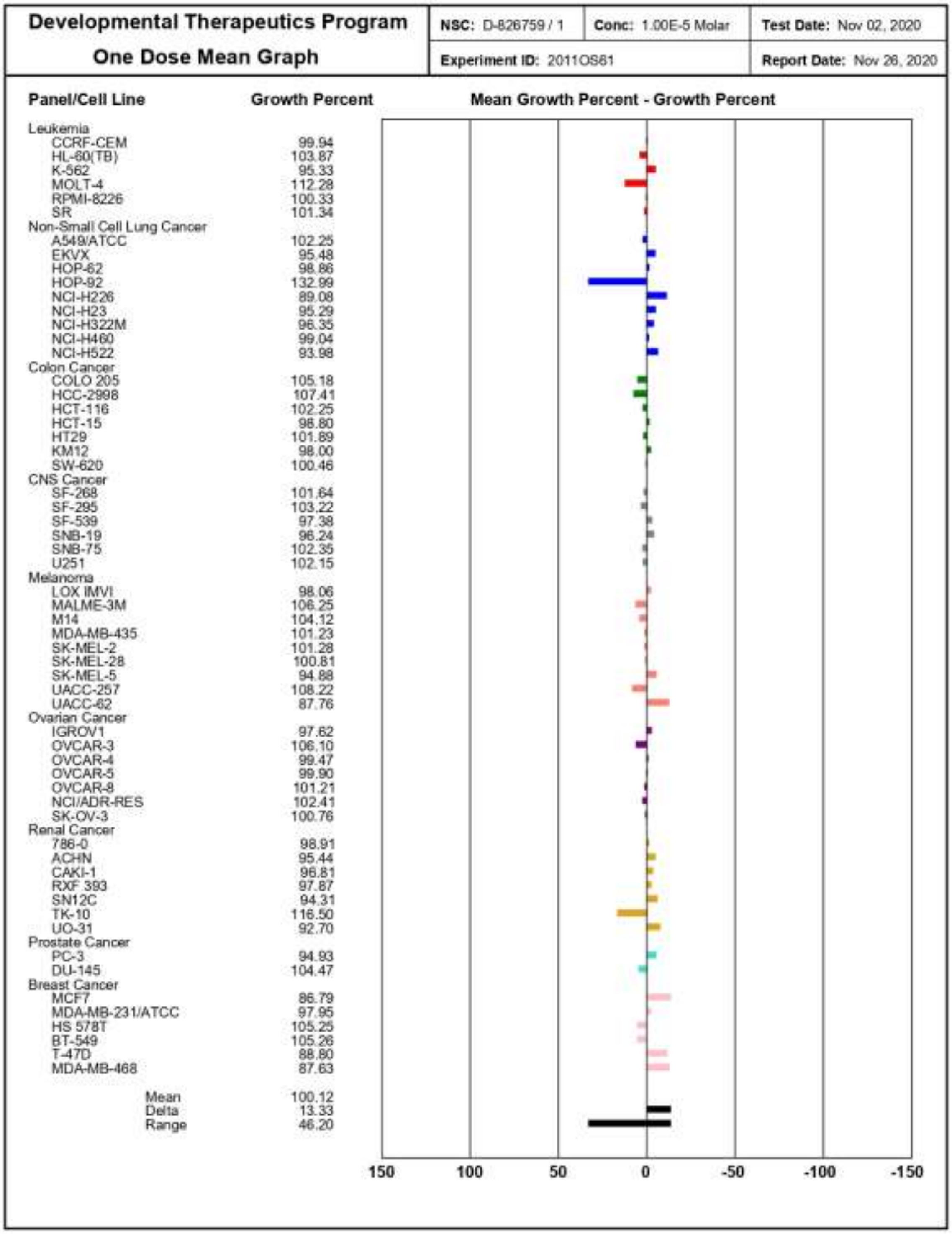

Figure S111. One dose mean graph for compound 7b (NSC 826759) at 10 μM

Information Classification: General

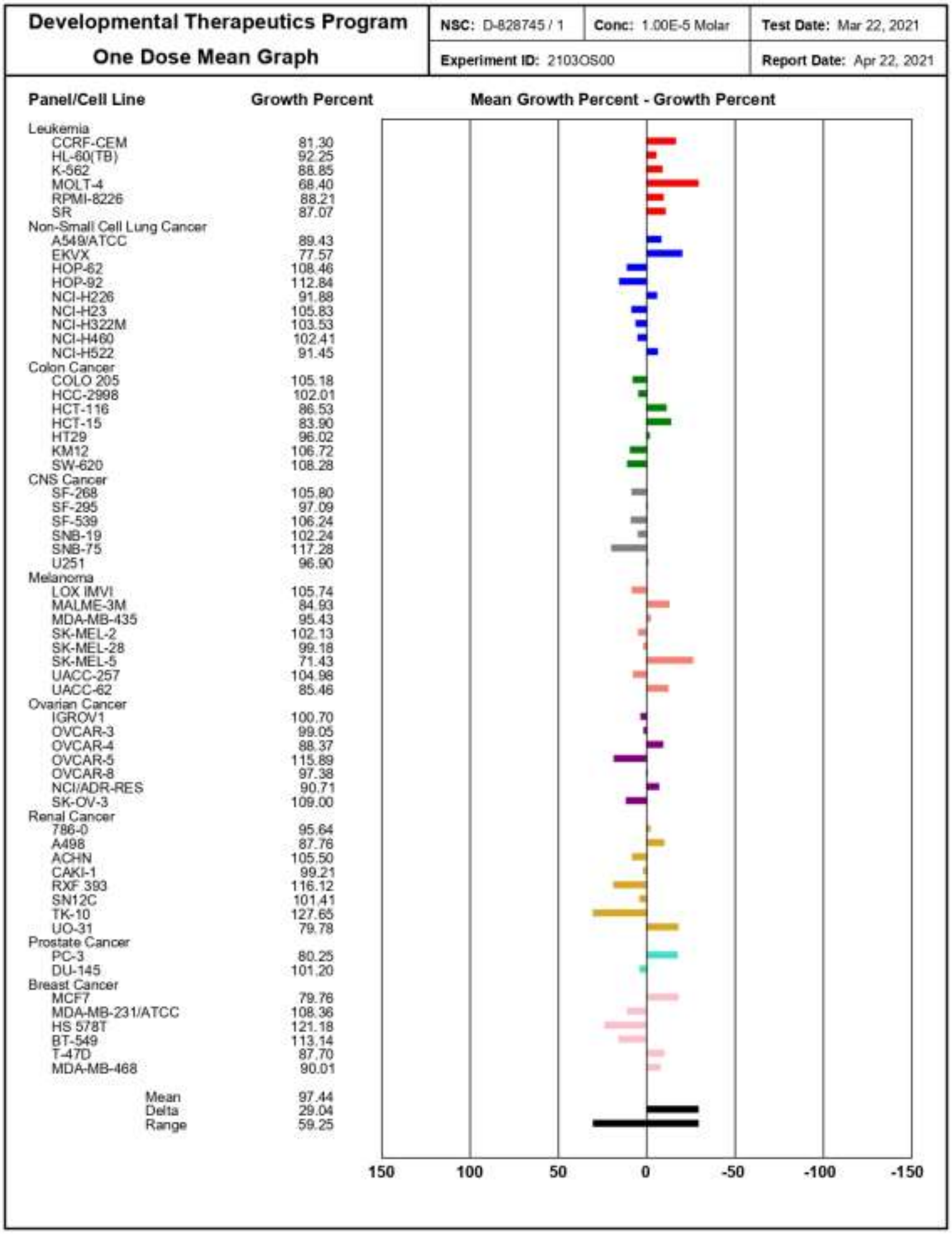

Figure S112. One dose mean graph for compound 7c (NSC 828745) at 10 μM

Information Classification: General

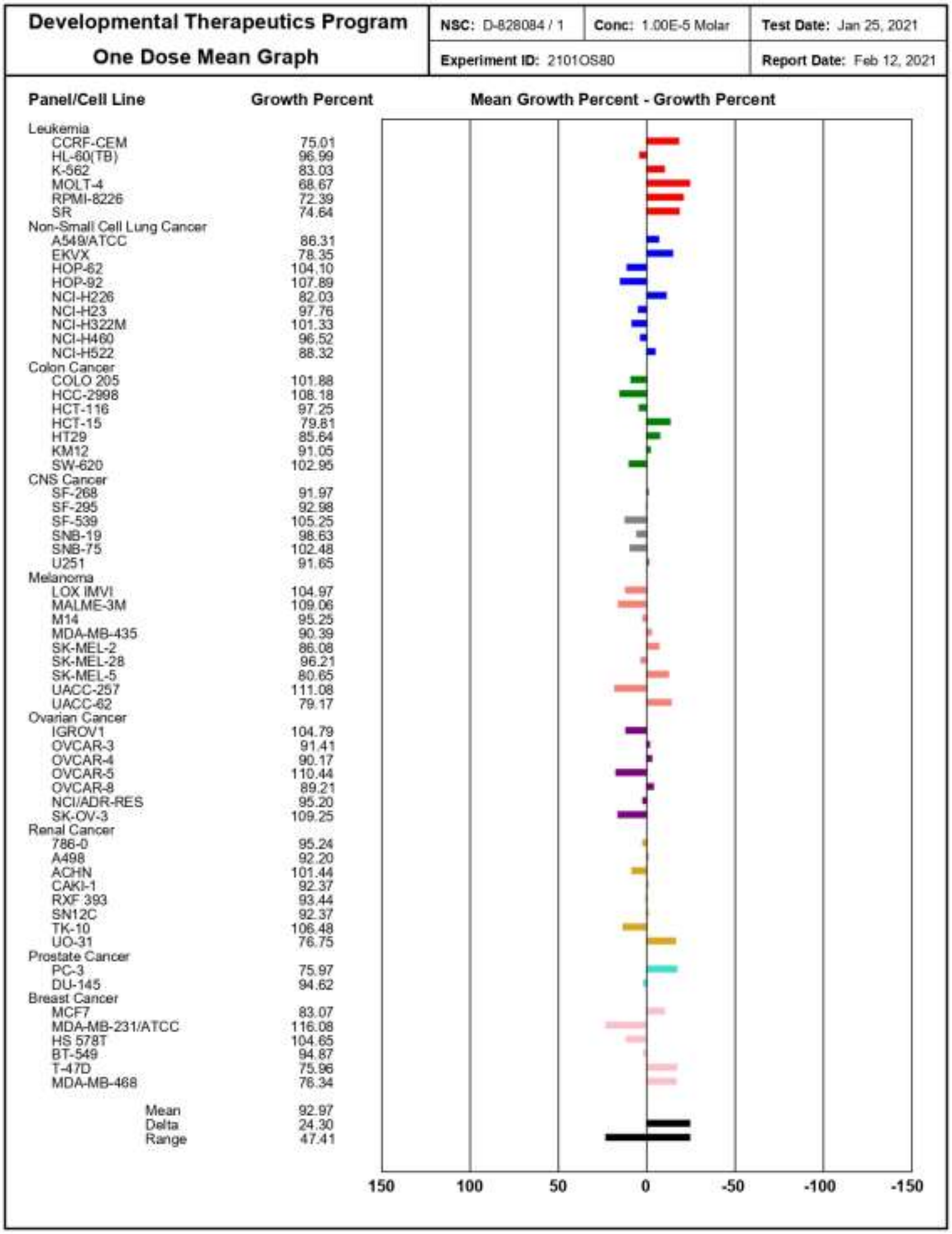

Figure S113. One dose mean graph for compound 7d (NSC 828084) at 10 μM

Information Classification: General

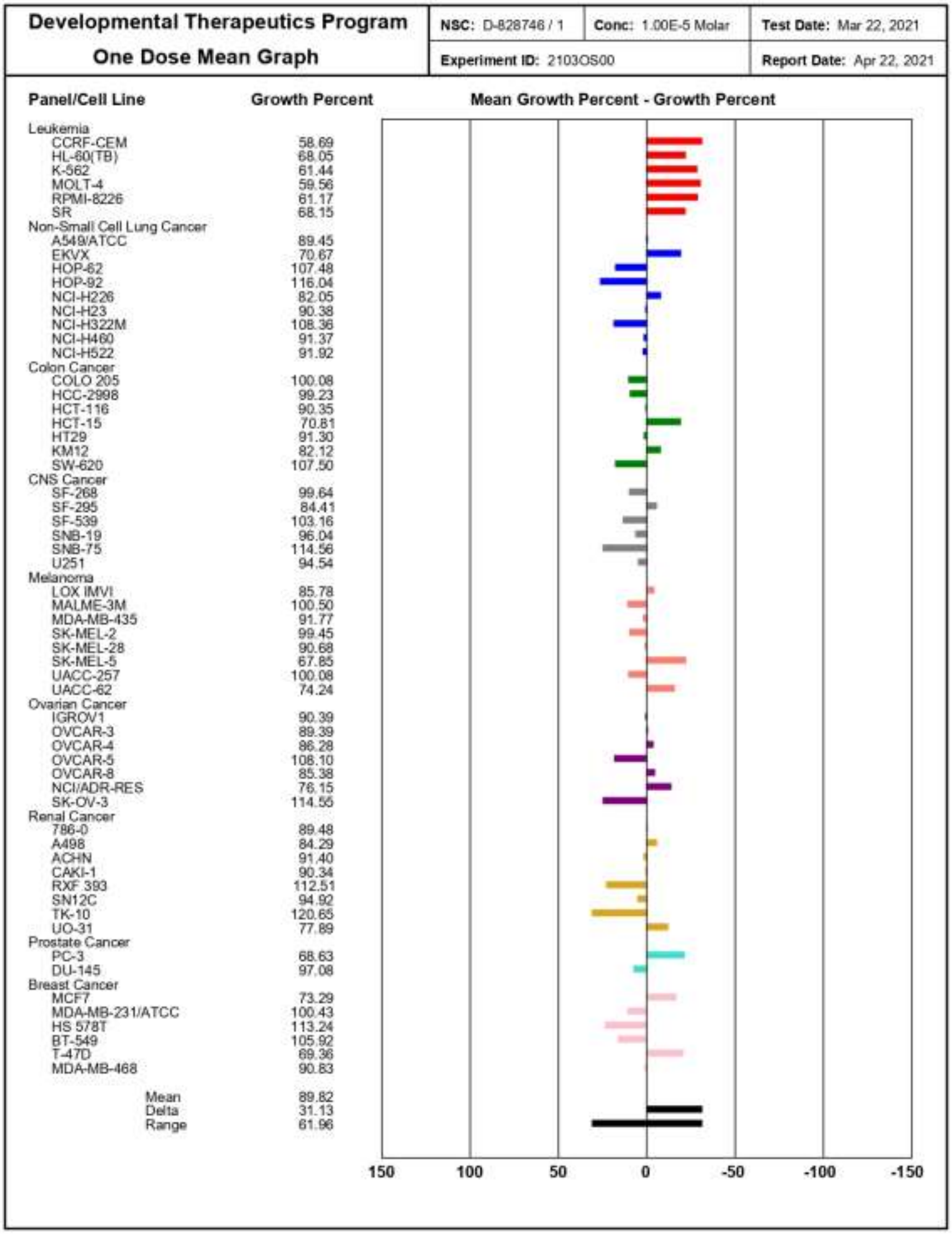

Figure S114. One dose mean graph for compound 7e (NSC 828746) at 10 µM

Information Classification: General

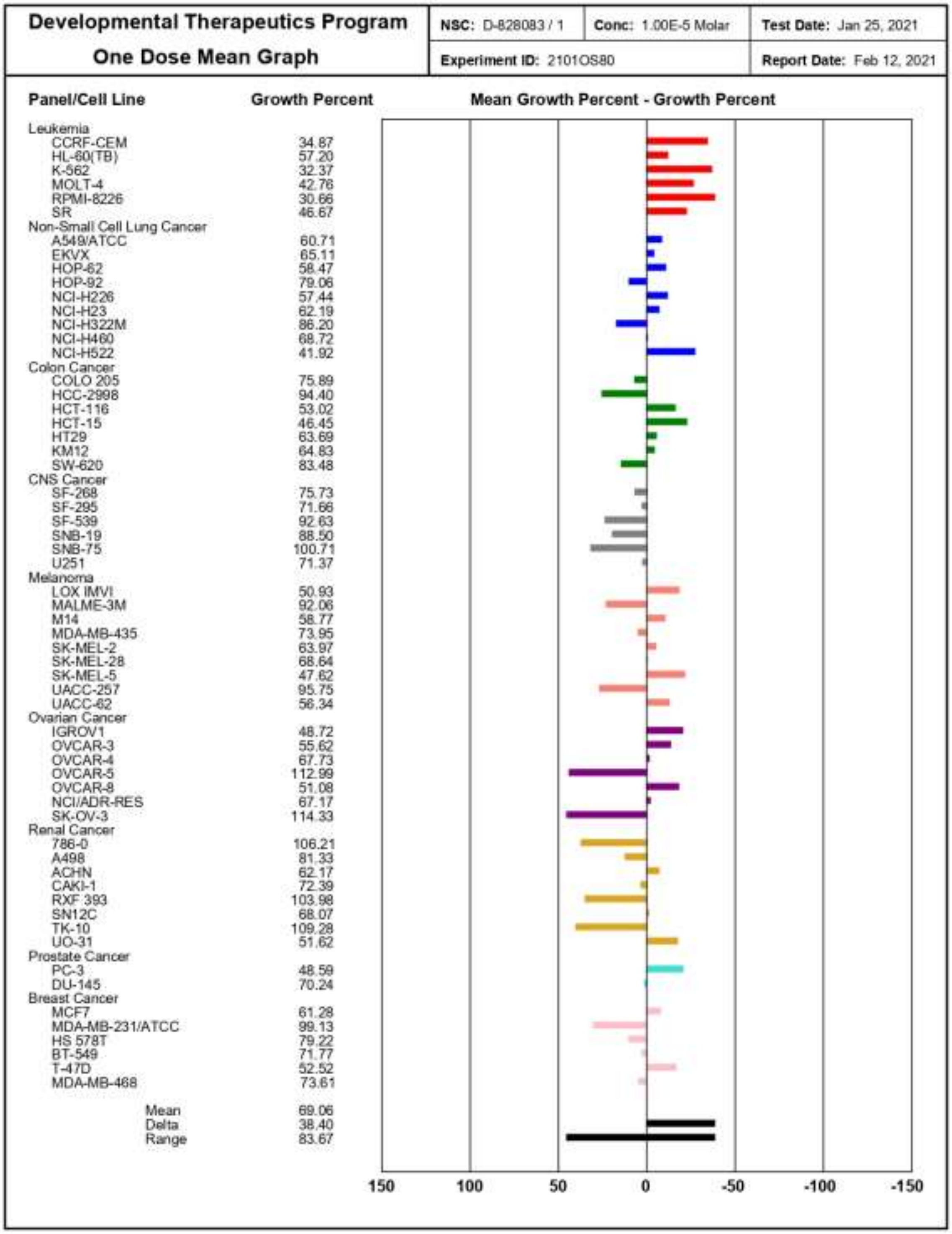

Figure S115. One dose mean graph for compound 7f (NSC 828083) at 10 µM

Information Classification: General

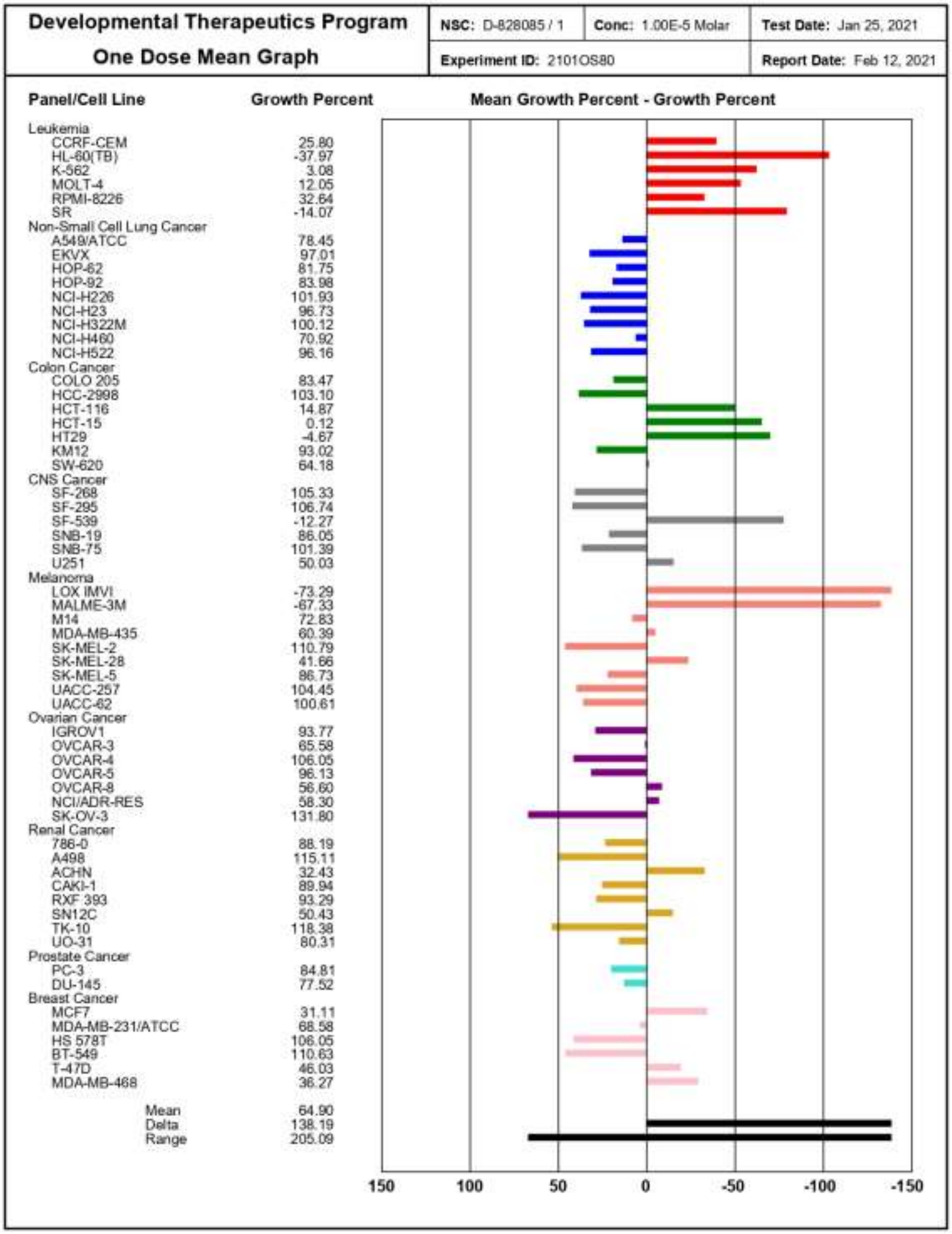

Figure S116. One dose mean graph for compound 7g (NSC 828085) at 10 µM

Information Classification: General

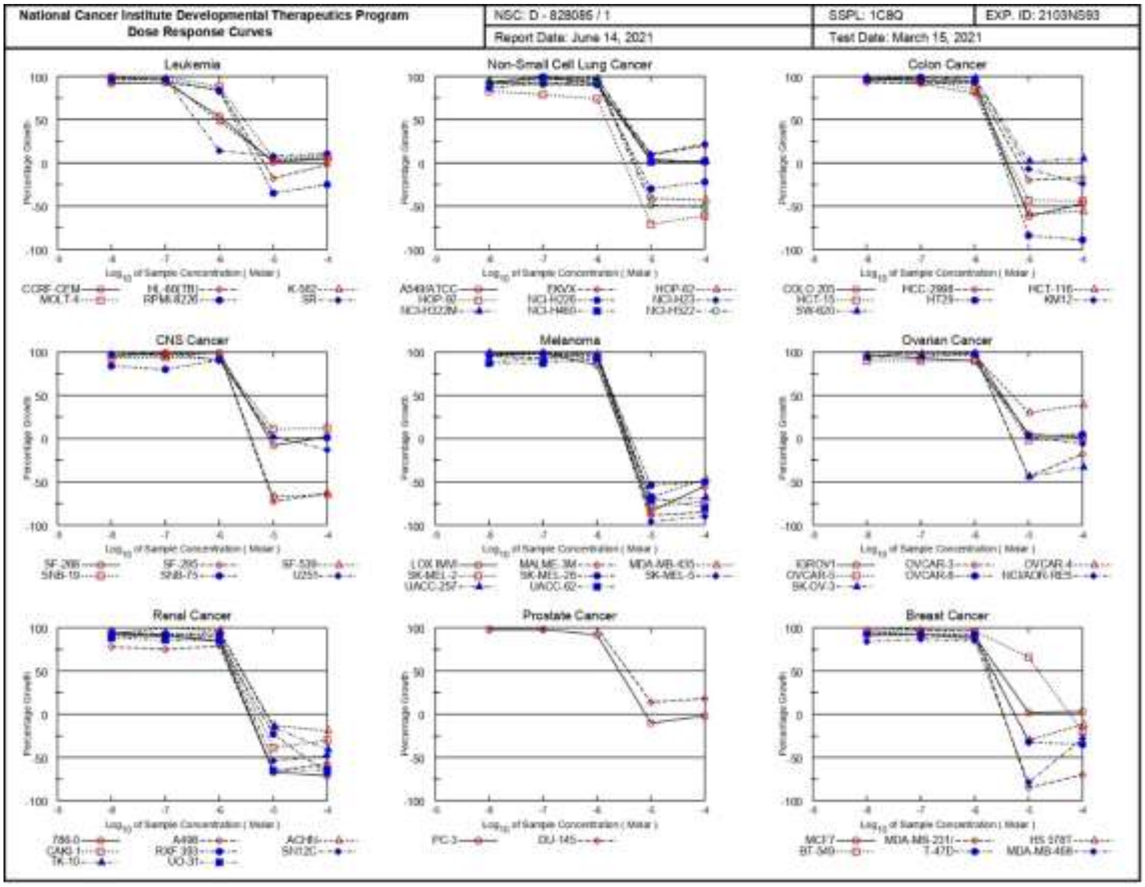

Figure S117. Dose-response curves (% growth versus sample concentration) for all cell lines with different subpanel obtained from the NCI's *in vitro* disease-oriented human cancer cells line for compound 7g on nine types of cancer

Information Classification: General

National Cancer Institute Developmental Therapeutics Program
In-Vitro Testing Results

NSC : D - 828085 / 1	Experiment ID : 2103NS93	Test Type : 08	Units : Molar
Report Date : June 14, 2021	Test Date : March 15, 2021	QNS :	MC :
COMI : AF_18	Stain Reagent : SRB Dual-Pass Related	SSPL : 1C8Q	

			Log10 Concentration												
	Time		Mean Optical Densities					Percent Growth							
Panel/Cell Line	Zero	Ctrl	-8.0	-7.0	-6.0	-5.0	-4.0	-8.0	-7.0	-6.0	-5.0	-4.0	GI50	TGI	LC50
**Leukemia**															
CCRF-CEM	0.362	1.910	1.788	1.805	1.204	0.383	0.442	92	93	54	1	5	1.21E-6	> 1.00E-4	> 1.00E-4
HL-60(TB)	0.544	2.716	2.546	2.567	2.400	0.447	0.533	92	93	85	-18	-2	2.20E-6	6.71E-6	> 1.00E-4
K-562	0.160	1.859	1.819	1.801	0.969	0.210	0.266	98	97	48	3	6	8.95E-7	> 1.00E-4	> 1.00E-4
MOLT-4	0.512	2.756	2.742	2.803	2.493	0.617	0.718	99	102	88	5	9	2.87E-6	> 1.00E-4	> 1.00E-4
RPMI-8226	0.892	2.400	2.347	2.354	2.150	0.584	0.665	96	97	83	-35	-25	1.92E-6	5.10E-6	> 1.00E-4
SR	0.181	1.757	1.731	1.713	0.401	0.305	0.358	98	97	14	8	11	3.69E-7	> 1.00E-4	> 1.00E-4
**Non-Small Cell Lung Cancer**															
A549/ATCC	0.375	2.036	1.915	2.014	1.905	0.436	0.393	93	99	92	4	1	2.99E-6	> 1.00E-4	> 1.00E-4
EKVX	0.568	1.859	1.766	1.747	1.734	0.684	0.821	93	91	90	9	20	3.13E-6	> 1.00E-4	> 1.00E-4
HOP-62	0.687	2.260	2.170	2.191	2.211	0.406	0.389	94	96	97	-41	-43	2.19E-6	5.05E-6	> 1.00E-4
HOP-92	1.363	1.964	1.859	1.835	1.809	0.399	0.533	83	79	74	-71	-61	1.47E-6	3.25E-6	7.19E-6
NCI-H226	0.665	1.633	1.509	1.560	1.548	0.465	0.519	87	92	91	-30	-22	2.19E-6	5.64E-6	> 1.00E-4
NCI-H23	0.633	2.377	2.275	2.336	2.335	0.806	1.020	94	98	98	10	22	3.49E-6	> 1.00E-4	> 1.00E-4
NCI-H322M	0.610	1.807	1.706	1.714	1.692	0.637	0.633	92	92	90	2	2	2.87E-6	> 1.00E-4	> 1.00E-4
NCI-H460	0.363	3.181	3.197	3.140	3.197	0.381	0.417	101	99	101	1	2	3.21E-6	> 1.00E-4	> 1.00E-4
NCI-H522	0.707	2.225	2.114	2.093	2.158	0.358	0.347	93	91	96	-49	-51	2.06E-6	4.56E-6	2.40E-5
**Colon Cancer**															
COLO 205	0.538	2.069	2.033	1.996	1.957	0.206	0.287	98	95	93	-62	-47	1.89E-6	3.98E-6	
HCC-2998	0.856	2.934	2.787	2.763	2.787	0.687	0.708	93	92	93	-20	-17	2.40E-6	6.67E-6	> 1.00E-4
HCT-116	0.274	2.494	2.379	2.316	2.050	0.112	0.120	95	92	80	-59	-56	1.64E-6	3.75E-6	8.57E-6
HCT-15	0.353	2.437	2.344	2.374	2.116	0.203	0.199	96	97	85	-43	-44	1.87E-6	4.62E-6	> 1.00E-4
HT29	0.187	1.431	1.394	1.416	1.354	0.029	0.021	97	99	94	-84	-89	1.76E-6	3.36E-6	6.40E-6
KM12	0.462	2.431	2.388	2.531	2.383	0.428	0.353	98	105	98	-7	-24	2.84E-6	8.49E-6	> 1.00E-4
SW-620	0.285	2.304	2.255	2.253	2.272	0.323	0.383	98	97	98	2	5	3.17E-6	> 1.00E-4	> 1.00E-4
**CNS Cancer**															
SF-268	0.738	2.414	2.353	2.390	2.389	0.681	0.770	96	99	99	-8	2	2.86E-6		> 1.00E-4
SF-295	0.615	2.783	2.702	2.736	2.797	0.165	0.226	96	98	101	-73	-63	1.96E-6	3.79E-6	7.36E-6
SF-539	0.590	2.179	2.187	2.095	2.173	0.198	0.207	101	95	100	-67	-65	1.99E-6	3.97E-6	7.93E-6
SNB-19	0.555	2.159	2.047	2.056	2.036	0.737	0.755	93	94	92	11	12	3.33E-6	> 1.00E-4	> 1.00E-4
SNB-75	1.549	2.644	2.470	2.428	2.545	1.542	1.556	84	80	91		1	2.80E-6		> 1.00E-4
U251	0.273	1.622	1.591	1.638	1.492	0.306	0.237	98	101	90	2	-13	2.88E-6	1.43E-5	> 1.00E-4
**Melanoma**															
LOX IMVI	0.220	1.613	1.565	1.594	1.404	0.036	0.100	97	99	85	-84	-55	1.61E-6	3.19E-6	6.30E-6
MALME-3M	0.680	1.438	1.403	1.384	1.448	0.074	0.104	95	93	101	-89	-85	1.86E-6	3.40E-6	6.23E-6
MDA-MB-435	0.460	2.279	2.262	2.267	2.239	0.148	0.243	99	99	98	-68	-47	1.94E-6	3.89E-6	
SK-MEL-2	1.100	2.385	2.436	2.434	2.453	0.229	0.297	104	104	105	-79	-73	1.99E-6	3.72E-6	6.95E-6
SK-MEL-28	0.641	2.177	2.118	2.144	2.099	0.296	0.319	96	98	95	-54	-50	2.00E-6	4.35E-6	9.43E-6
SK-MEL-5	0.936	3.205	3.188	3.158	3.099	0.037	0.098	99	98	95	-96	-90	1.73E-6	3.15E-6	5.74E-6
UACC-257	0.800	2.163	2.010	2.060	2.072	0.225	0.259	89	92	93	-72	-68	1.83E-6	3.67E-6	7.37E-6
UACC-62	0.648	2.549	2.296	2.299	2.403	0.211	0.130	87	87	92	-68	-80	1.84E-6	3.78E-6	7.77E-6
**Ovarian Cancer**															
IGROV1	0.377	1.728	1.682	1.638	1.591	0.447	0.411	97	93	90	5	2	2.96E-6	> 1.00E-4	> 1.00E-4
OVCAR-3	0.370	1.469	1.536	1.495	1.493	0.209	0.304	106	102	102	-44	-18	2.28E-6	5.03E-6	> 1.00E-4
OVCAR-4	0.814	2.462	2.483	2.425	2.401	1.313	1.461	101	98	96	30	39	5.03E-6	> 1.00E-4	> 1.00E-4
OVCAR-5	0.544	1.683	1.566	1.571	1.571	0.536	0.553	90	90	90	-2	1	2.74E-6		> 1.00E-4
OVCAR-8	0.435	2.068	2.061	2.093	2.031	0.492	0.516	100	102	98	3	5	3.21E-6	> 1.00E-4	> 1.00E-4
NCI/ADR-RES	0.413	1.598	1.537	1.614	1.587	0.434	0.389	95	101	99	2	-6	3.19E-6	1.70E-5	> 1.00E-4
SK-OV-3	0.815	1.897	1.837	1.844	1.871	0.455	0.547	94	95	98	-44	-33	2.17E-6	4.88E-6	> 1.00E-4
**Renal Cancer**															
786-0	0.677	2.611	2.467	2.422	2.316	0.225	0.195	93	90	85	-67	-71	1.69E-6	3.62E-6	7.74E-6
A498	1.406	2.309	2.106	2.079	2.117	0.485	0.600	78	75	79	-66	-57	1.58E-6	3.51E-6	7.81E-6
ACHN	0.287	1.483	1.404	1.499	1.446	0.251	0.232	93	101	97	-13	-19	2.68E-6	7.68E-6	> 1.00E-4
CAKI-1	0.639	2.298	2.204	2.166	2.158	0.390	0.447	94	92	92	-39	-30	2.08E-6	5.02E-6	> 1.00E-4
RXF 393	1.342	1.812	1.762	1.777	1.744	1.031	0.429	89	92	85	-23	-68	2.12E-6	6.12E-6	3.96E-5
SN12C	0.472	1.980	1.882	1.881	1.809	0.217	0.246	93	93	89	-54	-48	1.87E-6	4.18E-6	
TK-10	0.966	2.418	2.347	2.317	2.336	0.820	0.570	95	93	94	-15	-41	2.54E-6	7.28E-6	> 1.00E-4
UO-31	0.883	2.404	2.215	2.185	2.181	0.311	0.319	88	86	85	-65	-64	1.72E-6	3.70E-6	7.97E-6
**Prostate Cancer**															
PC-3	0.650	2.599	2.555	2.559	2.439	0.583	0.640	98	98	92	-10	-2	2.56E-6	7.91E-6	> 1.00E-4
DU-145	0.329	1.651	1.756	1.686	1.686	0.514	0.569	108	103	103	14	18	3.92E-6	> 1.00E-4	> 1.00E-4
**Breast Cancer**															
MCF7	0.470	2.651	2.450	2.476	2.435	0.521	0.541	91	92	90	2	3	2.86E-6	> 1.00E-4	> 1.00E-4
MDA-MB-231/ATCC	0.569	1.594	1.624	1.570	1.556	0.085	0.172	103	98	96	-85	-70	1.80E-6	3.40E-6	6.41E-6
HS 578T	1.354	2.281	2.249	2.203	2.165	0.951	1.189	96	92	87	-30	-12	2.09E-6	5.57E-6	> 1.00E-4
BT-549	1.246	2.339	2.301	2.300	2.295	1.971	0.996	96	96	96	66	-20	1.55E-5	5.86E-5	> 1.00E-4
T-47D	1.019	2.329	2.232	2.220	2.219	0.698	0.666	93	92	92	-32	-35	2.18E-6	5.54E-6	> 1.00E-4
MDA-MB-468	0.954	2.164	1.973	2.012	1.984	0.205	0.684	84	87	85	-79	-28	1.64E-6	3.31E-6	

Figure S118. Values of log molar concentration of response parameters (log$_{10}$ GI$_{50}$, log$_{10}$ TGI & log$_{10}$ LC$_{50}$) for compound 7g

Information Classification: General

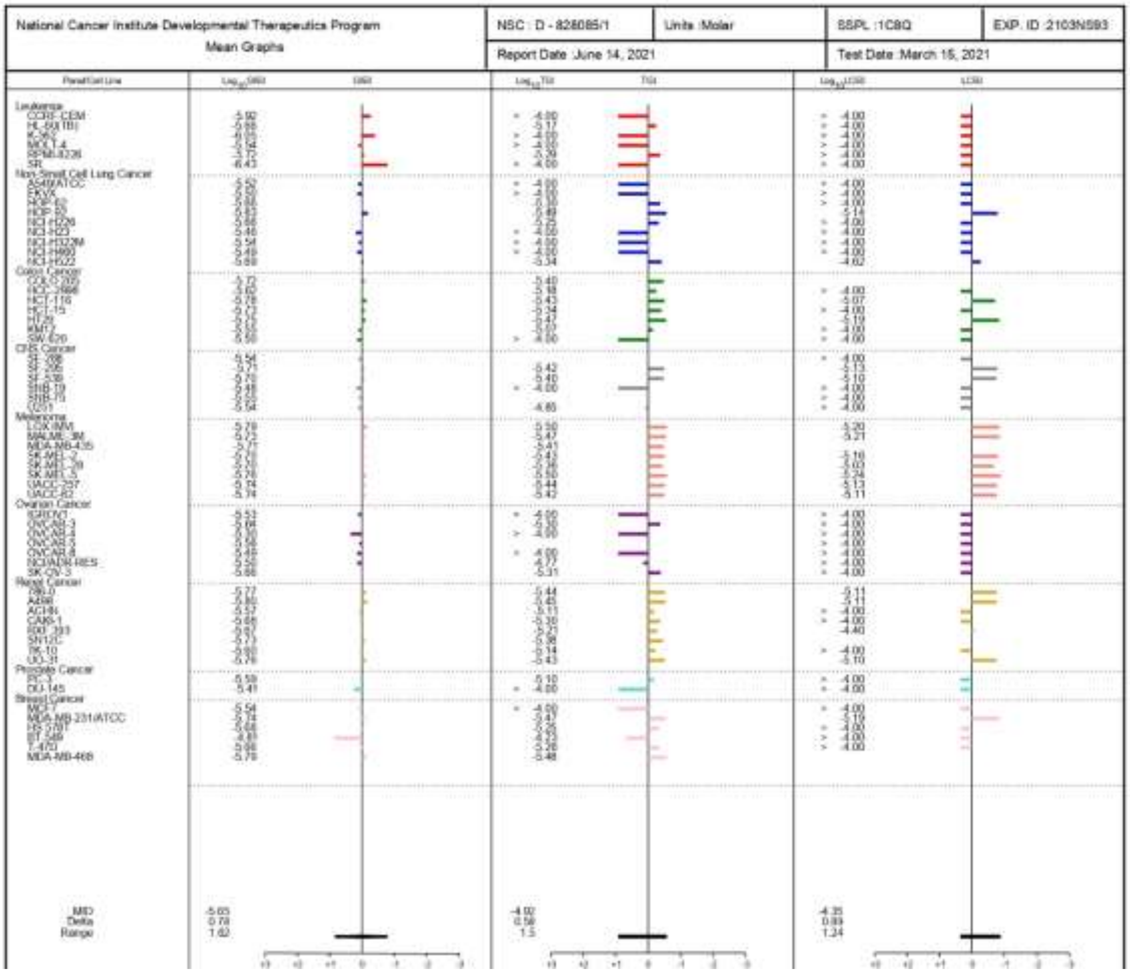

Figure S119. Mean Graphs of the log_{10} values (Molar) of GI_{50}, TGI, and LC_{50} obtained from the NCI 60 cell line experiments for compound 7g

Information Classification: General

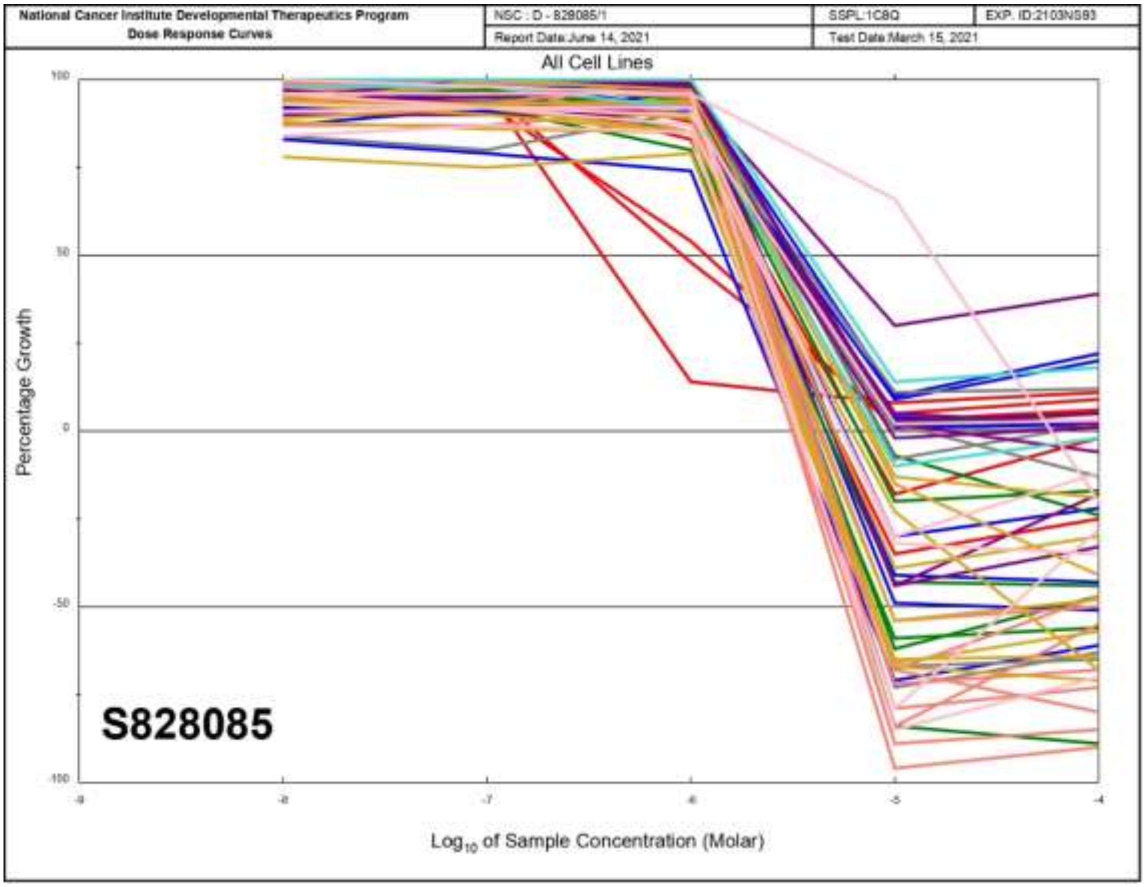

Figure S120. Dose-response curves for all cell lines in the NCI60 panel exposed compound 6d with tissue originated colors and shapes.

Information Classification: General

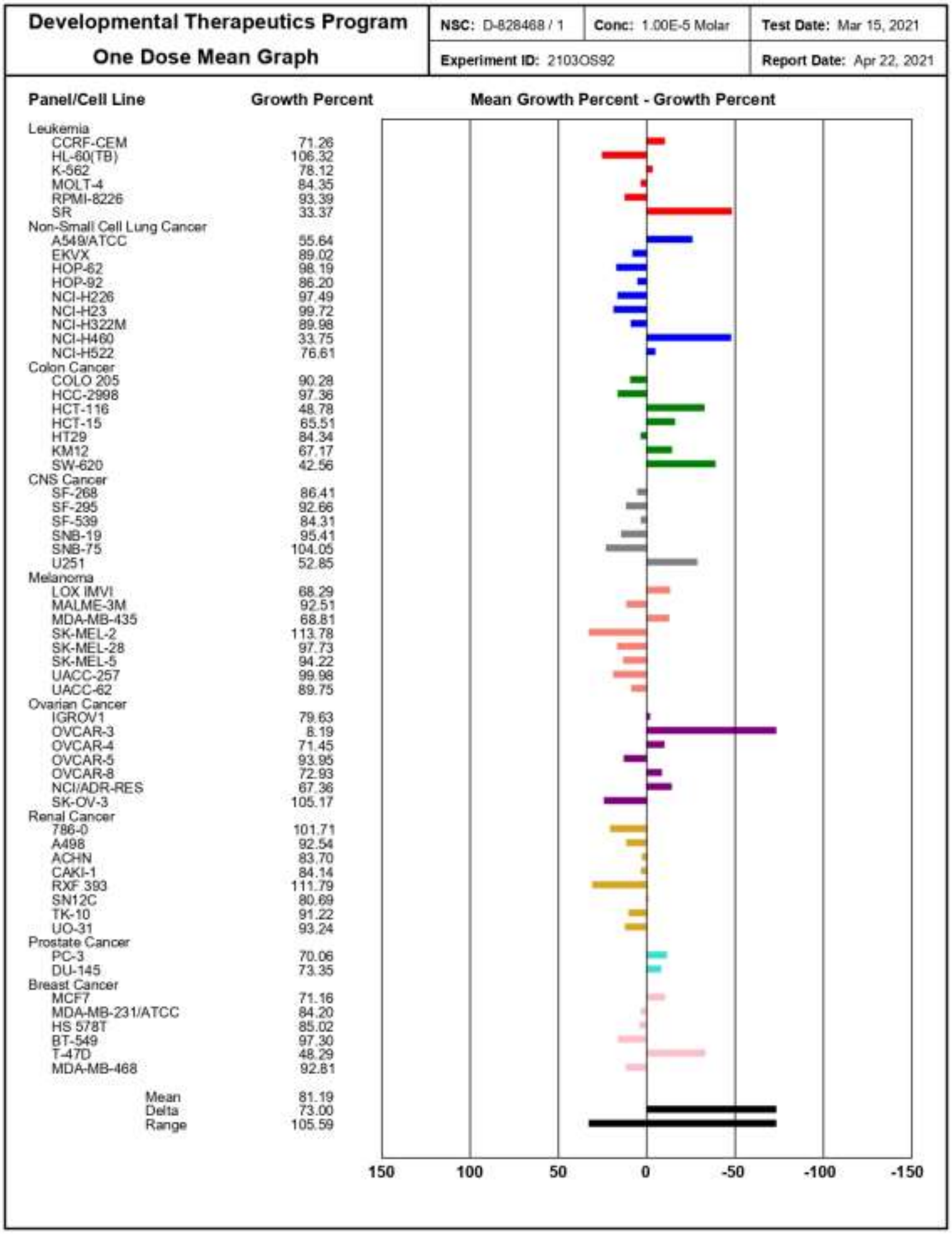

Figure S121. One dose mean graph for compound 7h (NSC 828468) at 10 µM

Information Classification: General

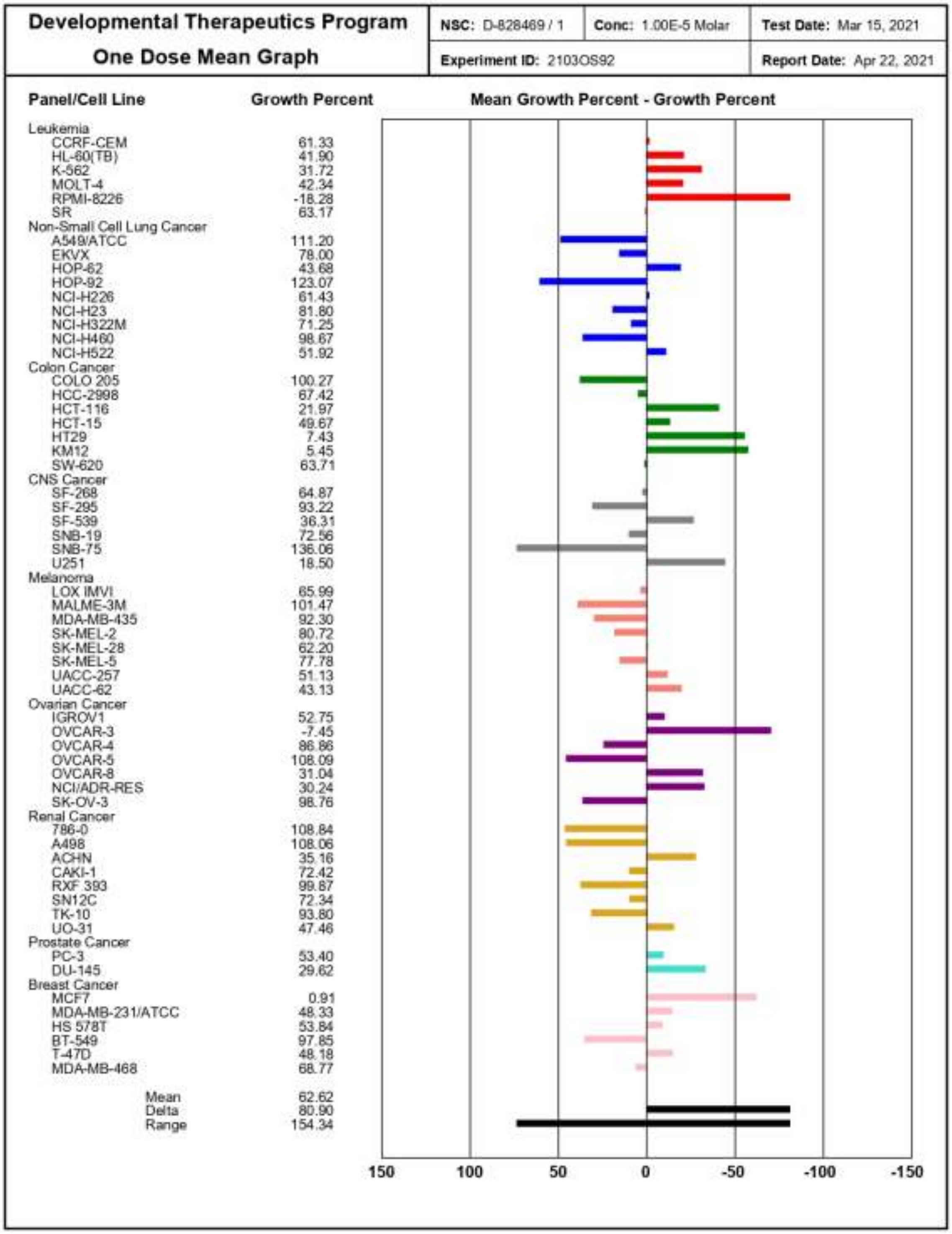

Figure S122. One dose mean graph for compound 7i (NSC 828469) at 10 µM

Information Classification: General

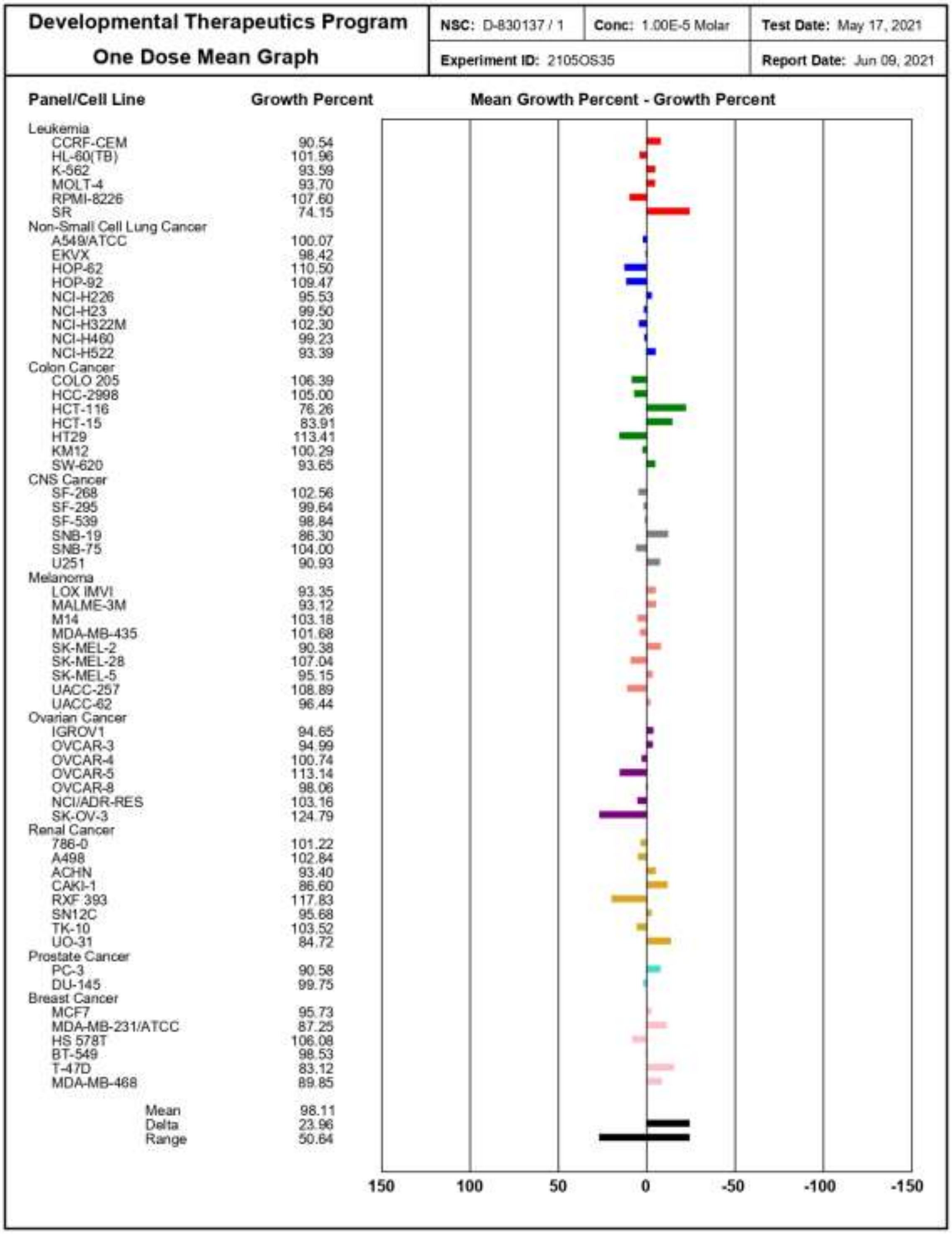

Figure S123. One dose mean graph for compound 7j (NSC 830137) at 10 μM

Information Classification: General

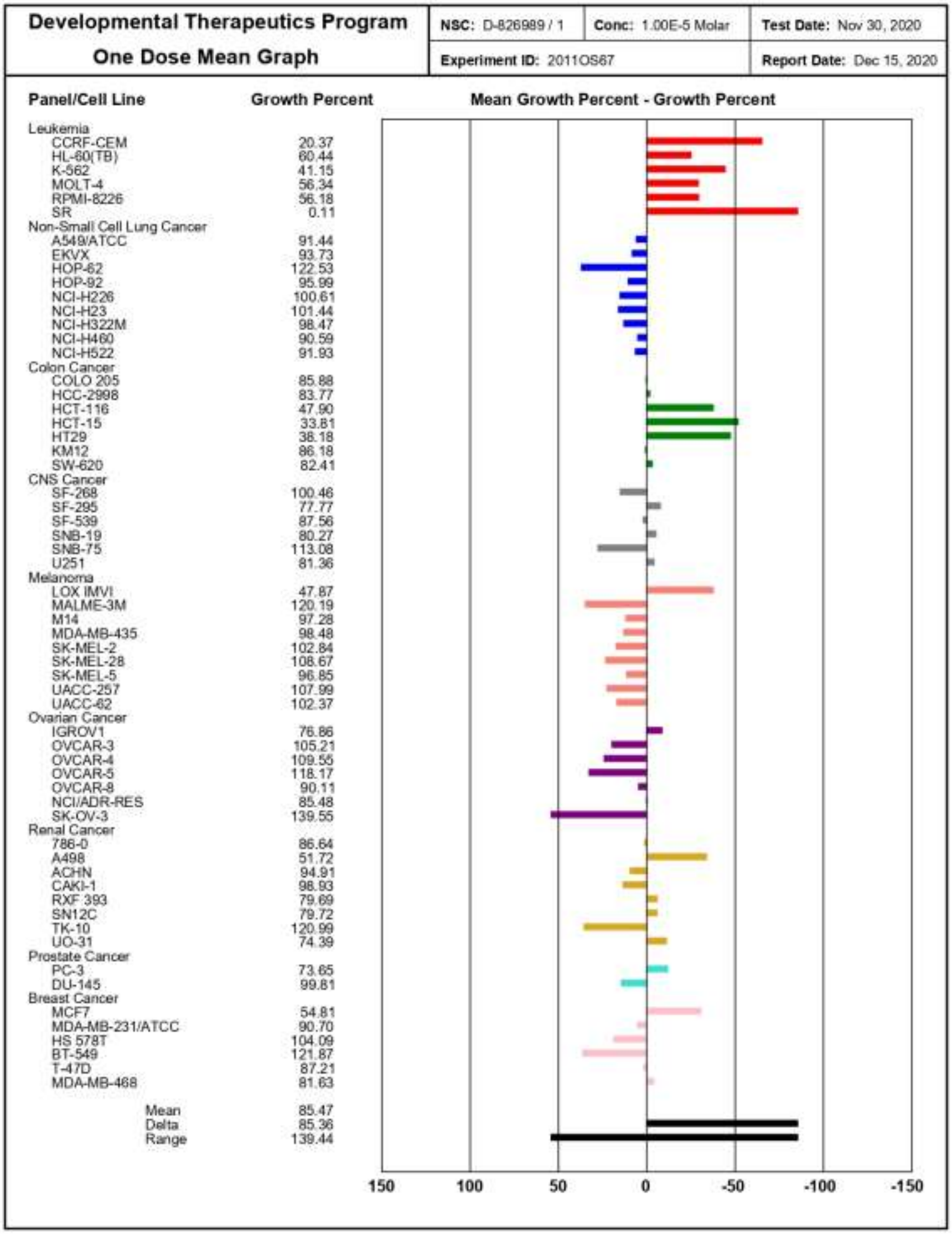

Figure S124. One dose mean graph for compound 7k (NSC 826989) at 10 µM

Information Classification: General

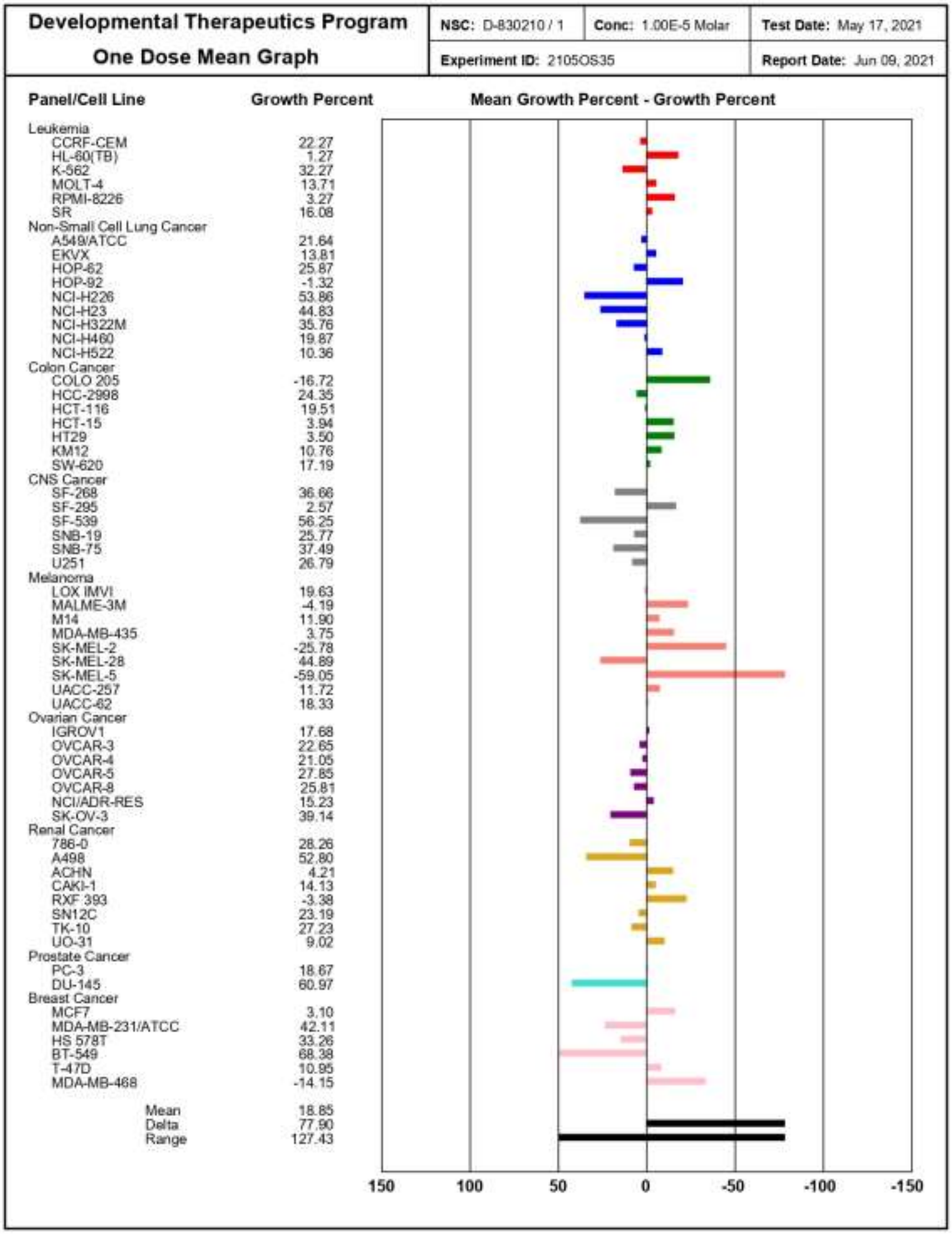

Figure S125. One dose mean graph for compound 7l (NSC 830210) at 10 μM

Information Classification: General

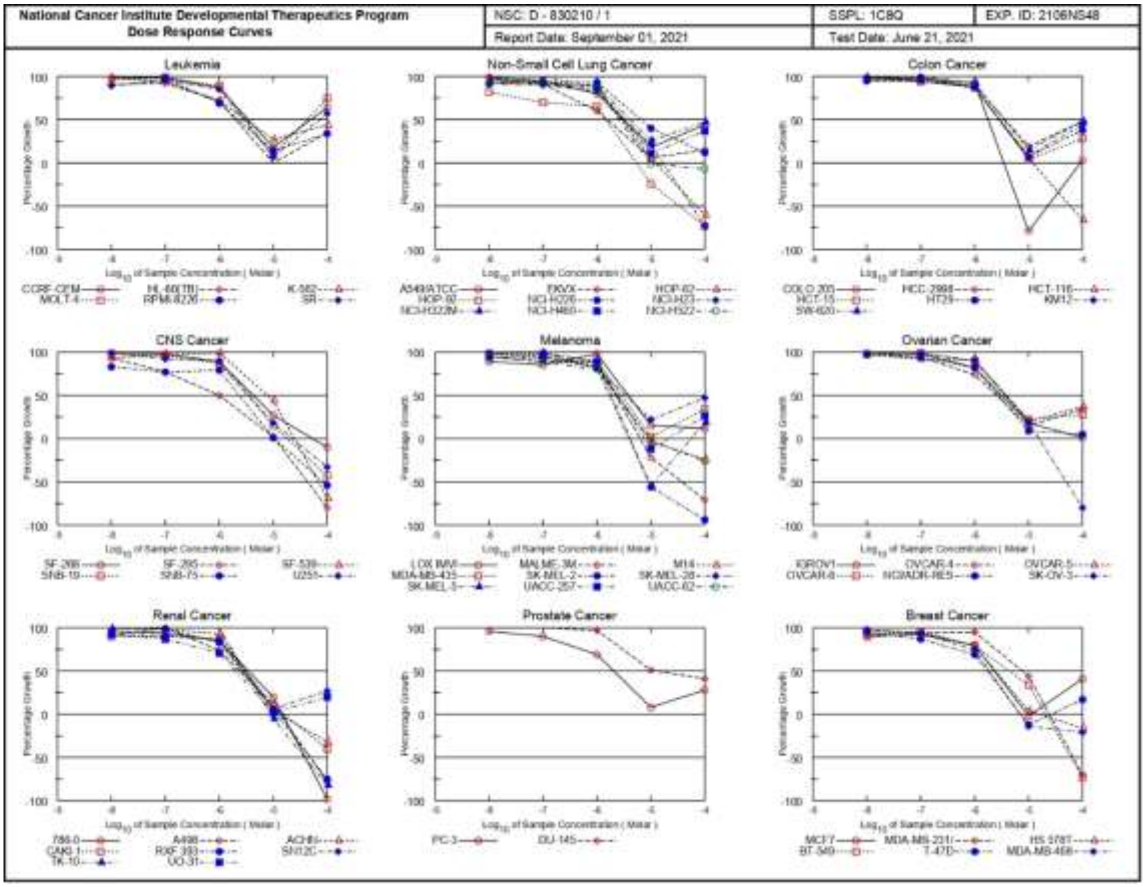

Figure S126. Dose-response curves (% growth versus sample concentration) for all cell lines with different subpanel obtained from the NCI's *in vitro* disease-oriented human cancer cells line for compound 7l on nine types of cancer

Information Classification: General

National Cancer Institute Developmental Therapeutics Program
In-Vitro Testing Results

NSC : D - 830210 / 1	Experiment ID : 2106NS48	Test Type : 08	Units : Molar
Report Date : September 01, 2021	Test Date : June 21, 2021	QNS :	MC :
COMI : AF_32	Stain Reagent : SRB Dual-Pass Related	SSPL : 1C8Q	

			Log10 Concentration												
	Time		Mean Optical Densities					Percent Growth							
Panel/Cell Line	Zero	Ctrl	-8.0	-7.0	-6.0	-5.0	-4.0	-8.0	-7.0	-6.0	-5.0	-4.0	GI50	TGI	LC50
Leukemia															
CCRF-CEM	0.396	2.044	2.007	2.015	1.828	0.685	1.445	98	98	87	18	64		> 1.00E-4	> 1.00E-4
HL-60(TB)	0.355	2.119	1.961	1.987	1.618	0.358	0.973	91	92	72		35	2.00E-6	> 1.00E-4	> 1.00E-4
K-562	0.159	2.006	1.969	1.899	1.481	0.666	0.981	98	94	72	27	44	3.08E-6	> 1.00E-4	> 1.00E-4
MOLT-4	0.444	2.690	2.746	2.656	2.441	0.651	2.139	103	99	89	9	75		> 1.00E-4	> 1.00E-4
RPMI-8226	0.819	3.062	3.095	3.024	2.375	1.144	1.571	101	98	69	14	34	2.26E-6	> 1.00E-4	> 1.00E-4
SR	0.290	1.338	1.225	1.301	1.183	0.364	0.888	89	96	85	7	57		> 1.00E-4	> 1.00E-4
Non-Small Cell Lung Cancer															
A549/ATCC	0.505	2.600	2.579	2.502	2.184	0.878	1.419	99	95	80	18	44	3.04E-6	> 1.00E-4	> 1.00E-4
EKVX	0.924	2.368	2.345	2.236	1.794	1.004	1.141	98	91	60	6	15	1.54E-6	> 1.00E-4	> 1.00E-4
HOP-62	1.064	2.645	2.584	2.557	2.447	1.145	0.425	96	94	88	5	-60	2.85E-6	1.20E-5	7.00E-5
HOP-92	1.347	1.832	1.747	1.689	1.661	1.024	0.368	82	70	65	-24	-73	1.47E-6	5.36E-6	3.42E-5
NCI-H226	1.204	2.837	2.708	2.747	2.725	1.857	1.397	92	94	93	40	12	6.48E-6	> 1.00E-4	> 1.00E-4
NCI-H23	0.694	2.393	2.271	2.230	2.107	1.054	0.188	93	90	83	21	-73	3.42E-6	1.68E-5	5.71E-5
NCI-H322M	0.749	2.147	2.076	2.037	1.994	1.106	1.402	95	92	89	26	47	4.12E-6	> 1.00E-4	> 1.00E-4
NCI-H460	0.276	2.712	2.891	2.821	2.422	0.553	1.188	107	104	88	11	37	3.14E-6	> 1.00E-4	> 1.00E-4
NCI-H522	1.276	3.066	2.911	2.906	2.807	1.261	1.190	91	91	86	-1	-7	2.57E-6	9.68E-6	> 1.00E-4
Colon Cancer															
COLO 205	0.746	2.329	2.324	2.279	2.224	0.154	0.791	100	97	93	-79	3	1.78E-6		
HCC-2998	0.709	2.814	2.716	2.700	2.603	1.117	1.713	95	95	90	19	48	3.68E-6	> 1.00E-4	> 1.00E-4
HCT-116	0.231	1.917	1.895	1.837	1.697	0.320	0.079	99	95	87	5	-66	2.83E-6	1.19E-5	5.99E-5
HCT-15	0.280	2.066	2.017	1.967	1.848	0.351	0.783	97	94	88	4	28	2.82E-6	> 1.00E-4	> 1.00E-4
HT29	0.426	2.468	2.362	2.456	2.198	0.575	1.207	95	99	87	7	38	2.90E-6	> 1.00E-4	> 1.00E-4
KM12	0.582	2.805	2.792	2.698	2.517	0.753	1.551	99	95	87	8	44	2.93E-6	> 1.00E-4	> 1.00E-4
SW-620	0.290	2.094	2.176	2.130	1.946	0.562	1.159	105	102	92	15	48	3.51E-6	> 1.00E-4	> 1.00E-4
CNS Cancer															
SF-268	0.761	2.369	2.369	2.330	2.187	1.193	0.683	100	98	89	27	-10	4.22E-6	5.29E-5	> 1.00E-4
SF-295	0.864	2.783	2.645	2.337	1.828	0.908	0.176	93	77	50	2	-80	1.01E-6	1.07E-5	4.34E-5
SF-539	0.879	2.703	2.581	2.657	2.685	1.673	0.270	93	97	99	44	-69	7.65E-6	2.43E-5	6.75E-5
SNB-19	0.579	1.822	1.800	1.769	1.659	0.765	0.328	98	96	87	15	-43	3.25E-6	1.80E-5	> 1.00E-4
SNB-75	1.972	2.974	2.799	2.739	2.763	1.984	0.906	83	77	79	1	-54	2.36E-6	1.05E-5	8.44E-5
U251	0.514	2.346	2.320	2.195	2.157	0.840	0.347	99	92	90	18	-33	3.56E-6	2.25E-5	> 1.00E-4
Melanoma															
LOX IMVI	0.259	1.819	1.648	1.582	1.790	0.487	0.443	89	85	98	15	12	3.77E-6	> 1.00E-4	> 1.00E-4
MALME-3M	0.727	1.430	1.396	1.355	1.345	0.564	0.212	95	89	88	-22	-71	2.21E-6	6.26E-6	3.71E-5
M14	0.438	1.678	1.650	1.621	1.471	0.421	0.332	98	95	83	-4	-24	2.40E-6	9.00E-6	> 1.00E-4
MDA-MB-435	0.585	2.802	2.749	2.756	2.582	0.635	1.346	98	98	90	2	34	2.86E-6	> 1.00E-4	> 1.00E-4
SK-MEL-2	1.796	2.910	2.884	2.840	2.704	0.787	0.110	98	94	82	-56	-94	1.69E-6	3.91E-6	9.01E-6
SK-MEL-28	0.786	2.247	2.258	2.228	1.990	1.102	1.469	101	99	82	22	47	3.41E-6	> 1.00E-4	> 1.00E-4
SK-MEL-5	0.835	3.327	3.322	3.314	3.063	0.379	1.310	100	99	89	-55	19	1.88E-6		
UACC-257	1.333	2.985	2.887	2.867	2.782	1.172	1.771	94	93	88	-12	26	2.39E-6		> 1.00E-4
UACC-62	0.907	2.740	2.630	2.542	2.382	0.891	0.668	94	89	80	-2	-26	2.35E-6	9.50E-6	> 1.00E-4
Ovarian Cancer															
IGROV1	0.522	2.180	2.271	2.146	1.886	0.817	0.534	105	98	82	18	1	3.16E-6	> 1.00E-4	> 1.00E-4
OVCAR-4	1.042	2.435	2.438	2.348	2.077	1.255	1.519	100	94	74	15	34	2.58E-6	> 1.00E-4	> 1.00E-4
OVCAR-5	0.642	1.795	1.758	1.707	1.694	0.875	1.063	97	92	91	20	37	3.80E-6	> 1.00E-4	> 1.00E-4
OVCAR-8	0.640	2.818	2.786	2.803	2.596	1.128	1.257	98	99	90	22	28	3.89E-6	> 1.00E-4	> 1.00E-4
NCI/ADR-RES	0.681	2.499	2.452	2.399	2.174	0.851	0.781	97	94	82	9	5	2.76E-6	> 1.00E-4	> 1.00E-4
SK-OV-3	0.951	1.916	1.961	1.901	1.824	1.130	0.190	105	98	90	19	-80	3.65E-6	1.54E-5	4.96E-5
Renal Cancer															
786-0	0.753	2.651	2.576	2.531	2.370	1.126	0.017	96	94	85	20	-98	3.44E-6	1.47E-5	3.92E-5
A498	1.444	2.322	2.250	2.333	2.096	1.537	0.319	92	101	74	11	-78	2.40E-6	1.32E-5	4.83E-5
ACHN	0.407	1.822	1.890	1.799	1.725	0.460	0.278	105	98	93	4	-32	3.04E-6	1.28E-5	> 1.00E-4
CAKI-1	0.756	2.227	2.084	2.087	2.038	0.896	0.453	90	90	87	10	-40	3.01E-6	1.55E-5	> 1.00E-4
RXF 393	0.860	2.018	2.099	2.011	1.823	0.916	0.211	107	99	83	5	-75	2.65E-6	1.15E-5	4.82E-5
SN12C	0.795	2.574	2.428	2.421	2.333	0.909	1.270	92	91	86	6	27	2.85E-6	> 1.00E-4	> 1.00E-4
TK-10	1.359	2.156	2.148	2.243	2.345	1.288	0.249	99	111	124	-5	-82	3.73E-6	9.11E-6	3.85E-5
UO-31	0.742	2.283	2.158	2.082	1.835	0.773	1.044	92	87	71	2	20	2.01E-6	> 1.00E-4	> 1.00E-4
Prostate Cancer															
PC-3	0.551	2.175	2.112	2.012	1.673	0.687	1.014	96	90	69	8	28	2.06E-6	> 1.00E-4	> 1.00E-4
DU-145	0.396	1.811	1.929	1.831	1.768	1.113	0.979	108	101	97	51	41	1.17E-5	> 1.00E-4	> 1.00E-4
Breast Cancer															
MCF7	0.627	2.753	2.559	2.587	2.322	0.613	1.492	91	92	80	-2	41	2.30E-6		> 1.00E-4
MDA-MB-231/ATCC	0.580	1.504	1.476	1.451	1.453	0.988	0.175	97	94	95	44	-70	7.65E-6	2.44E-5	6.70E-5
HS 578T	1.323	2.180	2.085	2.128	2.003	1.355	1.113	89	94	79	4	-16	2.44E-6	1.54E-5	> 1.00E-4
BT-549	1.348	2.034	1.985	2.009	1.891	1.583	0.370	93	96	79	34	-73	4.45E-6	2.09E-5	6.15E-5
T-47D	0.959	1.969	1.919	1.835	1.654	0.845	1.130	95	87	69	-12	17	1.71E-6		> 1.00E-4
MDA-MB-468	0.969	1.794	1.774	1.744	1.577	0.831	0.763	98	94	74	-14	-21	1.86E-6	6.89E-6	> 1.00E-4

Figure S127. Values of log molar concentration of response parameters (log$_{10}$ GI$_{50}$, log$_{10}$ TGI & log$_{10}$ LC$_{50}$) for compound 7l

Information Classification: General

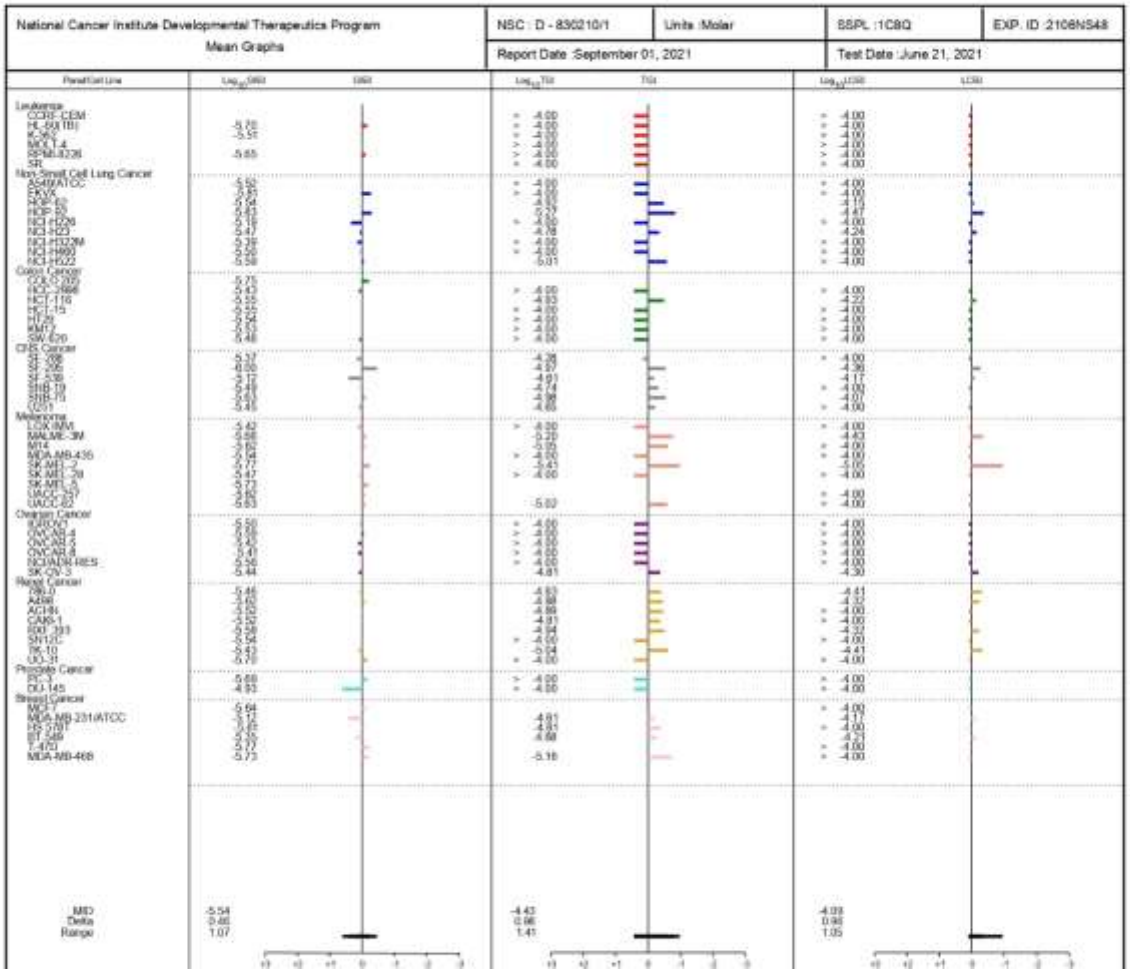

Figure S128. Mean Graphs of the log_{10} values (Molar) of GI_{50}, TGI, and LC_{50} obtained from the NCI 60 cell line experiments for compound 7l

Information Classification: General

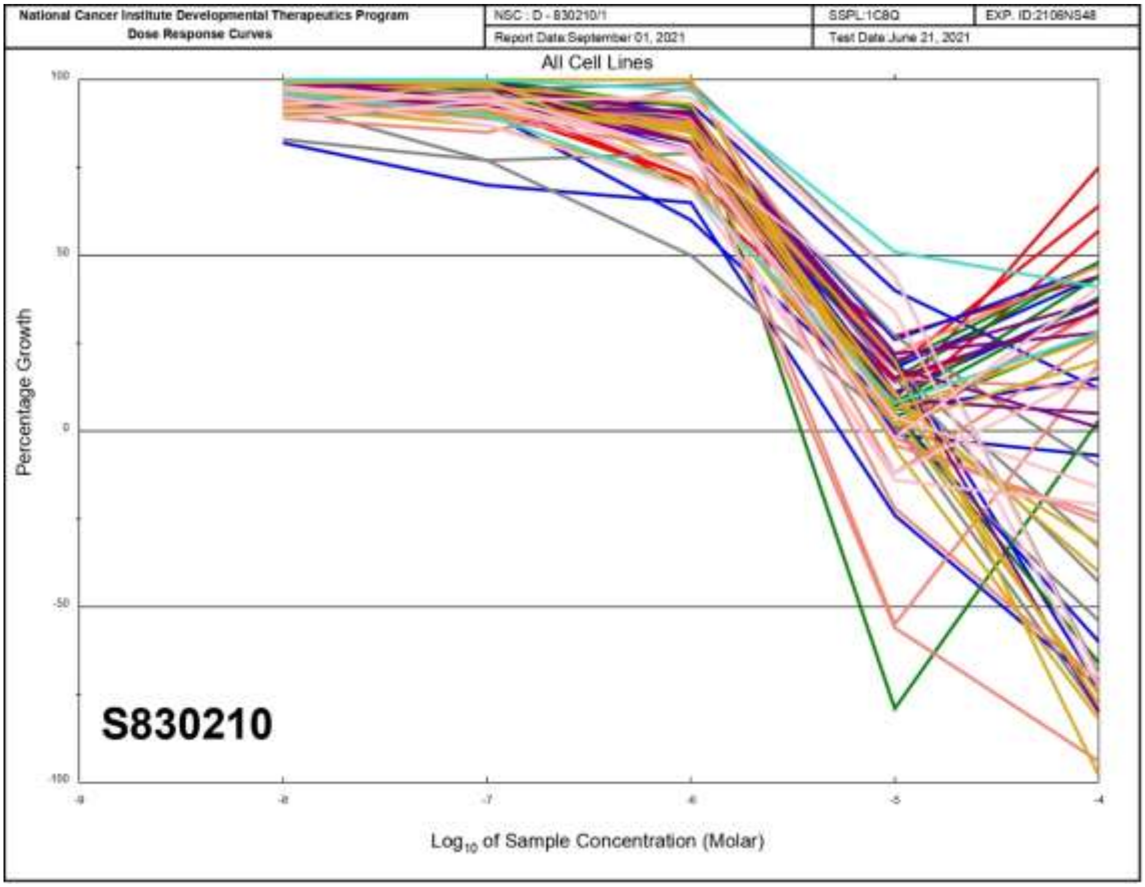

Figure S129. Dose-response curves for all cell lines in the NCI60 panel exposed compound 7l with tissue originated colors and shapes.

Information Classification: General

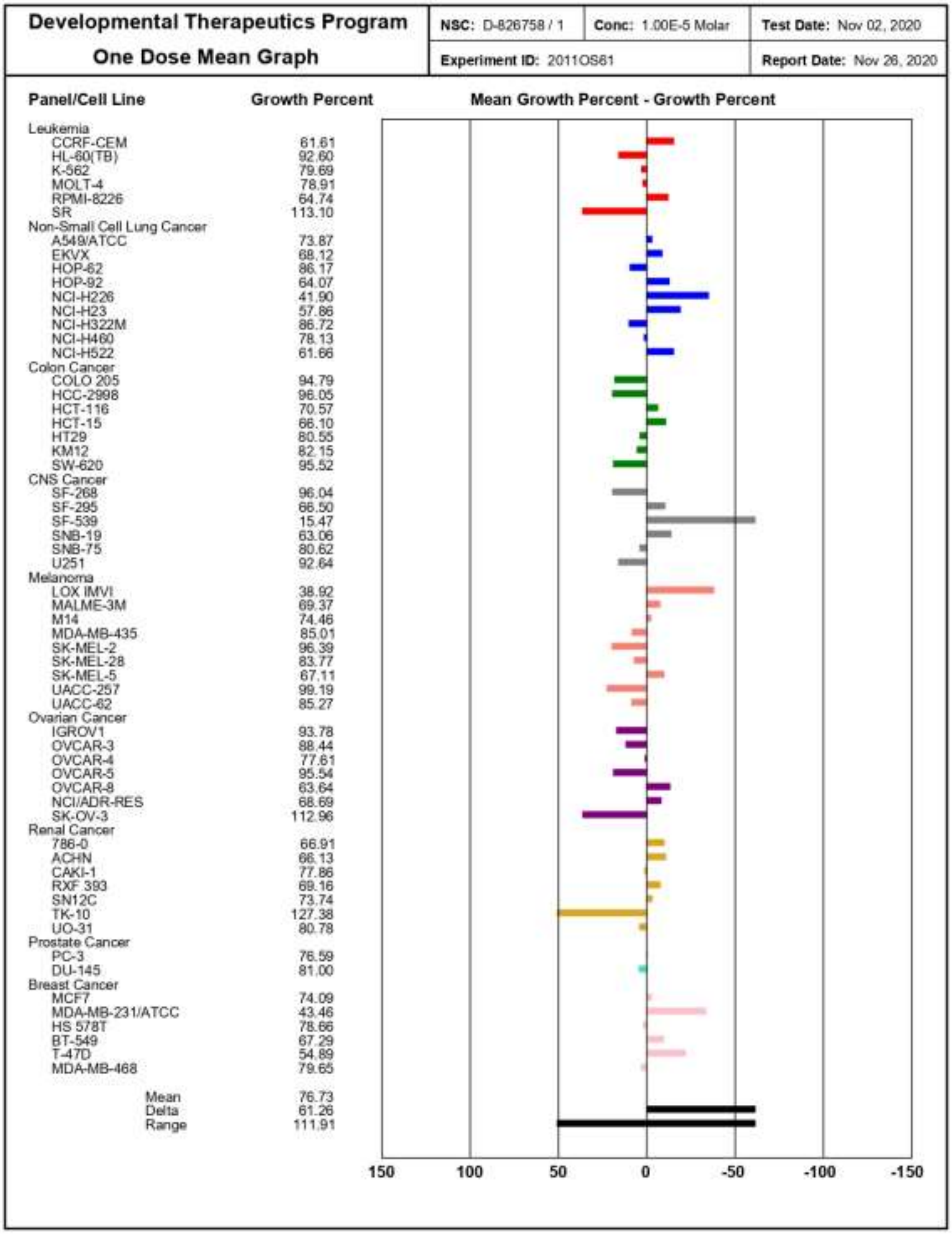

Figure S130. One dose mean graph for compound 8a (NSC 826758) at 10 µM

Information Classification: General

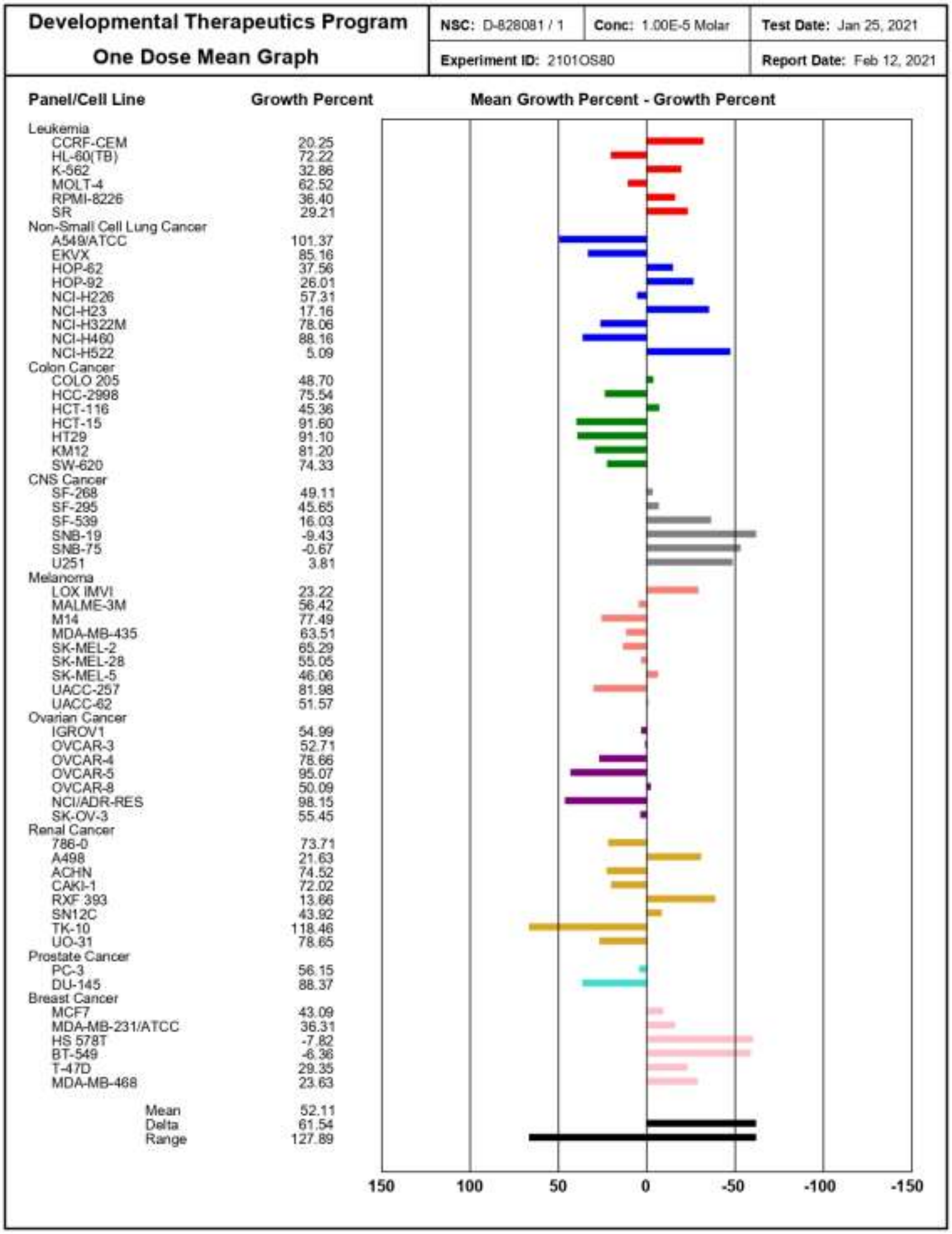

Figure S131. One dose mean graph for compound 8b (NSC 828081) at 10 µM

Information Classification: General

Developmental Therapeutics Program<br>One Dose Mean Graph	NSC: D-828470 / 1	Conc: 1.00E-5 Molar	Test Date: Mar 15, 2021
	Experiment ID: 2103OS92		Report Date: Apr 22, 2021

Panel/Cell Line	Growth Percent
**Leukemia**	
CCRF-CEM	104.54
HL-60(TB)	102.51
K-562	91.58
MOLT-4	101.03
RPMI-8226	98.53
SR	100.37
**Non-Small Cell Lung Cancer**	
A549/ATCC	108.33
EKVX	104.54
HOP-62	97.34
HOP-92	118.54
NCI-H226	101.61
NCI-H23	106.51
NCI-H322M	97.43
NCI-H460	106.88
NCI-H522	90.98
**Colon Cancer**	
COLO 205	104.56
HCC-2998	109.86
HCT-116	99.19
HCT-15	106.16
HT29	100.61
KM12	99.70
SW-620	111.66
**CNS Cancer**	
SF-268	102.56
SF-295	107.74
SF-539	98.01
SNB-19	103.58
SNB-75	90.49
U251	105.23
**Melanoma**	
LOX IMVI	105.03
MALME-3M	104.87
MDA-MB-435	104.76
SK-MEL-2	110.96
SK-MEL-28	108.72
SK-MEL-5	98.68
UACC-257	103.35
UACC-62	90.18
**Ovarian Cancer**	
IGROV1	98.62
OVCAR-3	111.08
OVCAR-4	106.53
OVCAR-5	106.25
OVCAR-8	99.60
NCI/ADR-RES	110.56
SK-OV-3	102.71
**Renal Cancer**	
786-0	100.43
A498	107.40
ACHN	108.95
CAKI-1	86.78
RXF 393	112.71
SN12C	103.62
TK-10	103.40
UO-31	81.22
**Prostate Cancer**	
PC-3	102.72
DU-145	111.60
**Breast Cancer**	
MCF7	97.79
MDA-MB-231/ATCC	102.54
HS 578T	101.30
BT-549	111.67
T-47D	100.53
MDA-MB-468	107.75
Mean	102.92
Delta	21.70
Range	37.32

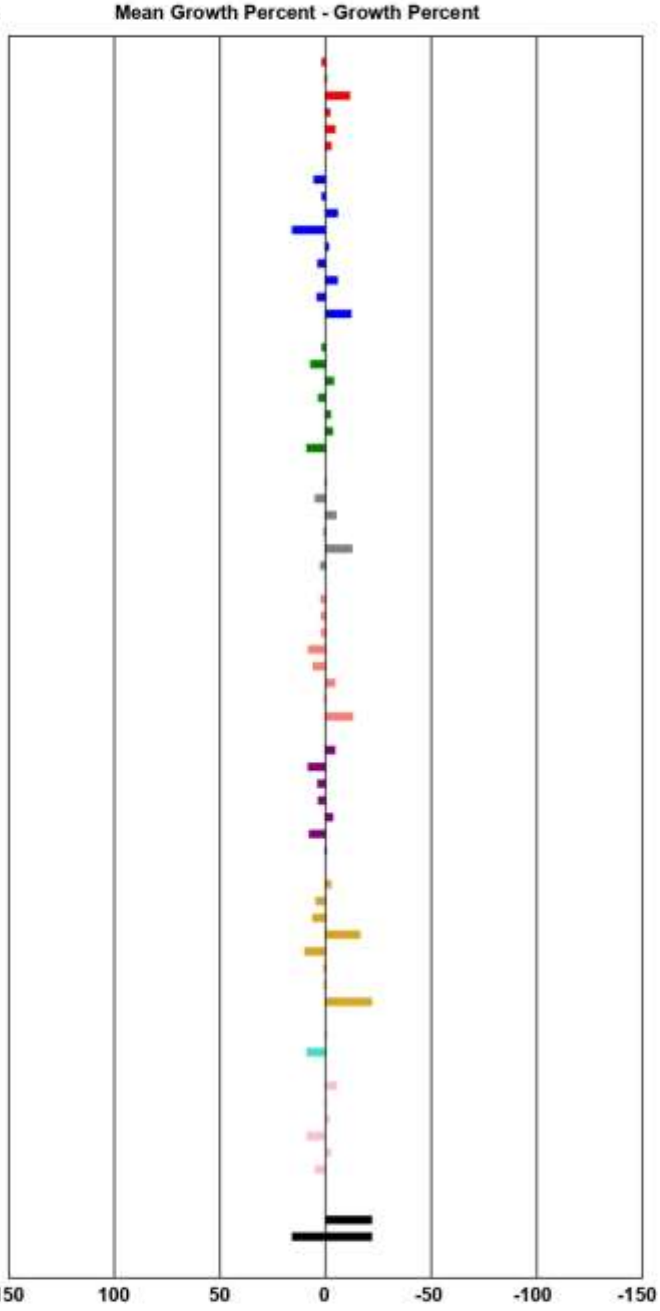

Figure S132. One dose mean graph for compound 8c (NSC 828470) at 10 µM

Information Classification: General

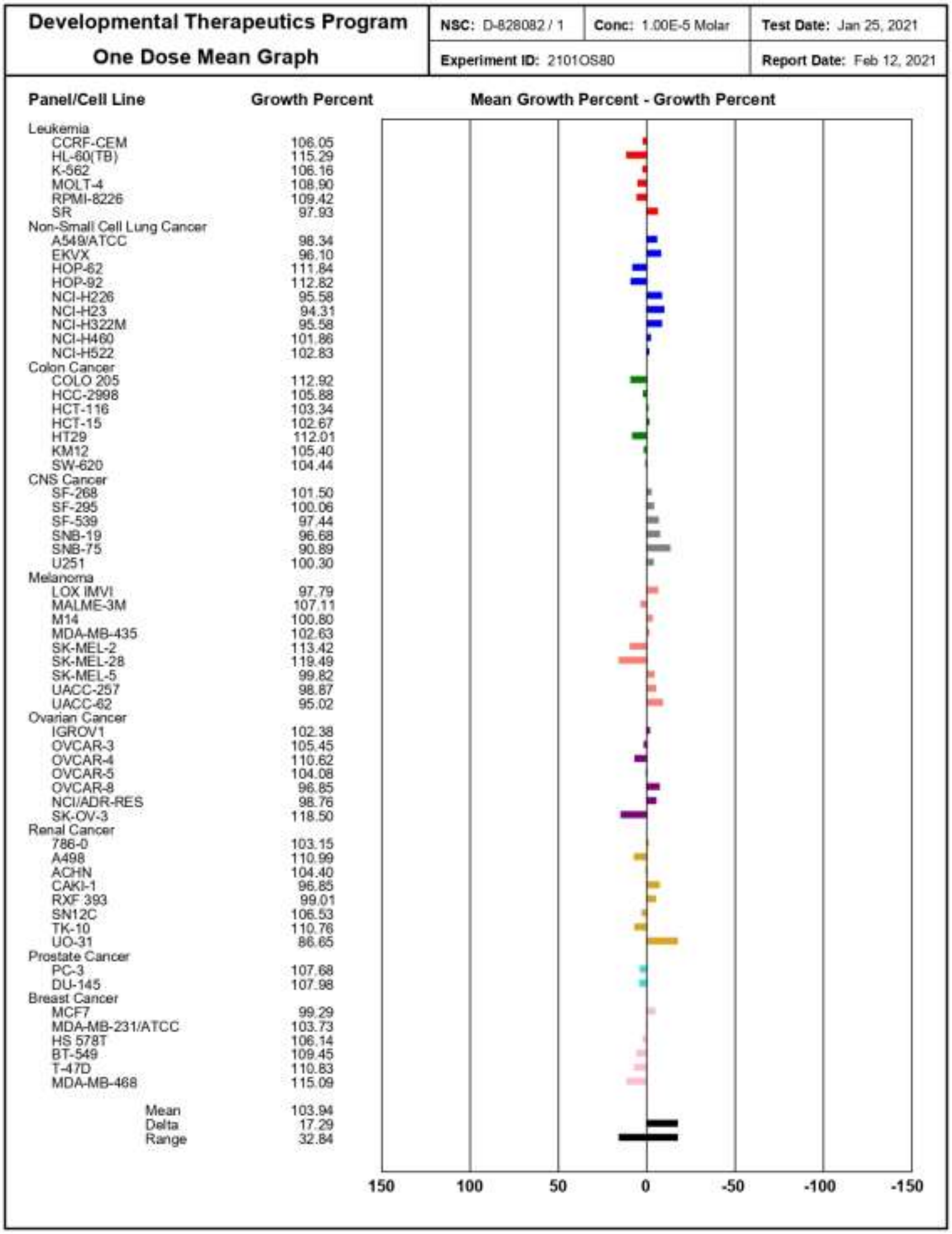

Figure S133. One dose mean graph for compound 8d (NSC 828082) at 10 μM

Information Classification: General

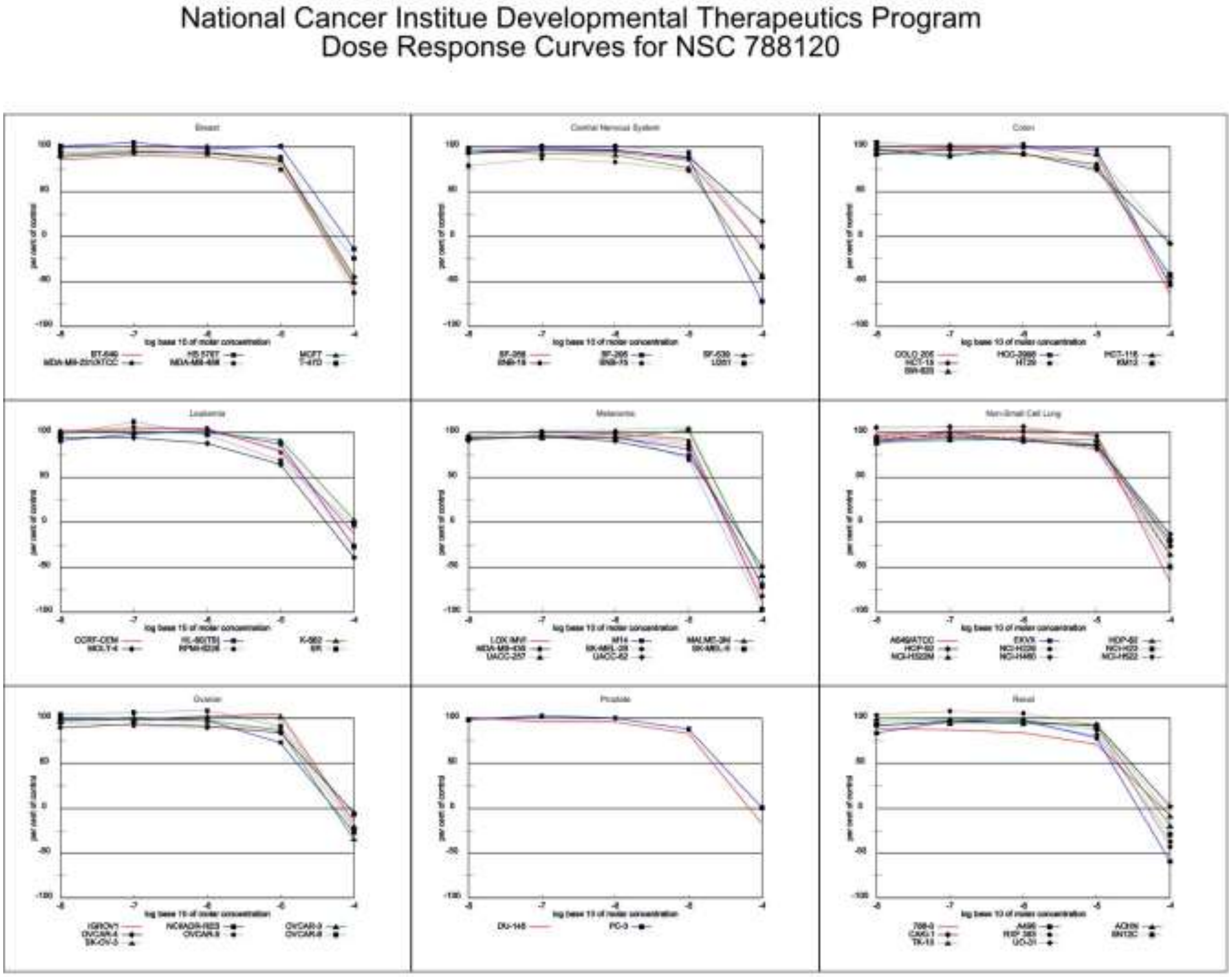

Figure S134. Dose-response curves (% growth versus sample concentration) for all cell lines with different subpanel obtained from the NCI's *in vitro* disease-oriented human cancer cells line for enasidenib on nine types of cancer

Information Classification: General

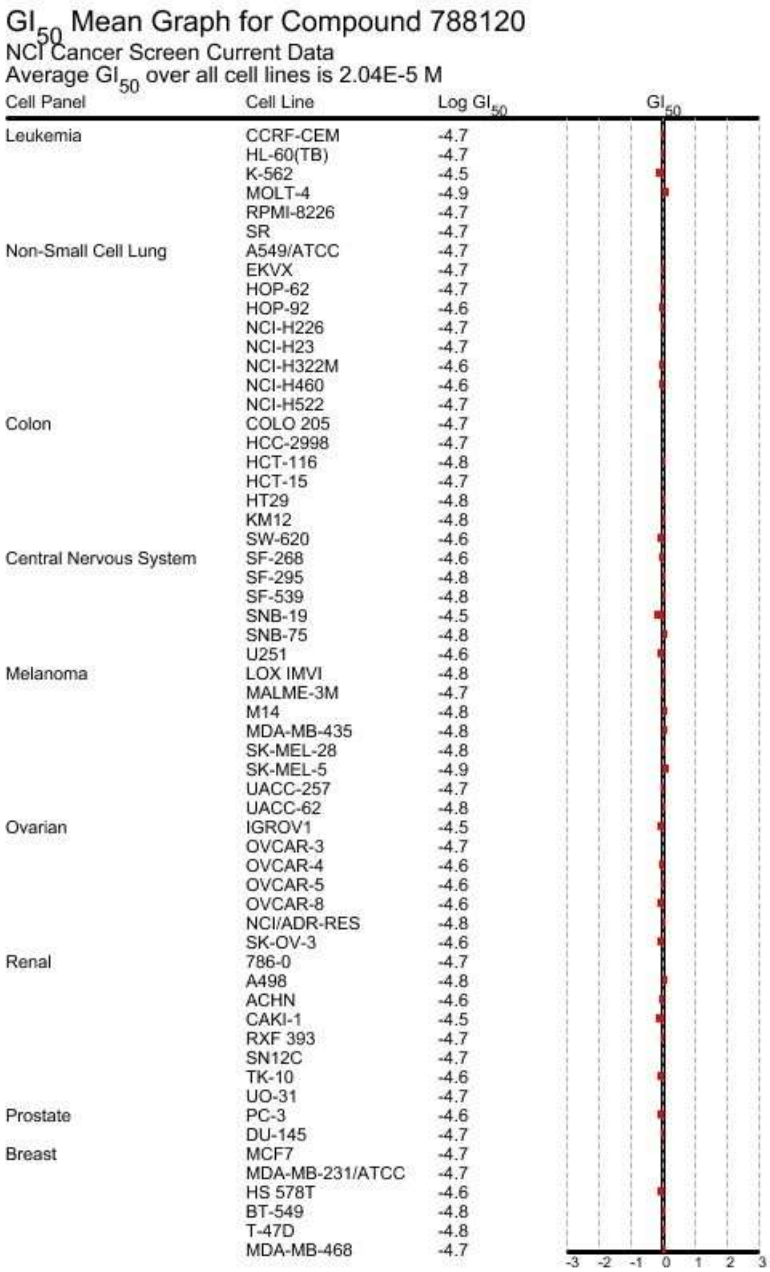
GI50 Mean Graph for Compound 788120

NCI Cancer Screen Current Data

Average GI50 over all cell lines is 2.04E-5 M

Cell Panel	Cell Line	Log GI50
Leukemia	CCRF-CEM	-4.7
	HL-60(TB)	-4.7
	K-562	-4.5
	MOLT-4	-4.9
	RPMI-8226	-4.7
	SR	-4.7
Non-Small Cell Lung	A549/ATCC	-4.7
	EKVX	-4.7
	HOP-62	-4.7
	HOP-92	-4.6
	NCI-H226	-4.7
	NCI-H23	-4.7
	NCI-H322M	-4.6
	NCI-H460	-4.6
	NCI-H522	-4.7
Colon	COLO 205	-4.7
	HCC-2998	-4.7
	HCT-116	-4.8
	HCT-15	-4.7
	HT29	-4.8
	KM12	-4.8
	SW-620	-4.6
Central Nervous System	SF-268	-4.6
	SF-295	-4.8
	SF-539	-4.8
	SNB-19	-4.5
	SNB-75	-4.8
	U251	-4.6
Melanoma	LOX IMVI	-4.8
	MALME-3M	-4.7
	M14	-4.8
	MDA-MB-435	-4.8
	SK-MEL-28	-4.8
	SK-MEL-5	-4.9
	UACC-257	-4.7
	UACC-62	-4.8
Ovarian	IGROV1	-4.5
	OVCAR-3	-4.7
	OVCAR-4	-4.6
	OVCAR-5	-4.6
	OVCAR-8	-4.6
	NCI/ADR-RES	-4.8
	SK-OV-3	-4.6
Renal	786-0	-4.7
	A498	-4.8
	ACHN	-4.6
	CAKI-1	-4.5
	RXF 393	-4.7
	SN12C	-4.7
	TK-10	-4.6
	UO-31	-4.7
Prostate	PC-3	-4.6
	DU-145	-4.7
Breast	MCF7	-4.7
	MDA-MB-231/ATCC	-4.7
	HS 578T	-4.6
	BT-549	-4.8
	T-47D	-4.8
	MDA-MB-468	-4.7

Figure S135. Mean Graphs of the log$_{10}$ values (Molar) of GI$_{50}$ obtained from the NCI 60 cell line experiments for enasidenib

Information Classification: General
